# High-throughput reprogramming of an NRPS condensation domain

**DOI:** 10.1038/s41589-023-01532-x

**Published:** 2024-02-02

**Authors:** Ines B. Folger, Natália F. Frota, Angelos Pistofidis, David L. Niquille, Douglas A. Hansen, T. Martin Schmeing, Donald Hilvert

**Affiliations:** 1 Laboratory of Organic Chemistry, ETH Zurich, Zurich, Switzerland; 2 Department of Biochemistry and Centre de Recherche en Biologie Structurale, McGill University, Montréal, Quebec Canada

**Keywords:** Biocatalysis, Natural products, Peptides

## Abstract

Engineered biosynthetic assembly lines could revolutionize the sustainable production of bioactive natural product analogs. Although yeast display is a proven, powerful tool for altering the substrate specificity of gatekeeper adenylation domains in nonribosomal peptide synthetases (NRPSs), comparable strategies for other components of these megaenzymes have not been described. Here we report a high-throughput approach for engineering condensation (C) domains responsible for peptide elongation. We show that a 120-kDa NRPS module, displayed in functional form on yeast, can productively interact with an upstream module, provided in solution, to produce amide products tethered to the yeast surface. Using this system to screen a large C-domain library, we reprogrammed a surfactin synthetase module to accept a fatty acid donor, increasing catalytic efficiency for this noncanonical substrate >40-fold. Because C domains can function as selectivity filters in NRPSs, this methodology should facilitate the precision engineering of these molecular assembly lines.

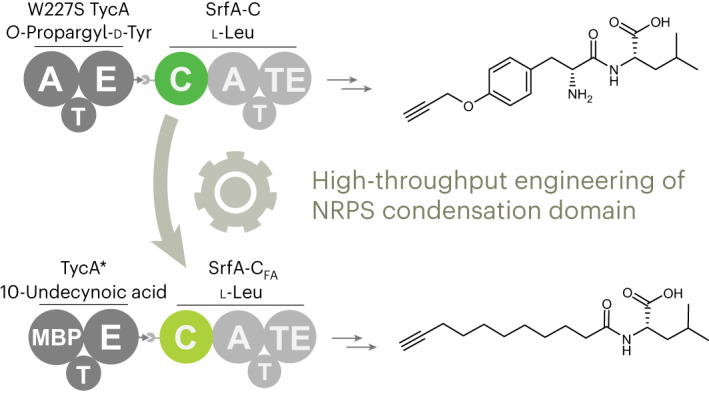

## Abstract (web summary)

Nonribosomal peptide synthetases produce valuable natural products but are challenging to engineer. Yeast surface display and fluorescence-activated cell sorting have now been combined to reprogram a condensation domain to recognize a noncanonical lipid substrate. This methodology may facilitate molecular tailoring of many biosynthetic assembly lines.

## Main

**Fig. 1:**
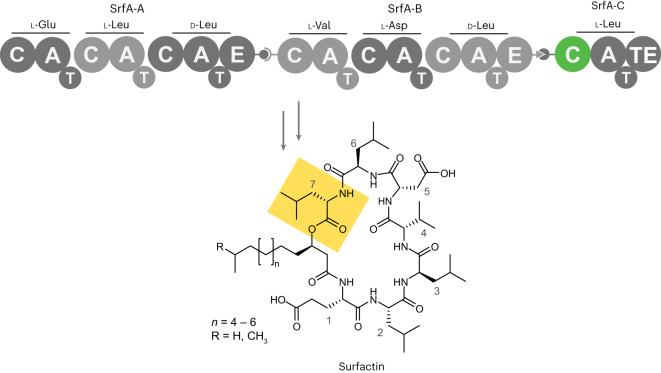
A typical NRPS. The enzymatic assembly line that produces the microbial lipopeptide antibiotic surfactin, surfactin synthetase, consists of the following three proteins: trimodular SrfA-A, trimodular SrfA-B and monomodular SrfA-C. The modules are composed of several functional domains, including C, A and T domains. In surfactin synthetase, biosynthesis is initiated by condensing a CoA-activated 3-hydroxy fatty acid to glutamate bound to the T domain of module 1. Each subsequent module performs peptide chain extension, and the final cyclic depsipeptide product is released by module 7’s TE domain. Epimerase (E) domains in modules 3 and 6 invert the chirality of the respective leucine residues. Two cognate pairs of docking domains (gray ball/socket and gray arrow/pacman representation) enable selective interaction between partner protein subunits. In the current study, we re-engineered the donor substrate specificity of the SrfA-C C domain (green) to enable direct acylation of the acceptor Leu (residue 7, yellow highlight) with a fatty acid rather than an amino acid.

Nonribosomal peptides (NRPs) represent a valuable source of clinical therapeutics^1,2^. These natural products are biosynthesized by multifunctional enzymatic assembly lines, called nonribosomal peptide synthetases (NRPSs), that use dedicated modules to incorporate each building block selectively and sequentially into the peptide scaffold (Fig. 1). NRPS modules typically possess three core domains, which perform substrate selection and activation (adenylation or A domains), shuttling of activated substrates (thiolation or T domains, also called peptide carrier proteins, PCP or CP domains) and peptide bond formation (condensation or C domains), respectively. Once product assembly is complete, the final peptide is typically released by a terminal thioesterase (TE) domain. The full NRPS assembly line may be a single megaenzyme containing all modules or may be divided into subunits that associate noncovalently through small ‘docking’ domains^3–5^.

The modularity of NRPSs is appealing from an engineering perspective^6–10^. Individual domains, modules and even entire proteins can be added, deleted or exchanged with counterparts from different pathways to create hybrid synthetases for the production of new antibiotics and other bioactive materials^11–19^. The specificities of gatekeeper A domains can also be reprogrammed to incorporate unnatural building blocks with unique properties^20–28^. Nevertheless, the success of such strategies is often limited. Exchanging subunits or modules can disrupt interdomain contacts, lowering product yields or abolishing activity^9,10^. Efficiency may be further reduced if other enzymes in the modified assembly line, particularly C domains^10,29,30^, fail to process noncognate substrates.

C domains are large enzymes (~450 amino acids), typically embedded in an NRPS protein or subunit^29,30^. They catalyze amide bond formation between an amino acid tethered to the downstream T domain and an amino acid or peptide tethered to the upstream T domain, leading to elongation of the growing peptide chain. Tolerance of C domains to noncanonical substrates can vary substantially. They are often selective with respect to the acceptor substrate^31–38^ and sometimes disfavor large changes in the donor substrate as well^25,38–41^. Indeed, distinct classes of C domains have evolved based on the nature of their donor substrate, including those specific for l-amino acids, d-amino acids and fatty acids^42^, although these proteins do not have discernibly different substrate binding pockets. Other C domains catalyze additional chemical reactions, including the dehydration of amino acids, to further boost the structural diversity of NRPS peptides^30,43–46^. The absence of general rules for deducing substrate specificity^29,30,47^ makes C-domain engineering more challenging than A-domain engineering. Compounding the problem, published C-domain assays^31–33,48,49^ have relatively low throughput, making the discovery of rare combinations of specificity-altering mutations a formidable, labor-intensive undertaking.

Here we describe a yeast display^50^ strategy that allows analysis and engineering of C-domain substrate selectivity in high throughput based on the functional display of an intact NRPS module on yeast. We used this system to reprogram the last C domain in surfactin synthetase to accept fatty acid substrates rather than amino acids. Strategic incorporation of such building blocks into NRP scaffolds could be used to target peptidyl drug candidates to cell membranes and thus enhance antimicrobial activity^51,52^. The ability to tune the properties of NRPS C domains has exciting potential to overcome many of the bottlenecks that have plagued previous efforts to adapt biosynthetic assembly lines for the sustainable production of non-natural products.

## Results

### High-throughput yeast display assay

**Fig. 2:**
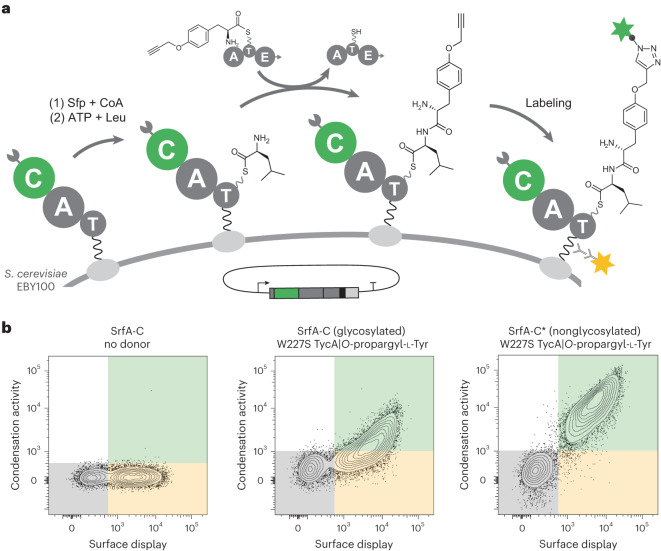
Assaying C-domain activity by yeast display. **a**, Schematic representation of enzymatic peptide bond formation on the surface of yeast. SrfA-C is displayed on the cell surface by fusion to the N-terminus of Aga2p via a short linker (black wavy line). The construct is primed with its ppant cofactor by the addition of CoA and the 4′-ppant transferase Sfp and loaded with the cognate amino acid, l-Leu, by action of the displayed A domain. Condensation is initiated by the addition of a donor module, W227S TycA, charged with *O*-propargyl-l-Tyr, in solution. Successful peptide bond formation is visualized by derivatizing the alkyne moiety in the dipeptidyl product with a biotin moiety using bioorthogonal click chemistry and adding fluorescently labeled streptavidin (R-PE, green star); a c-Myc epitope tag included in the linker was used to quantify display by immunofluorescence (FITC, yellow star). Active variants can then be isolated by FACS. The complementary docking domains are indicated by arrow and pacman appendages on the donor and acceptor domains, respectively. **b**, Flow cytometric analyses of SrfA-C modules on yeast (shown as contour plots with outliers (dots)). SrfA-C display is plotted (*x* axis, FITC) versus condensation activity (*y* axis, R-PE). Left, display of SrfA-C. Middle, yeast displaying glycosylated SrfA-C that were treated with 1.5 µM W227S TycA and 75 µM *O*-propargyl-l-Tyr for 15 min. Right, yeast displaying nonglycosylated SrfA-C* that were treated with 1.5 µM W227S TycA and 75 µM *O*-propargyl-l-Tyr for 15 min. The ‘SrfA-C no donor’ sample (left) was labeled with FITC and all other samples were labeled with FITC and R-PE immediately before analysis. The gray gate indicates cells that do not display any fluorescent label, the yellow gate indicates cells that show an FITC label and the green gate indicates cells that display both dyes. Source data

NRPS-catalyzed peptide condensation requires components of two NRPS modules because the natural substrates of C domains are donor and acceptor acyl-T domains. These T domains must be loaded with their cognate building blocks, either naturally by cognate A domains or using chemoenzymatic approaches^53,54^. To monitor C-domain activity, we envisaged displaying an entire NRPS module, comprising docking, C, A and T domains, in functional form on the surface of yeast, and adding an upstream module, together with the required substrates, in solution (Fig. 2a). Productive interaction of the two modules, made possible by matching docking domains^3–5^, would allow the C domain to catalyze amide bond formation between the substrate on the upstream module and the substrate on the displayed module. The resulting dipeptide product would be covalently linked to the T domain of the displayed module as a thioester and thus attached to the yeast surface. If the donor substrate contains an alkyne side chain, cells producing active C domains can be visualized by a bioorthogonal ‘click’ reaction and isolated by fluorescence-activated cell sorting (FACS).

We chose the SrfA-C module from surfactin synthetase for surface display. SrfA-C, which is well characterized functionally and structurally^55^, has the archetypal C, A and T domains, as well as a terminal TE domain that we omitted to prevent product off-loading after peptide bond formation. The core C–A–T domains of SrfA-C were fused to the yeast mating factor Aga2p to anchor the module to the yeast cell wall (Fig. 2a). A Myc tag was also included in the linker to allow determination of display efficiency by immunofluorescence labeling. Control experiments confirmed the successful expression and display of the NRPS module on EBY100 yeast cells (Fig. 2b). The T domain was subsequently modified post-translationally with phosphopantetheine (ppant) by addition of coenzyme A (CoA) and the 4′-ppant transferase Sfp^56^, enabling activation and transfer of the acceptor substrate, l-Leu, to the ppant cofactor by the displayed A domain.

SrfA-C is known to interact productively with the first module of tyrocidine synthetase A (TycA) via compatible docking domains and T:C interactions^3^. As the upstream module, we chose a previously engineered variant of TycA, W227S TycA^22^, which activates *O*-propargyl-l-Tyr. It was incubated with its substrate, ATP, Mg^2+^ and yeast cells displaying l-Leu-loaded SrfA-C for 15 min at room temperature. After washing the cells with phosphate buffer to remove the donor module, excess substrate and other reagents, the *O*-propargyl-d-Tyr-l-Leu product, tethered covalently to the ppant cofactor of the T domain on the yeast surface, was derivatized with biotin by copper(I)-catalyzed click chemistry^57,58^. Streptavidin modified with an R-phycoerythrin (R-PE) dye was added to visualize the labeled cells, which were analyzed by flow cytometry (Fig. 2b).

Although well-displayed, SrfA-C initially produced only modest yields of tethered dipeptide (Fig. 2b). Possible reasons for poor reactivity include loss of function of the displayed protein due to misfolding, product instability on yeast or unsuccessful docking of the upstream module with the displayed SrfA-C. Yeast, in contrast to many natural NRP producers, is also known to N-mannosylate proteins at Asn-Xaa-Ser/Thr sequences (where Xaa is any amino acid except Pro)^59^, which could adversely affect activity. To test whether SrfA-C is glycosylated, we reductively cleaved the displayed module from the yeast surface and analyzed it by mass spectrometry (MS). Tryptic digestion and peptide N-glycosidase F treatment indicated substantial *N*-glycosylation of Asn625 and Asn909 in the A domain of SrfA-C (Supplementary Fig. 1). To prevent this modification, we prepared a variant called SrfA-C* that has all potential *N*-glycosylation motifs disabled. Based on phylogenetic and structural analysis of similar A domains, residues Asn625, Ser787 and Asn909 were, respectively, mutated to Thr, Gln and Gln. SrfA-C*, in contrast to wild-type SrfA-C, efficiently formed the dipeptide on yeast when incubated with W227S TycA and substrates (Fig. 2b and Supplementary Fig. 2c). The signal was as high as for a positive control generated by directly ligating the authentic product precursor *O*-propargyl-d-Tyr-l-Leu-ppant to the displayed SrfA-C* module (Supplementary Fig. 2h), confirming that glycosylation had impaired A domain function in wild-type SrfA-C.

SrfA-C* docks with a variety of other NRPS donor modules as well, including TycA^βpY^ (TycA engineered to recognize *O*-propargyl-(S)-β-Tyr)^24^, W227S/H743A TycA (an E domain knockout)^60^ and W2742S ${{\rm{TycB}}3}^{{\rm{COM}}^{{\rm{D}}}{\rm{TycA}}}$ (an excised TycB3 variant equipped with the TycA docking domain)^3,23^, to generate yeast-displayed dipeptide products (Supplementary Fig. 2i–k). Negative controls with constructs lacking the ppant cofactor or inactivated by knockout mutations in the C or T domains (H147A/D151N SrfA-C* and S1003A SrfA-C*, respectively) could be displayed but did not form the dipeptide product (Supplementary Fig. 2d,e,g). Taken together, these results demonstrate that the C domain in the modified SrfA-C module effectively catalyzes dipeptide bond formation on yeast, covalently tethering the product to the displayed T domain.

### Engineering the SrfA-C C domain for fatty acid recognition

Although the terminal C domain in surfactin synthetase is known to tolerate some changes in its native peptide substrate^3,10,11^, we sought to expand its substrate scope to nonpeptidyl acyl donors. Incorporating lipids into NRP products is desirable because such moieties can be key to antimicrobial activity^51^. For example, the fatty acid component of the antibiotic daptomycin targets the NRP to bacterial cell membranes, resulting in microbial cell death^52^. Hence, C-domain-catalyzed incorporation of hydrophobic lipids into peptide scaffolds could be a general strategy to improve the pharmaceutical properties of many NRPs. Fatty acids are usually incorporated into NRPs by a specialized class of C domains called C_starter_ domains^42^, but we wondered whether yeast display could convert SrfA-C into a lipid-specific module.

To first test whether SrfA-C has any latent activity with inherently less reactive fatty acids, we examined its ability to process 10-undecynoic acid, which contains a terminal alkyne for bioorthogonal labeling. In the absence of an initiation module that can both activate this fatty acid and effectively interact with SrfA-C, we produced a modified TycA construct, TycA*, in which the A domain is replaced with maltose-binding protein (MBP) to improve solubility, and loaded the fatty acid onto its T domain using chemically synthesized 10-undecynoyl-pant in a one-pot enzymatic cascade (Supplementary Fig. 3)^53^. In vitro reaction of 10-undecynoyl-pant with the CoA biosynthetic enzymes pantothenate kinase (PanK), ppant adenylyltransferase (PPAT) and dephospho-coenzyme A kinase (DPCK) yielded 10-undecynoyl-CoA (liquid chromatography–high-resolution mass spectrometry (LC–HRMS (M − H)^−^): calculated, 930.2275; found, 930.2280). Incubating this mixture with TycA* in the presence of Sfp afforded 10-undecynoyl-ppant-TycA* (HRMS: calculated, 107,517.2 Da; found, 107,515.0 Da). Unmodified protein was not detected, indicating near quantitative modification of the TycA* construct with the lipid substrate. As expected, however, the thioester linking the fatty acid to the T domain is not particularly stable and was hydrolyzed completely after 1-h incubation in buffer at room temperature (Supplementary Note— High-resolution electrospray mass spectrometry (ESI–MS), TycA* primed with 10-undecynoyl-ppant).

When 10-undecynoyl-ppant-TycA* was incubated with yeast displaying SrfA-C*, trace 10-undecynoyl-l-Leu (**1**) was formed (Supplementary Fig. 2l), suggesting that the fatty acid is a poor substrate for the C domain. We quantified this activity in vitro using a bimodular assay, combining SrfA-C with 10-undecynoyl-ppant-TycA*, l-Leu, ATP and Mg^2+^ and monitoring the formation of **1** by LC–MS. Although activity was detected, the fatty acid was processed >15 times more slowly than *O*-propargyl-Tyr (Supplementary Fig. 4).

**Fig. 3:**
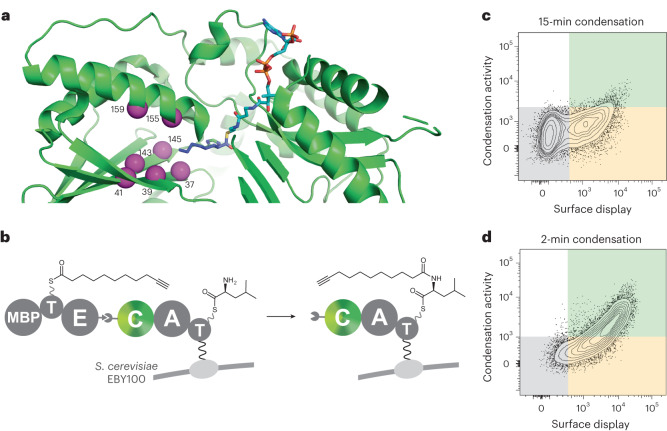
C-domain library design and sorting. **a**, Crystal structure of the SrfA-C C domain (PDB: 2VSQ, green cartoon)^55^ overlaid with the C8-CoA fatty acyl substrate of the RzmA crystal structure (PDB: 7C1S, cyan and blue sticks)^39^. The active site residue His147 (green sticks) is positioned in close proximity to the thioester of the fatty acyl substrate. Randomized residues Glu37, Ile39, Met41, Trp143, Tyr145, Phe155 and Val159 are shown as magenta spheres. **b**, Screening of a large C-domain library displayed on the surface of yeast for fatty acylation of l-Leu. The library of SrfA-C* variants was displayed on the cell surface and reacted with 10-undecynoyl-ppant-TycA*. Active C-domain variants were labeled as described in Fig. 2 and analyzed by FACS. **c**, Contour plot of the SrfA-C* C-domain library displayed on yeast and reacted for 15 min with 10-undecynoyl-ppant-TycA*. Cells displaying both fluorophores represent active variants that were sorted and enriched over five rounds of FACS with increasingly stringent conditions. **d**, Contour plot of the C-domain variant pool obtained after five rounds of sorting and enrichment, displayed on yeast and reacted for 2 min with 10-undecynoyl-ppant-TycA*. Surface display is shown on the *x* axis (FITC) and peptide bond formation on the *y* axis (R-PE). In gray, cells without PE and FITC labels; in yellow, cells that are labeled with FITC; in green, cells with both PE and FITC labels. Source data

To engineer the C domain of SrfA-C to accept fatty acyl donors efficiently, we designed a library based on structures of RzmA-Cs, a C_starter_ domain that initiates rhizomide A biosynthesis by acylation of a leucine with a short-chain fatty acid^39^. Although the C domains of RzmA and SrfA-C are only distantly related (20% sequence identity), they both have the classic C-domain fold^29^ (which superimpose with a root mean square deviation of 4.2 Å over 336 Cα atoms) and the conserved HHxxxDG active site motif. The lipid substrate of RzmA-Cs sits deep within a pocket in the N-terminal lobe of the C domain, and comparison of the structures suggested that expansion of the SrfA-C substrate pocket between the core β-sheet in the N-terminal lobe and the fourth α-helix, close to the active site, might improve 10-undecynoic acid recognition. We therefore prepared a large combinatorial library in which hydrophobic (Ile39, Met41, Trp143, Tyr145, Phe155 and Val159) and negatively charged (Glu37) residues lining this site were simultaneously randomized using degenerate codons that code for small and hydrophobic amino acids (Fig. 3a and Supplementary Table 1).

The resulting library, which contained 1.5 × 10^6^ different SrfA-C variants, was displayed on yeast and screened for activity with 10-undecynoyl-ppant-TycA* by FACS. To identify the most active catalysts in the population, the cells were sorted and enriched over five consecutive cycles, increasing stringency by reducing the reaction time (Fig. 3 and Supplementary Fig. 5). Sequencing 15 variants from the final cell pool yielded eleven distinct sequences possessing 3–7 mutations, which cluster into four closely related groups (Supplementary Fig. 6).

### Biochemical characterization of the reprogrammed C domain

**Fig. 4:**
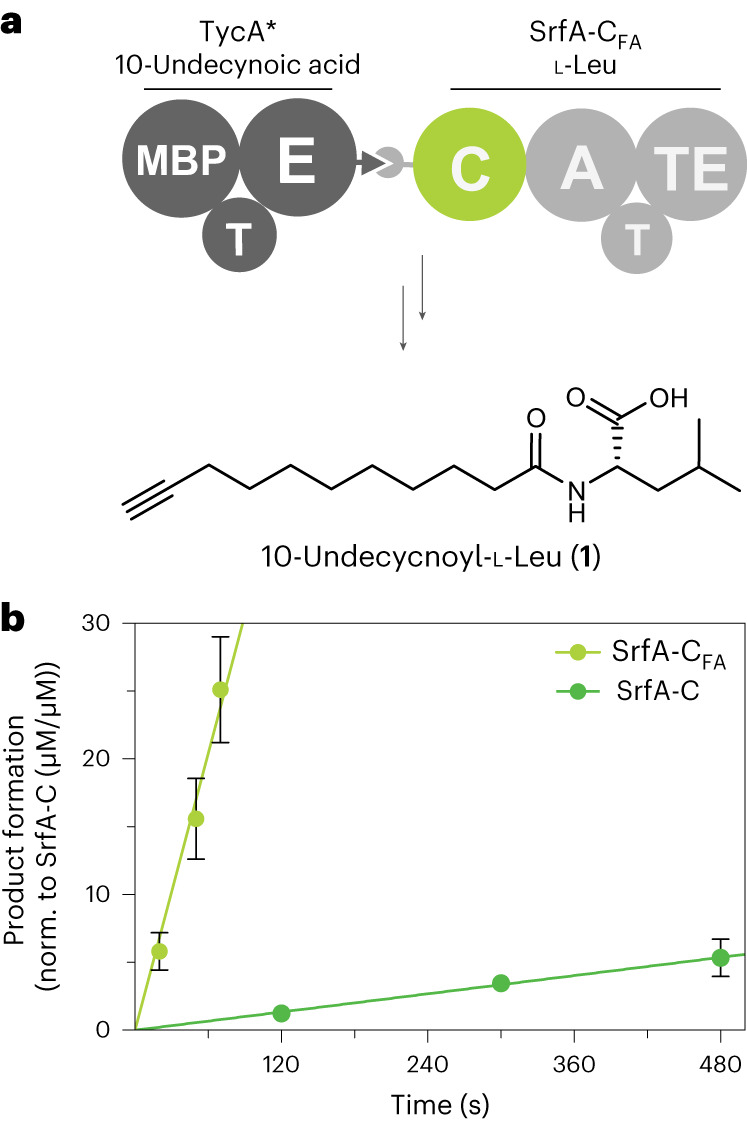
Kinetic characterization of SrfA-C_FA_. **a**, Enzymatic fatty acylation of l-Leu catalyzed by SrfA-C and SrfA-C_FA_ was performed in vitro by combining 10-undecynoyl-ppant-TycA* with SrfA-C_FA_, l-Leu, ATP and Mg^2+^ to produce 10-undecynoyl-l-Leu (**1**). **b**, Reaction kinetics for the biosynthesis of 10-undecynoyl-l-Leu (**1**). Product formation over time is shown for the bimodular synthetase TycA*/SrfA-C_FA_ (lime) and TycA*/SrfA-C wild type (green), normalized to 1 μM SrfA-C and SrfA-C_FA_, respectively. Each data point and corresponding error bar represent the average ± s.d. of triplicate measurements with a single biological sample (*n* = 1). Four biological replicates (*n* = 4) were used to determine apparent rate constants. Source data

Based on flow cytometric analysis of selected clones from sort 5 (Supplementary Fig. 7), we produced three SrfA-C variants (lacking the glycosylation mutations and reequipped with the TE domain) in *Escherichia coli* HM0079 for study in vitro. The purified proteins were kinetically characterized by mixing with 10-undecynoyl-ppant-TycA*, l-Leu, ATP and Mg^2+^, and monitoring the formation of 10-undecynoyl-l-Leu (**1**) by LC–MS (Fig. 4). Variant 10, harboring mutations W143T, Y145V and F155I, was found to be the best catalyst for rapid fatty acylation of l-Leu (Fig. 4 and Supplementary Figs. 4 and 8), giving an apparent rate constant of 38 ± 7 min^−1^. We call this enzyme SrfA-C_FA_. For comparison, wild-type SrfA-C is >40-fold less active under the same conditions, with an observed rate constant (*k*_obs_) of 0.91 ± 0.09 min^−1^.

Improved processing of the fatty acid by SrfA-C_FA_ also manifests in higher amounts of total product formed during the reaction (210 ± 40 versus 19 ± 6 total turnovers for SrfA-C_FA_ and SrfA-C, respectively; Supplementary Fig. 9). However, the reaction does not run to completion, presumably due to the limited stability of the 10-undecynoyl-ppant-TycA* thioester in the reaction buffer (Supplementary Fig. 9 and Supplementary Note). Consistent with this hypothesis, longer pre-incubation of the chemoenzymatic reaction mixture used to prepare 10-undecynoyl-ppant-TycA* decreased the amount of product formed, whereas three additions of freshly prepared 10-undecynoyl-ppant-TycA* to the in vitro reaction mixture increased product formation by 75%.

Interestingly, SrfA-C_FA_ shows twofold higher activity than wild-type SrfA-C with *O*-propargyl-l-Tyr (Supplementary Fig. 4). The side chains of *O*-propargyl-l-Tyr and 10-undecynoic acid are of similar length and enlarging the binding pocket of SrfA-C_FA_ presumably allows it to accommodate *O*-propargyl-l-Tyr better than the wild-type C domain. In the reactions with *O*-propargyl-l-Tyr, the donor substrate is catalytically regenerated, so considerably higher turnover numbers (1,900 ± 200) are also achieved. This suggests that an engineered TycA variant capable of catalytically reloading the lipid substrate would improve lipidation efficiency substantially.

### Transplantation of the engineered C domain

Swapping individual catalytic domains is a useful means of diversifying biosynthetic assembly lines^6–10^. We therefore asked whether the engineered C domain could replace the first C domain in tyrocidine synthetase^61^, which is located in the first module (TycB1) of the trimodular TycB protein. TycB1 naturally interacts with TycA and couples the upstream and downstream substrates d-Phe and l-Pro, so we expected that a modified TycB1 module containing the fatty acid-specific C domain would similarly engage productively with 10-undecynoyl-ppant-TycA* to afford lipidated products. For the swap, we chose domain boundaries based on a recently identified permissive recombination site between adjacent C and A domains^18^ and inserted the SrfA-C_FA_ C domain (residues Gln10–Gln430) between residues Val9 and Asn434 of TycB1 in a standalone C–A–T elongation module (TycB1_FA_) and in the full-length TycB protein (TycB_FA_).

**Fig. 5:**
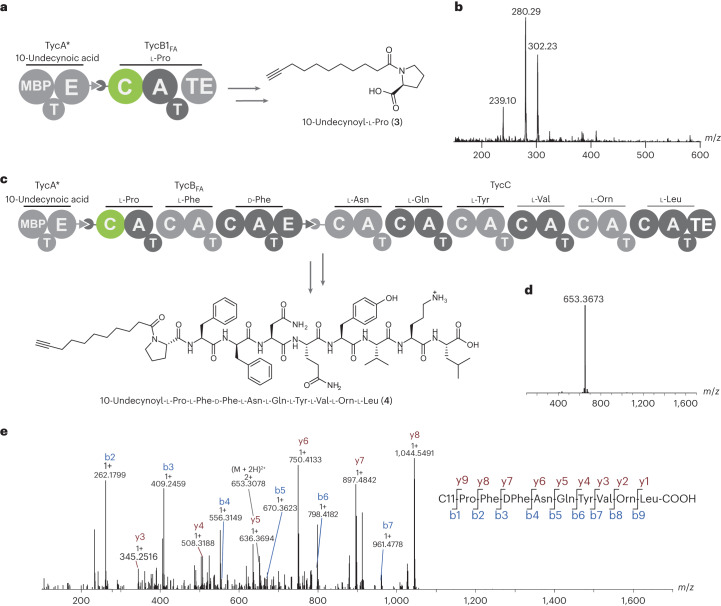
Biosynthesis of a nonribosomal lipopeptide by an engineered tyrocidine synthetase. The first C domain in tyrocidine synthetase was exchanged with the reprogrammed C domain of SrfA-C_FA_. **a**, The bimodular synthetase TycA*/TycB1_FA_ produces 10-undecynoyl-l-Pro (**3**) when supplied with 10-undecynoyl-ppant-TycA*, l-Pro, ATP and Mg^2+^. **b**, Product formation was confirmed by LC–MS. ((M + H)^+^: calculated, 280.19; found, 280.29; (M + Na)^+^: calculated, 302.17; found, 302.23; the species 239.10 corresponds to (M + H)^+^ of HEPES.) **c**, An engineered tyrocidine synthetase was generated by mixing 10-undecynoyl-ppant-TycA* and TycB_FA_ with TycC, producing a new lipopeptide 10-undecynoyl-l-Pro-l-Phe-d-Phe-l-Asn-l-Gln-l-Tyr-l-Val-l-Orn-l-Leu (**4**). **d**, LC–HRMS/MS analysis: mass spectrum for the doubly charged lipopeptide **4** ((M + 2H)^2+^: calculated, 653.3657; found, 653.3673). **e**, MS/MS spectrum of product **4**. Y ions are shown in brown and b ions in blue; respective charge and mass are indicated. All data are from one biological sample (*n* = 1). Chromatograms of LC–MS and LC–HRMS/MS analyses can be found in Supplementary Fig. 10. Source data

As anticipated, incubation of the standalone TycB1_FA_ module with 10-undecynoyl-ppant-TycA* in the presence of l-Pro, ATP and Mg^2+^ yielded 10-undecynoyl-l-Pro (**3**; Fig. 5a,b and Supplementary Fig. 10a). LC–MS analysis indicated that the TycB1_FA_-catalyzed 10-undecynoylation of l-Pro is only approximately fourfold less efficient than the SrfA-C_FA_-catalyzed reaction with l-Leu. Suboptimal domain boundaries between the swapped C and A domains, low tolerance for l-Pro at the acceptor site of the engineered C domain or slow off-loading of the product by the TE domain could easily account for this small decrease.

Similarly, when we combined 10-undecynoyl-ppant-TycA* with trimodular TycB_FA_ and the hexamodular TycC protein in the presence of all the required substrates (Fig. 5c), 10-undecynoyl-l-Pro-l-Phe-D-Phe-l-Asn-l-Gln-l-Tyr-l-Val-l-Orn-l-Leu (**4**) was formed, as detected by LC–MS and confirmed by LC–HRMS/MS (Fig. 5d,e and Supplementary Fig. 10b). Although further engineering will be required to optimize the efficiency of this artificial NRPS, the biosynthetic production of a new lipopeptide is notable considering that the system was not optimized for coupling a fatty acid to Pro or for the substrate preferences of other downstream domains. For the in vivo production of such peptides, we would also need to replace the preloaded TycA* donor module with a catalytic module evolved to activate fatty acids directly, for example, using the previously reported yeast display system to alter the substrate specificity of TycA^24^. Alternatively, the C domain could be evolved to react directly with CoA-activated fatty acid derivatives that can be produced in cells by fatty acyl-CoA ligases^62^.

### Structural characterization

**Fig. 6:**
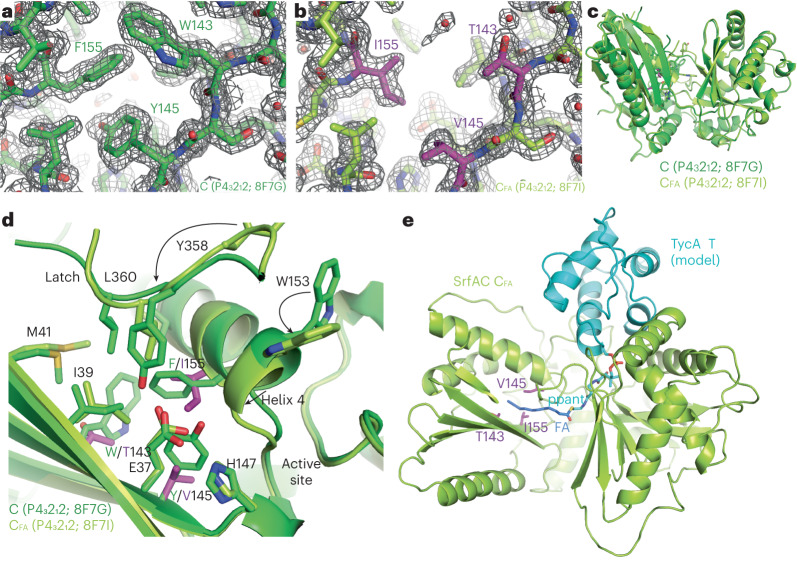
Structural analysis of the wild-type SrfA-C and SrfA-C_FA_ C domains. **a**, A 2F_O_–F_C_ electron density map, contoured at 1σ, of the wild-type SrfA-C C domain (green). **b**, The analogous 2F_O_–F_C_ electron density map, contoured at 1σ, of the fatty acid-specific C domain of SrfA-C_FA_ (light green with mutated residues in magenta). **c**,**d**, Overlay of the C domains of wild-type SrfA-C (green) and SrfA-C_FA_ (light green with mutated residues in magenta). **e**, An energy-minimized co-complex model of a 10-undecynoyl-ppant-T_TycA*_ homology model bound to the C domain of SrfA-C_FA_.

To gain insight into how the engineered SrfA-C_FA_ variant recognizes and processes a lipid substrate, we attempted to crystallize its C domain in complex with substrate analogs. Two unrelated, highly diffracting crystal forms of the SrfA-C_FA_ C domain were obtained and used to solve apo structures to high resolution (Fig. 6 and Supplementary Table 2). We performed cocrystallization and soaking with 10-undecynoyl-pantetheinamide (10-undecynoyl-NH-pant) and 10-undecynoyl-NH-CoA, nonhydrolyzable small molecule analogs of the donor substrates, but as reported for other C domains^36^, density for the ligands was not observed. We also undertook crystallization of the C domain covalently fused to the upstream donor complex, the 10-undecynoyl-NH-ppant-T domain, but crystals of the didomain did not grow. However, we also solved the high-resolution structures of wild-type SrfA-C, and comparison with SrfA-C_FA_ structures provides insight into the origins of the specificity shift (Fig. 6a,b and Supplementary Table 2).

The structures of the SrfA-C C domain show the classic pseudodimeric V-shape and are very similar to each other and to the C domain in the previously characterized full-length SrfA-C module (Protein Data Bank (PDB): 2VSQ (ref. ^55^); Supplementary Fig. 11a). Small differences in side chain conformations of residues lining the putative fatty acyl binding site are observed between our wild-type structures and 2VSQ (Supplementary Fig. 11b), and the adjacent segment of the latch loop, which is poorly ordered in 2VSQ, is also disordered. In the SrfA-C_FA_ structures, all mutations are clearly visible in electron density maps (Fig. 6a,b) and introduce more space between the middle of helix 4 and the β sheet (Supplementary Fig. 11c,d). Introduction of the W143T, Y145V and F155I mutations causes the following three connected changes (Fig. 6c,d): (1) the N-terminus of helix 4 shifts inward, toward the pocket mutations, by up to 2 Å; (2) the helix’s capping residue, Trp153, swings over to interact with the base of the latch; and (3) the latch shifts to pack Leu360 into the mutated pocket. However, in this conformation, the active site is not connected to the expanded mutated pocket, mainly because of the position of Glu37 and Leu360. Nevertheless, a reasonable 10-undecynoyl-ppant-TycA* model can be made that positions the distal end of the 10-undecynoyl moiety into the expanded pocket (Fig. 6e). Although this model is consistent with the mutations improving activity, cocrystal structures will ultimately be required to corroborate the proposed binding mode.

## Discussion

Customization of NRPS assembly lines for the biocatalytic production of bioactive peptide derivatives represents an attractive green alternative to chemical synthesis^6–10^. Nevertheless, despite some notable successes in reprogramming A domain specificities^20–28^ and swapping individual domains and modules^11–19,41,63^, disruption of critical domain interactions often introduces new bottlenecks in the resulting constructs that limit overall efficiency^9,10^. The substrate specificities of C domains^10,29^ further complicate these engineering efforts.

New strategies for bioengineering NRPSs that feature nontraditional exchange points^16,17^ and/or ‘synthetic zippers’ as artificial docking domains^64,65^ have been developed and shown in some systems to overcome the severe drop in productivity often seen in classical NRPS engineering approaches. Harnessing combinatorial mutagenesis and high-throughput screening as described in the current study represents both a powerful alternative for addressing such problems and a complementary tool that can be applied to engineered systems to increase their productivity. By displaying full-length NRPS modules on the surface of yeast, the condensation activity of the displayed module can be directly linked to a simple fluorescent readout that enables flow cytometric screening of libraries containing up to 10^8^ different variants^66,67^. Successful reprogramming of the SrfA-C C domain for coupling a fatty acid substrate with l-Leu highlights the efficacy of this approach. The >40-fold enhancement in fatty acylation obtained after a single round of mutagenesis and screening shows that C domain tolerance can be effectively expanded to building blocks substantially different from the native substrate. Moreover, the twofold improvement in *O*-propargyl-d-Tyr processing indicates that catalytic rates can be enhanced for multiple substrates simultaneously, broadening the substrate scope of C domains without compromising catalytic efficiency.

The small number of closely related variants found to catalyze the new lipidation reaction underscores the importance of screening large combinatorial libraries over rational design. Structure-based considerations would certainly have included mutation of Glu37 to a smaller residue, as the side chain of Glu37 blocks the fatty acid pocket in all crystal structures. However, such a substitution was not found in the best C-domain variant. Indeed, connectivity to the expanded binding pocket is not obvious in the structures of SrfA-C_FA_, so although we can rationalize why this variant is more advantageous than the wild type, we would not have predicted the successful mutations a priori. Interrogating each of the target residues individually using conventional low-throughput C-domain assays, and combining beneficial mutations to find the best variant, would also have been a comparatively arduous undertaking.

In principle, any NRPS module that can be displayed in functional form on yeast will be engineerable by an analogous mutagenesis and screening strategy. Although sequence optimization may be required to prevent post-translational glycosylation or other problems that adversely affect catalytic activity, as seen for SrfA-C, a wide range of bacterial and fungal proteins should be amenable to this approach. This capability opens the door to systematic, high-throughput exploration of the poorly understood molecular recognition determinants that underlie substrate selection by C domains. Although our study focused on repurposing the binding pocket for the donor substrate, chemoenzymatic loading of the displayed A domain with a range of amino acids would similarly allow the acceptor binding site to be probed and remodeled. Beyond engineering C-domain specificity, adapting the yeast display strategy described here to gain deeper insight into the catalytic mechanism of these enzymes and to optimize critical T–C, C–A and docking domain interfaces should be relatively straightforward.

In sum, yeast surface display of functional elongation modules provides a robust method for studying and rapidly engineering C domains, the catalytic engines of NRPS assembly lines. Together with an analogous high-throughput assay for altering A domain specificity^24,25^, high-throughput C-domain engineering lays the foundation for creating increasingly complex NRPS assembly lines via scalable, sustainable and minimally invasive methods. This enabling methodology paves the way for the effective biosynthesis of life-saving therapeutic agents and other applications in synthetic biology.

## Methods

### Materials

Tyrocidine synthetase proteins and variants were produced in *E. coli* and purified as previously described^23^. Sfp and the CoA biosynthetic enzymes, PanK, PPAT and DPCK, were also produced in *E. coli* and purified using similar procedures. See Supplementary Fig. 12 for SDS–PAGE analysis of these proteins. For details on the synthesis of 10-undecynoyl-pantetheine, amino acid substrates and lipidated products, see Supplementary Note—Materials and methods.

### Cloning of SrfA-C variants for yeast surface display

The plasmid pCT_tycA_AT^24^ was modified for the display of SrfA-C variants. Five fragments were amplified by PCR. Fragments 1 and 2, encoding the signal peptide and linker region, were produced from pCT_tycA_AT and primer pairs SP_f/SP_r and linker_f/linker_r, respectively. The third fragment, encoding SrfA-C without the TE domain, was amplified from genomic DNA with the primer pair srfC_f/srfC_r. Fragments 4 and 5, encoding Aga2p and a portion of the vector backbone, were generated from pCT_tycA_AT using the primer pairs aga2p_f/aga2p_r and vector_f/vector_r, respectively. Fragments 1–4 and 3–5 were assembled via overlap PCR with the primer pairs SP_f/aga2p_r and linker_f/vector_r, respectively. Fragments 1–4 were digested with EcoRI and XhoI, fragments 3–5 with XhoI and XbaI and pCT_tycA_AT with EcoRI and XbaI, and the three components were subsequently ligated to yield pCTDN_srfA-C. For cloning purposes, a SalI restriction site was subsequently introduced behind the C-domain coding region. Two fragments were amplified from the plasmid using primers P1/P5 and P6/H33, reassembled by overlap PCR using P1/H33, digested with NheI and XhoI and ligated into the backbone of pCTDN_srfA-C that had been digested with the same restriction enzymes to give the display plasmid pCT_srfA-C. To prevent post-translational glycosylation of SrfA-C in yeast, three point mutations (N625T, S787Q and N909Q) were introduced into the sequence encoding the A domain using overlap PCR and Gibson assembly with the primer pairs R7/P19, P18/P23, P22/P26 and P25/H33 to give pCT_srfA-C*. For detailed cloning procedures, see Supplementary Note—Materials and methods. Primer sequences are listed in Supplementary Table 3.

### Yeast display of SrfA-C and variants

Yeast surface display was adapted from the published protocol discussed in ref. ^50^ using optimized cell growth conditions, incubation times and substrate concentrations. pCT_srfA-C and pCT_srfA-C* were used to transform *Saccharomyces cerevisiae* EBY100 cells using the Frozen-EZ Yeast Transformation II Kit (Zymo Research) according to the manufacturer’s protocol. Transformed cells were plated on synthetic dextrose–casein amino acids (SD–CAA) plates (250 ml; 20 g l^−1^ d-(+)-glucose, 1.7 g l^−1^ Difco yeast nitrogen base without amino acids and ammonium sulfate (BD Biosciences), 0.5 g l^−1^ ammonium sulfate, 5 g l^−1^ Difco casamino acids (BD Biosciences), 40 mM Na_2_HPO_4_, 70 mM NaH_2_PO_4_, 182 g l^−1^ sorbitol, 16 g l^−1^ agar, pH 6) and incubated at 30 °C for 2–3 d. A preculture of a single clone was prepared in 2 ml SD–CAA medium (20 g l^−1^ d-(+)-glucose, 1.7 g l^−1^ Difco yeast nitrogen base without amino acids and ammonium sulfate (BD Biosciences), 0.5 g l^−1^ ammonium sulfate, 5 g l^−1^ Difco casamino acids (BD Biosciences), 40 mM Na_2_HPO_4_, 70 mM NaH_2_PO_4_, pH 6) and grown overnight (18–20 h) at 30 °C and 260 r.p.m. The preculture was subsequently diluted to optical density (OD)_600_ = 0.2 in 3 ml SD–CAA and regrown at 30 °C and 260 r.p.m. for 7–9 h until an OD_600_ of 2–4 was reached. The fresh culture was then diluted again to OD_600_ = 0.2 and stored for up to 2 d at 4 °C. For protein induction and surface display, the cell culture was regrown for 6 h at 30 °C and 260 r.p.m. and centrifuged at 500*g* at 4 °C for 3 min. The cell pellet was resuspended in 3 ml SG–CAA medium (20 g l^−1^ d-(+)-galactose, 1.7 g l^−1^ Difco yeast nitrogen base without amino acids or ammonium sulfate (BD Biosciences), 0.5 g l^−1^ ammonium sulfate, 5 g l^−1^ Difco casamino acids (BD Biosciences), 40 mM Na_2_HPO_4_, 70 mM NaH_2_PO_4_, pH 6) and shaken for 21–23 h at 20 °C and 230 r.p.m.

The efficiency of SrfA-C display on EBY100 was assessed by immunofluorescence labeling with the primary mouse anti-c-Myc antibody 9E10 (Roche, ROAMYC; final concentration 300 ng μl^−1^) and the secondary anti-mouse IgG-fluorescein isothiocyanate (FITC) antibody F2012 (Sigma-Aldrich; final concentration: 65 ng μl^−1^) in 10 μl permeabilization buffer (PMB; 7.2 mM NaH_2_PO_4_, 40 mM Na_2_HPO_4_, 137 mM NaCl, 2.7 mM KCl, 1 mM MgCl_2_, pH 7.4) per 100 μl cells^25^. Flow cytometric analysis was performed on an LSRFortessa Cell Analyzer (BD Biosciences) at the Flow Cytometry Core Facility of ETH Zurich. Display of SrfA-C constructs on yeast usually varied between 50% and 80% and was dependent on induction time as well as cell viability.

### LC–MS/MS analysis of yeast-displayed SrfA-C

Display of SrfA-C on EBY100 was performed as described above using 35 ml cell culture. After 23 h induction, the cells were centrifuged for 3 min at 5,000*g* at 4 °C and washed a total of four times with 50 ml PMB. The cell pellet was resuspended in 1 ml PMB containing 1 mM tris(2-carboxyethyl)phosphine (TCEP) and incubated at 4 °C for 25 min. The supernatant was transferred to a fresh tube and protein was concentrated in a Vivaspin 500 centrifugal concentrator. The sample was analyzed by SDS–PAGE using a Phast system and 7.5% gels (GE HealthCare) following the manufacturer’s protocols (Supplementary Fig. 1).

For LC–MS/MS analysis by the Functional Genomics Center Zurich, the sample was digested with trypsin. Specifically, 10 μl sample was diluted to 90 μl with 50 mM triethylammonium bicarbonate buffer (TEAB), and 100 μl of 20% trichloroacetic acid was added. The sample was washed twice with acetone, dissolved in 45 μl (50 mM) TEAB (pH 8.5) mixed with 0.9 μl TCEP (2 mM) and 1.4 μl 15 mM chloroacetylacetone, and incubated for 30 min at 60 °C. Subsequently, 5 μl of a trypsin stock solution (100 ng μl^−1^) was added, and the proteins were digested overnight. The sample was dried and subsequently dissolved in 20 μl (0.1%) formic acid.

For de-glycosylation of the digested sample, half of the solution was dried, redissolved in 20 μl (25 mM) ammonium bicarbonate buffer (pH 8.5), mixed with 1 μl PNGase F (1 U μl^−1^) and incubated overnight at 37 °C. The sample was subsequently dried and redissolved in 20 μl (0.1%) formic acid. The trypsin-digested and glycosylated and de-glycosylated peptide samples were analyzed by LC–MS/MS, injecting 2 μl on a nanoAcquity ultra-performance liquid chromatography system coupled to a Q Exactive mass spectrometer and analyzed using the software PEAKS^68^.

### Assaying peptide bond formation on yeast

To equip the displayed T domains with the ppant cofactor, 100 µl induced cells displaying SrfA-C or SrfA-C* were collected by centrifugation at 700*g* for 30 s at room temperature, washed 2–3 times with 90 µl PMB, resuspended in a total volume of 25 µl PMB supplemented with 4 µM Sfp, 500 µM CoA and 1 g l^−1^ BSA and incubated at room temperature for 20–25 min. The acceptor substrate was loaded onto the ppant cofactor by the displayed A domain in the same or in a separate step using 2 mM Leu and 100 µM ATP. To initiate peptide formation, the cells were washed with 90 µl PMB, collected and resuspended in 25 µl assay buffer (100 mM HEPES, 100 mM NaCl, 50 mM ATP, 10 mM MgCl_2_, pH 7.25) supplemented with 1.5 µM W227S TycA and 75 µM *O*-propargyl-l-Tyr and incubated at room temperature for 15 min. The cells were collected and washed with 90 µl PMB, and the cell pellet was resuspended in 25 µl PMB, supplemented with freshly prepared 4 mM ascorbate, 200 µM BPSA, 100 µM CuSO_4_ and 20 µM N_3_-PEG_3_-biotin (Sigma-Aldrich), and incubated for 15 min at room temperature to biotinylate any propargylated dipeptide product tethered to the yeast surface. The cells were collected and washed with 90 µl PMB. The cells were labeled with 300 ng μl^−1^ anti-c-Myc antibody 9E10 in 10 μl PMB, 65 ng μl^−1^ anti-mouse IgG-FITC antibody F2012 and 65 ng μl^−1^ streptavidin-R-PE (Thermo Fisher Scientific, S866) in 10 μl PMB^25^. The cells were collected, washed twice, resuspended in 400 µl PMB, transferred to FACS tubes and analyzed by flow cytometry as before.

As a positive control for maximal signal, the authentic dipeptide product was loaded directly onto the displayed SrfA-C* construct by treating the induced cells with 500 µM *O*-propargyl-d-Tyr-l-Leu-pant, the enzymes PPAT (final concentration: 45 µM), DPCK (45 µM) and PanK (45 µM), and Sfp (11 µM) in assay buffer for 25 min at room temperature. Biotinylation, fluorescent labeling and flow cytometric analysis were performed as described above.

Experiments with the alternative donor modules W2742S ${{\rm{TycB}}3}^{{\rm{COM}}^{{\mathrm{D}}}-{\rm{TycA}}}$, which also activates *O*-propargyl-d-Tyr ^23^; W227S/H743A TycA, which is specific for *O*-propargyl-l-Tyr^60^; and TycA^βpY^, which activates *O*-propargyl-(S)-β-Tyr^24^, were performed analogously.

### Preparation of 10-undecynoyl-ppant-TycA*

TycA*, the surrogate donor module for delivery of 10-undeycnoic acid, was produced from plasmid pSU18_tycA*, in which the sequence for the A domain of TycA was replaced with that of MBP (see Supplementary Note—Materials and methods for cloning details), in *E. coli* BL21 (DE3) cells and purified similar to wild-type TycA^23^. For post-translational modification, 10-undecynoyl-pant, PanK, PPAT, DPCK and Sfp were pre-incubated in assay buffer for 15 min at 37 °C, directly followed by addition of TycA* and incubation for an additional 5 min at 37 °C (final concentrations: 500 µM 10-undecynoyl-pant, 8 µM PanK, 8 µM PPAT, 8 µM DPCK, 2 µM Sfp and 195 µM TycA*). The reaction mixture containing the product 10-undeycnoyl-ppant-TycA* complex was used immediately in subsequent assays without further purification.

### Library construction and display

The C-domain library was constructed by homologous recombination in yeast. The vector backbone was generated by digesting pCT_srfA-C* with NheI and SalI, purified by 1% agarose gel electrophoresis and extracted using the Zymoclean Gel DNA Recovery Kit (Zymo Research). The library insert, which was designed to overlap with the backbone by 100 bp on each end, contained six degenerate codons (Supplementary Table 1). It was produced by amplifying three pCT_srfA-C* fragments using the primer pairs P1/R29, R30:R31(8:1)/R32:R33(8:1) and R34/P2, followed by assembly PCR in a second step using P1/P2 (all primer sequences are listed in Supplementary Table 3). Purified vector backbone and insert were used to transform electrocompetent EBY100 cells^50^, which were prepared as described in ref. ^69^ using 3 µg each of the insert and backbone for 800 µl competent cells. The cells were rescued in 16 ml (1 M) sorbitol:YPD (1:1) for 1 h at 30 °C and 225 r.p.m. Transformed cells were collected by centrifugation at 3,000*g* for 3 min at 4 °C, resuspended in minimal medium SD–CAA and plated out on SD–CAA in serial dilutions to determine the transformation efficiency. The library was regrown at 30 °C and 230 r.p.m. until an OD_600_ of 2–4 was reached and subsequently diluted to an OD_600_ of 0.2 (in 55 ml SD–CAA). The next day, the library was regrown at 30 °C and 230 r.p.m. until an OD_600_ of 2–4 was reached and subsequently diluted to an OD_600_ of 0.2. This step was repeated twice and cells were diluted to an OD_600_ of 0.2 and stored at 4 °C until further use. The integrity of the library was confirmed by Sanger sequencing.

### High-throughput screening of the SrfA-C library

The SrfA-C C-domain library was displayed on yeast by regrowing 55 ml of the transformed cell culture (OD_600_ = 0.2) for 6 h at 30 °C and 260 r.p.m. The cells were collected by centrifugation at 5,000*g* at 4 °C for 3 min, resuspended in 55 ml SG–CAA medium, shaken for 21–23 h at 20 °C and 230 r.p.m. and modified with ppant and l-Leu as before. For reactions with 10-undecynoic acid as the donor substrate, 3 ml cells were washed with 2.7 ml PMB, resuspended in a 750 μl reaction mixture containing 195 μM freshly prepared 10-undecynoyl-ppant-TycA* and incubated for 2–15 min at room temperature. After biotinylation and fluorescent labeling, the library was sorted using the FACSAria III cell sorter (BD Biosciences; at ~7,000–25,000 events per second) at the Flow Cytometry Core Facility of ETH Zurich. In the first sort, 7.4 × 10^7^ cells of the C-domain library were analyzed (approximately fivefold oversampling). After sorting, enriched cells were regrown at 30 °C and 250 r.p.m. in SD–CAA supplemented medium containing 100 μg ml^−1^ chloramphenicol to prevent bacterial contamination until an OD_600_ of 2–6 was reached. The cells were diluted to an OD_600_ = 0.2 and regrown until an OD_600_ of 2–4 was reached. The cells were again diluted to OD_600_ = 0.2 and stored until the next sort. Sorting and enrichment were repeated a total of five times, increasing stringency by reducing the incubation time from 15 to 2 min (Supplementary Fig. 5). After the final sort, genes of representative variants were isolated and analyzed by Sanger sequencing (Microsynth AG).

### Characterization of isolated SrfA-C variants on yeast

Variants 2, 4, 7, 10 (SrfA-C_FA_), 11, 13, 14 and 15 were individually displayed on yeast as described above. The samples were incubated with 50 µM 10-undecynoyl-ppant-TycA* for 100 μl cell samples at room temperature for 5 min and, following fluorescent labeling, analyzed by flow cytometry as described above. The sort 5 pool was treated identically for comparison.

### Kinetic characterization of selected SrfA-C variants

The genes for the three best C-domain variants from the individual yeast display assays were amplified from the pCT vector by PCR and assembled into the full-length C–A–T–TE SrfA-C module in the pTrc99a plasmid^70^. The C-terminally hexahistidine-tagged holo SrfA-C variants were produced in *E. coli* HM0079, purified at pH 7.4 and stored at pH 7.25 (see Supplementary Note—Materials and methods for details)^23^. The purified variants (1–2 μM) and 2 mM Leu were added to a solution of 10-undecynoyl-ppant-TycA*, freshly prepared as described above (final concentrations: 800 µM 10-undecynoyl-pant, 8 µM PanK, 8 µM PPAT, 8 µM DPCK, 2 µM Sfp and 426 µM TycA*) and incubated at 37 °C. The formation of 10-undecynoyl-l-Leu was monitored as a function of time by periodically analyzing aliquots by LC–MS. A synthetic 10-undecynoyl-l-Leu standard was used for product quantification via the isolated ion count for the product ((M + H)^+^) in LC–MS chromatograms. The experiments were performed in triplicate with four biological replicates. For details, see Supplementary Note—Materials and methods.

For the biosynthesis of *O*-propargyl-d-Tyr-l-Leu, the SrfA-C variants (1.5 μM) were incubated with 5 mM *O*-propargyl-l-Tyr, 5 mM Leu and 15 µM W227S TycA instead of 10-undecynoyl-pant-TycA* and 2 mM Leu, under otherwise identical conditions. The reactions were performed as time course experiments in duplicate or triplicate with three biological replicates and analyzed by HPLC. A synthetic *O*-propargyl-d-Tyr-l-Leu standard was used for product quantification at 220 nm. A background control reaction was prepared containing all reagents and enzymes except SrfA-C, and rates were corrected for the nonenzymatic formation of *O*-propargyl-l-Tyr-l-Leu.

### Replacement of the C domain of TycB1 with that of SrfA-C_FA_

The gene encoding the C domain of TycB1, a truncated Pro-specific elongation module from tyrocidine A synthetase with an appended TE domain^24^, and full-length TycB were replaced with the gene encoding for the C domain of SrfA-C_FA_ to yield pTrc99a_tycB1_ FA and pTrc99a_tycB_ FA. TycB1_FA_ and TycB_FA_ were produced in *E. coli* HM0079 cells and purified as reported for the corresponding wild-type proteins^23^. For detailed protocols, see Supplementary Note—Materials and methods.

### Biosynthesis of 10-undecynoyl-l-Pro (3)

Enzymatic fatty acylation of l-Pro was performed in vitro in HEPES buffer (100 mM HEPES, 100 mM NaCl, 50 mM ATP, 10 mM MgCl_2_, 0.1 units ml^−1^ inorganic pyrophosphatase from baker’s yeast, pH 7.25). TycB1_FA_ (1.7 μM) was mixed with 2 mM l-Pro, added to a solution of 10-undecynoyl-ppant-TycA*, which was freshly prepared as described above (final concentrations: 800 µM 10-undecynoyl-pant, 8 µM PanK, 8 µM PPAT, 8 µM DPCK, 2 µM Sfp and 426 µM TycA*), and incubated at 37 °C. The amount of product formed was estimated using an authentic 10-undecynoyl-l-Leu standard. Reactions were performed in duplicate, and product formation was analyzed by LC–MS after 4 h. For details, see Supplementary Note—Materials and methods.

### Biosynthesis of lipopeptide (4)

Biosynthesis of 10-undecynoyl-l-Pro-l-Phe-d-Phe-l-Asn-l-Gln-l-Tyr-l-Val-l-Orn-l-Leu was performed in vitro in HEPES buffer (100 mM HEPES, 100 mM NaCl, 50 mM ATP, 10 mM MgCl_2_, 0.1 units ml^−1^ inorganic pyrophosphatase from baker’s yeast, pH 7.25). TycB_FA_ (1 μM) and TycC (1 μM) were combined with all required amino acids (2 mM l-Phe, 1 mM l-Pro, 1 mM l-Asn, 1 mM l-Gln, 1 mM l-Tyr, 1 mM l-Val, 1 mM l-Orn and 1 mM l-Leu), added to a solution of 10-undecynoyl-ppant-TycA*, which was freshly prepared as described above (final concentrations: 553 µM 10-undecynoyl-pant, 8 µM PPAT, 8 µM DPCK, 8 µM PanK, 2 µM Sfp and 355 µM TycA*), and incubated at 37 °C. Reactions were performed in triplicate, and product formation was analyzed by LC–HRMS/MS after 2 h. For detailed protocols, see Supplementary Note—Materials and methods.

### Production and purification of the SrfA-C_FA_ C domain

The gene for the C domain of the SrfA-C_FA_ module was synthesized by Bio Basic and subcloned into a pET21 vector. The vector contains an N-terminal tobacco etch virus (TEV) cleavable octa-histidine tag. For details, see Supplementary Note—Materials and methods. The protein was expressed in *E. coli* BL21 (DE3) cells grown at 37 °C in lysogeny broth medium supplemented with 50 μg ml^−1^ kanamycin. Protein expression was induced when the culture reached an OD_600_ of 0.6–0.8, by adding 0.5 mM isopropyl ß-D-1-thiogalactopyranoside and shaking at 16 °C for 19 h. Cells were collected by centrifugation before protein purification. For purification of the C domain of SrfA-C_FA_ for later crystallography experiments, cell pellets were resuspended in buffer A (2 mM imidazole, 150 mM NaCl, 2 mM β-mercaptoethanol (β-ME), 25 mM HEPES, pH 7.5) and lysed by sonication. The lysate was clarified by centrifugation at 20,000*g* for 30 min at 4 °C. The supernatant was loaded onto a 5 ml HiTrap IMAC FF column (Cytiva) charged with Ni^2+^ equilibrated in buffer A. The column was washed with buffer A, and the protein was eluted with a 10–50% gradient of buffer B (250 mM imidazole, 150 mM NaCl, 2 mM β-ME, 25 mM HEPES, pH 7.5). Fractions containing purified protein were pooled and incubated with TEV protease during dialysis into buffer A overnight at room temperature. Protein was reloaded onto the HiTrap IMAC FF column to remove uncleaved protein, and the flow-through was collected. Protein was then applied to a MonoQ HR 16/60 column (Cytiva) equilibrated in buffer Q1 (0.5 mM TCEP, 25 mM HEPES, pH 7.5). Bound protein was washed with buffer Q1 plus 100 mM NaCl and eluted using a gradient of 100–600 mM NaCl over 80 ml. Protein was pooled, concentrated, and applied to a HiLoad 16/60 Superdex S75 column (GE Healthcare) equilibrated in size exclusion buffer (150 mM NaCl, 0.5 mM TCEP, 25 mM HEPES, pH 7.5). Purity was accessed by SDS–PAGE, and pure fractions were pooled, concentrated to 38.9 mg ml^−1^, flash-frozen in liquid nitrogen and stored at −80 °C.

### Protein crystallization and structure determination

The isolated C domain of SrfA-C_FA_ was used in sparse matrix crystallization trials to obtain initial crystallization conditions, with 3,000 crystallization conditions in 96-well plates at 4 °C and 22 °C assayed. The most promising crystals were optimized by fine screening in 24-well, sitting drop plates using 2 μl protein solution and 2 μl reservoir solution in the drop and a 500 μl reservoir volume. Two conditions produced diffraction-quality crystals. The first condition included 25 mg ml^−1^ of protein and a crystallization solution of 0.04 M KH_2_PO_4_, 16% (wt/vol) polyethylene glycol (PEG) 8 K and 20% (vol/vol) glycerol. The second condition included 25 mg ml^−1^ of protein and a crystallization solution of 0.16 M calcium acetate, 0.08 M sodium cacodylate (pH 6.5), 14.4% (wt/vol) PEG 8 K and 20% (vol/vol) glycerol. Wild-type SrfA-C C domain was also crystallized in these two conditions. Crystals were looped and flash-cooled in liquid nitrogen. Diffraction data sets were collected using beamline CMCF-ID of the Canadian Light Source, NE-CAT 24-ID-E beamline at the Advanced Photon Source (APS) and beamline 23ID-B at APS during the CCP4/APS school in macromolecular crystallography. Data were indexed and scaled with HKL2000 (ref. ^71^), HKL3000 (ref. ^72^) or DIALS^73^. Structure determination was accomplished by molecular replacement in Phaser^74^ using the C domain of the SrfA-C termination module structure (PDB: 2VSQ)^55^ as a search model, followed by iteratively building in Coot^75^ and refinement in Phenix^76^. The data collection and refinement statistics are summarized in Supplementary Table 2.

### Donor complex modeling

As co-complex structures were not forthcoming, we produced a model of 10-undecynoyl-ppant-T_TycA_ bound to the SrfA-C_FA_ C-domain variant. The T_TycA_ model was produced using RoseTTAFold^77^ and ligated to a model of 10-undecynoyl-ppant. The 10-undecynoyl-ppant-T_TycA_ was placed based on aminoacyl-ppant-T^49^ and fatty acyl-CoA structures^39^, and energy was minimized using Phenix, with 10-undecynoyl-ppant restraints generated in eLBOW^78^.

### Reporting summary

Further information on research design is available in the Nature Portfolio Reporting Summary linked to this article.

## Online content

Any methods, additional references, Nature Portfolio reporting summaries, source data, extended data, supplementary information, acknowledgements, peer review information; details of author contributions and competing interests; and statements of data and code availability are available at 10.1038/s41589-023-01532-x.

### Supplementary information

Supplementary Information Supplementary Note, Tables 1–3, Figs. 1–13, and supporting data for Supplementary Figs. 1 and 11.

Reporting Summary

Supplementary Data 1 Supporting data for Supplementary Figs. 1, 2 and 4–10.

Supplementary Data 2 Raw data for flow cytometry and FACS dot plots of Figs. 2 and 3.

Supplementary Data 3 Raw file for energy-minimized model of the engineered C domain with substrate–donor complex.

### Source data

Source Data Fig. 2 Exported statistics for flow cytometry analysis.

Source Data Fig. 3 Exported statistics for flow cytometry/FACS analysis.

Source Data Fig. 4 Statistical source data for enzyme apparent rate constants.

Source Data Fig. 5 Statistical source data for LC–MS and LC–HRMS/MS data.

## Data Availability

The X-ray crystal structures and diffraction data from this study were deposited in the Research Collaboratory for Structural Bioinformatics PDB under accession codes 8F7F, 8F7G, 8F7H and 8F7I. Source data for all figures in the main text and Supplementary Information have been supplied with the paper or as Supplementary Information files. Source data are provided with this paper.
