# Supplementary material for: High-throughput reprogramming of an NRPS condensation domain
Source: Nat Chem Biol. 2024 Feb 2;20(6):761–9. doi: 10.1038/s41589-023-01532-x (PMC11142918; doi:10.1038/s41589-023-01532-x)

nature chemical biology

Article

https://doi.org/10.1038/s41589-023-01532-x

High-throughput reprogramming of an NRPS condensation domain

In the format provided by the authors and unedited

Table of Contents

Materials & Methods 2

General 2

Chemical Synthesis 2

Plasmids 12

Cloning 12

Protein production 16

In Vitro NRPS Assays 19

Supplementary Tables 23

Supplementary Figures 27

Protein Sequences 41

DNA sequence of the yeast display plasmid *pCT_SrfA-C** 46

High-resolution mass spectrometry (ESI-MS) 49

NMR spectroscopy 54

References 76

Source Data 78

Source Data Suppl. Fig. 1 78

Source Data Suppl. Fig. 11 79

Materials & Methods

General

All reagents and chemicals were used as received. All solvents were analytical HPLC grade. Reagents were purchased from Tokyo Chemical Industry, Chem Impex, Acros, Apollo, ABCR, Fluorochem, Aapptec, Fisher Bioreagents, Merck or Sigma Aldrich and used as received. ATP was purchased from Meiya Pharmaceuticals and used as received. Preparative HPLC was conducted on a Waters system equipped with 2525 quaternary gradient pumps in line with a 2489 dual λ absorbance detector, a fraction collector, and a Gemini 5 µm NX C18 110 column (250 x 21.2 mm, Phenomenex) if not stated otherwise. High-resolution mass spectrometry (HRMS) and tandem mass spectrometry (MS/MS) were performed at the LOC Mass Spectrometry Service at ETH Zürich (MoBiAS) on a Bruker maxis-ESI-Qq-TOF-MS by electrospray ionization (ESI). For MS/MS analysis, the relevant $[M+H]^+$ species were selected for collision-induced dissociation (CID; collision cell Rf 1500 Vpp) with nitrogen as the collision gas. NMR spectra were recorded on a Bruker Avance-III 600 MHz, a Bruker Neo 500 MHz, a Bruker Avance III 400 MHz or a Varian Mercury-VX 300 MHz spectrometer at the NMR service of the Laboratory of Organic Chemistry (LOC) at ETH Zürich. ^{1}H-NMR spectra were recorded relative to the residual solvent peak ($CDCl_3$ δH 7.26 ppm, DMSO-d_6 δH 2.50 ppm or CD_3OD δH 3.31 ppm) and reported as follows: chemical shift (ppm), multiplicity, coupling constant (Hz), and integration. Multiplicity abbreviations are as follows: s = singlet, d = doublet, t = triplet, q = quartet, dd, doublet of doublets, td = triplet of doublets, m = multiplet. ^{13}C NMR spectra were recorded relative to the residual solvent peak ($CDCl_3$ δC 77.0 ppm, d_6-DMSO δC 39.5 ppm or MeOD δH 49.0 ppm). The HM0079 cell line[7] was obtained from M.A. Marahiel (Philipps Universität Marburg), EBY100 from A. Plückthun (University of Zurich), and BL21 (DE3) and XL1Blue from Agilent.

Chemical Synthesis

All reactions were performed under ambient atmosphere in vessels equipped with a PTFE-coated magnetic stir bar and stirred at room temperature unless stated otherwise. Analytical thin-layer chromatography (TLC) was performed with Merck 60 F_{254} pre-coated glass plates (0.25 mm), visualized by UV detection (254 nm), and analysed by extraction with MeOH followed by LC-MS (Waters H-class UPLC/SQD-2) analysis. Preparative TLC was conducted similarly, and separated product spots were analysed by LC-MS (Waters H-class UPLC/SQD-

2) prior to extraction with MeOH (3x 20 mL). Silica 230-400, 60 Å was used for flash chromatography.

O-Propargyl-L-Tyr[1] and *O*-propargyl-(S)-β-Tyr[2] were synthesized as described previously. (R)-Pantetheine, PMB-amino-pant, Boc-*O*-propargyl-D-Tyr and *O*-propargyl-D-Tyr-L-Leu were prepared using literature procedures[3–5].

(*R*)-Pantetheine *(Pant)*

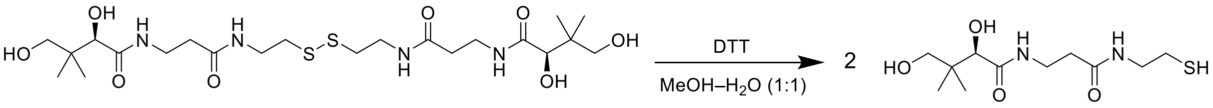

The reduction procedure was adapted from a previous report[3]. D-Pantethine (10.4 mmol, 5.80 g, 1.00 equiv.) was dissolved in degassed MeOH–H_2O (1:1, 20 mL) before addition of 1,4-dithiothreitol (15.6 mmol, 2.40 g, 1.50 equiv.) and stirred for 2 h at room temperature. (*R*)-Pantetheine was purified by flash chromatography using a gradient of 3% to 10% MeOH in DCM to give 4.90 g (84% yield) product as a colourless oil. Analytical data were in accord with published spectra[3].

(*R*)-Pantetheine-undec-10-ynethioate *(10-Undecynoyl-pant)*

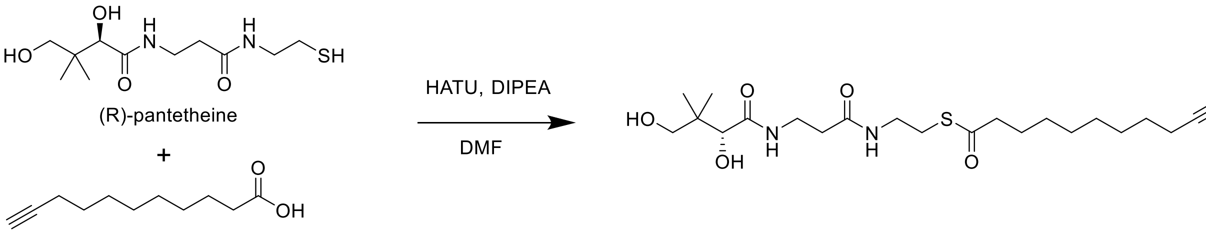

(*R*)-Pantetheine (83.5 µmol, 23.2 mg, 1.00 equiv.) and 10-undecynoic acid (0.10 mmol, 18.2 mg, 1.20 equiv.) were dissolved in anhydrous DMF (1 mL). Subsequently, HATU (95.8 µmol, 36.4 mg, 1.15 equiv.) and *N,N*-diisopropylethylamine (0.10 mmol, 17.4 µL, 1.20 equiv.) were slowly added and the mixture was stirred for 2 h. After concentration, the crude product was purified by preparative TLC using MeOH-$CHCl_3$ (1:9) as eluent. The solvent was removed under reduced pressure, and the product was obtained as a slightly yellow solid.

The final product was contaminated with tetramethylurea (^{1}H NMR: δ 2.54; s, 4H) in a molar ratio of 3:1 product:impurity. Approximately 18.6 mg product (50% yield) were obtained, not considering the contaminant. Since tetramethylurea should not interfere with subsequent

biochemical assays, the product was not further purified. The solvent was evaporated under high vacuum and a final product stock solution was prepared in DMSO and stored at -20°C for further use.

^{1}H NMR (500 MHz, DMSO-d6) δ = 8.10 (t, J = 5.7 Hz, 1H), 7.68 (t, J = 6.0 Hz, 1H), 5.38 (d, J = 5.5 Hz, 1H), 4.48 (t, J = 5.6 Hz, 1H), 3.69 (d, J = 5.4 Hz, 1H), 3.32 – 3.12 (m, 6H), 2.88 (t, J = 6.9 Hz, 2H), 2.73 (t, J = 2.7 Hz, 1H), 2.56 (t, J = 7.4 Hz, 2H), 2.24 (td, J = 7.1, 2.5 Hz, 2H), 2.13 (td, J = 7.0, 2.7 Hz, 2H), 1.54 (q, J = 7.1 Hz, 2H), 1.45 – 1.37 (m, 2H), 1.36 – 1.28 (m, 2H), 1.28 – 1.22 (m, 6H), 0.78 (d, J = 11.9 Hz, 6H) ppm.

^{13}C NMR (126 MHz, DMSO-d6) δ = 198.5, 172.8, 170.6, 84.5, 75.0, 71.1, 68.0, 43.4, 39.2, 38.1, 35.1, 34.7, 28.5, 28.2, 28.1, 28.0, 27.9, 27.9, 25.0, 20.9, 20.3, 17.6 ppm.

ESI HRMS: calculated $[M+Na]^+$ 465.2394, found 465.2392

3-((4*R*)-2-(4-Methoxyphenyl)-5,5-dimethyl-1,3-dioxane-4-carboxamido)propanoic acid *(S1)*

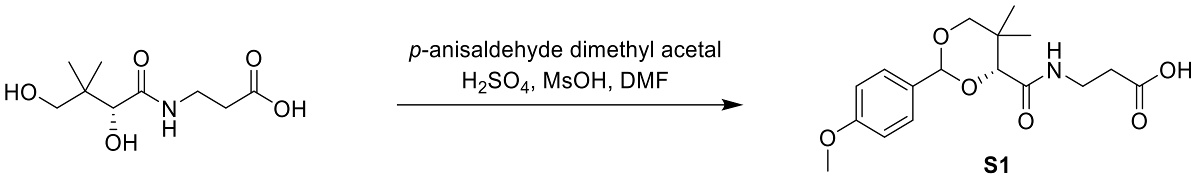

The synthetic procedure was adapted from a previous report[4]. Sodium pantothenate (20.7 mmol, 5.00 g, 1.00 equiv.) was dissolved in anhydrous DMF (40 mL) and 95% H_2SO_4 (20.7 mmol, 1.19 mL, 1.00 equiv.) was added dropwise while stirring. Subsequently, *p*-anisaldehyde dimethyl acetal (22.8 mmol, 4.15 g, 1.10 equiv.) and methanesulfonic acid (2.07 mmol, 0.13 mL, 0.10 equiv.) were added and the reaction was stirred for 24 h at room temperature. The reaction was diluted with H_2O and extracted with EtOAc (3x) before drying over Na_2SO_4 and concentration under reduced pressure. The crude product (**S1**) was triturated with Et_2O, stored at 4 °C overnight, and the organic layer was subsequently discarded. The product mass was confirmed by LC-MS and the product (2.20 g, 32% yield) was carried on to the next step without further purification.

2-Azidoethylamine

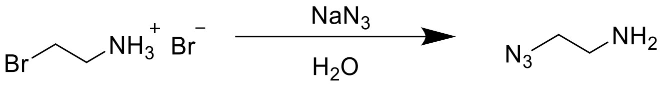

The synthetic procedure was adapted from a previous report[4]. 2-Bromoethylamine hydrobromide (48.0 mmol, 9.84 g, 1.00 equiv.) was solubilized in an aqueous solution of sodium azide (146 mmol, 9.52 g, 3.05 equiv.; 150 mL) and heated to 75 °C for 24 h. The mixture was subsequently cooled to 0 °C before addition of KOH (53.5 mmol, 3.00 g, 1.11 equiv.). The mixture was partitioned between Et_2O (3x 300 mL) and H_2O, and the organic layers were combined and dried over Na_2SO_4 before the solvent was evaporated under a stream of nitrogen. The product (1.90 g, 46% yield) was obtained as a beige solid.

(4*R*)-*N*-(3-((2-Azidoethyl)amino)-3-oxopropyl)-2-(4-methoxyphenyl)-5,5-dimethyl-1,3-dioxane-4-carboxamide *(S2)*

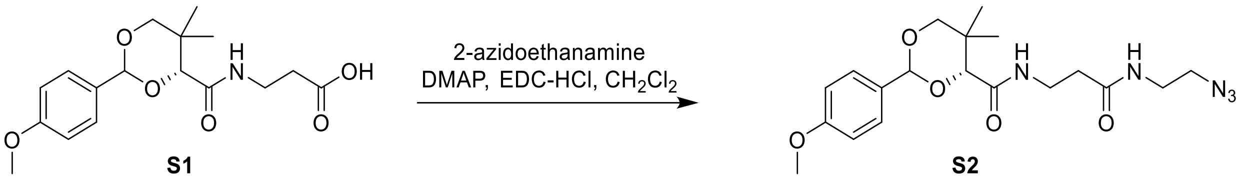

The synthetic procedure was adapted from a previous report[4]. Compound **S1** (6.53 mmol, 2.20 g, 1.00 equiv.), 2-azidoethylamine (9.80 mmol, 0.84 g, 1.50 equiv.), 1-(3-dimethyl-aminopropyl)-3-ethylcarbodiimide hydrochloride (9.80 mmol, 1.87 g, 1.50 equiv.) and catalytic amounts of 4-dimethylaminopyridine were dissolved in DCM (30 mL) and stirred for 12 h at room temperature. The solvent was evaporated under reduced pressure and the mixture partitioned between EtOAc and H_2O several times before the combined organic phase was washed with saturated $NaHCO_3$. The product was isolated by flash chromatography using in EtOAc as eluent. The product mass was confirmed by LC-MS analysis and the product (2.28 g, 86% yield) was carried on to the next step.

(4*R*)-*N*-(3-((2-Aminoethyl)amino)-3-oxopropyl)-2-(4-methoxyphenyl)-5,5-dimethyl-1,3-dioxane-4-carboxamide *(S3, PMB-amino-pant)*

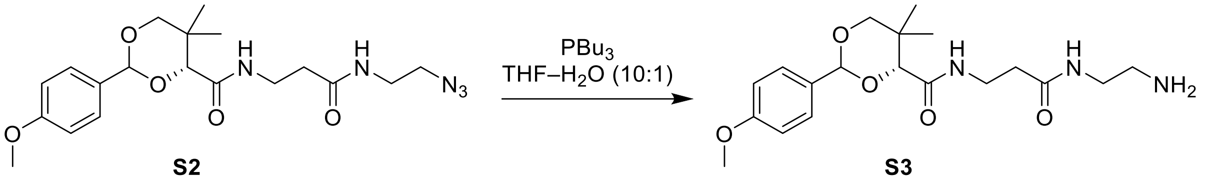

The synthetic procedure was adapted from a previous report[4]. Compound **S2** (4.88 mmol, 2.00 g, 1.00 equiv.) was dissolved in THF–H_2O (10:1, 25 mL). Tributylphosphine (9.78 mmol, 1.98 g, 2.00 equiv.) was added and the resulting mixture was stirred overnight. THF was removed under a stream of nitrogen, the aqueous phase was washed three times with Et_2O (3x), and H_2O was removed under vacuum to give **S3** (1.76 g, 95% yield) as a colourless oil.

Analytical data were in accord with published spectra:

^{1}H NMR (400 MHz, CD_3OD) δ = 7.54 – 7.43 (m, 2H), 7.02 – 6.90 (m, 2H), 5.55 (s, 1H), 4.17 (s, 1H), 3.82 (s, 3H), 3.78 – 3.66 (m, 2H), 3.50 (m, 2H), 3.24 (t, J = 6.3 Hz, 2H), 2.71 (t, J = 6.3 Hz, 2H), 2.44 (t, J = 6.6 Hz, 2H), 1.09 (d, J = 26.1 Hz, 6H) ppm.

(4*R*)-2-(4-Methoxyphenyl)-5,5-dimethyl-*N*-(3-oxo-3-((2-(undec-10-ynamido)ethyl) amino)propyl)-1,3-dioxane-4-carboxamide *(10-Undecynoyl-pant-amide)*

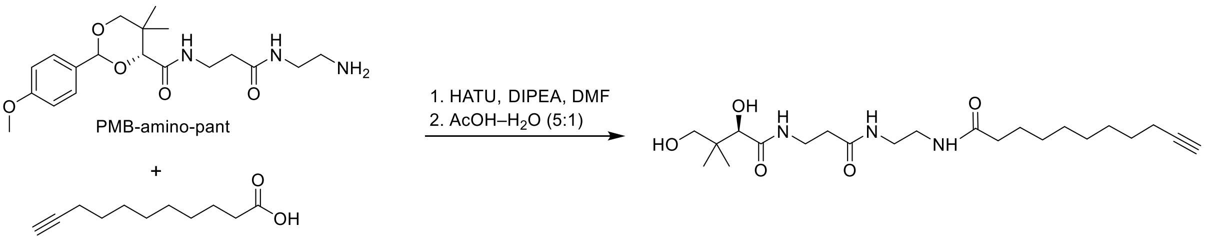

PMB-amino-pant (100 µmol, 37.9 mg, 1.00 equiv.) and 10-undecynoic acid (110 µmol, 20.0 mg, 1.10 equiv.) were solubilized in anhydrous DMF (1.5 mL). *N,N*-Diisopropyl-ethylamine (120 µmol, 20.4 µL, 1.20 equiv.) and HATU (110 µmol, 41.8 mg, 1.10 equiv.) were added and the reaction was stirred overnight. Solvents were evaporated under high vacuum and the intermediate was isolated by preparative TLC using EtOAc as eluent. The reaction intermediate was subsequently dissolved in AcOH-H_2O (5:1) and stirred for 4 h. Solvents were removed under reduced pressure and the crude product was subjected to preparative TLC with $CHCl_3$–MeOH (9:1) for purification. Pure product (16.7 mg, 38% yield) was obtained as a colourless solid.

^{1}H NMR (500 MHz, DMSO-d$_6$) δ = 7.95 (t, J = 5.5 Hz, 1H), 7.82 (t, J = 17.5, 5.7 Hz, 1H), 7.70 (t, J = 5.9 Hz, 1H), 5.50 (s, 1H), 4.56 (s, 1H), 3.70 (s, 1H), 3.32 – 3.18 (m, 3H), 3.18 – 3.12 (m, 1H), 3.09 – 3.03 (m, 4H), 2.72 (t, J = 2.7 Hz, 1H), 2.24 (td, J = 7.0, 2.2 Hz, 2H), 2.13 (td, J = 7.0, 2.7 Hz, 2H), 2.03 (t, J = 7.5 Hz, 2H), 1.51 – 1.38 (m, 4H), 1.36 – 1.16 (m, 8H), 0.78 (d, J = 17.1 Hz, 6H) ppm.

^{13}C NMR (126 MHz, DMSO) δ = 172.9, 172.3, 170.6, 84.5, 74.9, 71.1, 68.0, 39.0, 38.4, 38.2, 35.4, 35.2, 34.8, 28.7, 28.6, 28.4, 28.1, 27.9, 25.2, 21.0, 20.3, 17.7 ppm.

ESI HRMS: calculated $[M+Na]^+$ 448.2782, found 448.2779

Undec-10-ynoyl-L-Leucine *(10-Undecynoyl-L-Leu)*

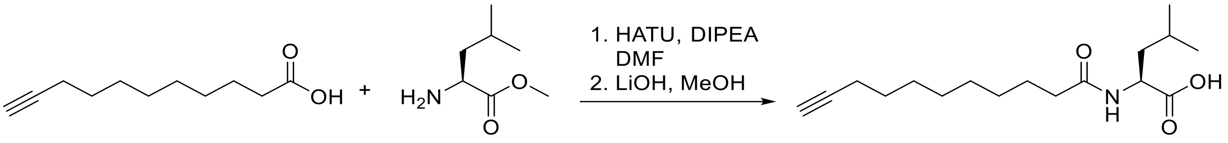

O-Methyl-L-leucine (120 µmol, 21.8 mg, 1.20 equiv.) and 10-undecynoic acid (100 µmol, 18.2 mg, 1.00 equiv.) were dissolved in anhydrous DMF (1.0 mL). Subsequently, *N,N*-diisopropylethylamine (120 µmol, 20.9 µL, 1.20 equiv.) and HATU (115 µmol, 43.7 mg, 1.15 equiv.) were added slowly. The mixture was stirred for 2 h and the resulting ester intermediate was purified by preparative TLC using MeOH-DCM (1:5) as eluent. The solvent was removed under reduced pressure and the intermediate was solubilized in H_2O–MeOH (3:1, 1 mL). After addition of LiOH·H_2O (130 µmol, 5.46 mg, 1.30 equiv.) the mixture was stirred for 3 h at room temperature. The pH was adjusted to 7 and the reaction mixture purified by preparative HPLC (10 mL injection, monitoring with a diode array detector at 220 nm and 254 nm, solvent A = H_2O + 0.1% TFA, solvent B = MeCN + 0.1% TFA, flow rate = 25 mL/min, 0 min = 5% B, 0 – 23 min ramp to 99% B, 23-25 min = 99% B, 25-27 min ramp to 5% B, 27-30 min re-equilibration = 5% B.). Individual fractions were analysed by LC-MS (Waters H-class UPLC/SQD-2). Pure fractions were combined, and the solvent was removed under high vacuum to yield the product as a yellow solid (2.97 mg, 10% yield).

^{1}H NMR (500 MHz, MeOD) δ = 4.42 (dd, J = 8.3, 6.7 Hz, 1H), 2.24 (t, J = 7.4 Hz, 2H), 2.18 – 2.12 (m, 3H), 1.74 – 1.66 (m, 1H), 1.66 – 1.58 (m, 4H), 1.54 – 1.46 (m, 2H), 1.45 – 1.37 (m, 2H), 1.37 – 1.28 (m, 6H), 0.95 (dd, J = 18.1, 6.5 Hz, 6H) ppm.

^{13}C NMR (126 MHz, MeOD) δ = 176.5, 176.3, 85.1, 69.3, 52.1, 41.7, 36.8, 30.3, 30.2, 30.1, 29.7, 29.7, 26.9, 26.1, 23.4, 21.8, 19.0 ppm.

ESI HRMS: calculated $[M+Na]^+$ 318.2040, found 318.2032

(*R*)-2-((*tert*-Butoxycarbonyl)amino)-3-(4-(prop-2-yn-1-yloxy)phenyl)propanoic acid *(Boc-O-propargyl-D-Tyr)*

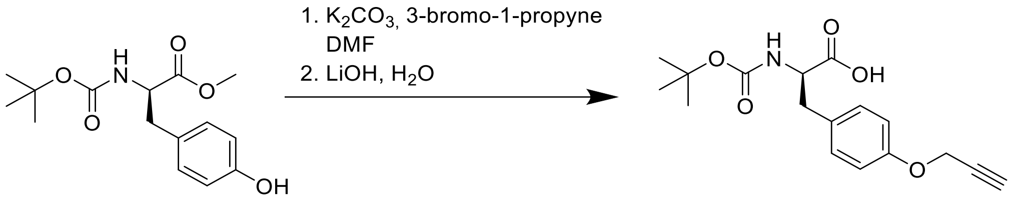

O-Methyl-Boc-D-tyrosine (8.46 mmol, 2.50 g, 1.00 equiv.) and K_2CO_3 (12.7 mmol, 1.75 mmol, 1.50 equiv.) were solubilized in anhydrous DMF (20 mL). Propargyl bromide (11.8 mmol, 1.32 mL, 1.40 equiv.) was added dropwise and the solution was stirred at room temperature overnight. Water was added to the reaction mixture and the intermediate was extracted using EtOAc (3x 50 mL). Organic layers were combined and washed with a saturated aqueous thiosulfate solution, and the solvent was evaporated under reduced pressure. The reaction intermediate was solubilized in THF–H_2O (3:1, 20 mL) and LiOH·H_2O (42.3 mmol, 1.78 g, 5.00 equiv.) was added. The reaction was stirred for 3 h and diluted with EtOAc and H_2O. The organic layer was discarded, and the aqueous phase was acidified to pH 3 and extracted with DCM (3x 50 mL). The product (2.45 g, 91% yield) was obtained as a yellow solid. NMR data were in accord with the reported spectrum of the L enantiomer[5].

ESI HRMS: calculated $[M+Na]^+$ 455.2153, found 455.2154

(*R*)-2-((*tert*-Butoxycarbonyl)amino)-3-(4-(prop-2-yn-1-yloxy)phenyl)propanoyl)-L-leucine *(Boc-O-propargyl-D-Tyr-L-Leu)*

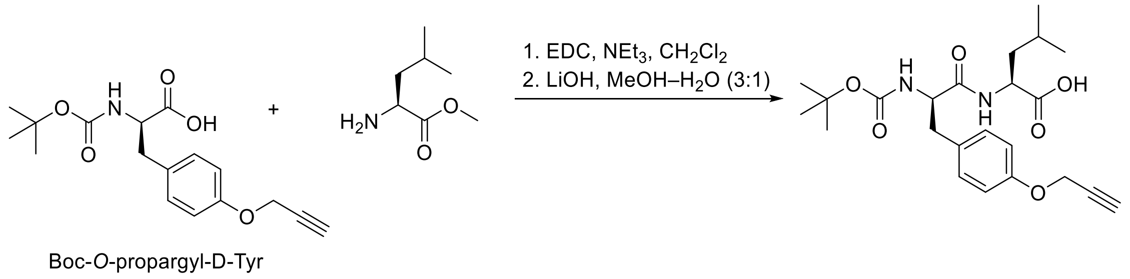

Boc-*O*-propargyl-D-tyrosine (0.35 mmol, 113 mg, 1.00 equiv.), *O*-Methyl-L-leucine (0.39 mmol, 70.5 mg, 1.10 equiv.), 1-ethyl-3-(3-dimethylaminopropyl)carbodiimide hydrochloride (0.77 mmol, 148 mg, 2.20 equiv.) and trimethylamine (0.77 mmol, 105 µL,

2.20 equiv.) were solubilized in DCM (2 mL) and stirred for 3 h. After evaporation of the solvent, the crude reaction was purified by preparative HPLC (10 mL injection, monitoring with a diode array detector at 220 nm and 254 nm, solvent A = H_2O + 0.1% TFA, solvent B = MeCN + 0.1% TFA, flow rate = 25 mL/min, 0 min = 5% B, 0 – 23 min ramp to 99% B, 23-25 min = 99% B, 25-27 min ramp to 5% B, 27-30 min re-equilibration = 5% B.). Individual fractions were analysed by LC-MS (Waters H-class UPLC/SQD-2). Pure fractions were combined, and the solvent was removed under high vacuum. The intermediate was solubilized in MeOH–H_2O (3:1, 4 mL), and LiOH·H_2O (0. 36 mmol, 15.0 mg, 1.02 equiv.) was added and stirred for 2 h. MeOH was removed under reduced pressure and the aqueous phase was adjusted to pH 3 before extraction with EtOAc (3x 10 mL). Organic layers were combined, dried over $NaSO_4$ and solvent was removed under reduced pressure. The crude product was subjected to the next reactions without further purification.

^{1}H NMR (500 MHz, MeOD) δ = 7.22 – 7.11 (m, 2H), 6.90 (m, 2H), 4.68 (d, J = 2.4 Hz, 2H), 4.41 – 4.21 (m, 2H), 3.01 - 2.95 (m, 1H), 2.91 (t, J = 2.4 Hz, 1H), 2.816 – 2.76 (m, 1H), 1.49 – 1.60 (m, 2H), 1.35 – 1.47 (m, 10H), 0.80 – 0.94 (m, 6H) ppm.

^{13}C NMR (126 MHz, MeOD) δ = 175.8, 174.2, 158.1, 157.4, 131.4, 131.2, 115.9, 80.7, 79.9, 76.6, 57.6, 56.6, 52.0, 41.6, 38.8, 28.7, 25.7, 23.5, 21.8 ppm.

ESI HRMS: calculated [M+Na]$^+$ 455.2153, found 455.2154

(*S*)-2-((*R*)-2-Amino-3-(4-(prop-2-yn-1-yloxy)phenyl)propanamido)-*N*-(2-(3-((*R*)-2,4-dihydroxy-3,3-dimethylbutanamido) propanamido)ethyl)-4-methylpentanamide

(O-Propargyl-D-Tyr-L-Leu-pant-amide)

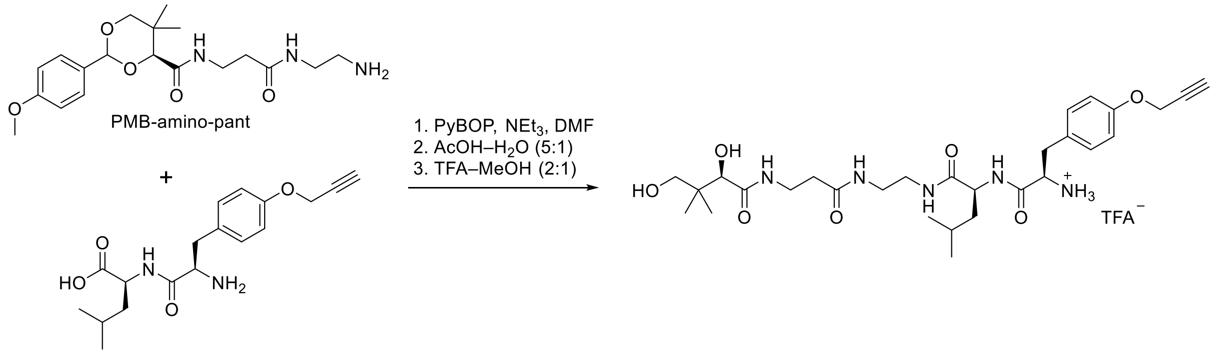

Boc-*O*-propargyl-D-Tyr-L-Leu (0.25 mmol, 0.11 g, 1.00 equiv.), PMB-amino-pant (0.31 mmol, 121 g, 1.23 equiv.), PyBOP (0.31 mmol, 160 g, 1.23 equiv.) and triethylamine (0.34 mmol, 46.0 µL, 1.34 equiv.) were dissolved in anhydrous DMF (3 mL) and reacted for 2 h. The mixture was diluted with H_2O and extracted with EtOAc (3x 20 mL). Organic layers

were combined, dried over $NaSO_4$ and the solvent was removed under reduced pressure. The crude intermediate was dissolved in AcOH–H_2O (5:1, 5 mL) and stirred for 1 h before solvents were removed under high vacuum. Subsequently, the crude intermediate was dissolved in TFA–MeOH (2:1, 2 mL) and stirred for 30 min. Solvents were evaporated under nitrogen flow and the crude product was purified by preparative HPLC (10 mL injection, monitoring with a diode array detector at 220 nm and 254 nm, solvent A = H_2O + 0.1% TFA, solvent B = MeCN + 0.1% TFA, flow rate = 25 mL/min, 0 min = 5% B, 0 – 23 min ramp to 99% B, 23-25 min = 99% B, 25-27 min ramp to 5% B, 27-30 min re-equilibration = 5% B.). Individual fractions were analysed by LC-MS (Waters H-class UPLC/SQD-2). Pure fractions were combined, and the solvent was removed under high vacuum to yield the pure product (2.53 mg, 2% yield) as a yellow solid. The sample was contaminated by small amounts of grease, acetic acid, ethyl acetate and acetonitrile, and the volatiles were removed by high vacuum before preparation of a stock solution in DMSO and storage at -20°C.

^{1}H NMR (500 MHz, MeOD) δ = 7.22 – 7.18 (m, 2H), 7.00 – 6.96 (m, 2H), 4.72 (d, J = 2.4 Hz, 2H), 4.20 – 4.08 (m, 2H), 3.90 (d, J = 10.5 Hz, 1H), 3.59 – 3.35 (m, 6H), 3.29 – 3.13 (m, 2H), 3.07 (d, J = 7.9 Hz, 2H), 2.95 (t, J = 2.4 Hz, 1H), 2.52 – 2.35 (m, 2H), 1.47 – 1.37 (m, 2H), 1.12 – 0.99 (m, 1H), 0.95 – 0.88 (m, 6H), 0.81 (dd, J = 25.4, 6.6 Hz, 6H) ppm.

^{13}C NMR (126 MHz, MeOD) δ = 176.2, 174.8, 174.6, 169.7, 158.8, 131.5, 128.2, 116.5, 79.7, 77.3, 76.9, 70.2, 56.7, 56.0, 53.4, 41.4, 41.0, 40.4, 39.8, 37.7, 36.9, 36.5, 25.4, 23.6, 21.7, 21.4, 20.9 ppm.

ESI HRMS: $[M+H]^+$: calculated, 576.3392; found, 576.3382

(*R*)-2-Amino-3-(4-(prop-2-yn-1-yloxy)phenyl) propanoyl)-L-leucine *(O-propargyl-D-Tyr-L-Leu)*

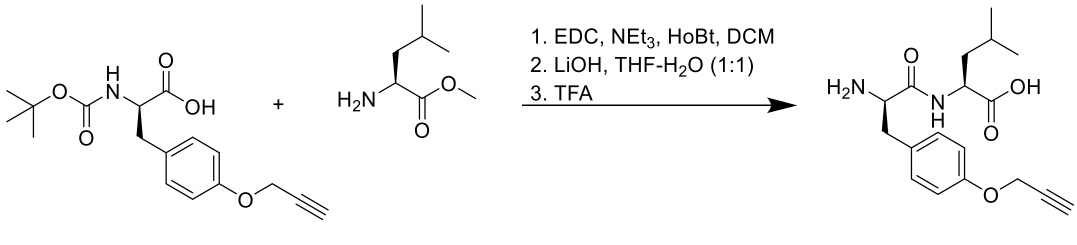

Boc-*O*-propargyl-D-tyrosine (0.54 mmol, 173 mg, 1.00 equiv.), *O*-methyl-L-leucine (0.59 mmol, 108 mg, 1.10 equiv.), 1-ethyl-3-(3-dimethylaminopropyl)carbodiimide hydrochloride (0.81 mmol, 155 mg, 1.50 equiv.), 1-hydroxybenzotriazol (0.65 mmol, 99.1 mg, 1.20 equiv.) and trimethylamine (1.08 mmol, 151 µL, 2.00 equiv.) were solubilized in DCM

(5 mL) and stirred for 1-3 h. The mixture was quenched by addition of saturated $NaHCO_3$ (5 mL), and the mixture was extracted with DCM (3x 30 mL). The organic phases were combined, and the methyl ester intermediate was isolated by flash chromatography using EtOAc as eluent. The intermediate was solubilized in THF–H_2O (1:1, 5 mL), LiOH·H_2O (1.62 mmol, 68.0 mg, 3.00 equiv.) was added, and the mixture was stirred for 1 h. THF was removed under reduced pressure and the aqueous phase was washed with DCM (1x 15 mL) and adjusted to pH 3 before extraction with DCM (3x 15 mL). Organic layers were combined, dried over $NaSO_4$ and solvent was removed under reduced pressure. The crude mixture was subsequently solubilized in TFA (10.8 mmol, 0.83 mL, 20 equiv.) and stirred for 1 h at room temperature before TFA was evaporated under nitrogen flow. The crude product was purified by preparative HPLC. (Waters system consisting of 515 pumps in line with a 2487 dual λ absorbance detector and a fraction collector using a Reprosil-Pur 120 C18-AQ column (150 x 20mm, 5 µm, Dr. Maisch GmbH, Basel, Switzerland); 5 mL injection, monitoring with a diode array detector at 220 nm and 254 nm, solvent A = H_2O + 0.1% TFA, solvent B = MeCN + 0.1% TFA, flow rate = 10 mL/min, 0-1 min = 5% B, 1-21 min ramp to 70% B, 21-24 min ramp to 95% B, 24-26 min = 95% B, 26-27 min = 99% B, 24-26 min ramp to 5% B, 26-30 min re-equilibration = 5% B). Individual fractions were analysed by LC-MS. Pure fractions were combined, and the solvent was removed under high vacuum to yield the pure product (145 mg, 81% yield) as a yellow solid.

^{1}H NMR (400 MHz, MeOD) δ = 7.23 – 7.15 (m, 2H), 7.01 – 6.93 (m, 2H), 4.72 (d, J = 2.4 Hz, 2H), 4.28 (dd, J = 10.8, 4.3 Hz, 1H), 4.03 (dd, J = 8.4, 7.1 Hz, 1H), 3.13 - 2.99 (m, 2H), 2.95 (t, J = 2.4 Hz, 1H), 1.60 – 1.38 (m, 2H), 1.21 – 1.05 (m, 1H), 0.82 (dd, J = 15.6, 6.6 Hz, 6H) ppm.

^{13}C NMR (101 MHz, MeOD) δ = 176.8, 169.4, 158.8, 131.5, 128.3, 116.4, 79.7, 76.9, 56.7, 56.0, 52.7, 41.6, 37.8, 25.6, 23.5, 21.6 ppm.

ESI HRMS: $[M+H]^+$: calculated, 333.1809; found, 333.1806

Plasmids

The plasmids pSU18_tycA, pSU18_tycA_W227S, pTrc99a_tycB, pTrc99A_tycC, pMG211-sfp, pTrc99a_tycB1_srfTE_P26G, pTrc99a_srfC_P26G, pCT_tycA_AT and pQE_H6 MBP were previously reported[1,2,6–8]. pET28a-Ec.coaD (pESC106) and pET28a-Ec.coaE (pESC124) were a gift from Tadhg Begley and Erick Strauss (Addgene plasmids #50388 and #50390; RRID: Addgene_50388, http://n2t.net/addgene:50388; RRID: Addgene_50390, http://n2t.net/addgene:50390;)[9]. The vector pET-29b(+) was purchased from Novagen.

Cloning

Expression media and buffers were prepared using purified H_2O (Nanopure system, Barnstead). Media and buffer components, kits, and enzymes were used as received from specified commercial suppliers. Commercial enzymes were purchased from NEB unless stated otherwise. Oligonucleotide primers were obtained from Microsynth AG (Switzerland). All described PCR reactions utilized Phusion HF polymerase (NEB) or Q5 High-Fidelity DNA Polymerase (NEB, for GC or AT rich DNA sequences) using the supplied HF or Q5 reaction buffers according to the manufacturer's protocol (50 µL total volume, 80 ng plasmid DNA, 0.5 µM primer, 0.2 mM dNTPs (Sigma), 1 µL Phusion HF or 0.5 µL Q5 High-Fidelity DNA Polymerase). DNA was purified on agarose gels (1%). DNA bands were extracted with the Gel DNA Recovery Kit (Zymo Research), optionally followed by the DNA clean and concentrator kit (Zymo Research). DNA was eluted from columns utilizing high-purity H_2O. Gibson assembly[10] was performed utilizing 100 bp DNA overlaps and 20 U/µL T5 exonuclease (adjusted from protocol[10]). 5 µL of premixed DNA fragments (~150 ng) were mixed with 5 µL Gibson mix and the reaction was incubated for 10 min at 37 °C followed by incubation at 50 °C for 1 h. Ligations were performed overnight with T4 ligase according to the manufacturer's protocol. Transformations were conducted with electrocompetent *E. coli* BL21 (DE3), XL1Blue, or HM0079[7] cells (50 µL, 80 ng DNA). The cells were rescued with SOC medium (1 mL) and incubated at 37 ◦C for 1 h before plating onto LB agar containing the respective antibiotic. Single colonies were selected and grown in overnight cultures using 5 mL LB medium containing the appropriate antibiotic. Plasmid DNA was isolated from pelleted cells using the ZymoPURE Plasmid Miniprep Kit (Zymo Research) according to the manufacturer's specifications. All cloned variants were verified by Sanger sequencing at Microsynth AG (Switzerland).

SrfA-C variants in pTrc99a

To clone SrfA-C variants identified during evolution from the pCT vector into pTrc99a[7], two fragments each were amplified from pTrc99a_srfC_P26G and the respective variant in pCT with primer pairs R41/R44 and R45/P40, respectively (see Supplementary Table 3). The fragments were assembled by overlap PCR using R41/P40 before inserting into the KnpI and SmaI digested pTrc99a_srfC_P26G by Gibson assembly.

pSU18_tycA_W227S/H743A

To introduce the H743A mutation into pSU18_tycA_W227S, two fragments were PCR amplified from the plasmid using the primer pairs I1/Q1 as well as Q2/I6. The two fragments were assembled using primer pair I1/I6 by overlap PCR and fused in a vector that was cut with EcoRI and BamHI utilizing Gibson assembly to yield the plasmid pSU18_tycA_W227S/H743A.

pSU18_tycB3__W2742S_COM_tycA

The construction of the TycB3 module comprising the COM domain of TycA was described previously[11]. Two fragments were amplified by PCR, one from pTrc99a_tycB_W2742S and one from pSU18_tycA*, using the primer pairs Q6/Q10 and Q8/I6, respectively. The two fragments were assembled by overlap PCR utilizing Q6/Q10. The assembled fragment and pSU18_tycA* were digested with BamHI and XhoI and subsequently ligated to yield pSU18_tycB3__W2742S_COM_tycA.

pCT_srfA-C

Bacillus subtilis genomic DNA (ATCC 21332) was isolated as reported previously[12]. The plasmid pCT_tycA_AT[2,13] was redesigned to display SrfA-C on the surface of yeast via its C-terminus. Five fragments were amplified by PCR. Fragments 1 and 2, encoding for the signal peptide and linker region) were produced using pCT_tycA_AT as a template and the primer pairs SP_f/SP_r or linker_f/linker_r, respectively. The third fragment, encoding SrfA-C without the TE domain, was amplified from the *Bacillus subtilis* genome with the primer pair srfC_f/srfC_r. Fragments 4 and 5, encoding Aga2p and a part of the vector backbone, were generated from pCT_tycA_AT using the primer pair aga2p_f/aga2p_r and vector_f/vector_r, respectively. Fragments 1-4 and fragments 3-5 were assembled by overlap PCR using the

primer pairs SP_f/aga2p_r and linker_f/vector_r, respectively. Fragment 1-4 was digested with EcoRI and XhoI, fragment 3-5 with XhoI and XbaI, and pCT_tycA_AT with EcoRI and XbaI, and the three components were subsequently ligated to yield pCTDN_srfA-C. For cloning purposes, a SalI restriction site was subsequently introduced behind the gene encoding the C domain using a silent mutation so as not to alter the translated protein sequence in the downstream A domain gene. Two fragments were amplified from the pCTDN_srfA-C plasmid using primers P1/P5 and P6/H33, reassembled by overlap PCR using P1/H33, digested with NheI and XhoI, and ligated into the backbone of pCTDN_srfA-C, which had been digested with the same restriction enzymes, to give the display plasmid pCT_srfA-C.

*pCT_srfA-C**

Using pCT_srfA-C as a template, four fragments were generated to introduce three point mutations—N625T, S787Q and N909Q—into the SrfA-C gene. Fragments 1-4 were amplified by PCR utilizing the primer pairs R7/P19, P18/P23, P22/P26, and P25/H33, respectively. All four fragments were subsequently assembled by overlap PCR with R7/H33 to create 100 bp overlaps with the vector backbone, which was generated by digestion of pCT_srfA-C with SalI and HincII. Insert and vector were assembled by Gibson assembly to yield pCT_srfA-C*.

*pCT_srfA-C*_S1003A*

To generate a T domain knockout, two fragments were amplified from pCT_SrfA-C* with the primers R7/P15 and P14/H33, respectively, and then assembled by overlap PCR using R7/H33. The insert was introduced into the SalI and HincII-digested backbone of pCT_srfA-C* by Gibson assembly to yield pCT_srfA-C*_S1003A.

*pCT_srfA-C*_K948A*

To generate a variant with reduced A domain activity, Lys948 in SrfA-C was mutated to alanine. Two fragments were amplified from pCT_srfA-C* with the primer pairs R7/P17 and P16/H33, respectively, and then assembled by overlap PCR using the primers R7/H33. The insert was introduced into the SalI and HincII-digested backbone of pCT_srfA-C* by Gibson assembly to yield pCT_srfA-C*_K948A.

*pCT_srfA-C*_H147A/D151N*

To generate a C domain knockout containing the H147A and D151N mutations, two fragments were amplified from pCT_srfA-C* with the primers P39/P3 and P4/P40, respectively, and then assembled by overlap PCR using P39/P40. The insert was introduced into a KpnI and SmaI-digested backbone pCT_srfA-C* by Gibson assembly to yield pCT_srfA-C*_H147A/D151N.

*pSU18_tycA**

pQE_H6 MBP and pSU18_tycA were used as templates to amplify two fragments by PCR with the primer pair DH1/DH2 and DH3/DH4, respectively. The two fragments were assembled with the primer pair DH5/DH4 by overlap PCR. The resulting fragment and pSU18_tycA were digested with BamHI and EcoRI and subsequently ligated to yield pSU18_tycA*.

pET-29(+)_panK

Genomic DNA was isolated from *E. coli* strain BL21 (DE3). Cells from an overnight culture (5 mL) were pelleted and resuspended in 10 mM Tris-HCl (pH 8.5, 200 µL) and washed with 25:24:1 phenol:$CHCl_3$:isoamyl alcohol (200 µL). The aqueous layer was separated, and the organic phase extracted once with 10 mM Tris-HCl (pH 8.5, 200 µL). All aqueous layers were combined and subsequently washed with 24:1 $CHCl_3$:isoamyl alcohol (360 µL). The aqueous phase was again separated and NH_4OAc was added to reach a final concentration of 0.75 M, followed by EtOH (900 µL). After vortexing, the sample was centrifuged for 10 min at 12,000 x g and 4°C. The pellet was carefully washed twice with 80% EtOH, dried and resuspended in 10 mM Tris-HCl (pH 8.5, 100 µL). *PanK* was amplified from genomic DNA using primer pair DH6/DH7. The fragment as well as pET-29b(+) were digested with NdeI and XhoI and subsequently ligated to yield pET-29b(+)_panK.

pTrc99a_tycB1_ FA

The C domain of SrfA-C_{FA} was amplified utilizing pTrc99a_srfA-C_FA and the primer pair R41/R57. Part of the A domain of TycB1 was amplified from pTrc99a_tycB1_srfTE_P26G utilizing the primer pair R58/S37. The two fragments were assembled by overlap PCR using the primers R41/S37. The plasmid pTrc99a_tycB1_srfTE_P26G was digested with KpnI and SmaI and subsequently ligated to the insert by Gibson assembly to yield pTrc99a_tycB1_ FA.

pTrc99a_tycB_FA

The plasmid was prepared as described for pTrc99a_tycB1_FA but utilizing pTrc99a_tycB as a template instead of pTrc99a_tycB1_srfTE_P26G.

pET21_srfA-C_FA_C_domain

The gene for the C domain of the SrfA-C_{FA} module was synthesized by Bio Basic Inc, and subcloned into a pET21 vector between NcoI and XhoI restriction sites. The vector contains an N-terminal tobacco etch virus (TEV) cleavable octa-histidine tag.

Protein production

Tyrocidine synthetase proteins

His_6-tagged W227S TycA, W227S/H743A TycA, W2742S TycB3$^{COM^{D}\text{-TycA}}$, TycA$^{\beta pY}$, TycB1$_{FA}$, TycB$_{FA}$ and TycC were produced in modified Studier medium[14] (25 g/L LB Miller broth, 25 mM Na_2HPO_4, 25 mM KH_2PO_4, 50 mM NH_4Cl, 5 mM Na_2SO_4, 0.5% v/v glycerol, 20 mM $MgSO_4$) as previously reported for TycA, TycB and TycC[6]. *Escherichia coli* HM0079 cells[7] containing the relevant plasmid for protein expression were obtained from glycerol cell stocks stored at -80 °C and grown overnight at 37 °C in LB Miller broth (5 mL) containing the appropriate antibiotic. The next day, modified Studier medium (800 mL; 2 L baffled flask) was supplemented with the appropriate antibiotic and inoculated with the overnight culture (1/800 v/v). The culture was incubated at 180 rpm and 37 °C until an OD_{600} of 2 was reached. The cultures were cooled to 20 °C and protein expression was induced by addition of IPTG (100 μM) after 10 min. After shaking the induced cultures for 18-22 h at 180 rpm and 20 °C, cells were pelleted by centrifugation at 4,000 x g and 4 °C for 30 min. Pellets were transferred to falcon tubes and frozen (-20 °C) for at least 1 h before protein purification.

The proteins were subsequently purified at 4 °C and pH 8. The pH of all buffers was adjusted with a WTW bench pH/mV meter (routine meter pH526) calibrated according to manufacturer specifications. Cell pellets (from 400 mL cultures) were thawed in a water bath at room temperature for 15 min and resuspended in lysis buffer (40 mL, 50 mM Tris, 500 mM NaCl, 10% v/v glycerol, pH 8.0) by vortexing. For lysis, 1-2 mg/mL lysozyme, 1-2 mg/mL polymyxin B, 0.04 mg/mL RNase A and 0.04 mg/mL DNase I were supplied to the cells and the resulting solution was incubated at 4 °C for 30-45 min before sonication (Dr. Heilscher, UP 200s sonic dismembrator, total sonication time of 10 min at 100% power at intervals of 1 min for sonication

and re-cooling period to not exceed 15 °C). Cellular debris were pelleted by centrifugation at 15,000 x g for 45 min and the supernatant was applied to Ni-NTA resin (Qiagen, 1 mL/40 mL cleared cell lysate) which was pre-equilibrated in lysis buffer. Protein loaded resin was washed 4-5x with 10 column volumes of wash buffer (50 mM Tris-HCl, 500 mM NaCl, 10% v/v glycerol, 20-30 mM imidazole, pH 8.0) applying manual syringe pressure. The His-tagged enzymes were eluted with 3 column volumes elution buffer (50 mM Tris-HCl, 500 mM NaCl, 10% v/v glycerol, 300 mM imidazole, pH 8.0). Subsequently, the proteins were buffer exchanged to storage buffer (50 mM HEPES, 100 mM NaCl, 10 mM $MgCl_2$, 10% v/v glycerol, pH 8.0) utilizing Amicon Ultra-15 Centrifugal Filter Units (Millipore), aliquoted, flash frozen in liquid N_2 and stored at -80 °C until use. Beforehand, protein concentrations were determined utilizing a Nanodrop 2000 spectrophotometer (Thermo Fisher) and extinction coefficients calculated using ProtParam (http://web.expasy.org/protparam/). Protein purity was assessed by SDS-PAGE using a Phast system and 7.5% gels (GE Healthcare) or a BioRad system and 4-20% Mini-PROTEAN® (TGX Stain-Free™) gels (BioRad), visualized with QuickBlue Protein stain (Lubio Science).

*TycA**

The N-terminally His_6-tagged fatty acid donor module was produced and purified as described above but using *E. coli* BL21 (DE3) cells as the production host and buffers at pH 7.4 for purification, and at pH 7.25 for storage. High-resolution mass spectrometric analysis of TycA* and 10-undecynoyl-ppant-TycA*, prepared as described in the main text, was performed by the Functional Genomics Centre Zurich. Protein was desalted using a C18 Zip Tip (Millipore) and analysed in MeOH:2-PrOH:0.2% formic acid (30:20:50). Nano ESI-MS analysis of the samples was performed on a Synapt G2_Si mass spectrometer.

SrfA-C and variants

Protein production and purification of C-terminally His_6-tagged SrfA-C and variants were performed as described for tyrocidine synthetase proteins but using buffers at pH 7.4 for purification and pH 7.25 for storage. Liquid chromatography high-resolution mass spectrometry for SrfA-C and SrfA-C_{FA} was performed at the LOC Mass Spectrometry Service at ETH Zürich on a Bruker maXis II ESI-Qq-TOF-MS by electrospray ionization (ESI), using an Acquity UPLC Protein BEH C4 column (Waters, 2.1mm x 100, 1.7µm). Solvent A = H_2O + 0.1% formic acid, solvent B = MeCN + 0.1% formic acid, flow rate = 0.25 mL/min,

0-1.5 min = 10% B, 1.5-2.5 min ramp to 15% B, 2.5-34 min ramp to 45% B, 34-35 min ramp to 95% B, 35-40 min = 95% B, 40-45 min = 10% B. Enzyme samples were desalted prior using C4 Zip Tips (Millipore) according to the manufacturer's protocol and analysed in 3:1 MeCN:H_2O, 0.1% formic acid.

PanK, PPAT, DPCK and Sfp

The His_6-tagged coenzyme A biosynthetic enzymes PanK, PPAT and DPCK as well as the 4-phosphopantetheine transferase Sfp were produced as described for tyrocidine synthetase proteins but using XL1-Blue cells (PanK) or *E. coli* BL21 (DE3) cells (Sfp, PPAT, DPCK) as the production host. Purification was performed as described for tyrocidine synthetase proteins but at room temperature using buffers at a pH of 7.4. The proteins were stored at -80 °C in storage buffer 2 (50 mM Tris-HCl, 1 mM EDTA, 10% v/v glycerol, pH 7.5). After Ni-NTA purification of Sfp, the buffer was exchanged to storage buffer 2 by dialysis overnight.

C domain of SrfA-C_{FA}

The protein was expressed in *Escherichia coli* BL21 (DE3) cells grown at 37 ºC in lysogeny broth (LB) medium supplemented with 50 µg/mL kanamycin. Protein expression was induced when the culture reached an OD_{600} of 0.6-0.8, by adding 0.5 mM IPTG, and shaking at 16 ºC for 19 h. Cells were harvested by centrifugation before protein purification. For purification of the C domain of SrfA-C_{FA} for later crystallography experiments, cell pellets were resuspended in buffer A (2 mM imidazole, 150 mM NaCl, 2 mM β-mercaptoethanol (β-ME), 25 mM HEPES pH 7.5), and lysed by sonication. The lysate was clarified by centrifugation at 20,000 x g for 30 min at 4 °C. The supernatant was loaded onto a 5 mL HiTrap IMAC FF column (Cytiva) charged with Ni^{2+} equilibrated in buffer A. The column was washed with buffer A, and protein was eluted with a 10-50% gradient of buffer B (250 mM imidazole, 150 mM NaCl, 2 mM β-ME, 25 mM HEPES, pH 7.5). Fractions containing purified protein were pooled and incubated with TEV protease during dialysis into buffer A overnight at room temperature. Protein was re-loaded onto the HiTrap IMAC FF column to remove uncleaved protein, and the flow-through was collected. Protein was then applied to a MonoQ HR 16/60 column (Cytiva) equilibrated in buffer Q1 (0.5 mM TCEP, 25 mM HEPES, pH 7.5). Bound protein was washed with buffer Q1 plus 100 mM NaCl and eluted using a gradient of 100-600 mM NaCl over 80 mL. Protein was pooled, concentrated, and applied to a HiLoad 16/60 Superdex S75 column (GE Healthcare) equilibrated in size exclusion buffer (150 mM NaCl, 0.5 mM TCEP, 25 mM HEPES, pH 7.5).

Purity was accessed by SDS-PAGE and pure fractions were pooled, concentrated to 38.9 mg/mL and flash-frozen in liquid nitrogen, and stored at -80 °C.

In Vitro NRPS Assays

*Biosynthesis of 10-Undecynoyl-L-Leu (**1**)*

Enzymatic fatty acylation of L-Leu was performed *in vitro* in HEPES buffer (100 mM, 100 mM NaCl, 50 mM ATP, 10 mM $MgCl_2$, 0.1 units/mL inorganic pyrophosphatase from baker's yeast, pH 7.25). Freshly loaded 10-undecynoyl-ppant-TycA* was prepared as described in the main text but using HEPES buffer. The reactions were initiated by addition of 10-undecynoyl-ppant-TycA* to solutions of SrfA-C wildtype or variant mixed with L-Leu and incubated at 37 °C in a water bath (final concentrations: 800 µM 10-undecynoyl-pant, 8 µM PanK, 8 µM PPAT, 8 µM DPCK, 2 µM Sfp and 426 µM TycA*, 2 mM L-Leu, 1 µM SrfA-C_{FA} and variants or 2 µM SrfA-C wildtype). For total turnover number experiments, 931 µM TycA* was used. To determine total turnover numbers as well as k_{obs} values, the reactions were performed as time course experiments in triplicate with four biological replicates. A synthetic 10-undecynoyl-L-Leu standard was used for product quantification using the isolated ion count for the product ($[M+H]^+$) in LC-MS chromatograms. Three or four samples (12.5 µL) were analysed between 20 sec and 80 sec and after 15 min (for reactions with SrfA-C variants) or between 0 min and 13 min and after 2 h (for SrfA-C wildtype reactions) and quenched by addition of 87.5 µL MeOH. Product was extracted by vortexing, and samples were cleared from protein precipitate by centrifugation at 16,000 x g for 10 min before adding 1 µL 10M NaOH per 22.5 µL sample (for accelerated hydrolysis of glycerol adducts) and analysis by LC-MS (Waters H-class UPLC/SQD-2).

For LC-MS analyses, 5 µL sampled were injected onto an Acquity UPLC BEH C-18 column (50 x 2.1 mm, 1.7 µm), monitoring ESI^+ for $[M+H]^+$. Solvent A = H_2O + 0.1% formic acid, solvent B = MeCN + 0.1% formic acid, flow rate = 1 mL/min, initial conditions = 5% B, 0-0.2 min = 5% B, 0.2-1.5 min ramp to 80% B, 1.5-2 min ramp to 100% B, 2-3 min = 100% B, 3-3.3 min ramp to 5% B, 3.3-4 min re-equilibration = 5% B.

To analyse the time course of the reaction, aliquots were removed at 0, 20, 45 and 70 sec and at 2.5, 4, 6, 8, 10, 15 and 30 min. Samples were worked up and analysed as described above. To determine k_{obs} values for SrfA-C variants 1 and 14, time course experiments were performed in duplicate with two biological replicates. Two aliquots were removed between 20 s and 80 s, worked up and analysed as described above.

For 10-undecynoyl-ppant-TycA* stability experiments, pre-incubation times for the reaction of TycA* with 10-undecynoic acid, PanK, PPAT, DPCK and Sfp were increased from 5 min to 20 min, and the samples were worked up and analysed after 10 min. Repeated addition of freshly prepared 10-undecynoyl-ppant-TycA* was performed after 10, 20 and 30 min. Samples were analysed after 10, 20, 30 and 40 min for product formation.

*Biosynthesis of O-Propargyl-D-Tyr-L-Leu (**2**)*

Enzymatic reactions for biosynthesis of *O*-propargyl-D-Tyr-L-Leu were performed *in vitro* in HEPES buffer (100 mM, 100 mM NaCl, 50 mM ATP, 10 mM $MgCl_2$, 0.1 units/mL inorganic pyrophosphatase from baker's yeast, pH 7.25) with W227S TycA (final concentration 15 µM), SrfA-C or SrfA-C_{FA} (1.5 µM) and the substrate amino acids (5 mM *O*-propargyl-L-Tyr and 5 mM L-Leu). All reactions were initiated by addition of SrfA-C or SrfA-C_{FA} and incubated at 37 °C in a water bath. To determine k_{obs} and total turnover numbers, the reactions were performed as time course experiments in duplicate or triplicate with three biological replicates. A synthetic *O*-propargyl-D-Tyr-L-Leu standard was used for product quantification at 220 nm. Aliquots (12.5 µL) were removed at 0, 5, 10 and 15 min, and after 5-7 h, and quenched by addition of 87.5 µL MeOH. Product was extracted by vortexing, and samples were cleared from protein precipitate by centrifugation at 16,000 x g for 10 min before addition of 1 µL 10 M NaOH per 22.5 µL sample (for accelerated hydrolysis of glycerol adducts) and analysis by HPLC (Ultimate 3000, Dionex). A background control reaction was prepared containing all reagents and enzymes except SrfA-C or variant; sample preparation, as well as time course experiments, were conducted as described for the reactions above. Rates were correlated for the non-enzymatic formation of *O*-propargyl-L-Tyr-L-Leu.

For HPLC analysis, 5 µL of each extracted sample were injected onto a Dr. Maisch Reprosil-Gold C18 column (100 x 2 mm, 3 µm) and monitored at 220 nm, 254 nm, and 280 nm. Solvent A = H_2O + 0.1% TFA, solvent B = MeCN + 0.1% TFA, flow rate = 0.75 mL/min, 0-1 min = 5% B, 1-5.5 min ramp to 100% B, 5.5-6.5 min = 100% B, 6.5-7 min re-equilibration = 5% B.

*Biosynthesis of 10-Undecynoyl-L-Pro (**3**)*

Enzymatic fatty acylation of L-Pro was performed *in vitro* in HEPES buffer (100 mM, 100 mM NaCl, 50 mM ATP, 10 mM $MgCl_2$, 0.1 units/mL inorganic pyrophosphatase from baker's yeast, pH 7.25). Freshly loaded 10-undecynoyl-ppant-TycA* was prepared as described in the main text but using HEPES buffer. The reactions were initiated by addition of 10-undecynoyl-ppant-TycA* to $TycB1_{FA}$ mixed with L-Pro and incubated at 37 °C in a water bath (final concentrations: 800 µM 10-undecynoyl-pant, 8 µM PanK, 8 µM PPAT, 8 µM DPCK, 2 µM Sfp and 426 µM TycA*, 2 mM L-Pro, 1.7 µM $TycB1_{FA}$). Reactions were performed in duplicate. Aliquots (25 µL) were removed after 4 h and quenched by addition of 75 µL MeOH. Product was extracted and worked up as described above and analysed by LC-MS: 5 µL aliquots were injected onto an Acquity UPLC BEH C-18 column (50 x 2.1 mm, 1.7 µm), monitoring ESI^+ for $[M+H]^+$. Solvent A = H_2O + 0.1% formic acid, solvent B = MeCN + 0.1% formic acid, flow rate = 0.9 mL/min, initial conditions = 5% B, 0-0.2 = 5% B, 0.2-1.5 min ramp to 80% B, 1.5-2 min ramp to 100% B, 2-3 min = 100% B, 3-3.3 min ramp to 5% B, 3.3-4 min re-equilibration = 5% B. The amount of product formed during the reaction was estimated using an authentic 10-undecynoyl-L-Leu standard.

Biosynthesis of Nonribosomal Lipopeptide (**4**)

Biosynthesis of lipopeptide was performed *in vitro* in HEPES buffer (100 mM, 100 mM NaCl, 50 mM ATP, 10 mM $MgCl_2$, 0.1 units/mL inorganic pyrophosphatase from baker's yeast, pH 7.25). Freshly loaded 10-undecynoyl-ppant-TycA* was prepared as described in the main text but using HEPES buffer. The reactions were started by mixing pre-loaded 10-undecynoyl-ppant-TycA* with TycC, $TycB_{FA}$, and all required amino acids and incubated at 37 °C in a water bath (final concentrations: 553 µM 10-undecynoyl-pant, 8 µM PanK, 8 µM PPAT, 8 µM DPCK, 2 µM Sfp and 355 µM TycA*, 2 mM L-Phe, 1 mM L-Pro, 1 mM L-Asn, 1 mM L-Gln, 1 mM L-Tyr, 1 mM L-Val, 1 mM L-Orn, 1 mM L-Leu, 1 µM $TycB_{FA}$ and 1 µM TycC). Reactions were performed in triplicate. Aliquots (25 µL) were removed after 2h and quenched by addition of 75 µL MeOH. Product was extracted, worked up and analysed by LC-MS (10 µL injections) as described for analysis of 10-undecynoyl-L-Pro. For LC-MS analysis $[M+2H]^{2+}$ of the expected product **4** was analysed. Product **4** was also analysed by LC-HRMS/MS (Bruker maxis-ESI-Qq-TOF-MS; ESI) by the LOC Mass Spectrometry Service at ETH Zürich. For product separation, 4 µL were injected onto an Aurora C18 column (AUR2-25075C18A-CSI; 25 cm x 75 µm, C18 1.6 µm) and the following mobile phase gradient was applied:

Solvent A = H_2O + 0.1% formic acid, solvent B = MeCN + 0.1% formic acid, flow rate = 0.3 µL/min, initial conditions = 2% B, 0-120 min ramp to 35% B, 120-122 min ramp to 95% B, 122-130 min = 95% B. $[M+2H]^{2+}$ of **4** was selected for CID (collision energy 31 – 51 eV) with nitrogen as collision gas.

Supplementary Tables

Supplementary Table 1: Smart degenerate codons used to create the C domain library[a,b]

Wildtype residue	Degenerate codon	Encoded amino acids
Glu37	NYT / GAA (8:1)	Phe, Ser, Leu, Pro, Ile, Thr, Val, Ala, Glu
Ile39	RBC	Ile, Thr, Ser, Val, Ala, Gly
Met41	DBC	Phe, Cys, Ile, Thr, Ser, Val, Ala, Gly
Trp143	DYK	Phe, Ser, Leu, Ile, Thr, Met, Val, Ala
Tyr145	DYK / TAC (8:1)	Phe, Ser, Leu, Ile, Thr, Met, Val, Ala, Tyr
Phe155	DYK	Phe, Ser, Leu, Ile, Thr, Met, Val, Ala
Val159	RBC	Ile, Thr, Ser, Val, Ala, Gly

[a] The degenerate base symbols are defined as follows: B = G+T+C; D = A+G+T; N = A+G+T+C; R = A+G; Y = T+C; K = G+T

[b] For combined codons, primers were mixed in the ratio indicated in brackets.

Supplementary Table 2: Data collection and refinement statistics[a]

	**SrfA-C_C_{wt} crystal form 1**	**SrfA-C_C_{FA} crystal form 1**	**SrfA-C_C_{wt} crystal form 2**	**SrfA-C_C_{FA} crystal form 2**
PDB ID	8F7G	8F7H	8F7F	8F7I
Wavelength (Å)	0.9537	0.9792	1	1
Space group	$P2_12_12_1$	$P2_12_12_1$	$P4_32_12$	$P4_32_12$
Cell dimensions				
a, b, c (Å)	75.8, 83.0, 86.0	75.7, 83.7, 86.4	75.7, 75.7, 227.3	76.0, 76.0, 230.0
α, β, γ (°)	90, 90, 90	90, 90, 90	90, 90, 90	90, 90, 90
Resolution (Å)	50-2.4 (2.44 -2.40)	50-1.9 (1.93-1.90)	50-1.62 (1.65-1.62)	50-1.58 (1.61-1.58)
R_{pim}	0.043 (0.280)	0.034 (1.202)	0.082 (0.623)	0.025 (0.403)
I/σI	13.3 (1.8)	35.4 (1.08)	8.8 (0.8)	31.9 (1.31)
$CC_{1/2}$	0.993 (0.784)	0.998 (0.390)	0.975 (0.432)	0.999 (0.709)
Completeness (%)	94.2 (71.8)	100 (100)	98.0 (97.2)	99.4 (100)
Redundancy	8.9 (2.7)	13.2 (13.5)	7.6 (6.0)	6.9 (6.4)
**Refinement**				
Resolution (Å)	46.90-2.40 (2.53-2.40)	47.09-1.93 (1.93-1.90)	45.60-1.62 (1.65-1.62)	36.07-1.58 (1.61-1.58)
No. reflections	42301	43869	84308	93097
R_{work}/R_{free}	0.219/0.265	0.199/0.232	0.179/0.199	0.204/0.216
No. atoms (non-hydrogen)				
Protein	3476	6940	6972	6870
Ligand	glycerol: 6	glycerol: 14	glycerol: 28	glycerol: 14
Water	40	162	697	447
B-factor	45	64	21	34
Mean R.M.S.D.				
Bond length (Å)	0.020	0.015	0.010	0.015
Bond angles (°)	0.5	1.3	1.1	1.3
Clash score	8	4	3	2
Ramachandran favoured	96.68%	98.58%	98.36	98.11%
Ramachandran outliers	0.2%	0%	0%	0%
Side chain rotamer outliers	4.3%	1.8%	0.5%	0.3%

[a] The structures were determined from a single crystal data collection. The values for the highest-resolution shell are represented in parentheses.

Supplementary Table 3: Primers used for construction of single genes and gene libraries[a]

Primer	Sequence 5'→3'
H33	GCT AAA AGT ACA GTG GGA ACA AAG TCG
I1	TTA CGA ATT CAT TAA AGA GGA GAA ATT AAC C
I6	ATC TGG ATC CTA GCG CAG TG
P1	CCT CTA TAC TTT AAC GTC AAG G
P2	GTT CAC TGC TTC CTT GAA CC
P3	CGA CGA TGC CGA AGC ACC ATC CAT TCA AAA TAA TAG CGT GGT AGC TCC ACA CCC ATT C
P4	GGA TGG TGC TTC GGC ATC GTC G
P5	GCA AAA ATT CTC TCT CCC TGT CGT CGA CGA TGT TGA TCG TGC TGA CAG G
P6	GAC AGG GAG AGA GAA TTT TTG C
P14	GCT CGG AGG GCA TGC CTT GAA GGC CAT GAC C
P15	GGT CAT GGC CTT CAA GGC ATG CCC TCC GAG C
P16	CCT TTA ACG ACG AAC GGG GCA GTC AAT AAA CGG CTG CTC C
P17	GGA GCA GCC GTT TAT TGA CTG CCC CGT TCG TCG TTA AAG G
P18	CCG GAA AGC CAA AAG GCA CTA TGA CTA CTC ATG CC
P19	GCC TTT TGG CTT TCC GG
P22	GGA AAG CCG ATC AGC AAT GCC CAA GTT TAT ATT CTG AAC GAG C
P23	GGC ATT GCT GAT CGG CTT TCC
P25	GCA TCA ATC AAT GCC TAC CTT GTG CAA CGA ACG CAG CTT TCA GC
P26	CAC AAG GTA GGC ATT GAT TGA TGC
P39	GCT TCC GGC TCG TAT GTT GTG TGG
P40	GGA TGA GCT GCT GAA TTG CTG TGA GAA GC
Q1	CCA TCC ACG ACC AAC GCA TGA ATT GC
Q2	GCA ATT CAT GCG TTG GTC GTG GAT GG
Q6	CGG TCC TCG AGA ATC CGG AAA ATG AAT TGC AAG AGA TCG
Q8	GAA GGC GAG TAC ACC CCC AGC GAT TTC AGC GTC
Q10	GAC GCT GAA ATC GCT GGG GGT GTA CTC GCC TTC
R7	CTC AAC CTG ATG GCT TCC C
R29	AAG GTA AAA GCT TTG GCC
R30	GGC CAA AGC TTT TAC CTT **NYT** CAA **RBC** ACG **DBC** AAA GTA AAA GGC AGC TTG AAT ATC

R31	GGC CAA AGC TTT CTT GAA CAA **RBC** ACG **DBC** AAA GTA AAA GGC AGC TTG AAT ATC
R32	CCA TCC GTC CAA AAT AAT GTG GTG **MRH** GCT **MRH** CAC CCA TTC AAA GCT TTC TTC
R33	CCA TCC GTC CAA AAT AAT GTG GTG GTA GCT **MRH** CAC CCA TTC AAA GCT TTC TTC
R34	AC ATT ATT TTG GAC GGA TGG TGC **DYK** GGC ATC GTC **RBC** CAG GAT CTA TTT AAG GTA TAC AAT G
R41	CCG ACA TCA TAA CGG TTC TGG
R44	CCC TTC CTG CAT CGG CGA TAG GTA ATA C
R45	GTA TTA CCT ATC GCC GAT GCA GGA AGG G
R57	GCA AAG CTC GAC TCC CGA ATT CTG GAT GAG CTG CTG AAT TGC
R58	GCA ATT CAG CAG CTC ATC CAG AAT TCG GGA GTC GAG CTT TGC
S37	AAC TCC CCG ACG ATC C
DH1	ATT ACG AAT TCA TTA AAG AGG AGA AAT TAA CCA TGC ATC ACC ATC ACC ATC ACC TCG AGA AAA TCG AAG AAG GTA AAC TGG
DH2	AAG ATC AGG CTC GGG GCT CGA ATT AGT CTG CG
DH3	CGC AGA CTA ATT CGA GCC CCG AGC CTG ATC TT
DH4	TAA TGG ATC CTA GCG CAG
DH5	TTA CGA ATT CAT TAA AGA GGA GAA ATT AAC C
DH6	GGG AAT TCC ATA TGA CCG CCA GAA ACA TGC TTA TGA G
DH7	CGC GGA TCC CTC GAG TTT GCG TAG TCT GAC CTC TTC TAC CGC
SP_f	GAT CGA ATT CTA CTT CAT ACA TTT TCA ATT AAG ATG CAG TTA CTT CGC TGT TTT TCA ATA
SP_r	CT CAT ACC AGC TGC TAA AAC GCT AGC AAT AAC AGA AAA TAT TGA AAA ACA GCG AAG TAA CTG C
linker_f	GC TTG CAG GAT GTA CTC GAG GGT GGA GGA GGT AGT GAA CAA AAG CTT ATT TCT GAA GAG GAC TTG G
linker_r	GT TGT CAG TTC CTG GCT TCC TCC ACC ACC TGA TCC ACC ACC TCC CAA GTC CTC TTC AGA AAT AAG C
srfC_f	GC GTT TTA GCA GCT GGT ATG AGT CAA TTT AGC AAG GAT CAG G
srfC_r	CC TCG AGT ACA TCC TGC AAG CCA TCA GAG
aga2p_f	GG AAG CCA GGA ACT GAC AAC TAT ATG CGA G
aga2p_r	G TTA TCA GAT CTC GAC CTA TTA AAA AAC ATA CTG TGT GTT TAT GGG
vector_f	G TAT GTT TTT TAA TAG GTC GAG ATC TGA TAA CAA CAG TG
vector_r	GTG CTC TAG ATT CCG ATG CTG

[a] The degenerate codons used for library construction are bolded. The degenerate base symbols are defined as follows: B = G+T+C; D = A+G+T; M = A+C; N = A+G+T+C; R = A+G; Y = T+C; K = G+T; H = A+T+C

Supplementary Figures

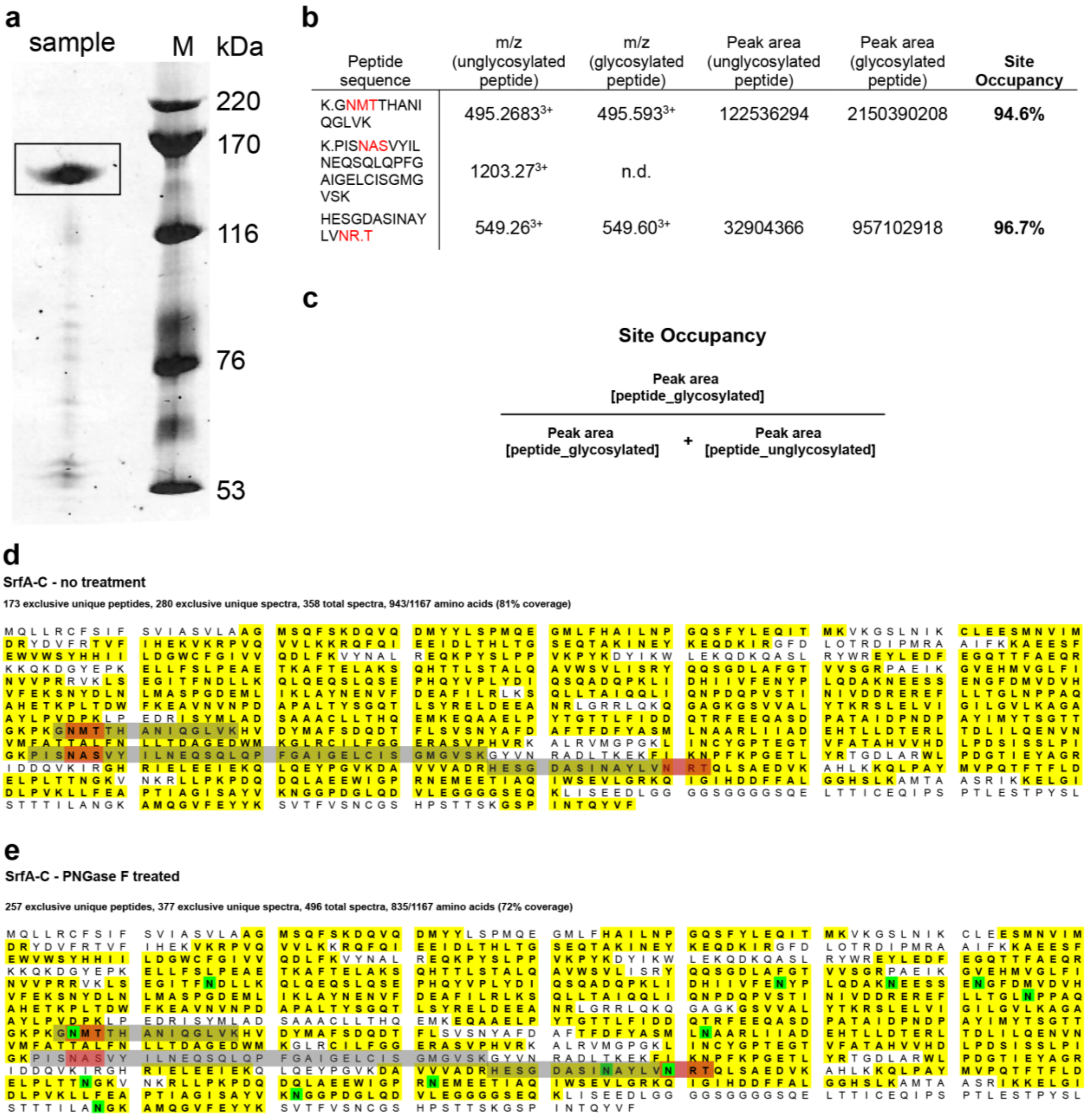

Supplementary Fig. 1: Glycosylation of SrfA-C displayed on yeast. (**a**) SDS-PAGE analysis of SrfA-C after reductive cleavage of the Aga2p complex on yeast. The band for SrfA-C migrates more slowly than expected (observed: 148 kDa versus calculated: 129 kDa), likely due to glycosylation[15–17]. This experiment was repeated twice and gave similar results. (**b**) Tryptic peptides of SrfA-C containing Asn-Xaa-Ser/Thr N-glycosylation motifs (red), where Xaa can be any amino acid except Pro, were analysed before and after deglycosylation with PNGase F. The m/z values for the glycosylated and deglycosylated peptides are listed. Note that PNGase F treatment also results in deamidation of the glycosylated asparagine (+1 Da). *n.d. not detected**.** (**c**) For peptides that were detected, the corresponding peaks in LC chromatograms were extracted for determination of the site occupancy, which corresponds to the degree of glycosylation. The tryptic SrfA-C peptides (yellow) identified by LC-MS/MS before (**d**) and after (**e**) PNGase F treatment. The peptides that contain an N-glycosylation motif (red) are highlighted in grey.

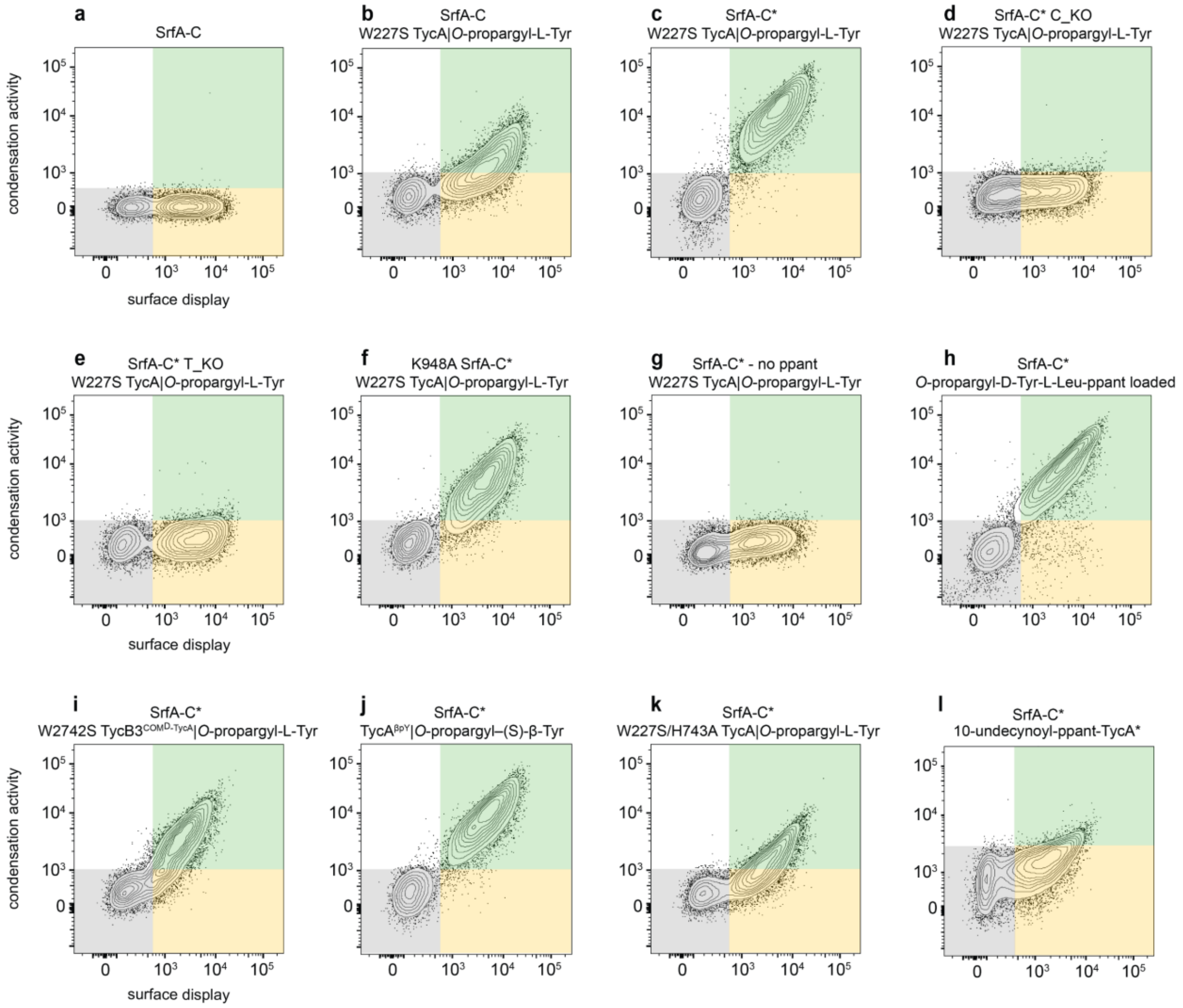

Supplementary Fig. 2: Flow cytometric analysis of SrfA-C constructs on yeast. Flow cytometric analyses are shown as contour plots with outliers (dots). SrfA-C display is plotted (x-axis, FITC) versus condensation activity (y-axis, R-PE). All samples were labelled with FITC and R-PE immediately before analysis. The grey gate indicates cells that do not display any fluorescent label, the yellow gate indicates cells that show a FITC label, and the green gate indicates cells that display both dyes. All condensation reactions were performed with 1.5 µM enzyme and 75 µM amino acids for 15 min, except for the reaction shown in (**l**). (**a**) Yeast displaying SrfA-C, labelled only with FITC. Reactions of W227S TycA, *O*-propargyl-L-Tyr, L-Leu and ATP with yeast displaying (**b**) wildtype SrfA-C; (**c**) non-glycosylated SrfA-C*; (**d**) H147A/D151N SrfA-C*, a variant with an inactive C domain due to the indicated active site mutations; (**e**) S1003A SrfA-C*, with a T domain mutation that prevents pantetheinylation; (**f**) K948A SrfA-C*, which has a mutation that reduces the activity of the acceptor A domain; and (**g**) non-glycosylated SrfA-C* lacking the ppant cofactor. (**h**) Yeast displaying non-glycosylated SrfA-C* post-translationally modified with *O*-propargyl-D-Tyr-L-Leu-ppant. Yeast displaying non-glycosylated SrfA-C* after reaction with upstream modules (**i**) W2742S TycB3$^{\mathrm{COM^{D}\text{-}TycA}}$ and *O*-propargyl-L-Tyr; (**j**) TycA$^{\beta pY}$ and *O*-propargyl-(S)-β-Tyr, (**k**) W227S/H743A TycA and *O*-propargyl-L-Tyr, and (**l**) 10-undecynoyl-ppant-TycA* (final concentration: 195 µM; 15 min reaction).

Percentages of specified gates shown in Supplementary Fig. 2 of the parent cell population, which are single EBY100 cells (and represent 65-80% of the total cell population; see Supplementary Fig. 13 for single cell gating strategy):

	grey	yellow	green
**a**	28.3%	71.6%	0.09%
**b**	21.7%	38.5%	38.4%
**c**	26.1%	2.66%	68.5%
**d**	38.7%	58.8%	0.96%
**e**	24.1%	72.6%	1.73%
**f**	26.1%	9.20%	63.6%
**g**	40.4%	57.4%	0.67%
**h**	20.2%	5.16%	70.6%
**i**	24.1%	17.9%	55.5%
**j**	27.5%	4.16%	67.0%
**k**	24.3%	44.2%	32.3%
**l**	38.6%	55.3%	4.16%

.

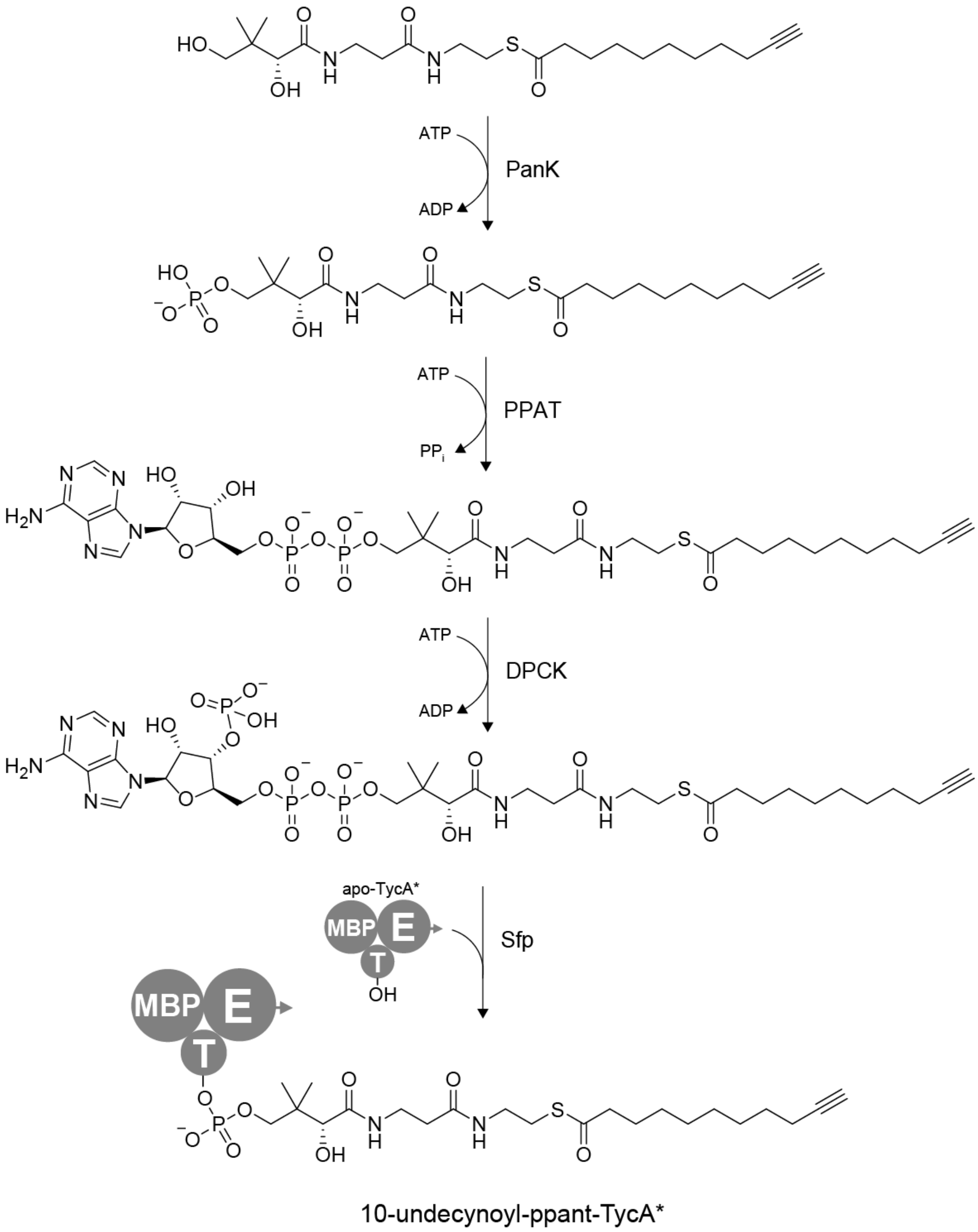

Supplementary Fig. 3: Chemoenzymatic loading of fatty acyl-ppant onto TycA*. 10-Undecynoic acid was first coupled chemically to pant, and 10-undecynoyl-pant was subsequently converted to 10-undecynoyl-CoA and loaded onto the T domain of apo-TycA* in a one-pot enzymatic reaction[18]. The enzyme cascade involves pant phosphorylation with pantothenate kinase (PanK), adenylation of the ppant intermediate with phosphopantetheine adenylyltransferase (PPAT), phosphorylation of the dephospho-CoA with dephospho-coenzyme A kinase (DPCK) and, finally, loading of 10-undecynoyl-ppant onto the apo T domain using the 4-phosphopantetheine transferase Sfp. MBP, maltose binding protein; T and E are the thiolation and epimerase domains, respectively, of TycA. The TycA docking domain is shown as a grey arrow.

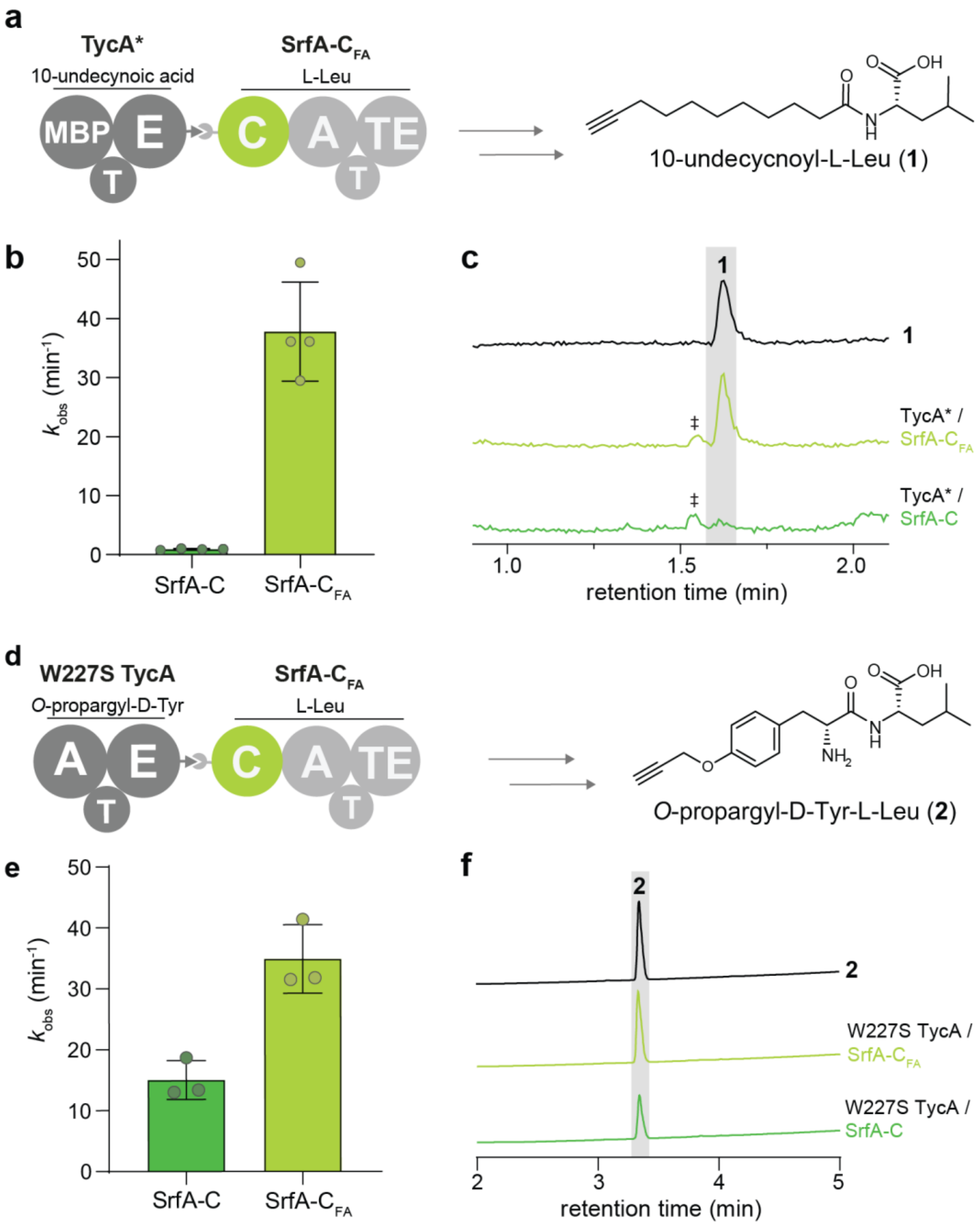

Supplementary Figure 4: In vitro characterization of SrfA-C$_{FA}$ in a bimodular assay. (**a**) 10-Undecynoyl-ppant-TycA* was combined with SrfA-C$_{FA}$ or wt SrfA-C, L-Leu, ATP and Mg^{2+} to produce 10-undecynoyl-L-Leu (**1**). (**b**) Apparent rate constants, k_{obs}, for biosynthesis of **1** with SrfA-C (green) or SrfA-C$_{FA}$ (lime). The data are from independent experiments with four biological replicates (n = 4), each measured three times; the bars show the average ± SD. (**c**) LC-MS chromatograms (total ion count versus time) of a synthetic standard of product **1** (black) and the crude reaction mixtures produced by the bimodular synthetases TycA*/SrfA-C$_{FA}$ (lime) and TycA*/SrfA-C (green) after 15 min and 2 h, respectively (1 µM SrfA-C$_{FA}$ and SrfA-C was used in each case). ‡, unknown contamination that was observed in all biosynthetic reactions (m/z = 409). (**d**) W227S TycA was combined with SrfA-C$_{FA}$ or wt SrfC-A, the required amino acids, ATP and Mg^{2+} for biosynthesis of *O*-propargyl-D-Tyr-L-Leu (**2**). (**e**) Apparent rate constants for biosynthesis of **2** with SrfA-C$_{FA}$ (lime) and SrfA-C (green). Three independent experiments were performed (n = 3), using two to three technical replicates; the bars show the average ± SD. (**f**) HPLC chromatograms of a synthetic standard of dipeptide **2** (black) and the crude reaction mixtures after 6 h.

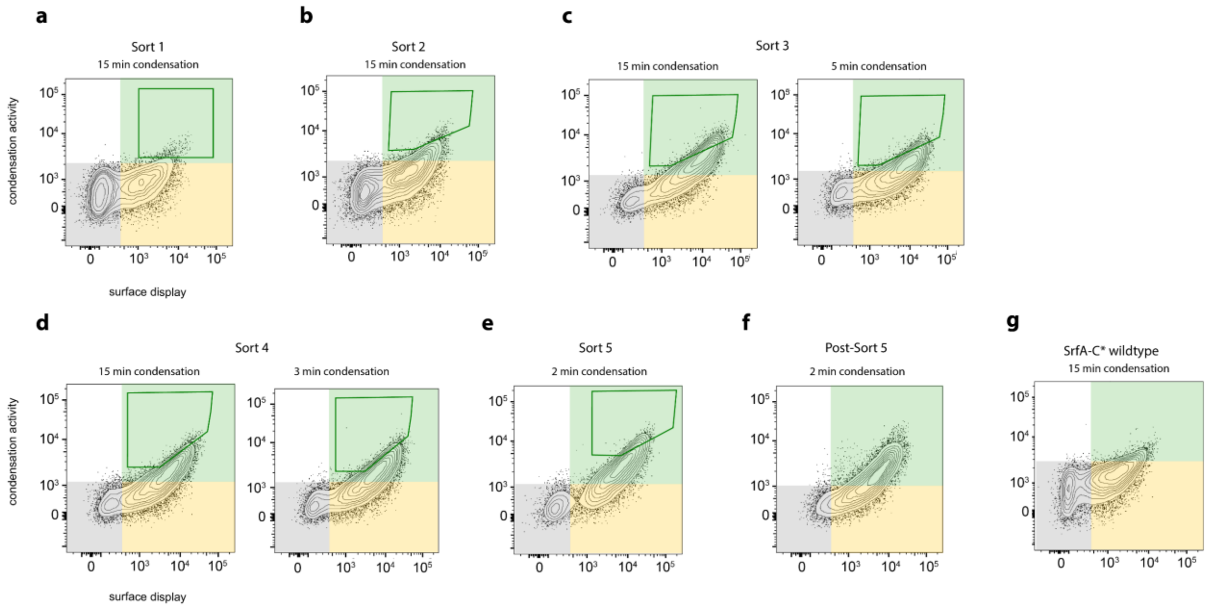

Supplementary Fig. 5: FACS of the SrfA-C* library variants enriched over five rounds of sorting. The FACS data are shown as contour plots with outliers (dots). Yeast display (x-axis, FITC) is plotted versus condensation activity (y-axis, R-PE). The grey gate indicates cells that do not display SrfA-C*, the yellow gate indicates cells that display the NRPS module but do not react with 10-undecynoyl-ppant-TycA*, and the green gates indicates cells displaying catalytically competent variants. The dark green frame indicates cells that were collected during sorting. Contour plots with outliers from **(a)** sort 1, **(b)** sort 2, **(c)** sort 3, **(d)** sort 4, and **(e)** sort 5. The cell pools were reacted with 195 µM 10-undecynoyl-ppant-TycA*, 2 mM L-Leu and 100 µM ATP for 2-15 min and then reanalysed. **(f)** The enriched population of SrfA-C* variants obtained from sort 5 that were incubated with 195 µM 10-undecynoyl-ppant-TycA*, 2 mM L-Leu and 100 µM ATP for 2 min. **(g)** SrfA-C* wildtype displayed on yeast and incubated as described in (**f**), but for 15 min. As indicated, the cells for sorts 3 and 4 were each incubated for several lengths of time. However, the enriched populations were combined prior to the next sort since subsequent flow cytometric analysis indicated that they were similar.

Percentages of specified gates shown in Supplementary Fig. 5 of the parent cell population, which are single EBY100 cells (and represent 65-80% of the total cell population; see Supplementary Fig. 13 for single cell gating strategy):

Cell pool	Reaction time (min)	Ratio of labelled cells		
		grey	yellow	green
**Sort 1**	15	52.4%	42.2%	1.94%
**Sort 2**	15	30.8%	60.4%	6.76%
**Sort 3**	15	14.9%	45.9%	38.0%
**Sort 3**	5	16.0%	56.0%	27.3%
**Sort 4**	15	13.5%	44.0%	40.7%
**Sort 4**	3	15.2%	55.5%	27.1%
**Sort 5**	2	17.8%	48.0%	35.6%
**Post-Sort 5**	2	14.3%	43.2%	39.3%
**SrfA-C* wildtype**	15	38.6%	55.3%	4.16%

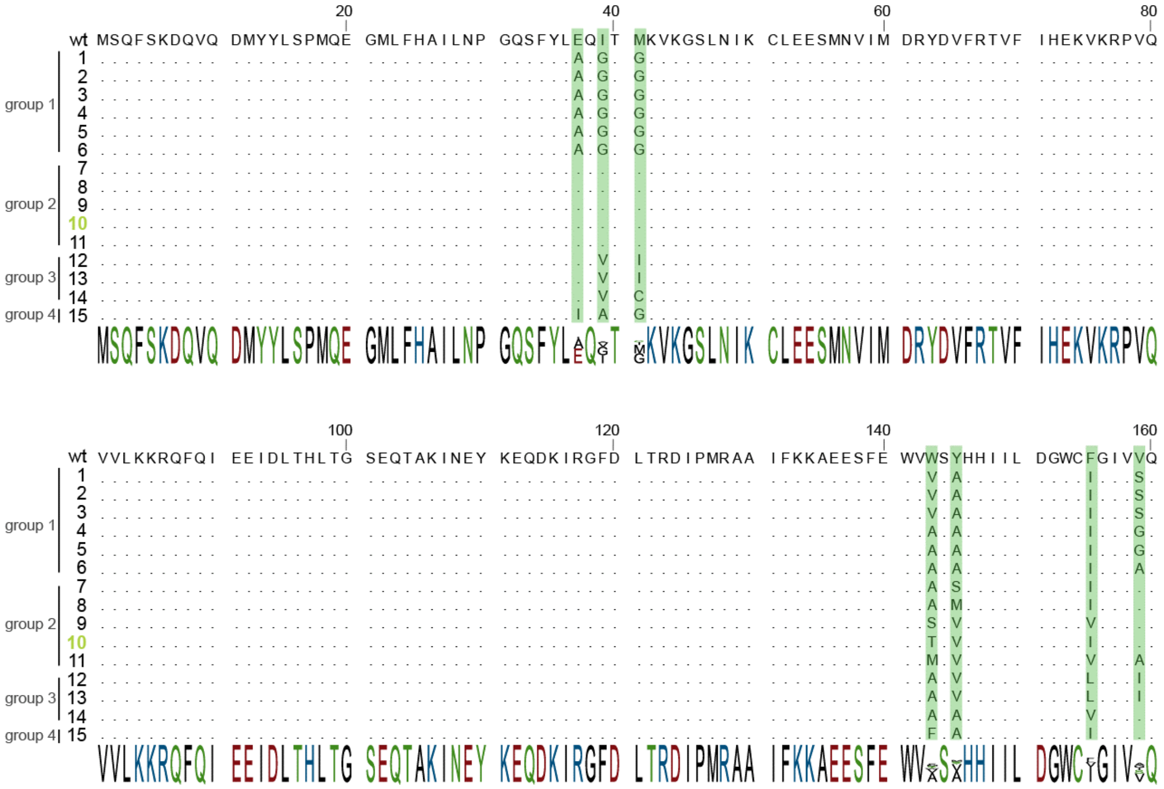

Supplementary Fig. 6: Protein sequences of enriched SrfA-C variants that efficiently process 10-undecynoic acid. Sequence alignment of wt SrfA-C with fifteen variants that were obtained after five rounds of FACS screening against 10-undecynoic acid as the donor substrate. SrfA-C$_{FA}$, which contains three mutations, is variant 10. Residues targeted in the library are highlighted in green. Sequence groups are indicated on the left. Because the original library design did not encode the amino acid methionine at position 41, a cloning artefact must be responsible for retention of wild-type Met41 in the group 2 variants. The ATG codon encoding Met41 may have been introduced by a random single point mutation or due to an improperly digested vector.

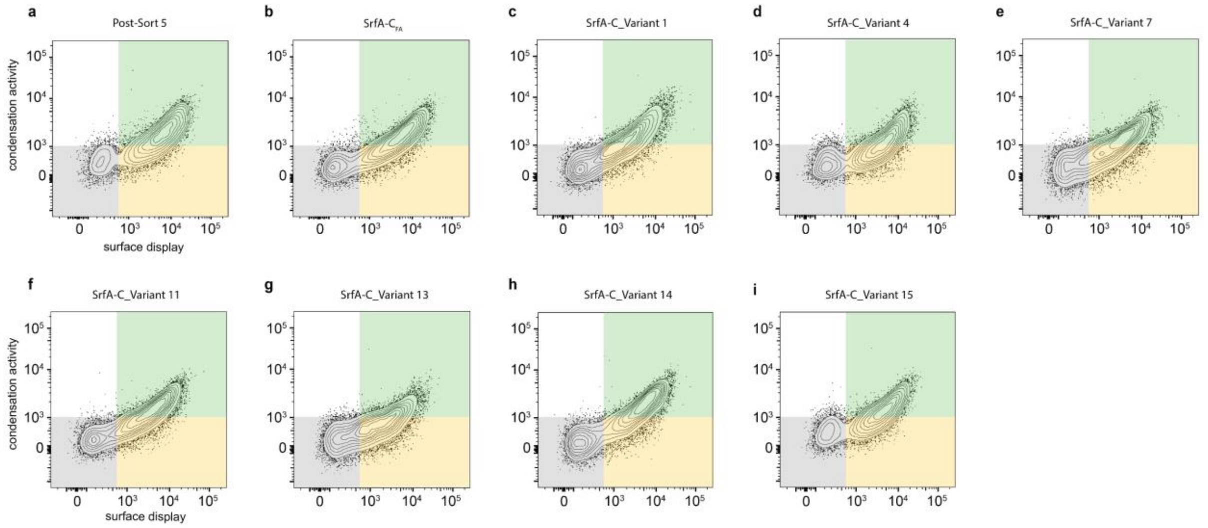

Supplementary Fig. 7: Flow cytometry analysis of SrfA-C single C domain variants on yeast after sort 5. Flow cytometry analysis is shown as contour plot with outliers (dots). Fluorescence activated cell sorting is shown as contour plot with outliers (dots). Yeast display (x-axis, FITC) is plotted versus condensation activity (y-axis, R-PE). The grey gate indicates cells that are not fluorescently labelled, the yellow gate indicates cells labelled with FITC and the green gates indicates cells labelled with both R-PE and FITC. Contour plot of yeast cells displaying **(a)** the cell pool after sort 5, **(b)** SrfA-C_{FA} (Variant 10), **(c)**, SrfA-C_Variant 1, **(d)**, SrfA-C_Variant 4, **(e)**, SrfA-C_Variant 7, **(f)**, SrfA-C_Variant 11, **(g)**, SrfA-C_Variant 13, **(h)**, SrfA-C_Variant 14, **(i)**, SrfA-C_Variant 15, reacted with 50 µM 10-undecynoyl-ppant-TycA* for 5 min.

Percentages of specified gates shown in Supplementary Fig. 7 of the parent cell population, which are single EBY100 cells (and represent 65-80% of the total cell population; see Supplementary Fig. 13 for single cell gating strategy):

**Variant**	**Ratio of labelled cells**		
	grey	yellow	orange
Post-Sort 5 Cell Pool	16.7%	49.2%	34.2%
SrfA-C_{FA} (Variant10)	22.2%	48.9%	26.8%
SrfA-C_Variant 1	25.8%	44.8%	25.8%
SrfA-C_Variant 4	24.8%	60.2%	13.8%
SrfA-C_Variant 7	22.1%	55.2%	17.5%
SrfA-C_Variant 11	22.3%	52.8%	22.0%
SrfA-C_Variant 13	24.9%	62.1%	9.09%
SrfA-C_Variant 14	26.8%	42.3%	26.7%
SrfA-C_Variant 15	20.6%	58.2%	20.7%

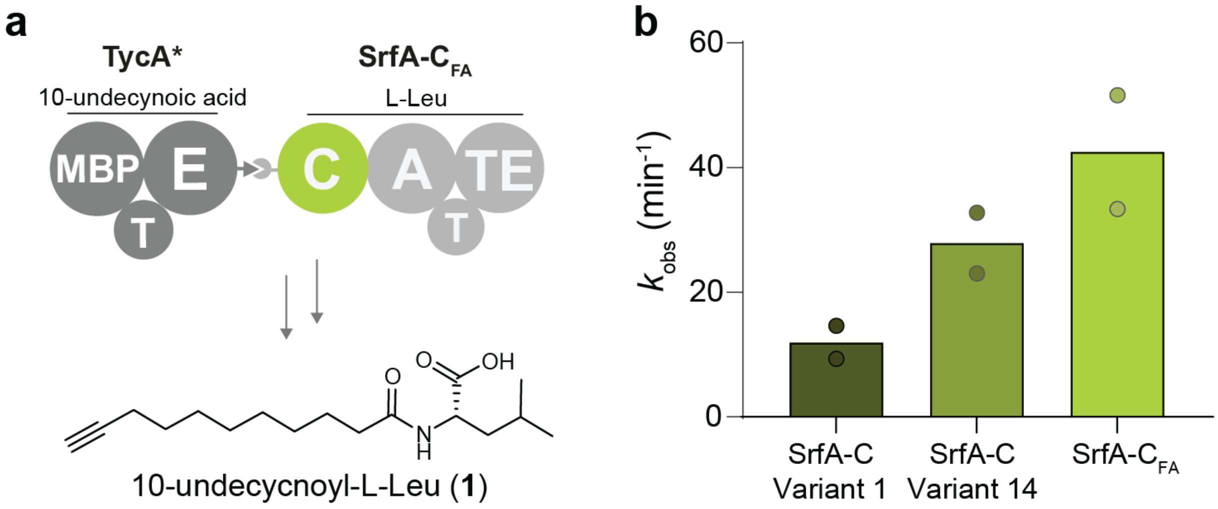

Supplementary Figure 8: In vitro kinetic comparison of SrfA-C$_{FA}$ and variants. (**a**) Single variants that showed high activity in the flow cytometry assay when individually displayed on yeast cells were analysed *in vitro* in a bimodular assay. 10-Undecynoyl-ppant-TycA* was combined with SrfA-C variants 1, 10 (now called SrfA-C$_{FA}$) or 14 and incubated with L-Leu, ATP and Mg^{2+} to produce 10-undecynoyl-L-Leu (**1**). (**b**) Apparent rate constants, k_{obs}, for biosynthesis of **1** with SrfA-C variant 1 (dark green), SrfA-C variant 14 (olive green) or SrfA-C$_{FA}$ (lime). The data show the results of independent experiments with two biological replicas (n = 2), each measured twice. The average rate constants are indicated by the height of the bars.

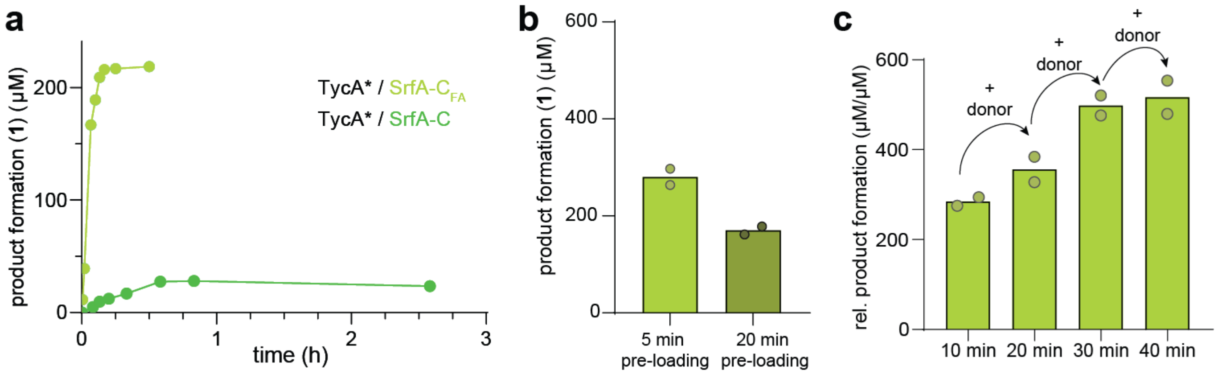

Supplementary Figure 9: Evaluation of 10-undecynoyl-ppant-TycA* stability *in vitro.* (a) Formation of product **1** over time for the bimodular synthetases TycA*/SrfA-C (green) and TycA*/SrfA-C_{FA} (lime). **(b)** Total product obtained after 10 min incubation of SrfA-C_{FA} with 10-undecynoyl-ppant-TycA*, prepared by pre-incubating TycA* with 10-undecynoyl-CoA and Sfp for either 5 or 20 min. The data were obtained from a single biological sample (n = 1); the bars correspond to the average product yield obtained in duplicate measurements. **(c)** Relative amount of product formed (normalized to 1 µM SrfA-C_{FA} in the reaction mixture) after 10, 20, 30 and 40 min incubation of SrfA-C_{FA} with 10-undecynoyl-ppant-TycA*. Freshly prepared 10-undecynoyl-ppant-TycA* (the "donor") was added every 10 min. The data were obtained from a single biological sample (n = 1); the bars corresponded to the average of duplicate measurements. All data were generated with single batches of SrfA-C and SrfA-C_{FA}.

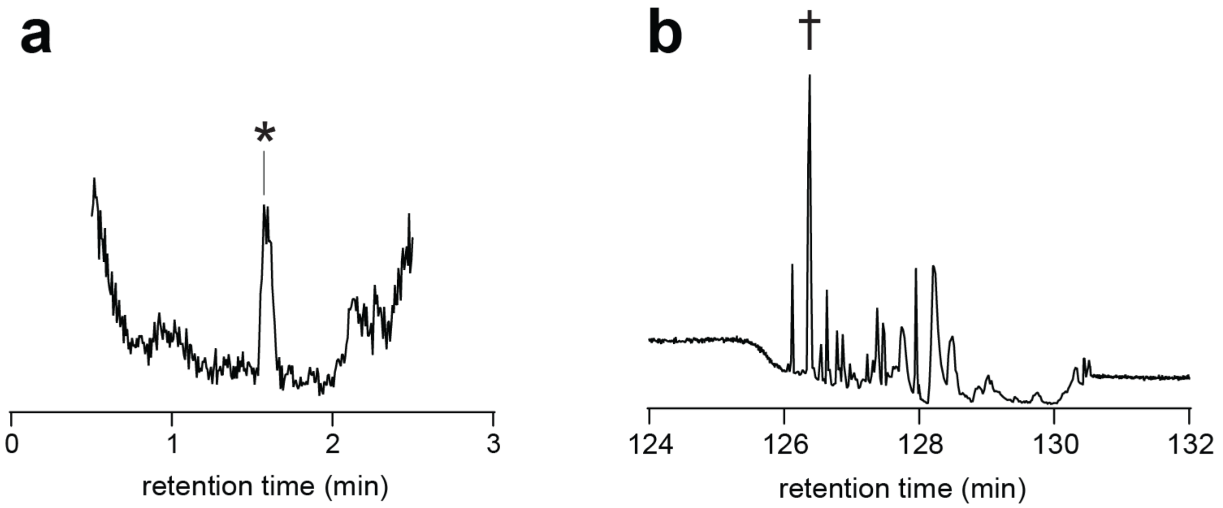

Supplementary Figure 10: Biosynthesis of lipidated products by synthetases containing an engineered C domain. (a) LC-MS chromatogram (total ion count versus retention time) showing 10-undecynoyl-L-Pro (**3**), indicated by the asterisk, produced by the bimodular TycA*/TycB1$_{FA}$ synthetase. **(b)** LC-HRMS/MS chromatogram (total ion count versus retention time) showing 10-undecynoyl-L-Pro-L-Phe-D-Phe-L-Asn-L-Gln-L-Tyr-L-Val-L-Orn-L-Leu (**4**), indicated by the dagger, produced by 10-undecynoyl-ppant-TycA*/TycB$_{FA}$/TycC.

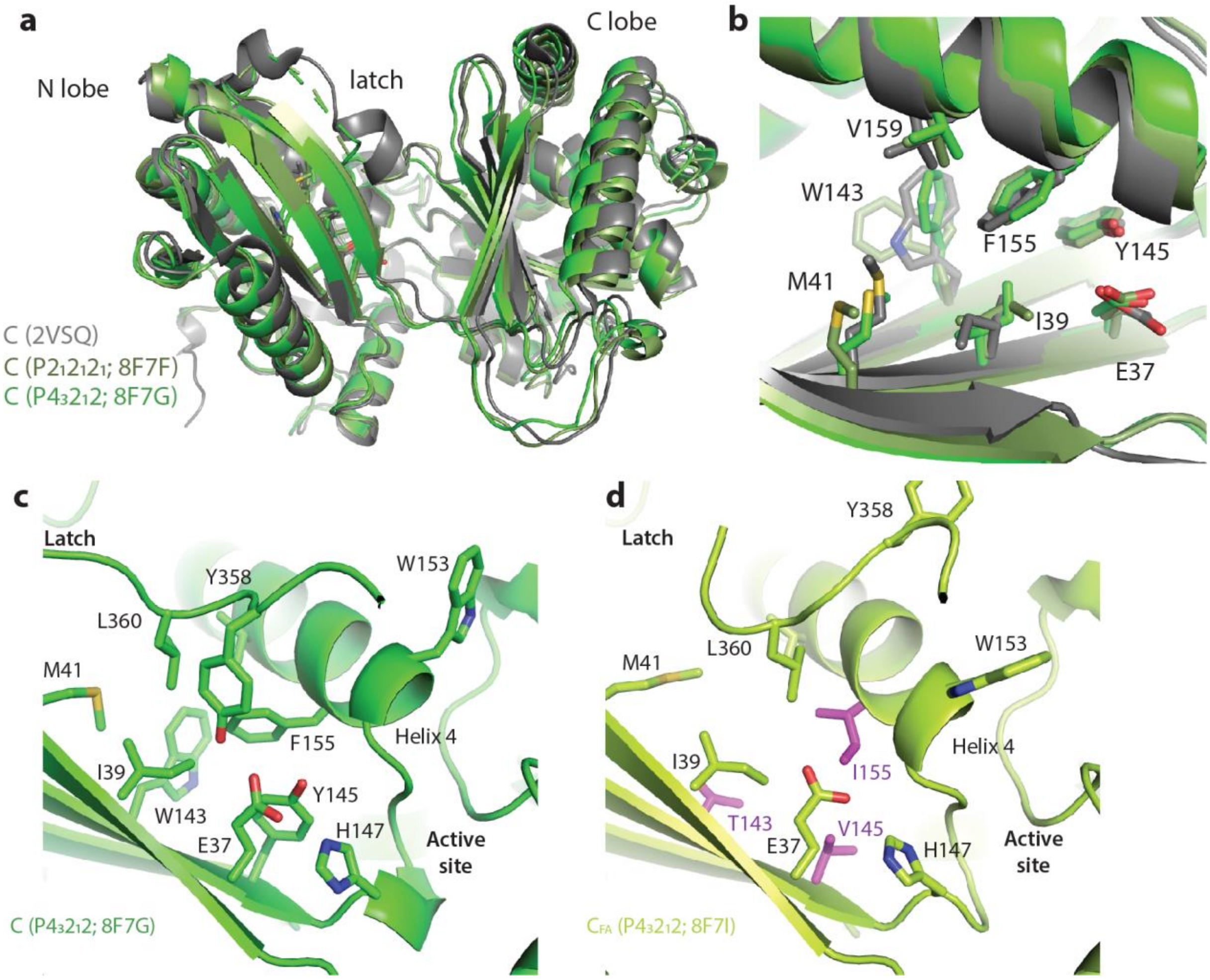

Supplementary Figure 11: Comparison of wild type SrfA-C C domain structures. (a) Overview of the new structures of the wild type extracted C domain (green, dark green) and the C domain from the previously published[19] SrfA-C module (grey). **(b)** Target residues that were mutagenized in the yeast display library. **(c)** Wildtype C domain active site from the current study. **(d)** Active site of the C domain of the SrfA-C_{FA} module (light green) with mutated residues shown as magenta sticks.

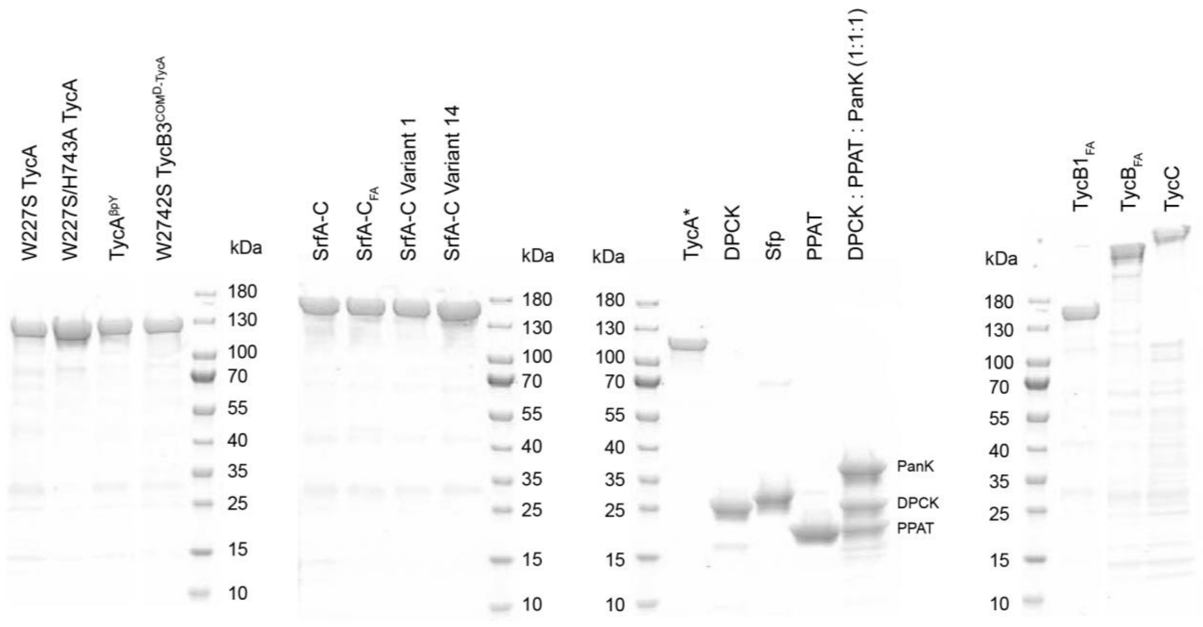

Supplementary Figure 12: SDS-PAGE analysis of Ni-NTA purified proteins. W227S TycA, MW = 123,769 Da; W227S/H743A TycA, MW = 123,703 Da; TycA$^{\beta pY}$, MW = 123,708 Da; W2742S TycB3$^{COM_D\text{-}TycA}$, MW = 123,422 Da; TycB1$_{FA}$, MW = 145,790 Da; TycB$_{FA}$, MW = 406,082 Da; TycC, MW = 725,327 Da; SrfA-C, MW = 144,933 Da; SrfA-C$_{FA}$, MW = 144,751 Da; TycA*, MW = 107,012 Da; DPCK, MW = 24,785 Da; Sfp, MW = 27,088 Da; PPAT, MW = 19,638 Da; PanK, MW = 37,425 Da. For the three enzymes PanK, PPAT and DPCK that convert pantetheine to coenzyme A, an equimolar mixture that was used to post-translationally modify the yeast displayed SrfA-C module was analysed (100 µM each). SDS-PAGE analysis was repeated at least twice and always yielded similar results.

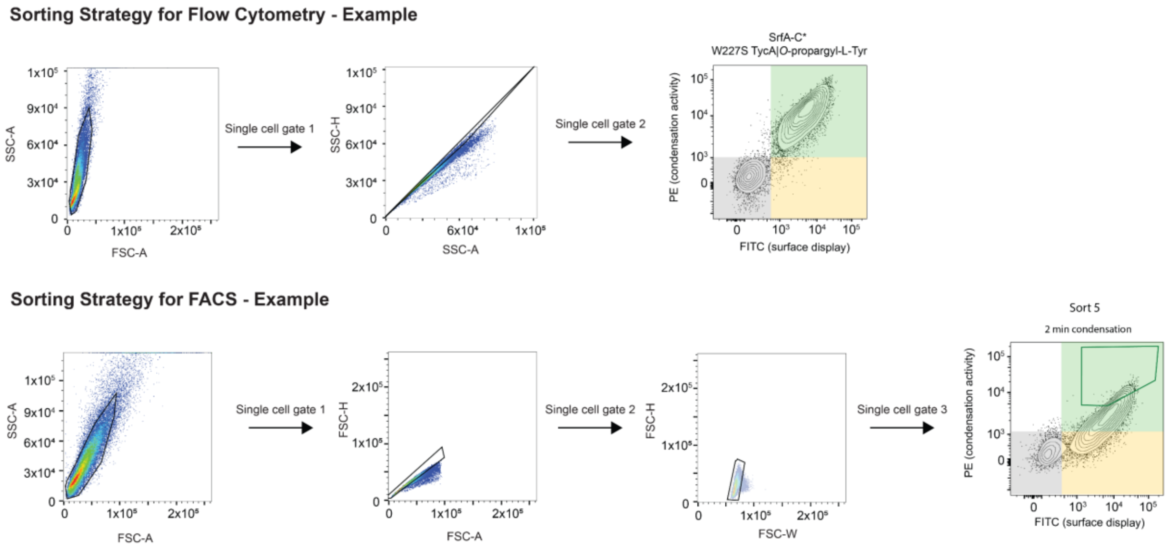

Supplementary Figure 13: Single cell gating strategies for flow cytometry and FACS. SrfA-C displaying yeast cells were gated for isolation of single cells before analysis or sorting. The percentage of single cells usually varied between 65-80% of the total cell population. For flow cytometric analyses and sorting, single yeast cells were isolated via forward and side scatter plots (SSC-A vs FSC-A, SSC-H vs SSC-A or FSC-H vs FSC-A and FSC-H vs FSC-W; gates are represented as black boxes). Single cells were analysed and sorted respective to their negative controls to determine boundaries of the grey gate (cells that are not fluorescently labelled), the yellow gate (cells labelled with FITC) and the green gate (cells labelled with both R-PE and FITC).

Protein Sequences

Protein sequences of W227S TycA,[6] TycA$^{\beta pY}$,[2] TycC,[6] and Sfp[2] were reported previously.

W227S/H743A TycA

MHHHHHHSGR SVANQANLID NKRELEQHAL VPYAQGKSIH QLFEEQAEAF PDRVAIVFEN RRLSYQELNR
KANQLARALL EKGVQTDSIV GVMMEKSIEN VIAILAVLKA GGAYVPIDIE YPRDRIQYIL QDSQTKIVLT
QKSVSQLVHD VGYSGEVVVL DEEQLDARET ANLHQPSKPT DLAYVIYTSG TTGKPKGTML EHKGIANLQS
FFQNSFGVTE QDRIGLFASM SFDASVSEMF MALLSGASLY ILSKQTIHDF AAFEHYLSEN ELTIITLPPT
YLTHLTPERI TSLRIMITAG SASSAPLVNK WKDKLRYINA YGPTETSICA TIWEAPSNQL SVQSVPIGKP
IQNTHIYIVN EDLQLLPTGS EGELCIGGVG LARGYWNRPD LTAEKFVDNP FVPGEKMYRT GDLAKWLTDG
TIEFLGRIDH QVKIRGHRIE LGEIESVLLA HEHITEAVVI AREDQHAGQY LCAYYISQQE ATPAQLRDYA
AQKLPAYMLP SYFVKLDKMP LTPNDKIDRK ALPEPDLTAN QSQAAYHPPR TETESILVSI WQNVLGIEKI
GIRDNFYSLG GDSIQAIQVV ARLHSYQLKL ETKDLLNYPT IEQVALFVKS TTRKSDQGII AGNVPLTPIQ
KWFFGKNFTN TGHWNQSSVL YRPEGFDPKV IQSVMDKIIE HHDALRMVYQ HENGNVVQHN RGLGGQLYDF
FSYNLTAQPD VQQAIEAETQ RLHSSMNLQE GPLVKVALFQ TLHGDHLFLA IHALVVDGIS WRILFEDLAT
GYAQALAGQA ISLPEKTDSF QSWSQWLQEY ANEADLLSEI PYWESLESQA KNVSLPKDYE VTDCKQKSVR
NMRIRLHPEE TEQLLKHANQ AYQTEINDLL LAALGLAFAE WSKLAQIVIH LEGHGREDII EQANVARTVG
WFTSQYPVLL DLKQTAPLSD YIKLTKENMR KIPRKGIGYD ILKHVTLPEN RGSLSFRVQP EVTFNYLGQF
DADMRTELFT RSPYSGGNTL GADGKNNLSP ESEVYTALNI TGLIEGGELV LTFSYSSEQY REESIQQLSQ
SYQKHLLAII AHCTEKKEVE RTPSDFSVKG LQMEEMDDIF ELLANTLR

The W227S and H743A mutations are highlighted in red.

W2742S TycB3$^{COM^{D}\text{-}TycA}$

MHHHHHHLEN PENELQEIEM LTAAEKQMLL VAFNDTHREY RADQTIQQLF EELAEKMPEH TALVFEEKRM
SFRELNERAN QLAAVLREKG VGPAQIVALL VERSAEMVIA TLATLKAGGA FLPVDPDYPE ERIRYMLEDS
QAKLVVTHAH LLHKVSSQSE VVDVDDPGSY ATQTDNLPCA NTPSDLAYII YTSGTTGKPK GVMLEHKGVA
NLQAVFAHHL GVTPQDRAGH FASISFDASV WDMFGPLLSG ATLYVLSRDV INDFQRFAEY VRDNAITFLT
LPPTYAIYLE PEQVPSLRTL ITAGSASSVA LVDKWKEKVT YVNGYGPTES TVCATLWKAK PDEPVETITI
GKPIQNTKLY IVDDQLQLKA PGQMGELCIS GLSLARGYWN RPELTAEKFV DNPFVPGTKM YRTGDLARWL
PDGTIEYLGR IDHQVKIRGH RVELGEVESV LLRYDTVKEA AAITHEDDRG QAYLCAYYVA EGEATPAQLR
AYMENELPNY MVPAFFIQLE KMPLTPNDKI DRKALPKPNQ EENRTEQYAA PQTELEQLLA GIWADVLGIK
QVGTQDNFFE LGGDSIKAIQ VSTRLNASGW TLAMKELFQY PTIEEAALRV IPNSRESEQG VVEGEIALTP
IQKWFFANNF TDRHHWNQAV MLFREDGFDE GLVRQAFQQI VEHHDALRMV YKQEDGAIKQ INRGLTDERF
RFYSYDLKNH ANSEARILEL SDQIQSSIDL EHGPLVHVAL FATKDGDHLL VAIHHLVVDG VSWRILFEDF
SSAYSQALHQ QEIVLPKKTD SFKDWAAQLQ KYADSDELLR EVAYWHNLET TTTTAALPTD FVTADRKQKH
TRTLSFALTV PQTENLLRHV HHAYHTEMND LLLTALGLAV KDWAHTNGVV INLEGHGRED IQNEMNVTRT
IGWFTSQYPV VLDMEKAEDL PYQIKQTKEN LRRIPKKGIG YEILRTLTTS QLQPPLAFTL RPEISFNYLG
QFESDGKTGG FTFSPLGTGQ LFSPESERVF LLDISAMIED GELRISVGYS RLQYEEKTIA SLADSYRKHL
LGIIEHCMAK EEGEYTPSDF SVKGLQMEEM DDIFELLANT LR

The green, underlined sequence corresponds to the COM domain of TycA

TycA*

```
MHHHHHHLEK IEEGKLVIWI NGDKGYNGLA EVGKKFEKDT GIKVTVEHPD KLEEKFPQVA ATGDGPDIIF
WAHDRFGGYA QSGLLAEITP DKAFQDKLYP FTWDAVRYNG KLIAYPIAVE ALSLIYNKDL LPNPPKTWEE
IPALDKELKA KGKSALMFNL QEPYFTWPLI AADGGYAFKY ENGKYDIKDV GVDNAGAKAG LTFLVDLIKN
KHMNADTDYS IAEAAFNKGE TAMTINGPWA WSNIDTSKVN YGVTVLPTFK GQPSKPFVGV LSAGINAASP
NKELAKEFLE NYLLTDEGLE AVNKDKPLGA VALKSYEEEL AKDPRIAATM ENAQKGEIMP NIPQMSAFWY
AVRTAVINAA SGRQTVDEAL KDAQTNSSPE PDLTANQSQA AYHPPRTETE SILVSIWQNV LGIEKIGIRD
NFYSLGGDSI QAIQVVARLH SYQLKLETKD LLNYPTIEQV ALFVKSTTRK SDQGIIAGNV PLTPIQKWFF
GKNFTNTGHW NQSSVLYRPE GFDPKVIQSV MDKIIEHHDA LRMVYQHENG NVVQHNRGLG GQLYDFFSYN
LTAQPDVQQA IEAETQRLHS SMNLQEGPLV KVALFQTLHG DHLFLAIHHL VVDGISWRIL FEDLATGYAQ
ALAGQAISLP EKTDSFQSWS QWLQEYANEA DLLSEIPYWE SLESQAKNVS LPKDYEVTDC KQKSVRNMRI
RLHPEETEQL LKHANQAYQT EINDLLLAAL GLAFAEWSKL AQIVIHLEGH GREDIIEQAN VARTVGWFTS
QYPVLLDLKQ TAPLSDYIKL TKENMRKIPR KGIGYDILKH VTLPENRGSL SFRVQPEVTF NYLGQFDADM
RTELFTRSPY SGGNTLGADG KNNLSPESEV YTALNITGLI EGGELVLTFS YSSEQYREES IQQLSQSYQK
HLLAIIAHCT EKKEVERTPS DFSVKGLQME EMDDIFELLA NTLR
```

The green, underlined sequence encodes the maltose binding protein (MBP). The TycA T and E domains are shown in black.

SrfA-C wildtype

```
MSQFSKDQVQ DMYYLSPMQE GMLFHAILNP GQSFYLEQIT MKVKGSLNIK CLEESMNVIM DRYDVFRTVF
IHEKVKRPVQ VVLKKRQFQI EEIDLTHLTG SEQTAKINEY KEQDKIRGFD LTRDIPMRAA IFKKAEESFE
WVWSYHHIIL DGWCFGIVVQ DLFKVYNALR EQKPYSLPPV KPYKDYIKWL EKQDKQASLR YWREYLEDFE
GQTTFAEQRK KQKDGYEPKE LLFSLPEAET KAFTELAKSQ HTTLSTALQA VWSVLISRYQ QSGDLAFGTV
VSGRPAEIKG VEHMVGLFIN VVPRRVKLSE GITFNDLLKQ LQEQSLQSEP HQYVPLYDIQ SQADQPKLID
HIIVFENYPL QDAKNEESSE NGFDMVDVHV FEKSNYDLNL MASPGDEMLI KLAYNENVFD EAFILRLKSQ
LLTAIQQLIQ NPDQPVSTIN IVDDREREFL LTGLNPPAQA HETKPLTDWF KEAVNVNPDA PALTYSGQTL
SYRELDEEAN RLGRRLQKQG AGKGSVVALY TKRSLELVIG ILGVLKAGAA YLPVDPKLPE DRISYMLADS
AAACLLTHQE MKEQAAELPY TGTTLFIDDQ TRFEEQASDP ATAIDPNDPA YIMYTSGTTG KPKGNMTTHA
NIQGLVKHVD YMAFSDQDTF LSVSNYAFDA FTFDFYASML NAARLIIADE HTLLDTERLT DLILQENVNV
MFATTALFNL LTDAGEDWMK GLRCILFGGE RASVPHVRKA LRVMGPGKLI NCYGPTEGTV FATAHVVHDL
PDSISSLPIG KPISNASVYI LNEQSQLQPF GAIGELCISG MGVSKGYVNR ADLTKEKFIK NPFKPGETLY
RTGDLARWLP DGTIEYAGRI DDQVKIRGHR IELEEIEKQL QEYPGVKDAV VVADRHESGD ASINAYLVNR
TQLSAEDVKA HLKKQLPAYM VPQTFTFLDE LPLTTNGKVN KRLLPKPDQD QLAEEWIGPR NEMEETIAQI
WSEVLGRKQI GIHDDFFALG GHSLKAMTAA SRIKKELGID LPVKLLFEAP TIAGISAYVK NGGPDGLQDV
TIMNQDQEQI IFAFPGVLGY GLMYQNLSSR LPSYKLCAFD FIEEEDRLDR YADLIQKLQP EGPLTLFGYS
AGCSLAFEAA KKLEGQGRIV QRIIMVDSYK KQGVSDLDGR TVESDVEALM NVNRDNEALN SEAVKQGLKQ
KTHAFYSYYV NLISTGQVKA DIDLLTSGAD FDIPEWLASW EEATTGAYRM KRGFGTHAEM LQGETLDRNA
GILLEFLNTQ TVTVSGSRSH HHHHH
```

The blue, underlined sequence corresponds to the C domain of wild-type SrfA-C. The residues that were targeted for mutagenesis are highlighted in cyan.

SrfA-C$_{FA}$

MSQFSKDQVQ DMYYLSPMQE GMLFHAILNP GQSFYLEQIT MKVKGSLNIK CLEESMNVIM DRYDVFRTVF
IHEKVKRPVQ VVLKKRQFQI EEIDLTHLTG SEQTAKINEY KEQDKIRGFD LTRDIPMRAA IFKKAEESFE
WVTSVHHIIL DGWCIGIVVQ DLFKVYNALR EQKPYSLPPV KPYKDYIKWL EKQDKQASLR YWREYLEDFE
GQTTFAEQRK KQKDGYEPKE LLFSLPEAET KAFTELAKSQ HTTLSTALQA VWSVLISRYQ QSGDLAFGTV
VSGRPAEIKG VEHMVGLFIN VVPRRVKLSE GITFNDLLKQ LQEQSLQSEP HQYVPLYDIQ SQADQPKLID
HIIVFENYPL QDAKNEESSE NGFDMVDVHV FEKSNYDLNL MASPGDEMLI KLAYNENVFD EAFILRLKSQ
LLTAIQQLIQ NPDQPVSTIN IVDDREREFL LTGLNPPAQA HETKPLTDWF KEAVNVNPDA PALTYSGQTL
SYRELDEEAN RLGRRLQKQG AGKGSVVALY TKRSLELVIG ILGVLKAGAA YLPVDPKLPE DRISYMLADS
AAACLLTHQE MKEQAAELPY TGTTLFIDDQ TRFEEQASDP ATAIDPNDPA YIMYTSGTTG KPKGNMTTHA
NIQGLVKHVD YMAFSDQDTF LSVSNYAFDA FTFDFYASML NAARLIIADE HTLLDTERLT DLILQENVNV
MFATTALFNL LTDAGEDWMK GLRCILFGGE RASVPHVRKA LRVMGPGKLI NCYGPTEGTV FATAHVVHDL
PDSISSLPIG KPISNASVYI LNEQSQLQPF GAIGELCISG MGVSKGYVNR ADLTKEKFIK NPFKPGETLY
RTGDLARWLP DGTIEYAGRI DDQVKIRGHR IELEEIEKQL QEYPGVKDAV VVADRHESGD ASINAYLVNR
TQLSAEDVKA HLKKQLPAYM VPQTFTFLDE LPLTTNGKVN KRLLPKPDQD QLAEEWIGPR NEMEETIAQI
WSEVLGRKQI GIHDDFFALG GHSLKAMTAA SRIKKELGID LPVKLLFEAP TIAGISAYVK NGGPDGLQDV
TIMNQDQEQI IFAFPGVLGY GLMYQNLSSR LPSYKLCAFD FIEEEDRLDR YADLIQKLQP EGPLTLFGYS
AGCSLAFEAA KKLEGQGRIV QRIIMVDSYK KQGVSDLDGR TVESDVEALM NVNRDNEALN SEAVKQGLKQ
KTHAFYSYYV NLISTGQVKA DIDLLTSGAD FDIPEWLASW EEATTGAYRM KRGFGTHAEM LQGETLDRNA
GILLEFLNTQ TVTVSGSRSH HHHHH

The blue, underlined sequence corresponds to the engineered specific C domain of SrfA-C$_{FA}$ with the mutations conferring fatty acid specificity highlighted in cyan. For production in *E. coli*, the mutations introduced to prevent glycosylation were not included in the sequence encoding the A domain since *E. coli* does not recognize eukaryotic N-glycosylation motifs.

TycB1$_{FA}$

MSQFSKDQVQ DMYYLSPMQE GMLFHAILNP GQSFYLEQIT MKVKGSLNIK CLEESMNVIM DRYDVFRTVF
IHEKVKRPVQ VVLKKRQFQI EEIDLTHLTG SEQTAKINEY KEQDKIRGFD LTRDIPMRAA IFKKAEESFE
WVTSVHHIIL DGWCIGIVVQ DLFKVYNALR EQKPYSLPPV KPYKDYIKWL EKQDKQASLR YWREYLEDFE
GQTTFAEQRK KQKDGYEPKE LLFSLPEAET KAFTELAKSQ HTTLSTALQA VWSVLISRYQ QSGDLAFGTV
VSGRPAEIKG VEHMVGLFIN VVPRRVKLSE GITFNDLLKQ LQEQSLQSEP HQYVPLYDIQ SQADQPKLID
HIIVFENYPL QDAKNEESSE NGFDMVDVHV FEKSNYDLNL MASPGDEMLI KLAYNENVFD EAFILRLKSQ
LLTAIQQLIQ NSGVELCQIP LLTEAETSQL LAKRTETAAD YPAATMHELF SRQAEKTPEQ VAVVFADQHL
TYRELDEKSN QLARFLRKKG IGTGSLVGTL LDRSLDMIVG ILGVLKAGGA FVPIDPELPA ERIAYMLTHS
RVPLVVTQNH LRAKVTTPTE TIDINTAVIG EESRAPIESL NQPHDLFYII YTSGTTGQPK GVMLEHRNMA
NLMRFTFDQT NIAFHEKVLQ YTTCSFDVCY QEIFSTLLSG GQLYLITNEL RRHVEKLFAF IQEKQISILS
LPVSFLKFIF NEQDYAQSFP RCVKHIITAG EQLVVTHELQ KYLRQHRVFL HNHYGPSETH VVTTCTMDPG
QAIPELPPIG KPISNTGIYI LDEGLQLKPE GIVGELYISG ANVGRGYLHQ PELTAEKFLD NPYQPGERMY
RTGDLARWLP DGQLEFLGRI DHQVKIRGHR IELGEIESRL LNHPAIKEAV VIDRADETGG KFLCAYVVLQ
KALSDEEMRA YLAQALPEYM IPSFFVTLER IPVTPNGKTD RRALPKPEGS AKTKADYVAP TTELEQKLVA
IWEQILGVSP IGIQDHFFTL GGHSLKAIQL ISRIQKECQA DVPLRVLFEQ PTIQALAAYV EGGSDGLQDV
TIMNQDQEQI IFAFPGVLGY GLMYQNLSSR LPSYKLCAFD FIEEEDRLDR YADLIQKLQP EGPLTLFGYS
AGCSLAFEAA KKLEGQGRIV QRIIMVDSYK KQGVSDLDGR TVESDVEALM NVNRDNEALN SEAVKQGLKQ
KTHAFYSYYV NLISTGQVKA DIDLLTSGAD FDIPEWLASW EEATTGAYRM KRGFGTHAEM LQGETLDRNA
GILLEFLNTQ TVTVSGSRSH HHHHH

The blue, underlined sequence corresponds to the transplanted C domain from SrfA-C$_{FA}$ with the mutations conferring fatty acid specificity highlighted in cyan.

TycB$_{FA}$

MSQFSKDQVQ DMYYLSPMQE GMLFHAILNP GQSFYLEQIT MKVKGSLNIK CLEESMNVIM DRYDVFRTVF
IHEKVKRPVQ VVLKKRQFQI EEIDLTHLTG SEQTAKINEY KEQDKIRGFD LTRDIPMRAA IFKKAEESFE
WVTSVHHIIL DGWCIGIVVQ DLFKVYNALR EQKPYSLPPV KPYKDYIKWL EKQDKQASLR YWREYLEDFE
GQTTFAEQRK KQKDGYEPKE LLFSLPEAET KAFTELAKSQ HTTLSTALQA VWSVLISRYQ QSGDLAFGTV
VSGRPAEIKG VEHMVGLFIN VVPRRVKLSE GITFNDLLKQ LQEQSLQSEP HQYVPLYDIQ SQADQPKLID
HIIVFENYPL QDAKNEESSE NGFDMVDVHV FEKSNYDLNL MASPGDEMLI KLAYNENVFD EAFILRLKSQ
LLTAIQQLIQ NSGVELCQIP LLTEAETSQL LAKRTETAAD YPAATMHELF SRQAEKTPEQ VAVVFADQHL
TYRELDEKSN QLARFLRKKG IGTGSLVGTL LDRSLDMIVG ILGVLKAGGA FVPIDPELPA ERIAYMLTHS
RVPLVVTQNH LRAKVTTPTE TIDINTAVIG EESRAPIESL NQPHDLFYII YTSGTTGQPK GVMLEHRNMA
NLMHFTFDQT NIAFHEKVLQ YTTCSFDVCY QEIFSTLLSG GQLYLITNEL RRHVEKLFAF IQEKQISILS
LPVSFLKFIF NEQDYAQSFP RCVKHIITAG EQLVVTHELQ KYLRQHRVFL HNHYGPSETH VVTTCTMDPG
QAIPELPPIG KPISNTGIYI LDEGLQLKPE GIVGELYISG ANVGRGYLHQ PELTAEKFLD NPYQPGERMY
RTGDLARWLP DGQLEFLGRI DHQVKIRGHR IELGEIESRL LNHPAIKEAV VIDRADETGG KFLCAYVVLQ
KALSDEEMRA YLAQALPEYM IPSFFVTLER IPVTPNGKTD RRALPKPEGS AKTKADYVAP TTELEQKLVA
IWEQILGVSP IGIQDHFFTL GGHSLKAIQL ISRIQKECQA DVPLRVLFEQ PTIQALAAYV EGGEESAYLA
IPQAEPQAYY PVSSAQKRML ILNQLDPHST VYNLPVAMIL EGTLDKARLE HAISNLVARH ESLRTSFHTI
NGEPVSRIHE QGHLPIVYLE TAEEQVNEVI LGFMQPFDLV TAPLCRVGLV KLAENRHVLI IDMHHIISDG
VSSQLILNDF SRLYQNKALP EQRIHYKDFA VWEKAWTQTT DYQKQEKYWL DRFAGEIPVL NLPMDYPRPA
VQSFEGERYL FRTEKQLLES LQDVAQKTGT TLYMVLLAAY HVLLSKYSGQ DDVMIGTVTA GRVHPDTESM
TGMFVNTLAM RNQSAPTKTF RQFLLEVKDN TLAAFEHGQY PFEELVEKLA IQRNRSRNPL FDTLFILQNM
DADLIELDGL TVTPYVPEGE VAKFDLSLEA SENQAGLSFC FEFCTKLFAR ETIERMSLHY LQILQAVSAN
TEQELAQIEM LTAHEKQELL VHFNDTAALY PAESTLSQLF EDQAQKTPEQ TAVVFGDKRL TYRELNERAN
QLAHTLRAKG VQAEQSVGIM AQRSLEMAIG IIAILKAGGA YVPIDPDYPN ERIAYMLEDC EARLVLTQQQ
LAEKMTANVE CLYLDEEGSY SPQTENIEPI HTAADLAYII YTSGTTGRPK GVMVEHRGIV NSVTWNRDEF
ALSVRDSGTL SLSFAFDAFA LTFFTLIVSG STVVLMPDHE AKDPIALRNL IAAWECSYVV FVPSMFQAIL
ECSTPADIRS IQAVMLGGEK LSPKLVQLCK AMHPQMSVMN AYGPTESSVM ATYLRDTQPD QPITIGRPIA
NTAIYIVDQH HQLLPVGVVG EICIGGHGLA RGYWKKPELT AEKFVANPAV PGERMYKTGD LGRWLHDGTI
DFIGRVDDQI KVRGYRIEVG EIEAVLLAYD QTNEAIVVAY QDDRGDSYLA AYVTGKTAIE ESELRAHLLR
ELPAYMVPTY LIQLDAFPLT PNGKVDRKAL PKPEGKPATG AAYVAPATEV EAKLVAIWEN ALGISGVGVL
DHFFELGGHS LKAMTVVAQV HREFQIDLLL KQFFAAPTIR DLARLIEHSE QAAGAAIQPA EPQAYYPVSS
AQQRMYLLHQ LEGAGISYNT PGIIMLEGKL DREQLANALQ ALVDRHDILR TSFEMVGDEL VQKIHDRVAV
NMEYVTAEEQ QIDDLFHAFV RPFDLSVPPL LRMSLVKLAD ERHLLLYDMH HIAADAASIT ILFDELAELY
QGRELPEMRI QYKDFAVWQK ALHESDAFKQ QEAYWLSTFA GNITAVDVPT DFPRPAVKSF AGGQVTLSMD
QELLSALHEL AAHTNTTLFM VLLAAYNVLL AKYAGQDDII VGTPISGRSR AELAPVVGMF VHTLAIRNKP
TAEKTFKQFL QEVKQNALDA FDHQDYPFES LVEKLGIPRD PGRNPLFDTM FILQNDELHA KTLDQLVYRP
YESDSALDVA KFDLSFHLTE RETDLFLRLE YCTKLFKQQT VERMAHHFLQ ILRAVTANPE NELQEIEMLT
AAEKQMLLVA FNDTHREYRA DQTIQQLFEE LAEKMPEHTA LVFEEKRMSF RELNERANQL AAVLREKGVG
PAQIVALLVE RSAEMVIATL ATLKAGGAFL PVDPDYPEER IRYMLEDSQA KLVVTHAHLL HKVSSQSEVV
DVDDPGSYAT QTDNLPCANT PSDLAYIIYT SGTTGKPKGV MLEHKGVANL QAVFAHHLGV TPQDRAGHFA
SISFDASVWD MFGPLLSGAT LYVLSRDVIN DFQRFAEYVR DNAITFLTLP PTYAIYLEPE QVPSLRTLIT
AGSASSVALV DKWKEKVTYV NGYGPTESTV CATLWKAKPD EPVETITIGK PIQNTKLYIV DDQLQLKAPG
QMGELCISGL SLARGYWNRP ELTAEKFVDN PFVPGTKMYR TGDLARWLPD GTIEYLGRID HQVKIRGHRV
ELGEVESVLL RYDTVKEAAA ITHEDDRGQA YLCAYYVAEG EATPAQLRAY MENELPNYMV PAFFIQLEKM
PLTPNDKIDR KALPKPNQEE NRTEQYAAPQ TELEQLLAGI WADVLGIKQV GTQDNFFELG GDSIKAIQVS
TRLNASGWTL AMKELFQYPT IEEAALRVIP NSRESEQGVV EGEIALTPIQ KWFFANNFTD RHHWNQAVML
FREDGFDEGL VRQAFQQIVE HHDALRMVYK QEDGAIKQIN RGLTDERFRF YSYDLKNHAN SEARILELSD
QIQSSIDLEH GPLVHVALFA TKDGDHLLVA IHHLVVDGVS WRILFEDFSS AYSQALHQQE IVLPKKTDSF
KDWAAQLQKY ADSDELLREV AYWHNLETTT TTAALPTDFV TADRKQKHTR TLSFALTVPQ TENLLRHVHH
AYHTEMNDLL LTALGLAVKD WAHTNGVVIN LEGHGREDIQ NEMNVTRTIG WFTSQYPVVL DMEKAEDLPY
QIKQTKENLR RIPKKGIGYE ILRTLTTSQL QPPLAFTLRP EISFNYLGQF ESDGKTGGFT FSPLGTGQLF
SPESERVFLL DISAMIEDGE LRISVGYSRL QYEEKTIASL ADSYRKHLLG IIEHCMAKEE GEYTPSDLGD
EELSMEELEN ILEWIGSRSH HHHHH

The blue, underlined sequence corresponds to the transplanted C domain from SrfA-C_{FA} with the mutations conferring fatty acid specificity highlighted in cyan.

PanK (pantothenate kinase)

MSIKEQTLMT PYLQFDRNQW AALRDSVPMT LSEDEIARLK GINEDLSLEE VAEIYLPLSR LLNFYISSNL RRQAVLEQFL GTNGQRIPYI ISIAGSVAVG KSTTARVLQA LLSRWPEHRR VELITTDGFL HPNQVLKERG LMKKKGFPES YDMHRLVKFV SDLKSGVPNV TAPVYSHLIY DVIPDGDKTV VQPDILILEG LNVLQSGMDY PHDPHHVFVS DFVDFSIYVD APEDLLQTWY INRFLKFREG AFTDPDSYFH NYAKLTKEEA IKTAMTLWKE INWLNLKQNI LPTRERASLI LTKSANHAVE EVRLRKLEHH HHHH

This CoA biosynthetic enzyme was expressed heterologously in *E. coli* from plasmid pET-29b(+)_panK, upon induction with IPTG.

PPAT (phosphopantetheine adenylyltransferase)

HHHHHHSSGL VPRGSHMQKR AIYPGTFDPI TNGHIDIVTR ATQMFDHVIL AIAASPSKKP MFTLEERVAL AQQATAHLGN VEVVGFSDLM ANFARNQHAT VLIRGLRAVA DFEYEMQLAH MNRHLMPELE SVFLMPSKEW SFISSSLVKE VARHQGDVTH FLPENVHQAL MAKLA

This CoA biosynthetic enzyme was expressed heterologously in *E. coli* from plasmid pET28a-Ec.coaD (pESC106), which was a gift from Tadhg Begley & Erick Strauss (Addgene plasmid #50388; http://n2t.net/addgene:50388; RRID:Addgene_50388)[9].

DPCK (dephospho-coenzyme A kinase)

MGSSHHHHHH SSGLVPRGSH MRYIVALTGG IGSGKSTVAN AFADLGINVI DADIIARQVV EPGAPALHAI ADHFGANMIA ADGTLQRRAL RERIFANPEE KNWLNALLHP LIQQETQHQI QQATSPYVLW VVPLLVENSL YKKANRVLVV DVSPETQLKR TMQRDDVTRE HVEQILAAQA TREARLAVAD DVIDNNGAPD AIASDVARLH AHYLQLASQF VSQEKP

This CoA biosynthetic enzyme was expressed heterologously in *E. coli* from plasmid pET28a-Ec.coaE (pESC124) was a gift from Tadhg Begley & Erick Strauss (Addgene plasmid #50390; http://n2t.net/addgene:50390; RRID:Addgene_50390)[9].

DNA sequence of the yeast display plasmid *pCT_SrfA-C**

TTTCTTTTTTTACTTTCTATTTTTAATTTATATATTTATATTAAAAAATTTAAATTATAATTATTTTTATAGCAC
GTGATGAAAAGGACCCAGGTGGCACTTTTCGGGGAAATGTGCGCGGAACCCCTATTTGTTTATTTTTCTAAATAC
ATTCAAATATGTATCCGCTCATGAGACAATAACCCTGATAAATGCTTCAATAATATTGAAAAAGGAAGAGTATGA
GTATTCAACATTTCCGTGTCGCCCTTATTCCCTTTTTTGCGGCATTTTGCCTTCCTGTTTTTGCTCACCCAGAAA
CGCTGGTGAAAGTAAAAGATGCTGAAGATCAGTTGGGTGCACGAGTGGGTTACATCGAACTGGATCTCAACAGCG
GTAAGATCCTTGAGAGTTTTCGCCCCGAAGAACGTTTTCCAATGATGAGCACTTTTAAAGTTCTGCTATGTGGCG
CGGTATTATCCCGTATTGACGCCGGGCAAGAGCAACTCGGTCGCCGCATACACTATTCTCAGAATGACTTGGTTG
AGTACTCACCAGTCACAGAAAAGCATCTTACGGATGGCATGACAGTAAGAGAATTATGCAGTGCTGCCATAACCA
TGAGTGATAACACTGCGGCCAACTTACTTCTGACAACGATCGGAGGACCGAAGGAGCTAACCGCTTTTTTTCACA
ACATGGGGGATCATGTAACTCGCCTTGATCGTTGGGAACCGGAGCTGAATGAAGCCATACCAAACGACGAGCGTG
ACACCACGATGCCTGTAGCAATGGCAACAACGTTGCGCAAACTATTAACTGGCGAACTACTTACTCTAGCTTCCC
GGCAACAATTAATAGACTGGATGGAGGCGGATAAAGTTGCAGGACCACTTCTGCGCTCGGCCCTTCCGGCTGGCT
GGTTTATTGCTGATAAATCTGGAGCCGGTGAGCGTGGGTCTCGCGGTATCATTGCAGCACTGGGGCCAGATGGTA
AGCCCTCCCGTATCGTAGTTATCTACACGACGGGCAGTCAGGCAACTATGGATGAACGAAATAGACAGATCGCTG
AGATAGGTGCCTCACTGATTAAGCATTGGTAACTGTCAGACCAAGTTTACTCATATATACTTTAGATTGATTTAA
AACTTCATTTTTAATTTAAAAGGATCTAGGTGAAGATCCTTTTTGATAATCTCATGACCAAAATCCCTTAACGTG
AGTTTTCGTTCCACTGAGCGTCAGACCCCGTAGAAAAGATCAAAGGATCTTCTTGAGATCCTTTTTTTCTGCGCG
TAATCTGCTGCTTGCAAACAAAAAAACCACCGCTACCAGCGGTGGTTTGTTTGCCGGATCAAGAGCTACCAACTC
TTTTTCCGAAGGTAACTGGCTTCAGCAGAGCGCAGATACCAAATACTGTTCTTCTAGTGTAGCCGTAGTTAGGCC
ACCACTTCAAGAACTCTGTAGCACCGCCTACATACCTCGCTCTGCTAATCCTGTTACCAGTGGCTGCTGCCAGTG
GCGATAAGTCGTGTCTTACCGGGTTGGACTCAAGACGATAGTTACCGGATAAGGCGCAGCGGTCGGGCTGAACGG
GGGGTTCGTGCACACAGCCCAGCTTGGAGCGAACGACCTACACCGAACTGAGATACCTACAGCGTGAGCTATGAG
AAAGCGCCACGCTTCCCGAAGGGAGAAAGGCGGACAGGTATCCGGTAAGCGGCAGGGTCGGAACAGGAGAGCGCA
CGAGGGAGCTTCCAGGGGGAAACGCCTGGTATCTTTATAGTCCTGTCGGGTTTCGCCACCTCTGACTTGAGCGTC
GATTTTTGTGATGCTCGTCAGGGGGGCGGAGCCTATGGAAAAACGCCAGCAACGCGGCCTTTTTACGGTTCCTGG
CCTTTTGCTGGCCTTTTGCTCACATGTTCTTTCCTGCGTTATCCCCTGATTCTGTGGATAACCGTATTACCGCCT
TTGAGTGAGCTGATACCGCTCGCCGCAGCCGAACGACCGAGCGCAGCGAGTCAGTGAGCGAGGAAGCGGAAGAGC
GCCCAATACGCAAACCGCCTCTCCCCGCGCGTTGGCCGATTCATTAATGCAGCTGGCACGACAGGTTTCCCGACT
GGAAAGCGGGCAGTGAGCGCAACGCAATTAATGTGAGTTAGCTCACTCATTAGGCACCCCAGGCTTTACACTTTA
TGCTTCCGGCTCGTATGTTGTGTGGAATTGTGAGCGGATAACAATTTCACACAGGAAACAGCTATGACCATGATT
ACGCCAAGCTCGAAATTAACCCTCACTAAAGGGAACAAAAGCTGGTACCAATTCCTTGAATTTTCAAAAATTCTT
ACTTTTTTTTTGGATGGACGCAAAGAAGTTTAATAATCATATTACATGGCATTACCACCATATACATATCCATAT
ACATATCCATATCTAATCTTACTTATATGTTGTGGAAATGTAAAGAGCCCCATTATCTTAGCCTAAAAAAACCTT
CTCTTTGGAACTTTCAGTAATACGCTTAACTGCTCATTGCTATATTGAAGTACGGATTAGAAGCCGCCGAGCGGG
TGACAGCCCTCCGAAGGAAGACTCTCCTCCGTGCGTCCTCGTCTTCACCGGTCGCGTTCCTGAAACGCAGATGTG
CCTCGCGCCGCACTGCTCCGAACAATAAAGATTCTACAATACTAGCTTTTATGGTTATGAAGAGGAAAAATTGGC
AGTAACCTGGCCCCACAAACCTTCAAATGAACGAATCAAATTAACAACCATAGGATGATAATGCGATTAGTTTTT
TAGCCTTATTTCTGGGGTAATTAATCAGCGAAGCGATGATTTTTGATCTATTAACAGATATATAAATGCAAAAAC
TGCATAACCACTTTAACTAATACTTTCAACATTTTCGGTTTGTATTACTTCTTATTCAAATGTAATAAAAGTATC
AACAAAAAATTGTTAATATACCTCTATACTTTAACGTCAAGGAGAAAAAACCCCGGATCGAATTCCCTACTTCAT
ACATTTTCAATTAAGATGCAGTTACTTCGCTGTTTTTCAATATTTTCTGTTATTGCTAGCGTTTTAGCAGCTGGT
ATGAGTCAATTTAGCAAGGATCAGGTTCAAGATATGTATTACCTATCGCCGATGCAGGAAGGGATGCTTTTTCAT
GCCATCCTGAATCCCGGCCAAAGCTTTTACCTTGAACAAATCACGATGAAAGTAAAAGGCAGCTTGAATATCAAA
TGTCTTGAAGAAAGCATGAATGTGATCATGGACCGGTACGATGTATTTCGTACCGTGTTCATTCACGAAAAAGTA
AAAAGACCTGTCCAAGTCGTATTGAAAAAACGGCAGTTCCAAATAGAAGAAATCGATCTGACACACTTAACGGGC
AGCGAGCAAACAGCCAAAATCAATGAGTACAAAGAACAGGATAAGATCAGGGGTTTTGATTTGACGCGGGATATT
CCGATGCGGGCAGCCATTTTCAAGAAAGCTGAAGAAAGCTTTGAATGGGTGTGGAGCTACCACCACATTATTTTG
GACGGATGGTGCTTCGGCATCGTCGTACAGGATCTATTTAAGGTATACAATGCTCTGCGCGAACAAAAGCCGTAC
AGCCTGCCGCCCGTCAAACCGTATAAAGACTACATCAAGTGGCTTGAAAAGCAGGATAAACAAGCATCGCTCCGC
TACTGGCGCGAGTACTTGGAGGACTTTGAAGGACAAACGACGTTTGCGGAGCAAAGGAAGAAACAGAAGGACGGC
TATGAGCCGAAAGAGCTGCTCTTTTCACTGCCGGAGGCGGAAACAAAGGCTTTTACTGAGCTTGCAAAATCGCAG

CATACCACTTTAAGTACGGCGCTGCAGGCAGTCTGGAGCGTATTGATCAGCCGCTATCAGCAGTCTGGCGATTTG
GCCTTCGGTACAGTTGTTTCAGGGCGTCCCGCGGAAATCAAAGGCGTTGAACATATGGTCGGGCTGTTTATCAAC
GTTGTCCCGAGACGTGTGAAGCTGTCTGAGGGTATCACATTTAACGACTTGCTCAAGCAGCTGCAGGAGCAATCG
CTGCAGTCTGAGCCGCACCAATATGTGCCGCTTTATGACATCCAAAGCCAGGCCGATCAGCCGAAACTGATTGAC
CACATCATTGTTTTTGAGAACTATCCGCTTCAGGATGCGAAAAATGAAGAAAGCAGTGAAAACGGCTTTGATATG
GTGGATGTTCATGTTTTTGAGAAGTCGAATTATGATCTCAACCTGATGGCTTCCCCGGGAGATGAGATGCTGATT
AAGCTTGCCTATAATGAGAATGTGTTTGATGAGGCGTTTATCCTGCGCTTGAAATCTCAGCTTCTCACAGCAATT
CAGCAGCTCATCCAGAATCCGGATCAGCCTGTCAGCACGATCAACATCGTCGACGACAGGGAGAGAGAATTTTTG
CTAACCGGCTTAAACCCGCCGGCTCAAGCTCATGAAACAAAGCCTCTGACGGATTGGTTCAAGGAAGCAGTGAAC
GTCAATCCGGATGCACCGGCGCTTACGTATTCCGGCCAGACCCTGTCTTATCGCGAATTAGATGAGGAAGCGAAC
CGCCTTGGACGCCGGCTGCAAAAACAAGGTGCGGGCAAAGGCTCTGTTGTAGCGCTGTACACGAAGCGCTCACTT
GAACTGGTGATCGGCATTCTCGGTGTATTAAAGGCGGGAGCAGCCTATCTGCCGGTTGATCCGAAGCTGCCAGAG
GACCGAATCTCGTATATGCTGGCTGACAGTGCGGCAGCCTGTCTTCTGACGCATCAGGAGATGAAAGAACAAGCG
GCTGAGCTGCCGTATACAGGCACAACGCTCTTCATTGATGATCAAACACGGTTTGAAGAACAGGCAAGCGATCCT
GCAACCGCGATTGATCCTAATGATCCGGCATATATCATGTACACGTCCGGCACAACCGGAAAGCCAAAAGGCACT
ATGACTACTCATGCCAATATTCAAGGTTTGGTCAAGCATGTAGACTACATGGCATTTTCTGATCAGGATACGTTC
TTGTCGGTTTCGAATTACGCCTTTGACGCATTTACCTTTGATTTCTATGCTTCTATGCTGAATGCGGCACGGCTC
ATTATCGCAGACGAACATACGCTGCTTGATACAGAACGGCTCACAGATCTGATTCTGCAAGAGAATGTCAATGTC
ATGTTTGCGACAACCGCACTATTTAATCTTCTCACAGATGCGGGAGAGGATTGGATGAAGGGGCTTCGCTGTATA
TTATTCGGCGGAGAGCGCGCCTCAGTGCCTCATGTCAGAAAAGCGCTGCGGGTCATGGGGCCGGGCAAGCTGATT
AACTGCTACGGGCCGACTGAGGGAACAGTGTTTGCGACAGCTCACGTCGTGCATGATCTGCCGGATTCCATCTCC
TCATTGCCGATTGGAAAGCCGATCAGCAATGCCCAAGTTTATATTCTGAACGAGCAAAGCCAGCTCCAGCCATTC
GGGGCGATCGGTGAACTGTGCATCAGCGGAATGGGCGTGTCAAAAGGATATGTAAATCGTGCTGACCTCACGAAG
GAAAAGTTTATTAAGAACCCGTTCAAGCCGGGAGAAACGCTTTACCGCACAGGGGATTTAGCGCGCTGGCTGCCG
GATGGAACGATTGAATACGCCGGCCGTATTGACGACCAGGTCAAAATACGCGGACACCGGATTGAGCTTGAAGAA
ATCGAAAAGCAGCTGCAGGAATACCCAGGTGTGAAAGATGCGGTCGTTGTGGCGGACCGCCATGAGTCTGGCGAT
GCATCAATCAATGCCTACCTTGTGCAACGAACGCAGCTTTCAGCTGAGGACGTGAAGGCGCACCTGAAAAAACAG
CTTCCTGCTTATATGGTGCCGCAAACCTTTACCTTCTTGGATGAGCTTCCTTTAACGACGAACGGGAAAGTCAAT
AAACGGCTGCTCCCAAAACCTGATCAGGATCAGCTGGCGGAAGAATGGATTGGACCGCGGAACGAGATGGAAGAA
ACAATCGCACAAATATGGTCTGAGGTTCTCGGCAGAAAGCAAATTGGCATTCATGACGATTTCTTTGCGCTCGGA
GGGCATTCCTTGAAGGCCATGACCGCCGCGTCCCGCATCAAGAAAGAGCTCGGGATTGATCTTCCAGTAAAGCTT
TTGTTTGAAGCGCCGACGATCGCCGGCATTTCAGCGTATGTGAAAAACGGGGGTCCTGATGGCTTGCAGGATGTA
CTCGAGGGTGGAGGAGGTAGTGAACAAAAGCTTATTTCTGAAGAGGACTTGGGAGGTGGTGGATCAGGTGGTGGA
GGAAGCCAGGAACTGACAACTATATGCGAGCAAATCCCCTCACCAACTTTAGAATCGACGCCGTACTCTTTGTCA
ACGACTACTATTTTGGCCAACGGGAAGGCAATGCAAGGAGTTTTTGAATATTACAAATCAGTAACGTTTGTCAGT
AATTGCGGTTCTCACCCCTCAACAACTAGCAAAGGCAGCCCCATAAACACACAGTATGTTTTTTAATAGGTCGAG
ATCTGATAACAACAGTGTAGATGTAACAAAATCGACTTTGTTCCCACTGTACTTTTAGCTCGTACAAAATACAAT
ATACTTTTCATTTCTCCGTAAACAACATGTTTTCCCATGTAATATCCTTTTCTATTTTTCGTTCCGTTACCAACT
TTACACATACTTTATATAGCTATTCACTTCTATACACTAAAAACTAAGACAATTTTAATTTTGCTGCCTGCCAT
ATTTCAATTTGTTATAAATTCCTATAATTTATCCTATTAGTAGCTAAAAAAAGATGAATGTGAATCGAATCCTAA
GAGAATTGAGCTCCAATTCGCCCTATAGTGAGTCGTATTACAATTCACTGGCCGTCGTTTTACAACGTCGTGACT
GGGAAAACCCTGGCGTTACCCAACTTAATCGCCTTGCAGCACATCCCCCCTTCGCCAGCTGGCGTAATAGCGAAG
AGGCCCGCACCGATCGCCCTTCCCAACAGTTGCGCAGCCTGAATGGCGAATGGCGCGACGCGCCCTGTAGCGGCG
CATTAAGCGCGGCGGGTGTGGTGGTTACGCGCAGCGTGACCGCTACACTTGCCAGCGCCCTAGCGCCCGCTCCTT
TCGCTTTCTTCCCTTCCTTTCTCGCCACGTTCGCCGGCTTTCCCCGTCAAGCTCTAAATCGGGGGCTCCCTTTAG
GGTTCCGATTTAGTGCTTTACGGCACCTCGACCCCAAAAAACTTGATTAGGGTGATGGTTCACGTAGTGGGCCAT
CGCCCTGATAGACGGTTTTTCGCCCTTTGACGTTGGAGTCCACGTTCTTTAATAGTGGACTCTTGTTCCAAACTG
GAACAACACTCAACCCTATCTCGGTCTATTCTTTTGATTTATAAGGGATTTTGCCGATTTCGGCCTATTGGTTAA
AAAATGAGCTGATTTAACAAAAATTTAACGCGAATTTTAACAAAATATTAACGTTTACAATTTCCTGATGCGGTA
TTTTCTCCTTACGCATCTGTGCGGTATTTCACACCGCAGGCAAGTGCACAAACAATACTTAAATAAATACTACTC
AGTAATAACCTATTTCTTAGCATTTTTGACGAAATTTGCTATTTTGTTAGAGTCTTTTACACCATTTGTCTCCAC
ACCTCCGCTTACATCAACACCAATAACGCCATTTAATCTAAGCGCATCACCAACATTTTCTGGCGTCAGTCCACC
AGCTAACATAAAATGTAAGCTTTCGGGGCTCTCTTGCCTTCCAACCCAGTCAGAAATCGAGTTCCAATCCAAAAG
TTCACCTGTCCCACCTGCTTCTGAATCAAACAAGGGAATAAACGAATGAGGTTTCTGTGAAGCTGCACTGAGTAG

```
TATGTTGCAGTCTTTTGGAAATACGAGTCTTTTAATAACTGGCAAACCGAGGAACTCTTGGTATTCTTGCCACGA
CTCATCTCCATGCAGTTGGACGATATCAATGCCGTAATCATTGACCAGAGCCAAAACATCCTCCTTAGGTTGATT
ACGAAACACGCCAACCAAGTATTTCGGAGTGCCTGAACTATTTTTATATGCTTTTACAAGACTTGAAATTTTCCT
TGCAATAACCGGGTCAATTGTTCTCTTTCTATTGGGCACACATATAATACCCAGCAAGTCAGCATCGGAATCTAG
AGCACATTCTGCGGCCTCTGTGCTCTGCAAGCCGCAAACTTTCACCAATGGACCAGAACTACCTGTGAAATTAAT
AACAGACATACTCCAAGCTGCCTTTGTGTGCTTAATCACGTATACTCACGTGCTCAATAGTCACCAATGCCCTCC
CTCTTGGCCCTCTCCTTTTCTTTTTTCGACCGAATTAATTCTTAATCGGCAAAAAAAGAAAAGCTCCGGATCAAG
ATTGTACGTAAGGTGACAAGCTATTTTTCAATAAAGAATATCTTCCACTACTGCCATCTGGCGTCATAACTGCAA
AGTACACATATATTACGATGCTGTCTATTAAATGCTTCCTATATTATATATATAGTAATGTCGTTTATGGTGCAC
TCTCAGTACAATCTGCTCTGATGCCGCATAGTTAAGCCAGCCCCGACACCCGCCAACACCCGCTGACGCGCCCTG
ACGGGCTTGTCTGCTCCCGGCATCCGCTTACAGACAAGCTGTGACCGTCTCCGGGAGCTGCATGTGTCAGAGGTT
TTCACCGTCATCACCGAAACGCGCGAGACGAAAGGGCCTCGTGATACGCCTATTTTTATAGGTTAATGTCATGAT
AATAATGGTTTCTTAGGACGGATCGCTTGCCTGTAACTTACACGCGCCTCGTATCTTTTAATGATGGAATAATTT
GGGAATTTACTCTGTGTTTATTTATTTTTATGTTTTGTATTTGGATTTTAGAAAGTAAATAAAGAAGGTAGAAGA
GTTACGGAATGAAGAAAAAAAAAATAAACAAAGGTTTAAAAAATTTCAACAAAAAGCGTACTTTACATATATATTT
ATTAGACAAGAAAAGCAGATTAAATAGATATACATTCGATTAACGATAAGTAAAATGTAAAATCACAGGATTTTC
GTGTGTGGTCTTCTACACAGACAAGATGAAACAATTCGGCATTAATACCTGAGAGCAGGAAGAGCAAGATAAAAG
GTAGTATTTGTTGGCGATCCCCCTAGAGTCTTTTACATCTTCGGAAAACAAAAACTATTTTTTCTTTAA
```

- Signal peptide and linker (yellow)
- Docking, C, A and T domains of SrfA-C (cyan)
- c-myc tag (magenta)
- aga2p (green)
- mutations introduced to prevent post-translational glycosylation (red)

High-resolution mass spectrometry (ESI-MS)

apo-TycA*

Calculated MW: 107,012.0 Da

Found MW: 107,010.0 Da

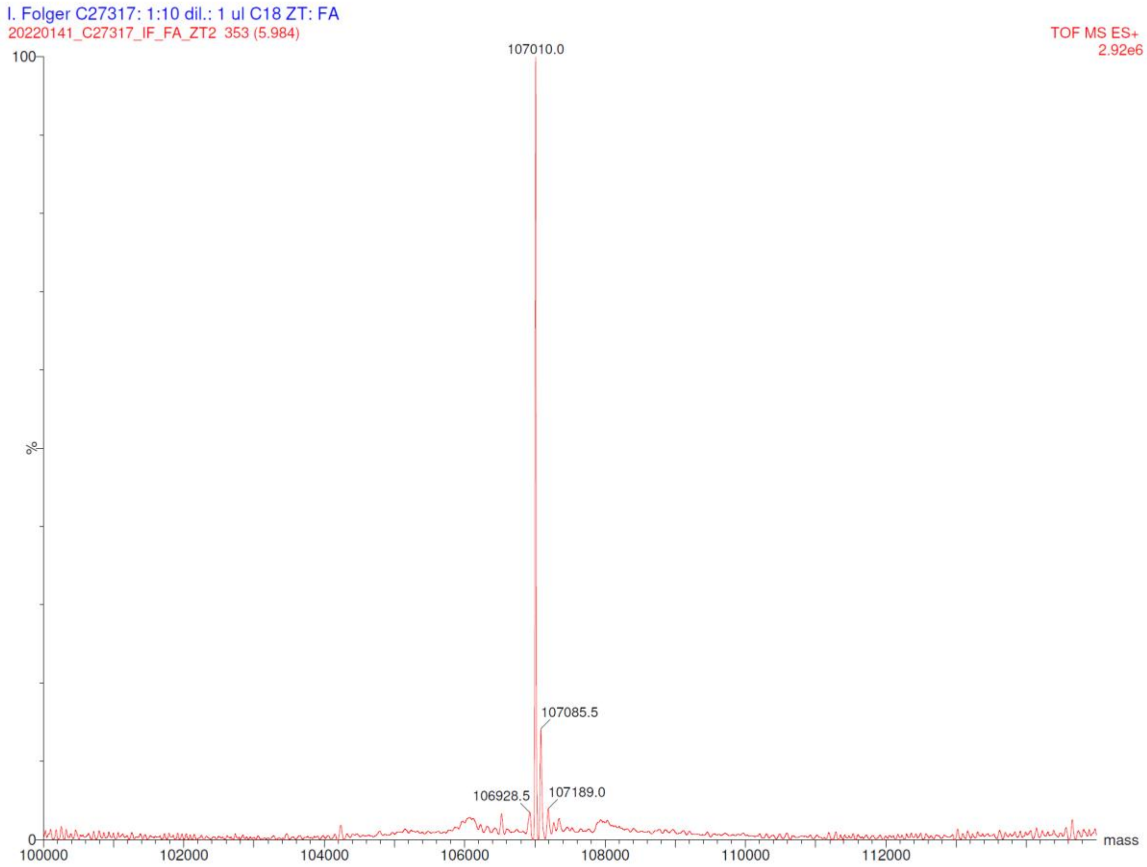

TycA* primed with 10-undecynoyl-ppant

Calculated MW: 107,517.2 Da

Found MW: 107,515.0 Da

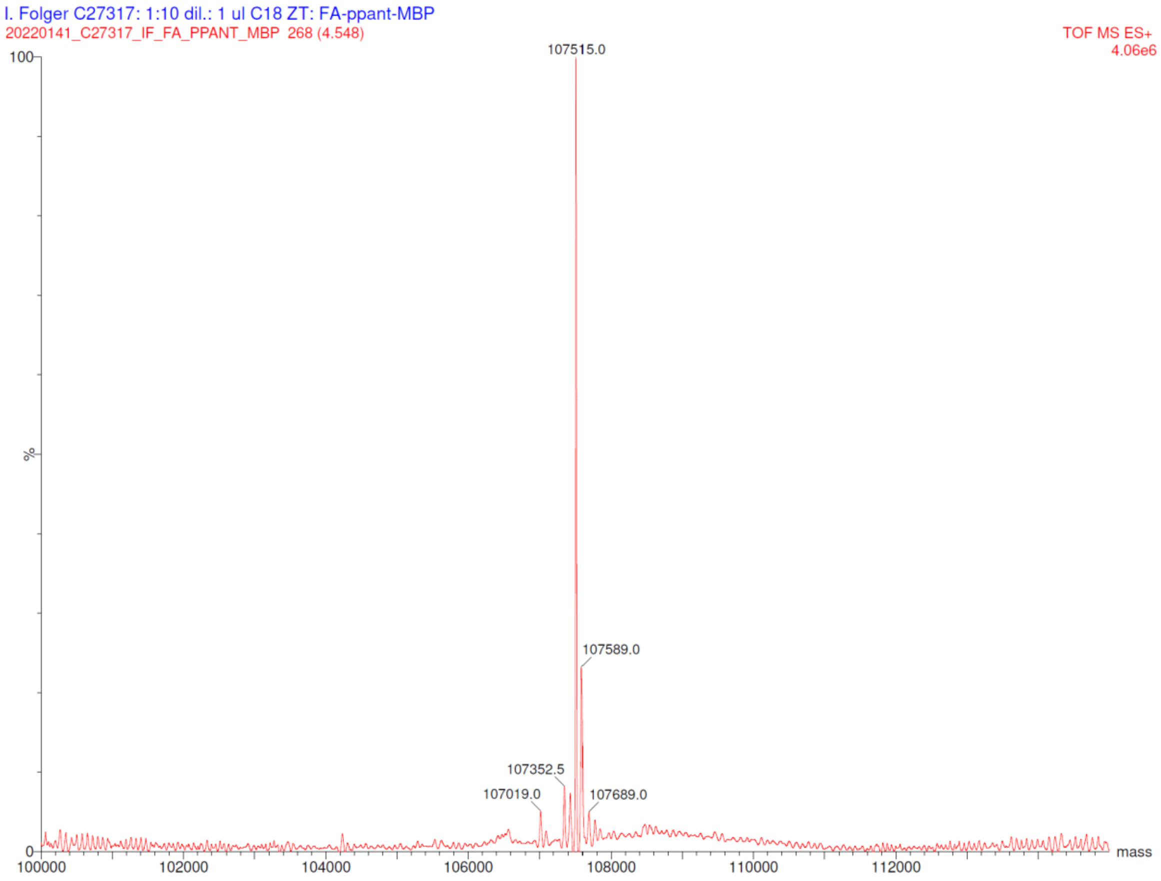

TycA* primed with 10-undecynoyl-ppant after incubation for 1 h at RT

The found mass corresponds within error to apo-TycA*, indicating complete hydrolysis of the thioester.

Found MW: 107,006.0 Da

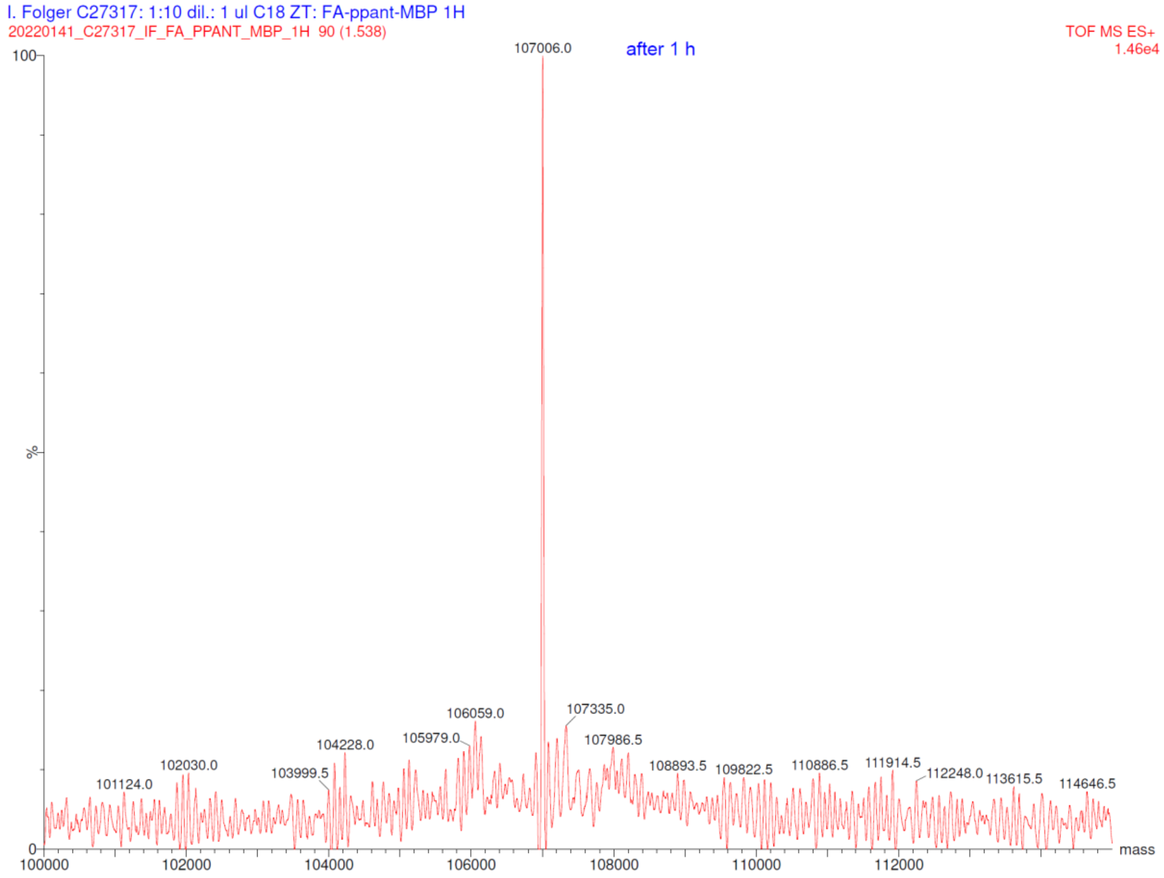

holo-SrfA-C wildtype

N-terminal Met cleaved off by *E. coli* following translation and ppant post-translationally loaded onto Ser1003 by Sfp.

Calculated MW: 145,142.5 Da

Found MW: 145,141.6 Da

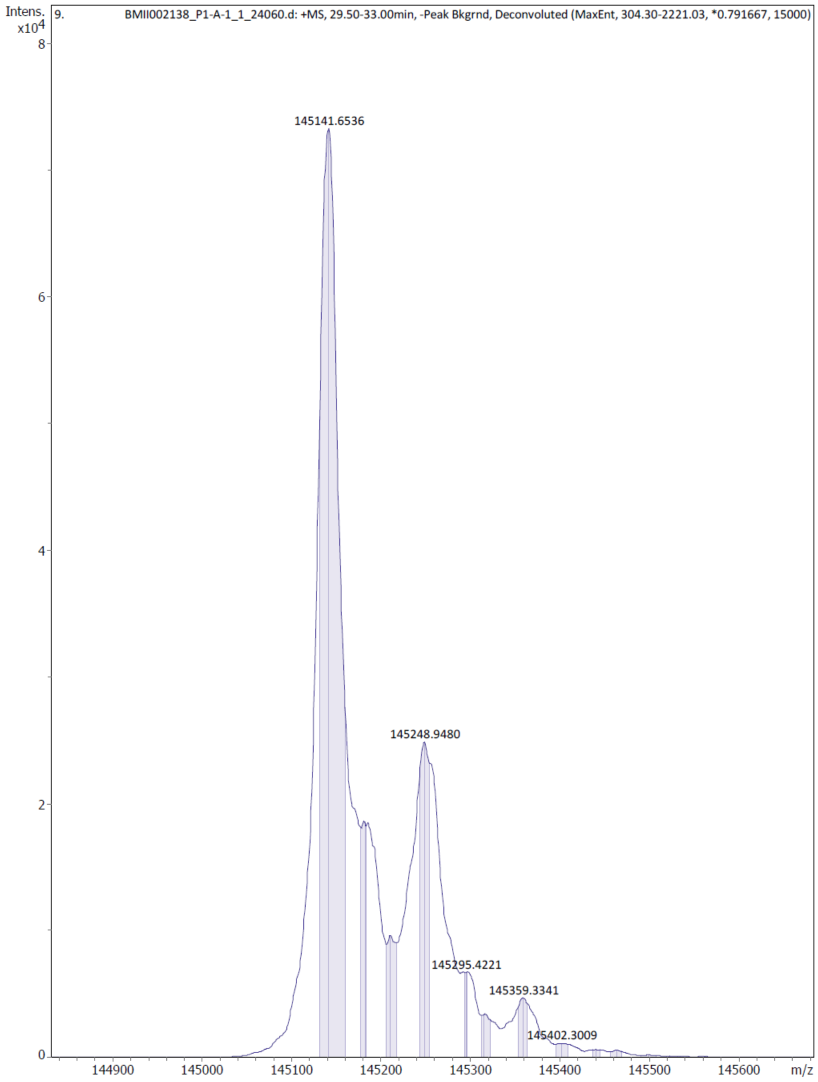

<u>holo-SrfA-C_{FA}</u>

N-terminal Met cleaved off by *E. coli* following translation and ppant post-translationally loaded onto Ser1003 by Sfp.

Calculated MW: 144,959.4 Da

Found MW: 144,958.7 Da

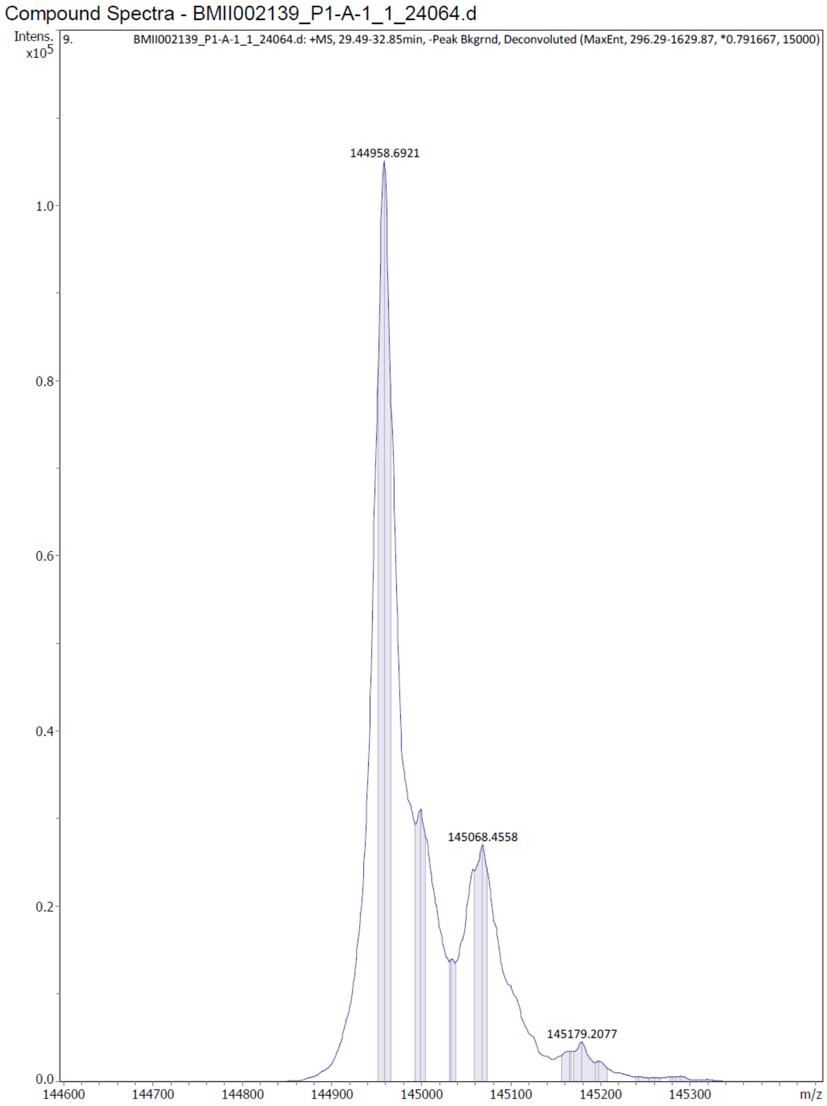

NMR spectroscopy

10-undecynoyl-pant: ^{1}H-NMR (DMSO-*d*6)

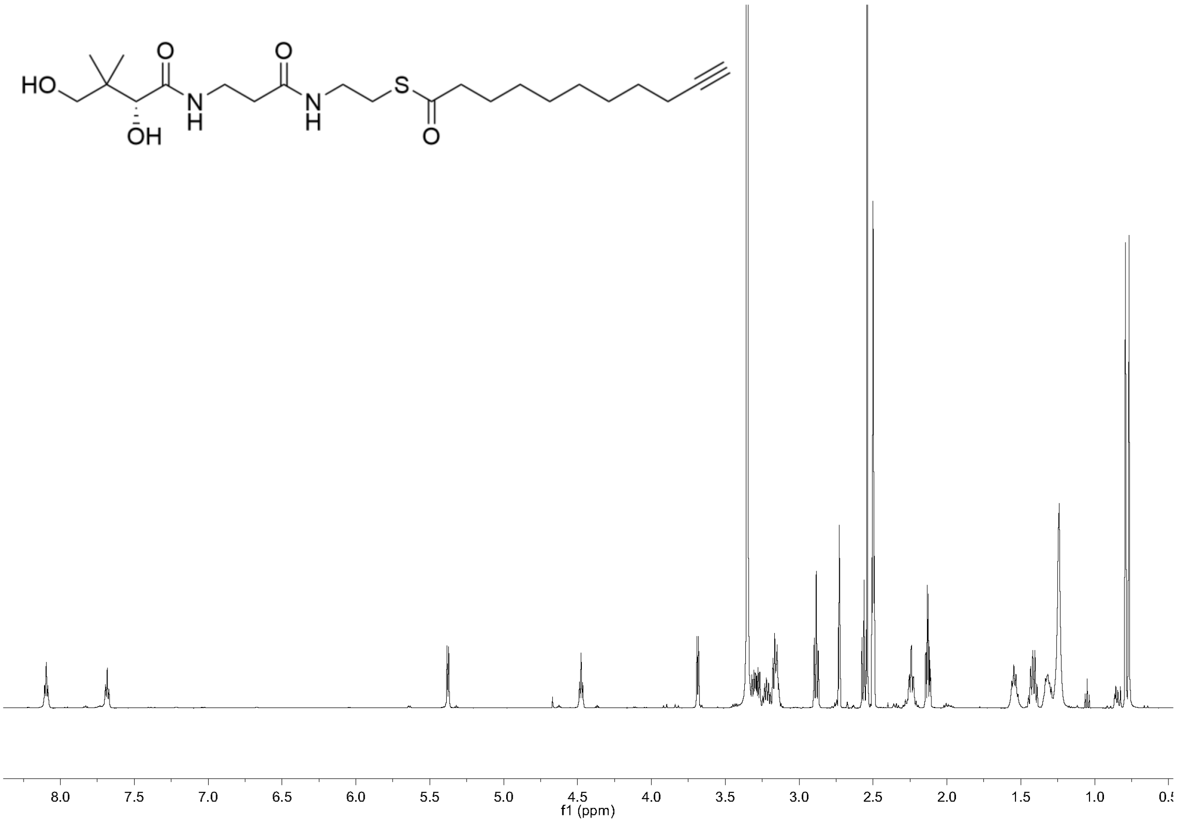

10-undecynoyl-pant: ^{13}C-NMR (DMSO-d_6)

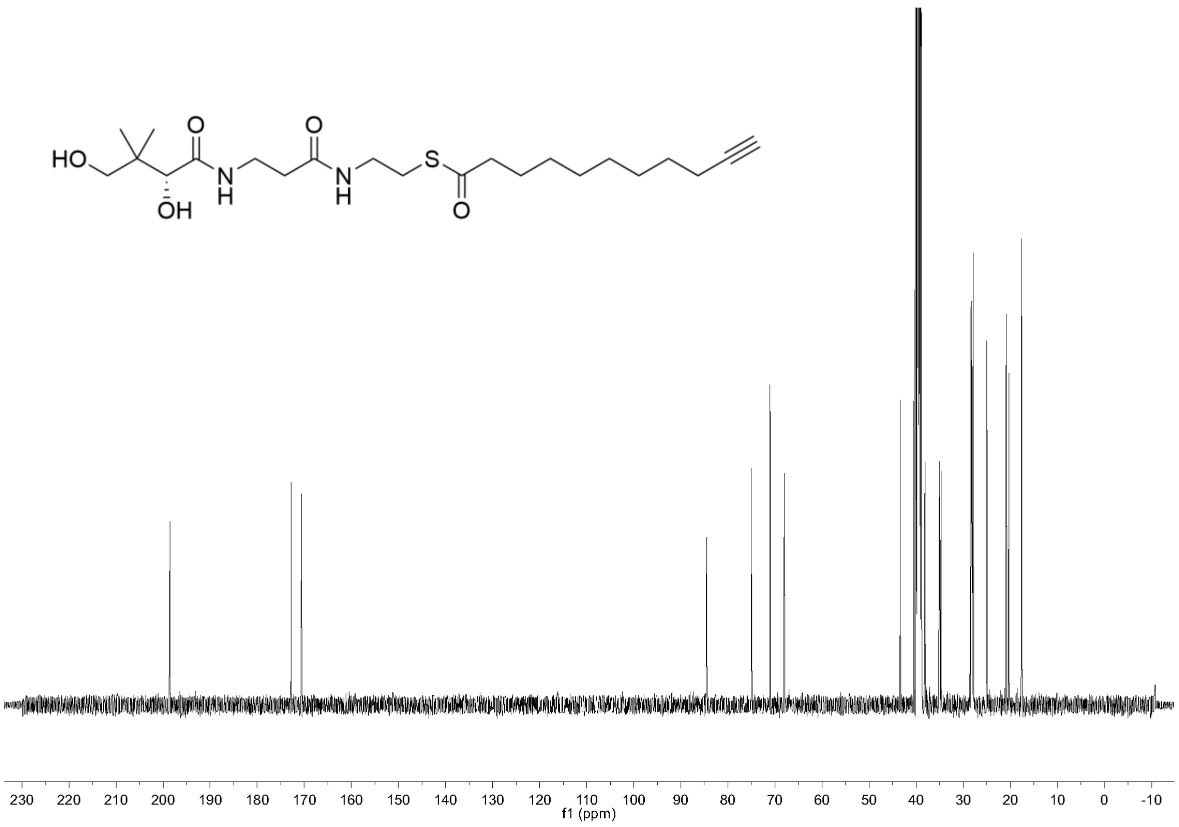

10-undecynoyl-pant: HSQC (DMSO-d_6)

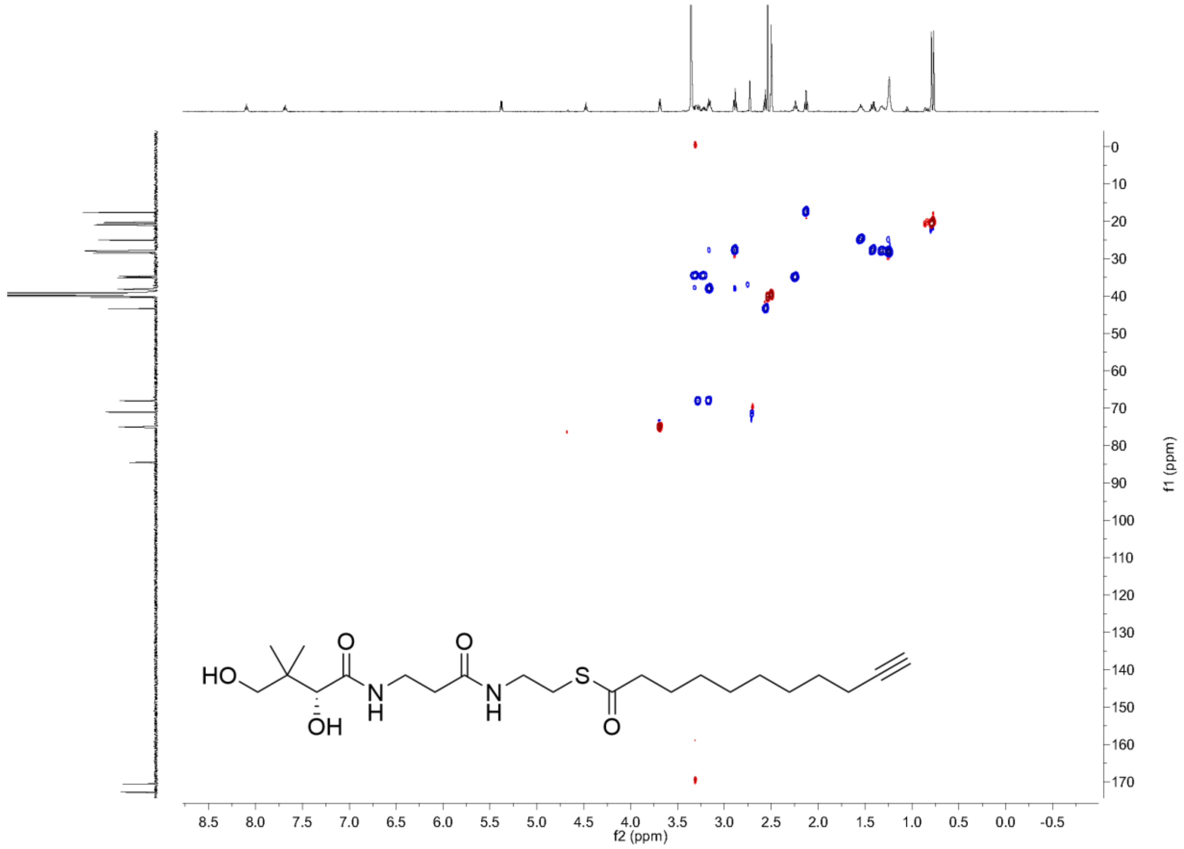

10-undecynoyl-pant: HMBC (DMSO-d_6)

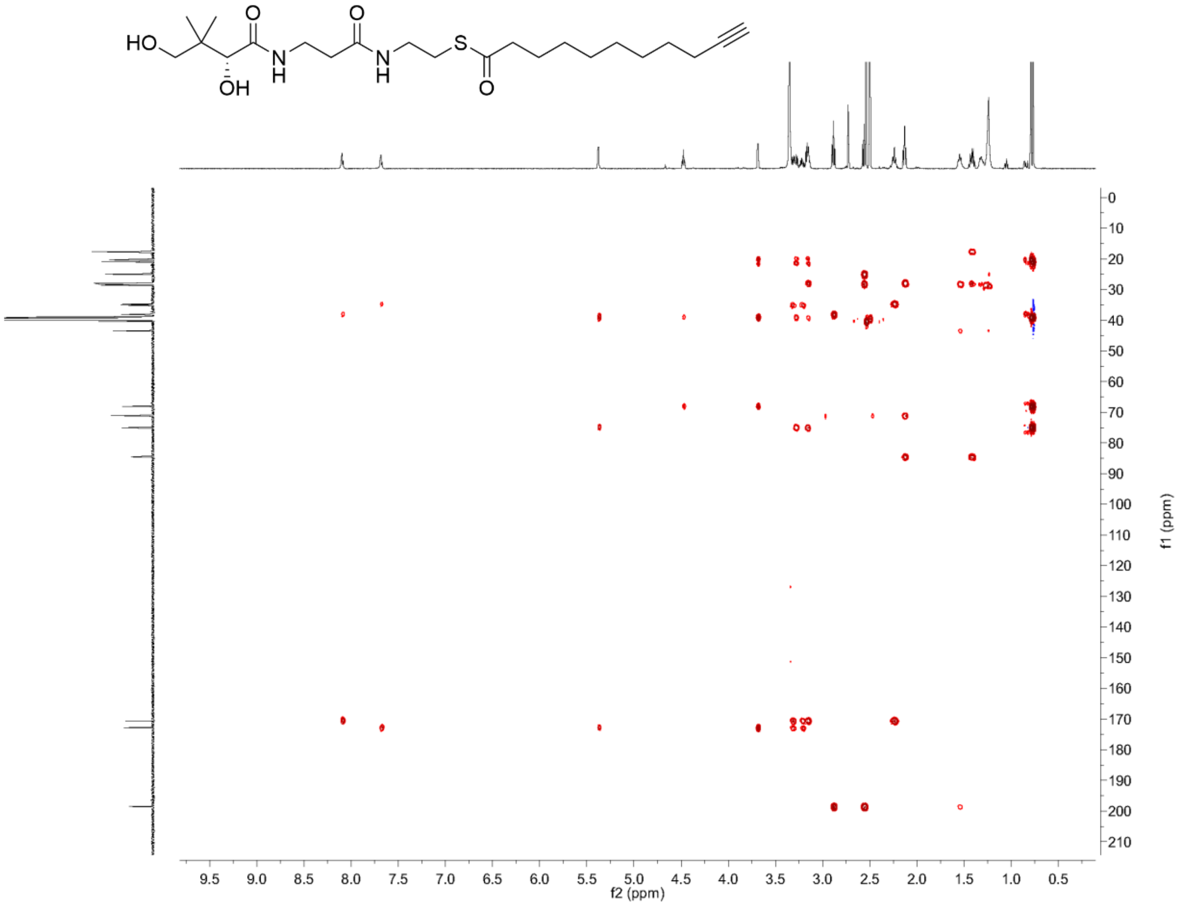

PMB-amino-pantetheine: ^{1}H-NMR (MeOD)

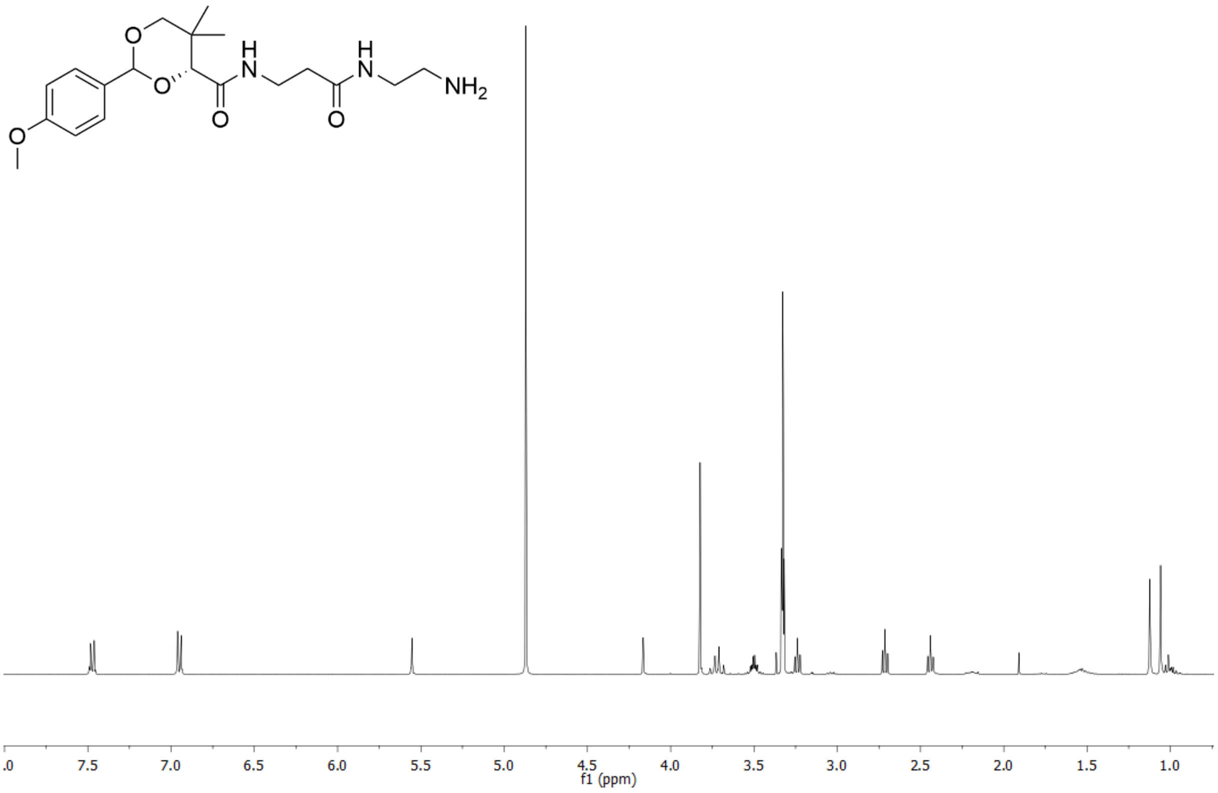

10-undecynoyl-pant-amide: ^{1}H-NMR (DMSO-*d*6)

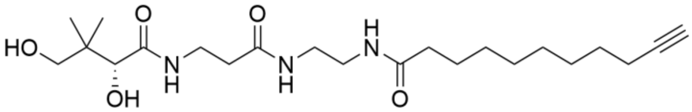

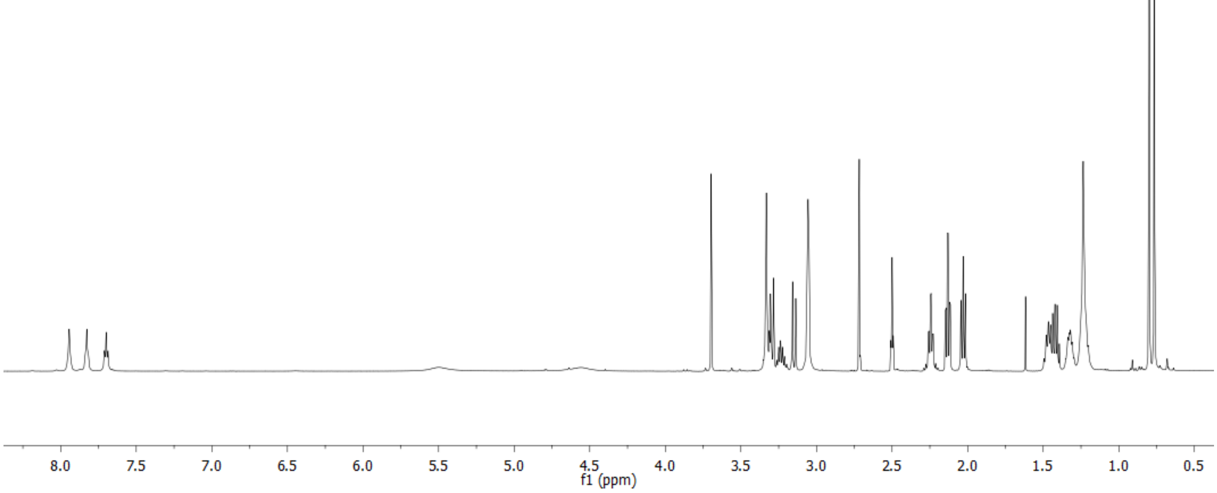

10-undecynoyl-pant-amide: ^{13}C-NMR (DMSO-*d*6)

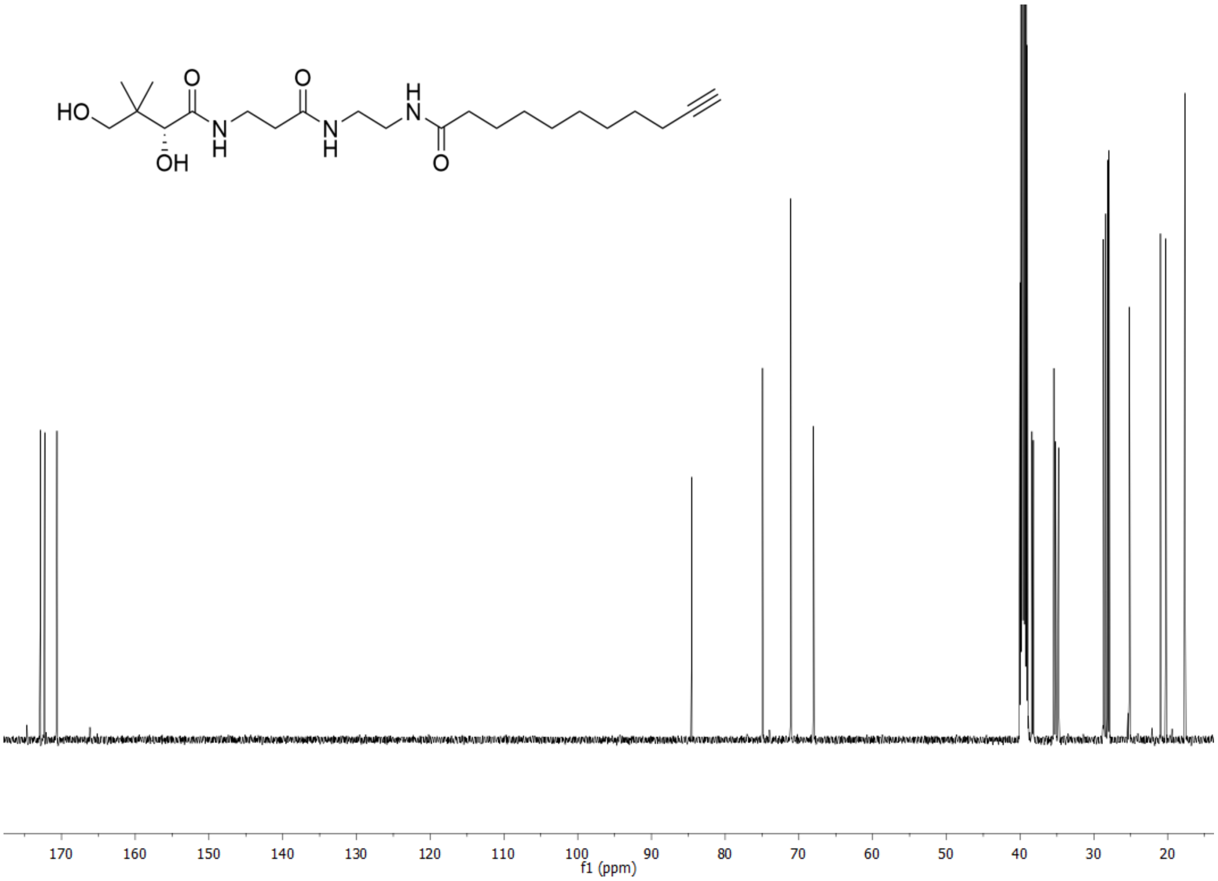

10-undecynoyl-pant-amide: HSQC (DMSO-*d*6)

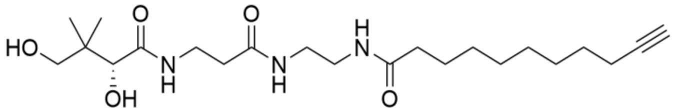

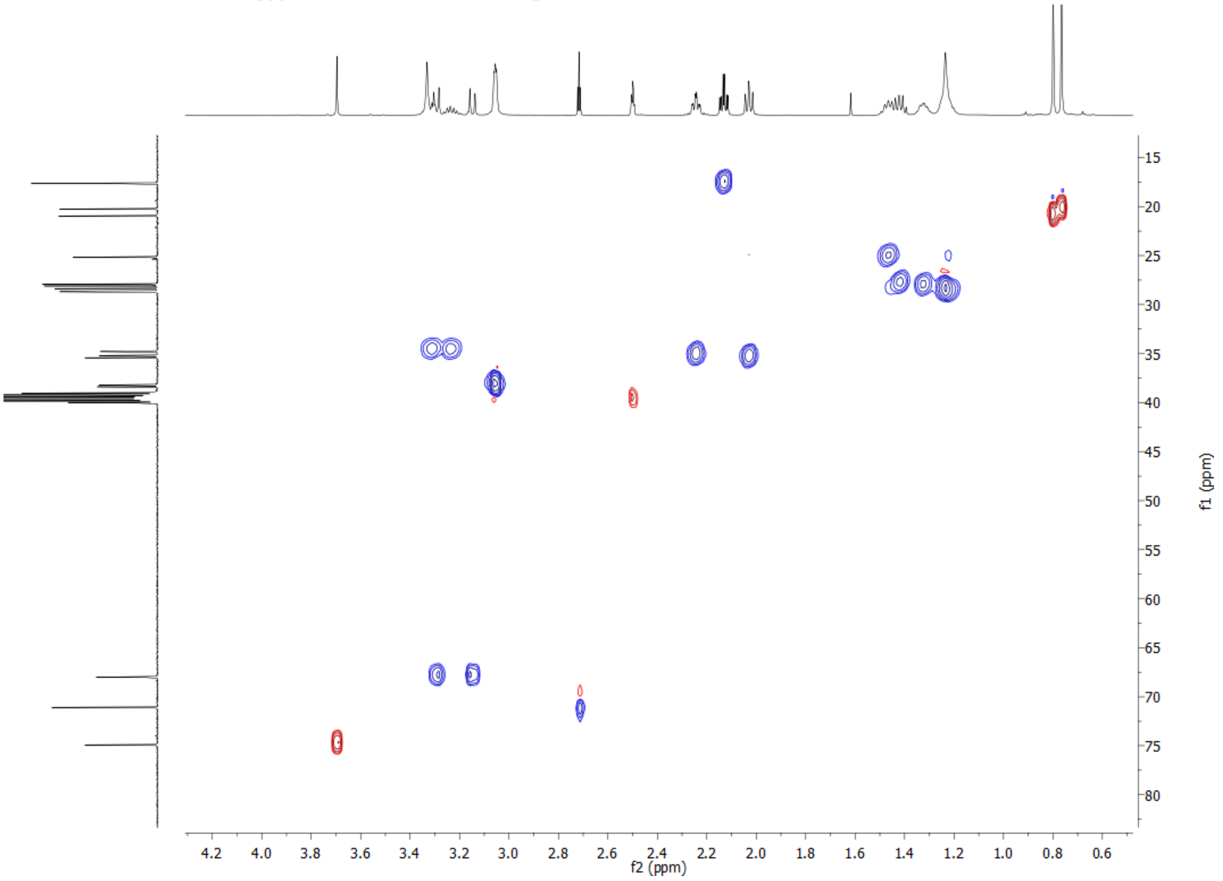

10-undecynoyl-pant-amide: HMBC (DMSO-*d6*)

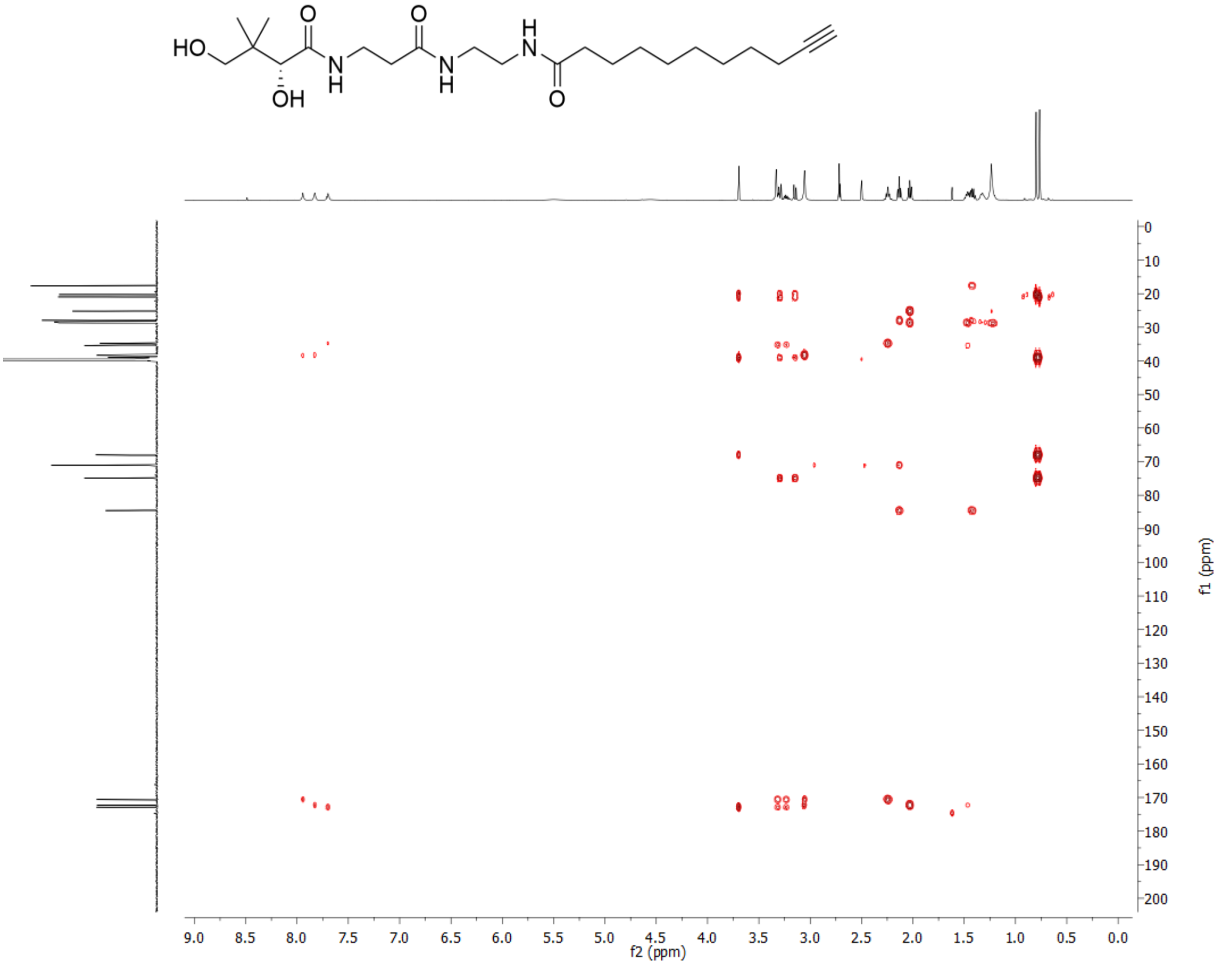

10-undecynoyl-L-Leu: ^{1}H-NMR (MeOD)

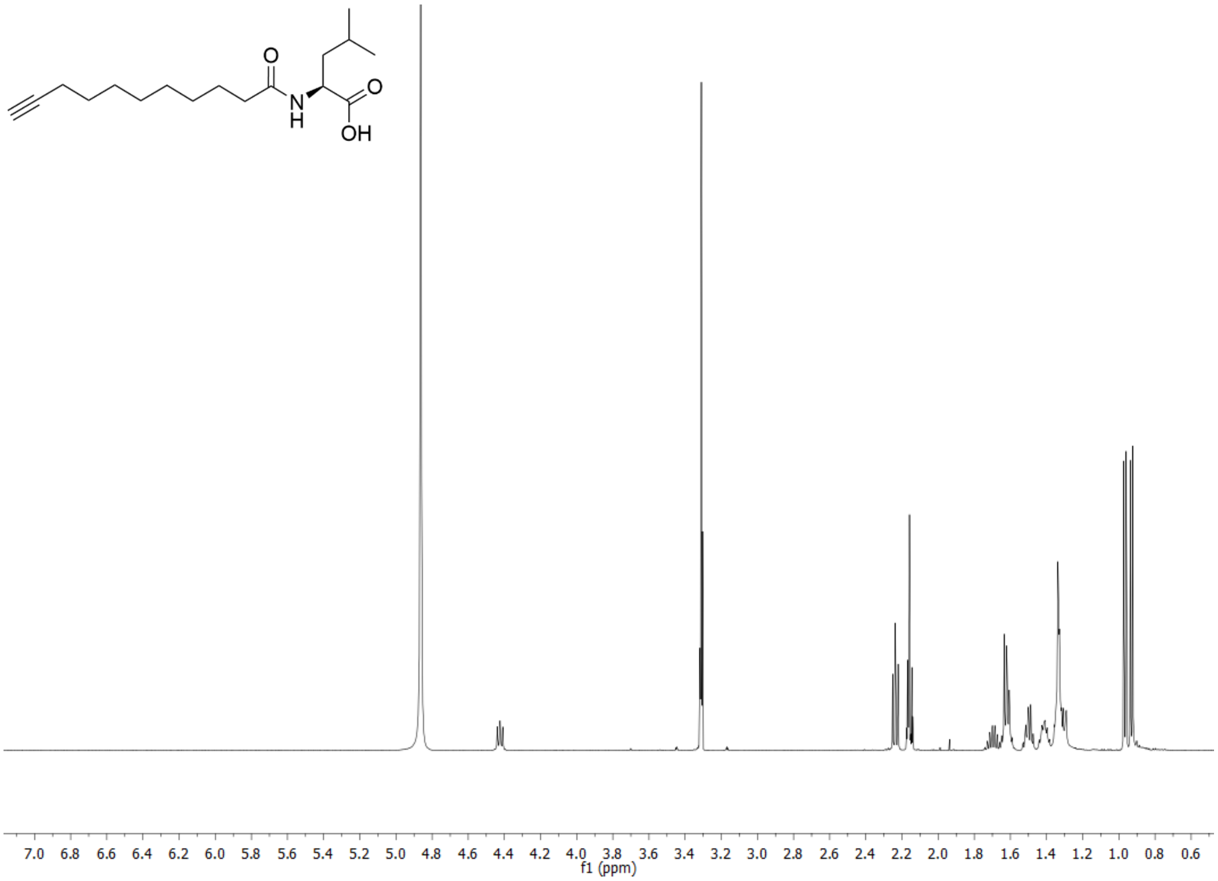

10-undecynoyl-L-Leu: ^{13}C-NMR (MeOD)

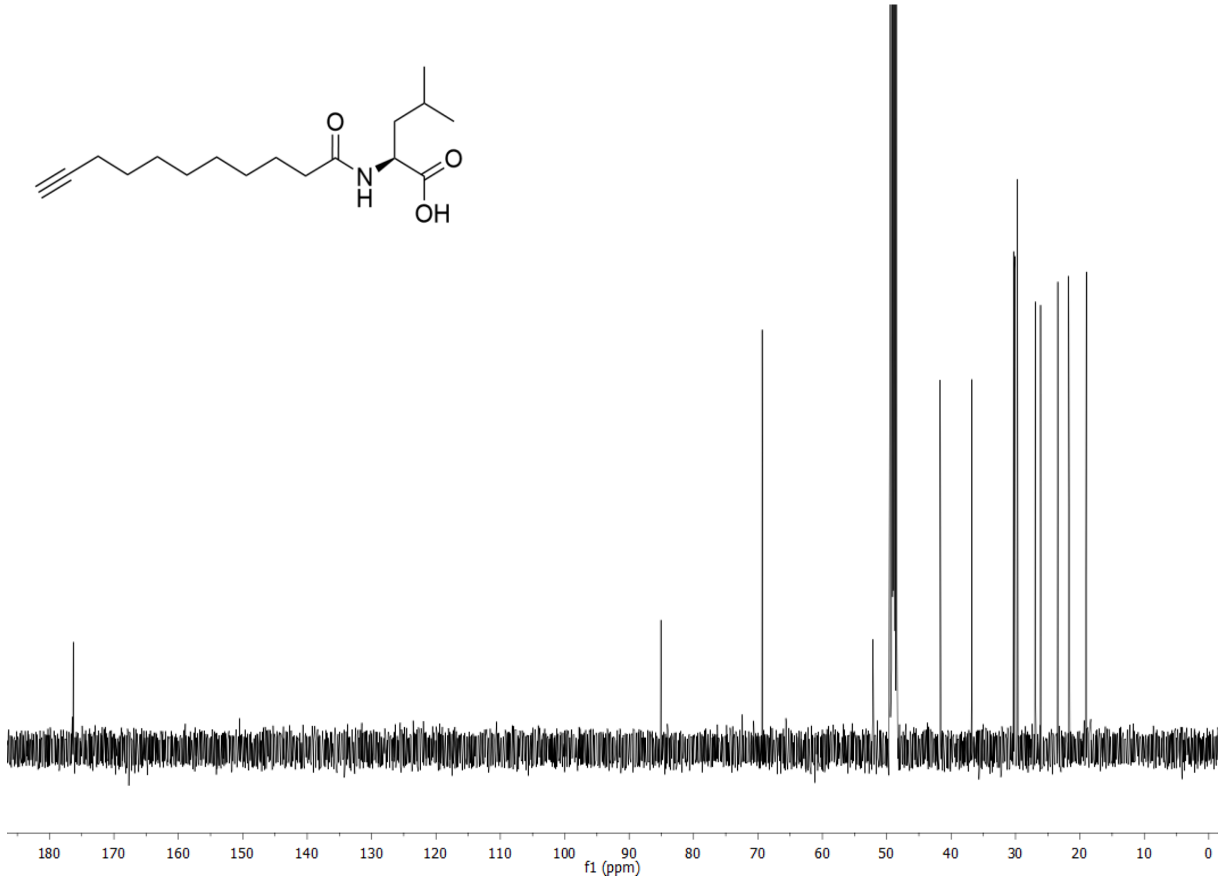

10-undecynoyl-L-Leu: HSQC (MeOD)

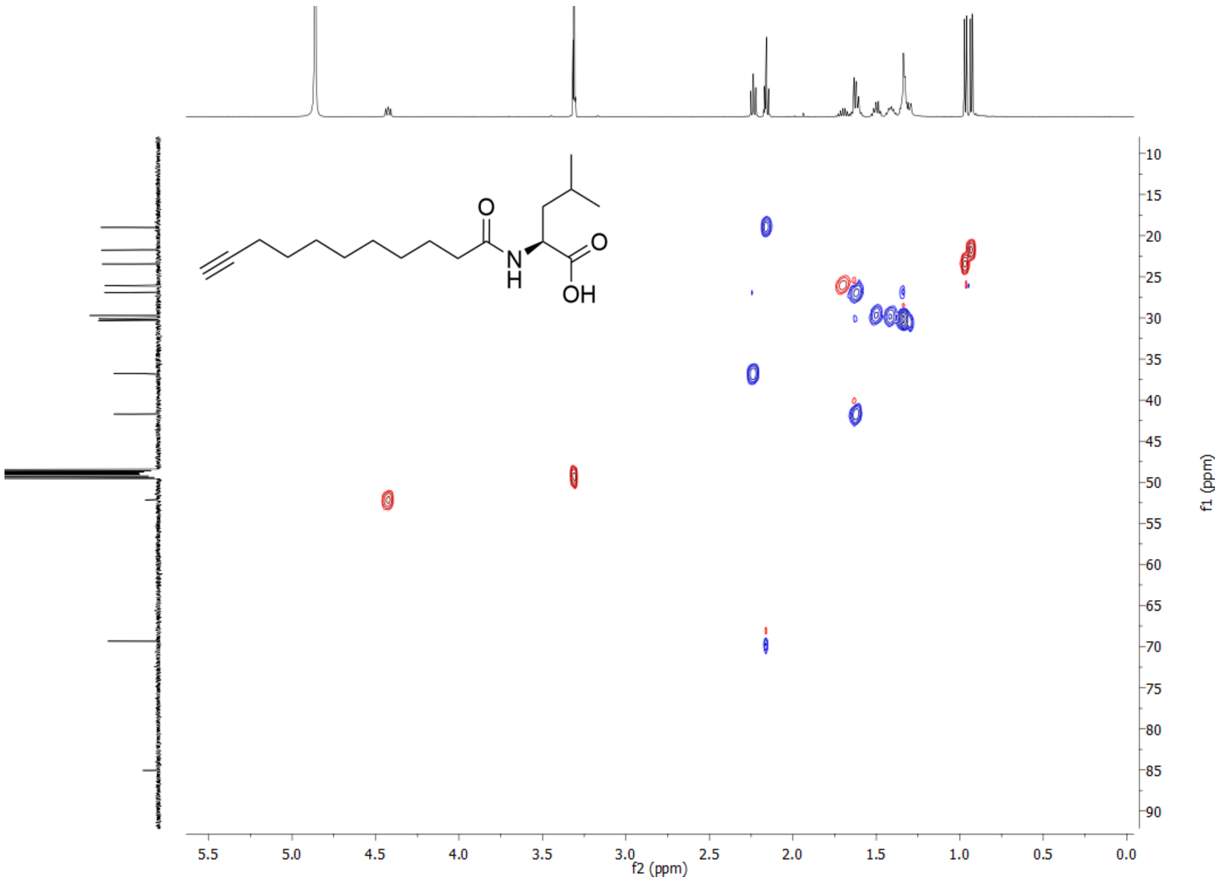

10-undecynoyl-L-Leu: HMBC (MeOD)

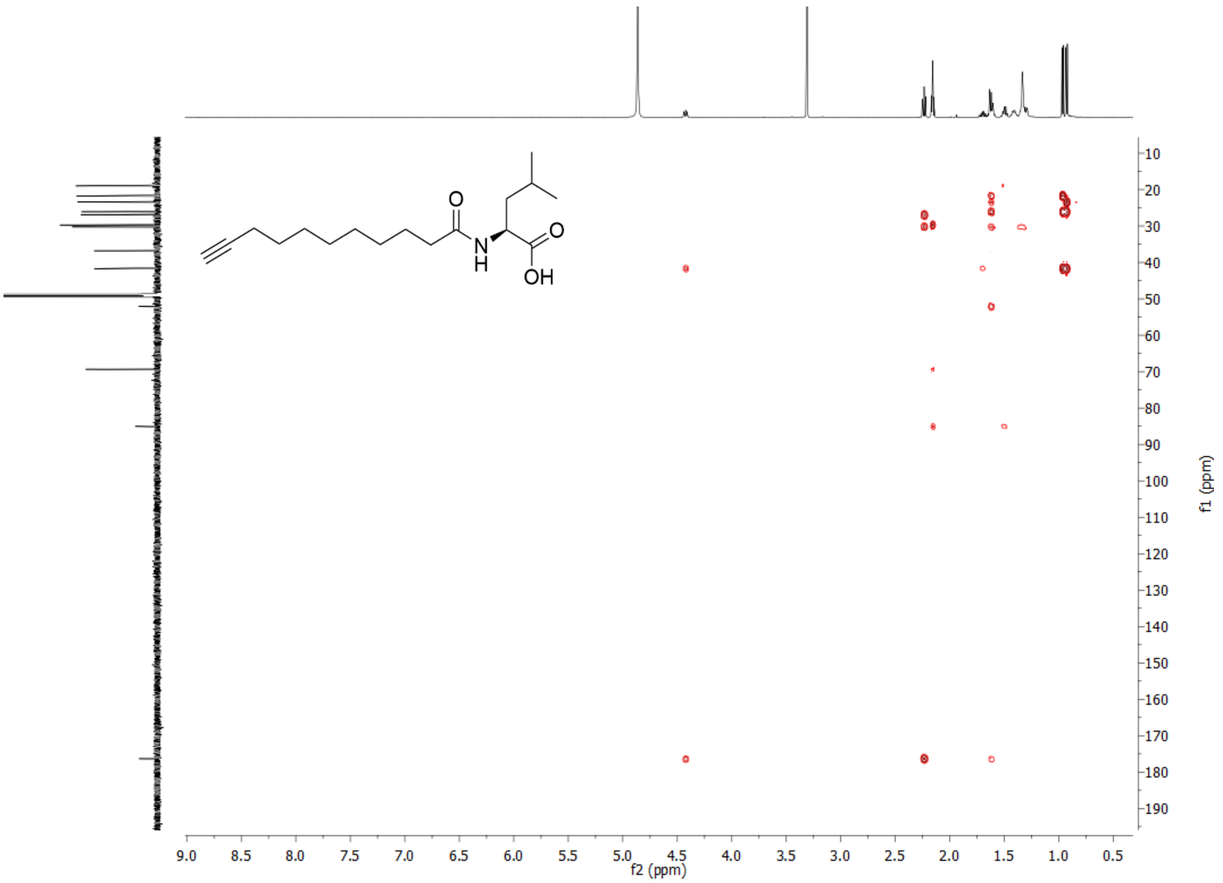

Boc-*O*-propargyl-D-Tyr-L-Leu: ^{1}H-NMR (MeOD)

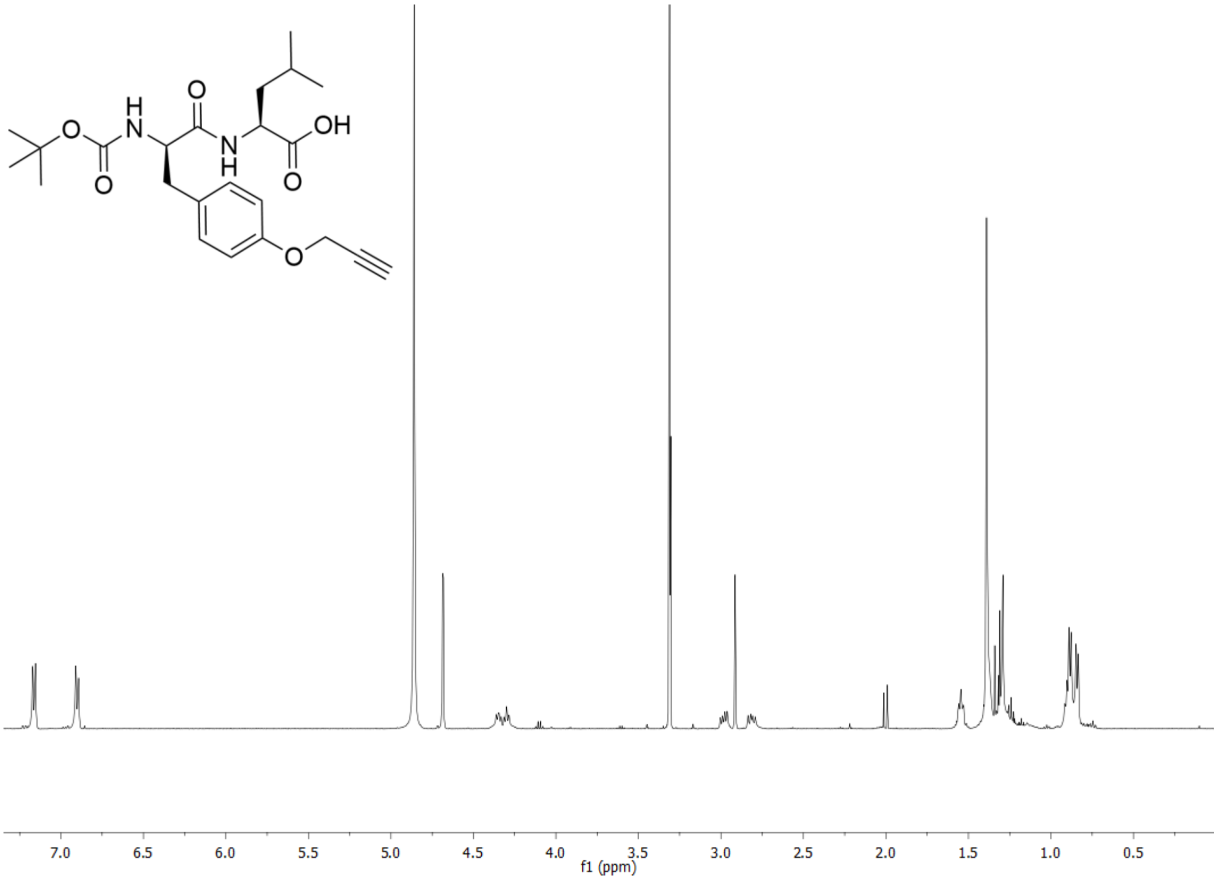

Boc-*O*-propargyl-D-Tyr-L-Leu: ^{13}C-NMR (MeOD)

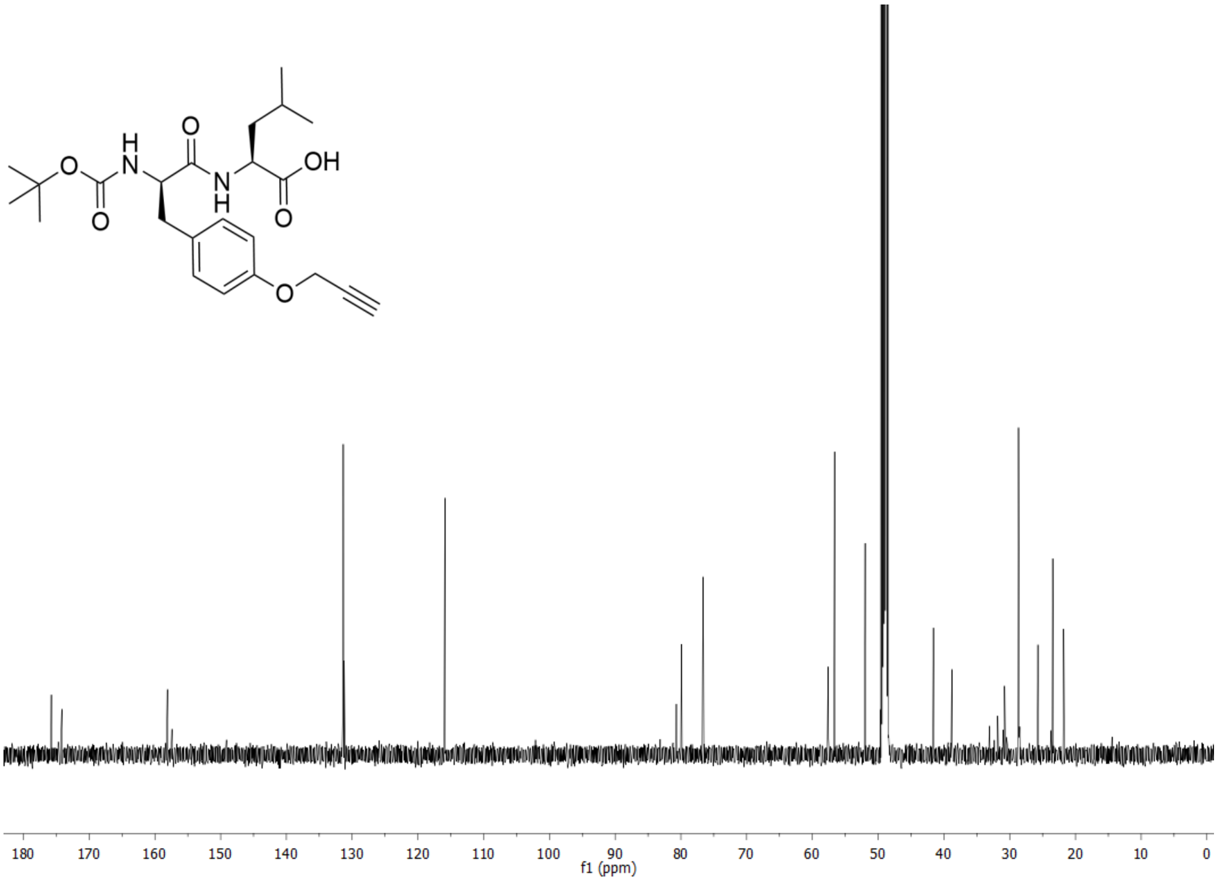

Boc-*O*-propargyl-D-Tyr-L-Leu: HSQC (MeOD)

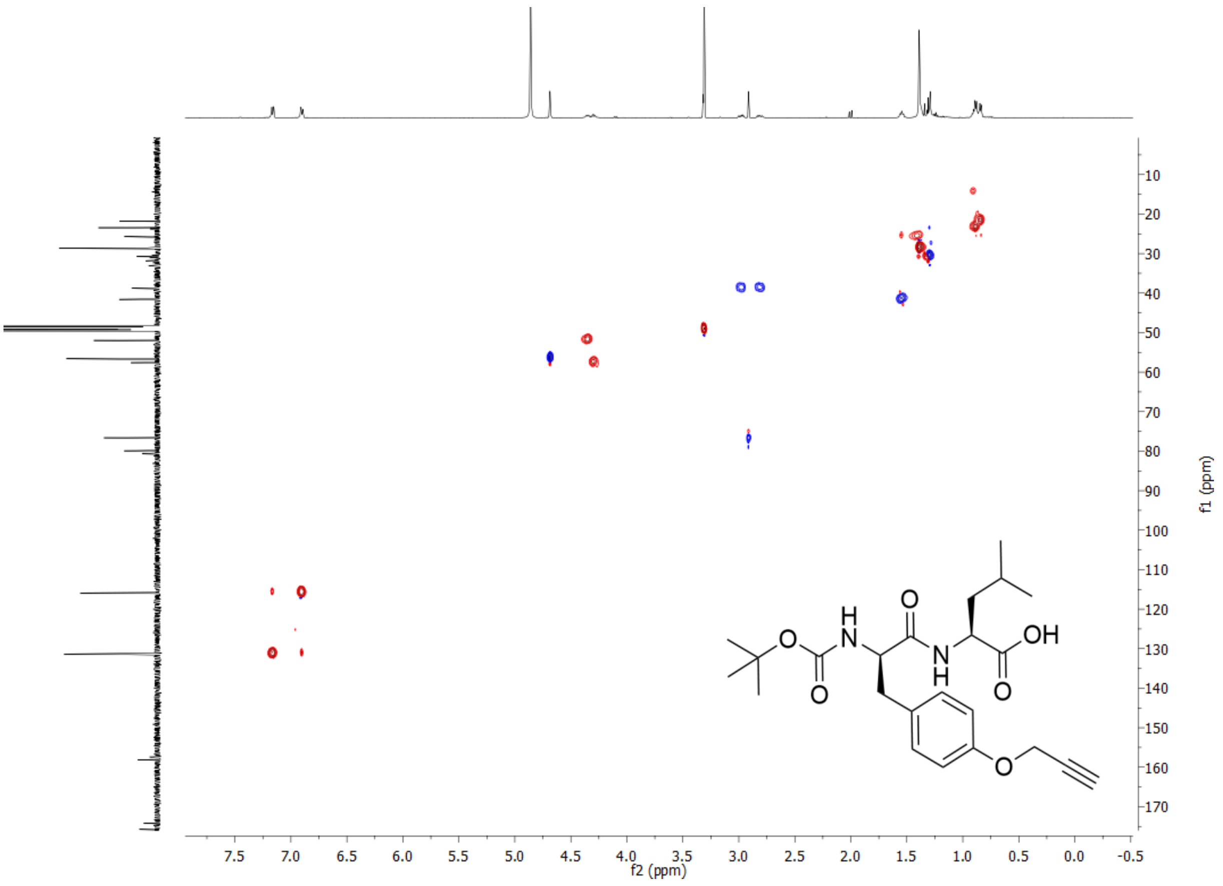

***O*-propargyl-D-Tyr-L-Leu-pant-amide:** ^{1}H-NMR (MeOD)

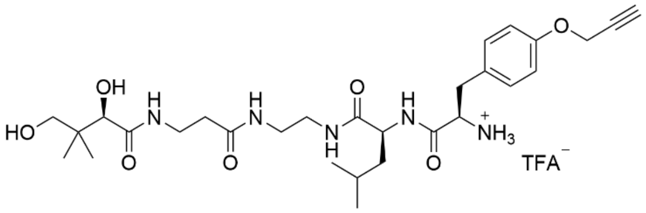

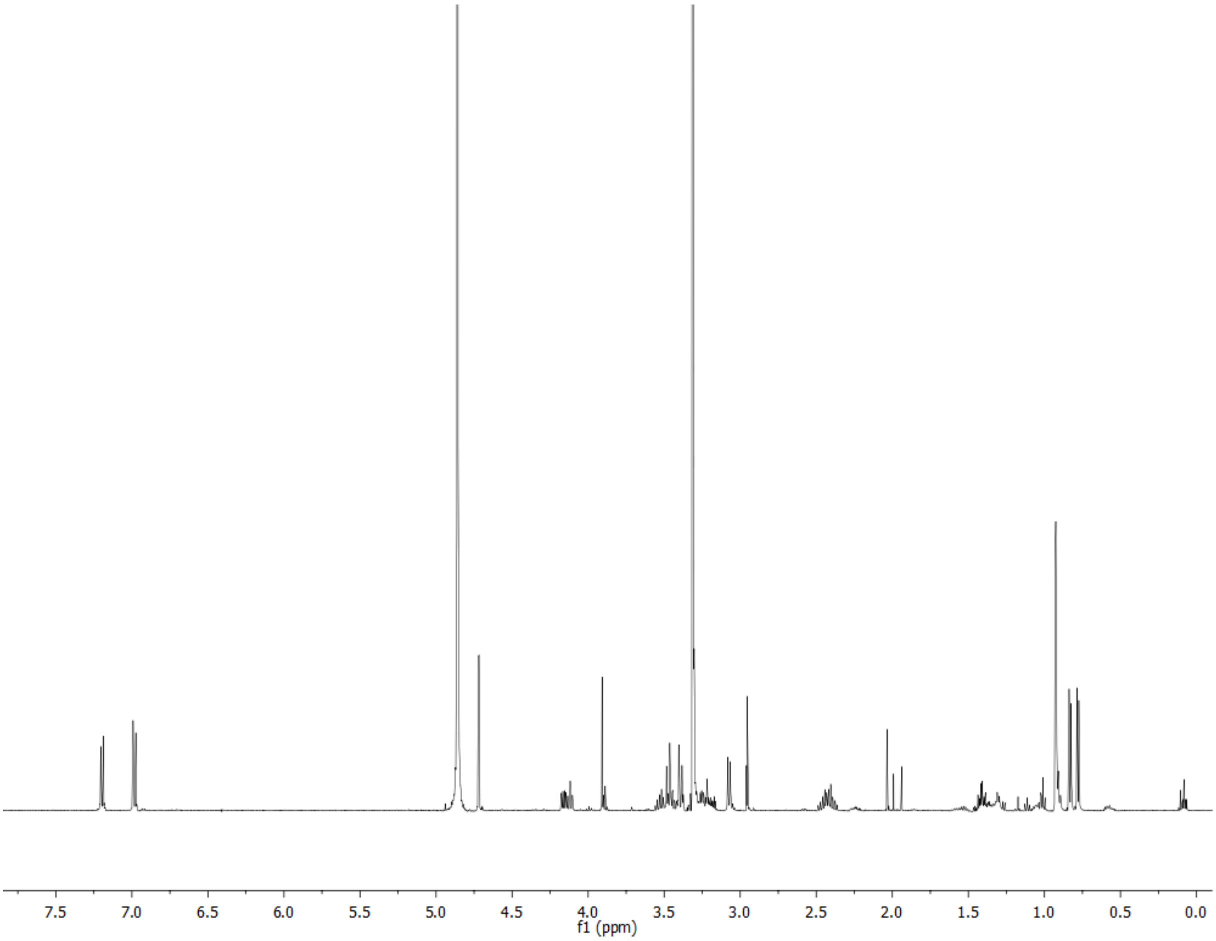

***O*-propargyl-D-Tyr-L-Leu-pant-amide:** ^{13}C-NMR (MeOD)

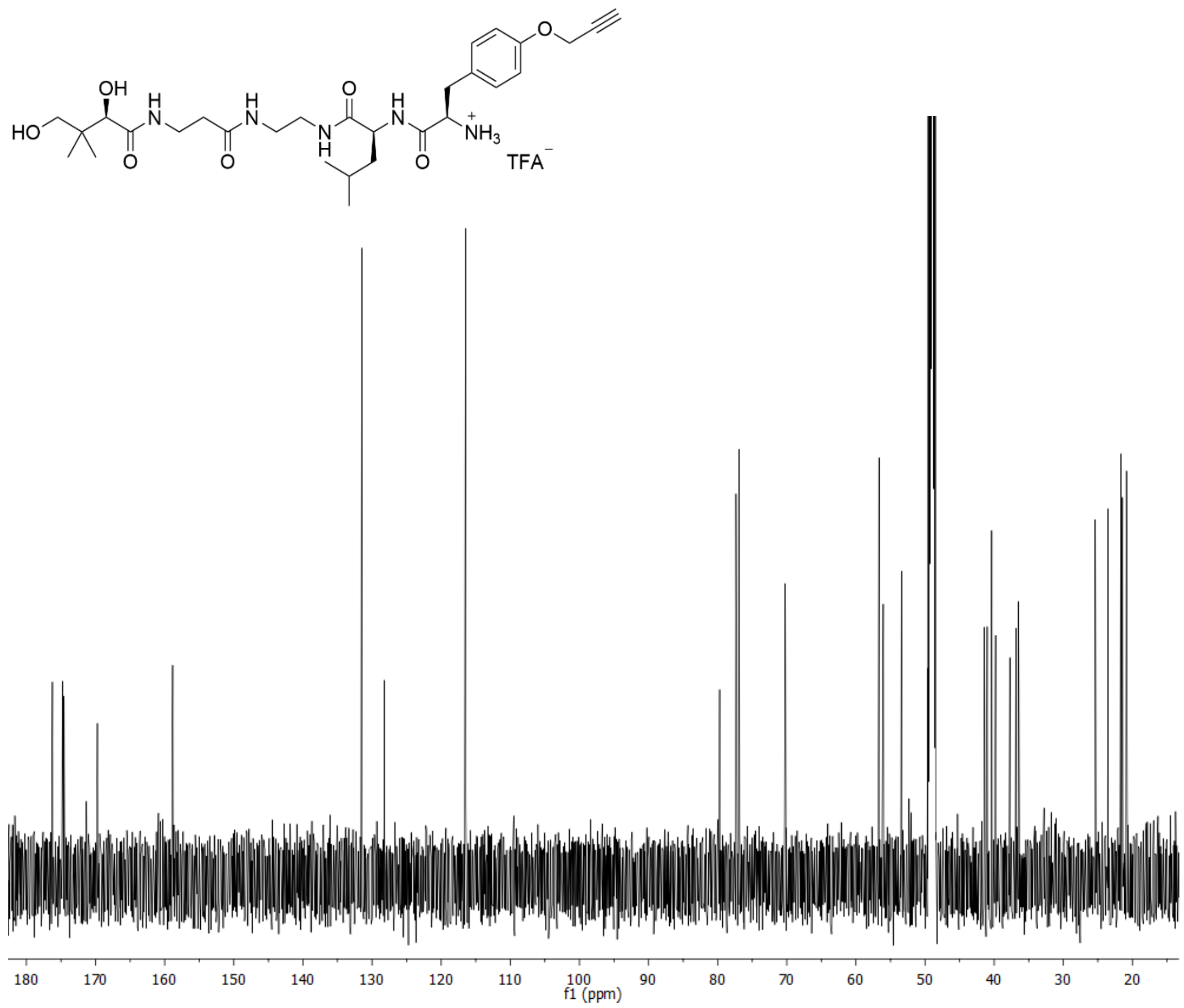

***O*-propargyl-D-Tyr-L-Leu-pant-amide:** HSQC (MeOD)

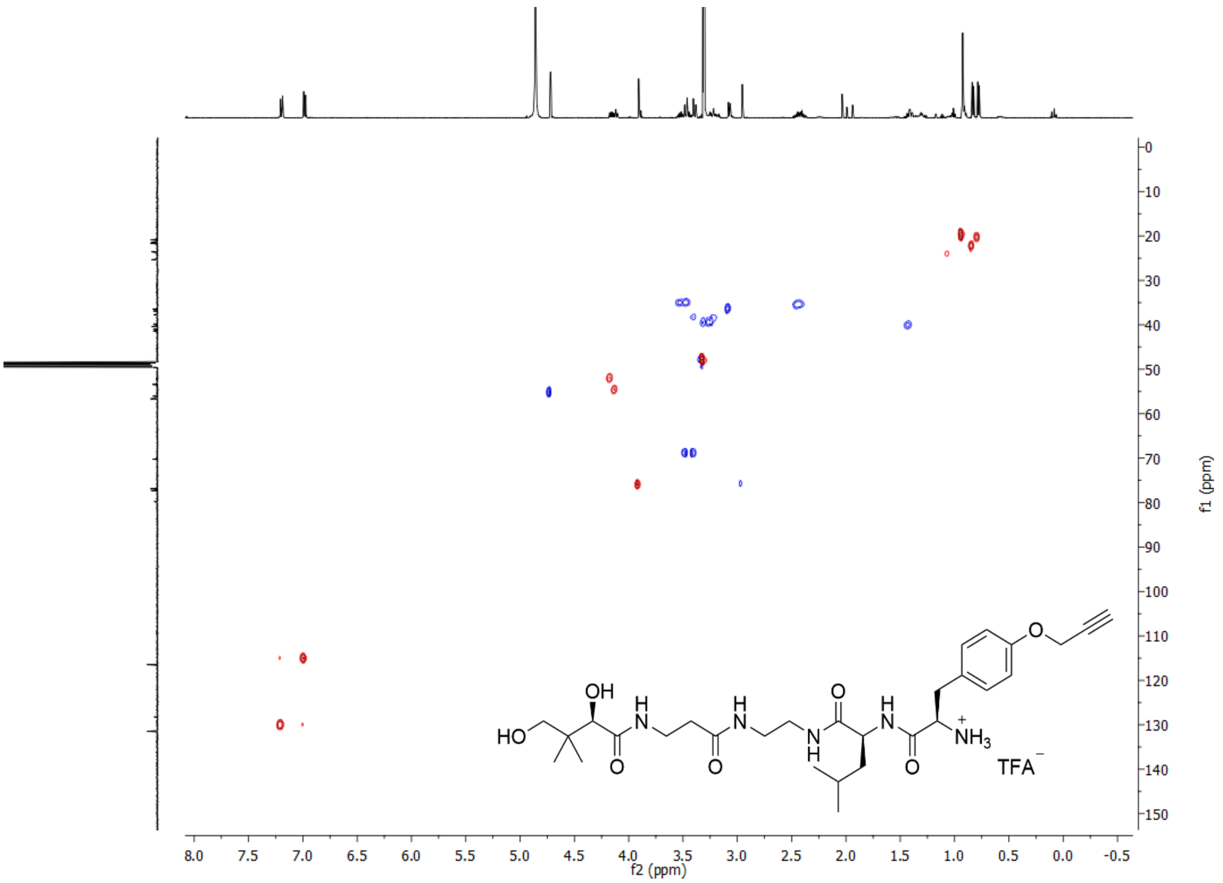

***O*-propargyl-D-Tyr-L-Leu-pant-amide:** HMBC (MeOD)

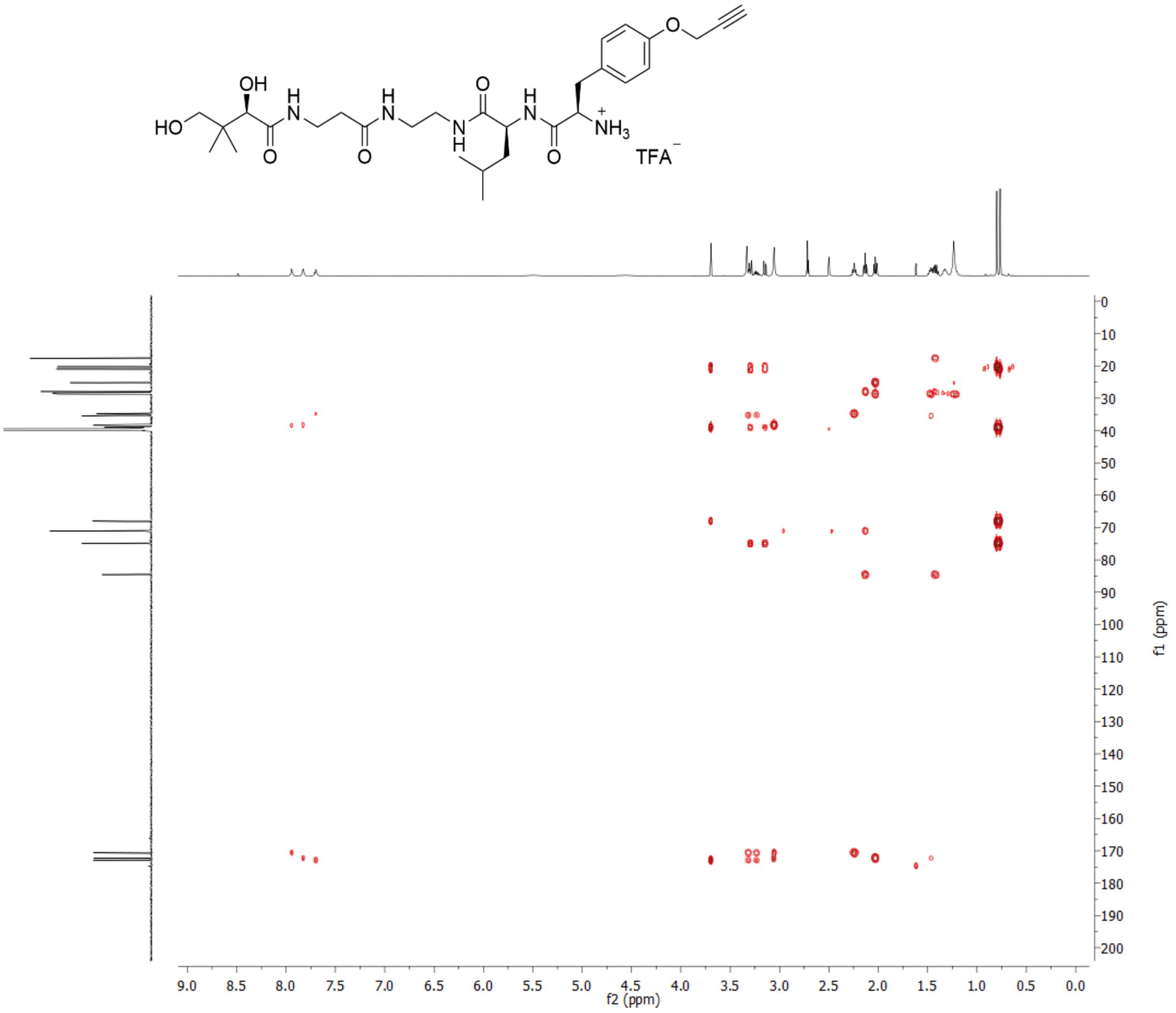

***O*-propargyl-D-Tyr-L-Leu:** ^{1}H-NMR (MeOD)

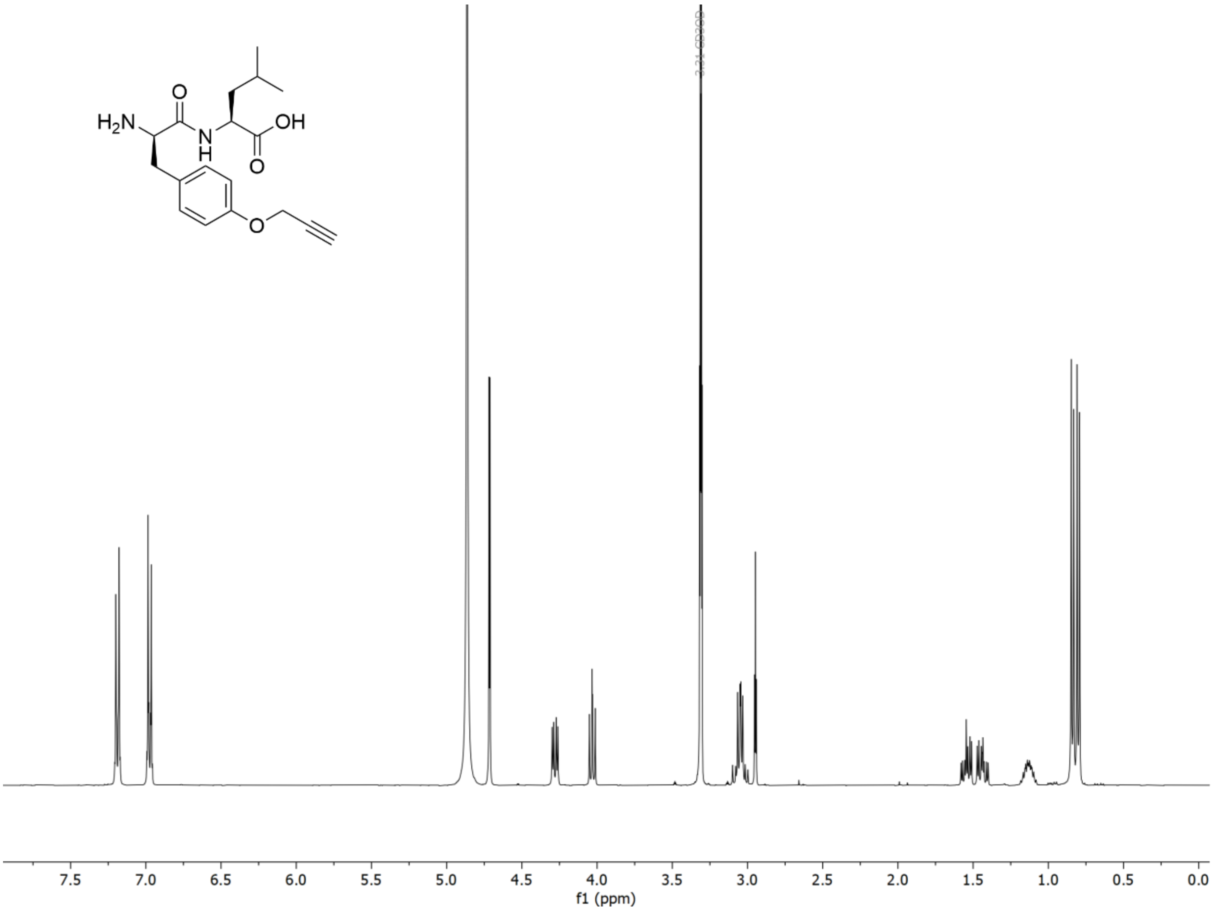

***O*-propargyl-D-Tyr-L-Leu:** ^{13}C-NMR (MeOD)

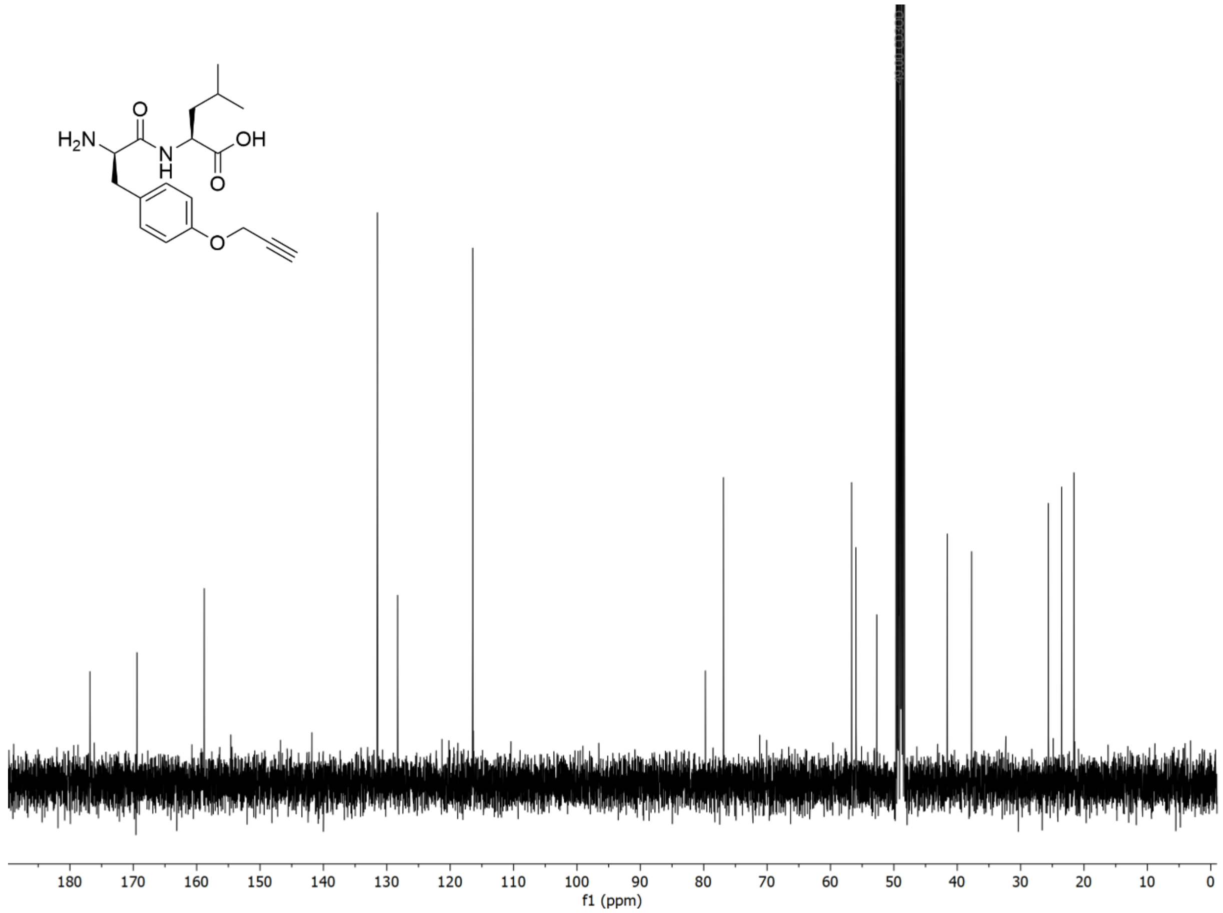

References

1. Kries, H. *et al.* Reprogramming nonribosomal peptide synthetases for ‘clickable’ amino acids. *Angew. Chem. Int. Ed.* **53**, 10105–10108 (2014).

2. Niquille, D. L. *et al.* Nonribosomal biosynthesis of backbone-modified peptides. *Nat. Chem.* **10**, 282–287 (2018).

3. Yan, X., Akinnusi, T. O., Larsen, A. T. & Auclair, K. Synthesis of 4′-aminopantetheine and derivatives to probe aminoglycoside N-6′-acetyltransferase. *Org. Biomol. Chem.* **9**, 1538–1546 (2011).

4. Beld, J., Cang, H. & Burkart, M. D. Visualizing the chain-flipping mechanism in fatty-acid biosynthesis. *Angew. Chem. Int. Ed.* **53**, 14456–14461 (2014).

5. Zemskov, I., Altaner, S., Dietrich, D. R. & Wittmann, V. Total synthesis of microcystin-LF and derivatives thereof. *J. Org. Chem.* **82**, 3680–3691 (2017).

6. Niquille, D. L., Folger, I. B., Basler, S. & Hilvert, D. Biosynthetic functionalization of nonribosomal peptides. *J. Am. Chem. Soc.* **143**, 2736–2740 (2021).

7. Gruenewald, S., Mootz, H. D., Stehmeier, P. & Stachelhaus, T. In vivo production of artificial nonribosomal peptide products in the heterologous host Escherichia coli. *Appl. Environ. Microbiol.* **70**, 3282–3291 (2004).

8. Der, B. S. *et al.* Metal-mediated affinity and orientation specificity in a computationally designed protein homodimer. *J. Am. Chem. Soc.* **134**, 375–385 (2012).

9. Strauss, E. & Begley, T. P. The antibiotic activity of N-pentylpantothenamide results from its conversion to ethyldethia-coenzyme A, a coenzyme A antimetabolite. *J. Biol. Chem.* **277**, 48205–48209 (2002).

10. Gibson, D. G. *et al.* Enzymatic assembly of DNA molecules up to several hundred kilobases. *Nat. Methods* **6**, 343–345 (2009).

11. Hahn, M. & Stachelhaus, T. Selective interaction between nonribosomal peptide synthetases is facilitated by short communication-mediating domains. *Proc. Natl. Acad. Sci. U. S. A.* **101**, 15585–15590 (2004).

12. Pospiech, A. & Neumann, B. A versatile quick-prep of genomic DNA from Gram-positive bacteria. *Trends Genet.* **11**, 217–218 (1995).

13. Wang, Z., Mathias, A., Stavrou, S. & Neville, D. M. A new yeast display vector permitting free scFv amino termini can augment ligand binding affinities. *Protein Eng. Des. Sel.* **18**, 337–343 (2005).

14. Studier, F. W. Protein production by auto-induction in high-density shaking cultures. *Protein*

Expr. Purif. **41**, 207–234 (2005).

15. Scheller, C., Krebs, F., Wiesner, R., Wätzig, H. & Oltmann-Norden, I. A comparative study of CE-SDS, SDS-PAGE, and Simple Western—Precision, repeatability, and apparent molecular mass shifts by glycosylation. *Electrophoresis* **42**, 1521–1531 (2021).

16. Frank, R. N. & Rodbard, D. Precision of sodium dodecyl sulfate-polyacrylamide-gel electrophoresis for the molecular weight estimation of a membrane glycoprotein: Studies on bovine rhodopsin. *Arch. Biochem. Biophys.* **171**, 1–13 (1975).

17. Banker, G. A. & Cotman, C. W. Measurement of Free Electrophoretic Mobility and Retardation Coefficient of Protein-Sodium Dodecyl Sulfate Complexes by Gel Electrophoresis: A method to validate molecular weight estimates. *J. Biol. Chem.* **247**, 5856–5861 (1972).

18. Nazi, I., Koteva, K. P. & Wright, G. D. One-pot chemoenzymatic preparation of coenzyme A analogues. *Anal. Biochem.* **324**, 100–105 (2004).

19. Tanovic, A., Samel, S. A., Essen, L.-O. & Marahiel, M. A. Crystal structure of the termination module of a nonribosomal peptide synthetase. *Science* **321**, 659–663 (2008).

Source Data

Source Data Suppl. Fig. 1

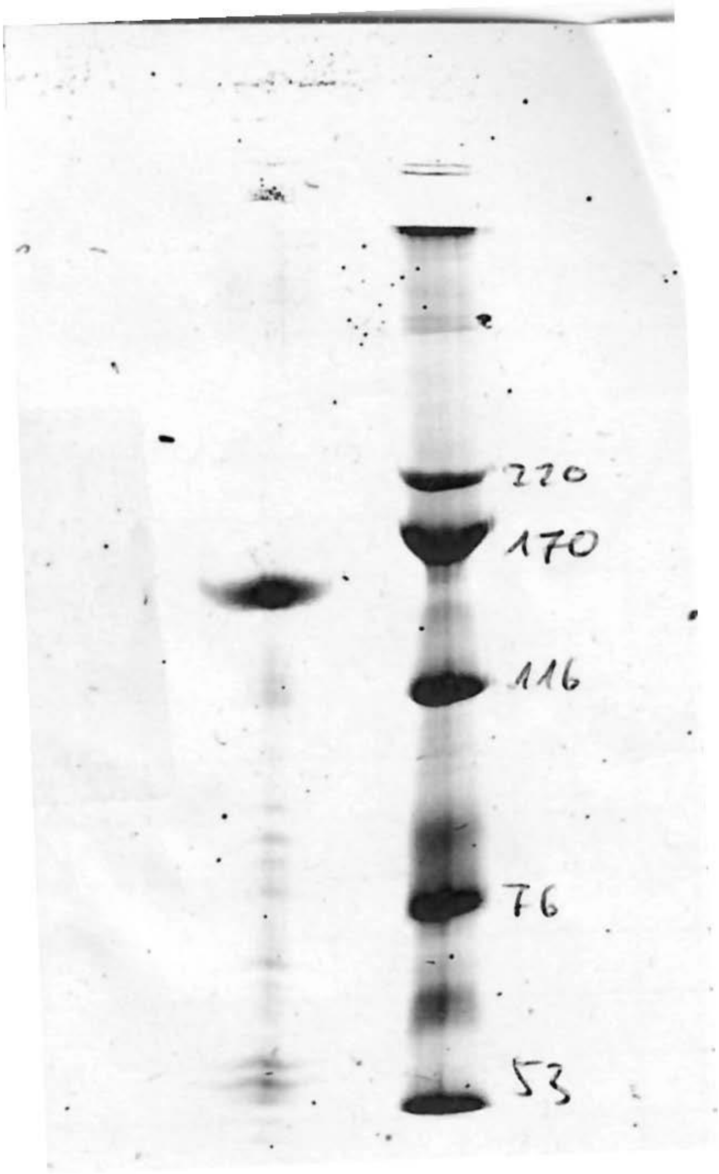

Source Data Suppl. Fig. 11

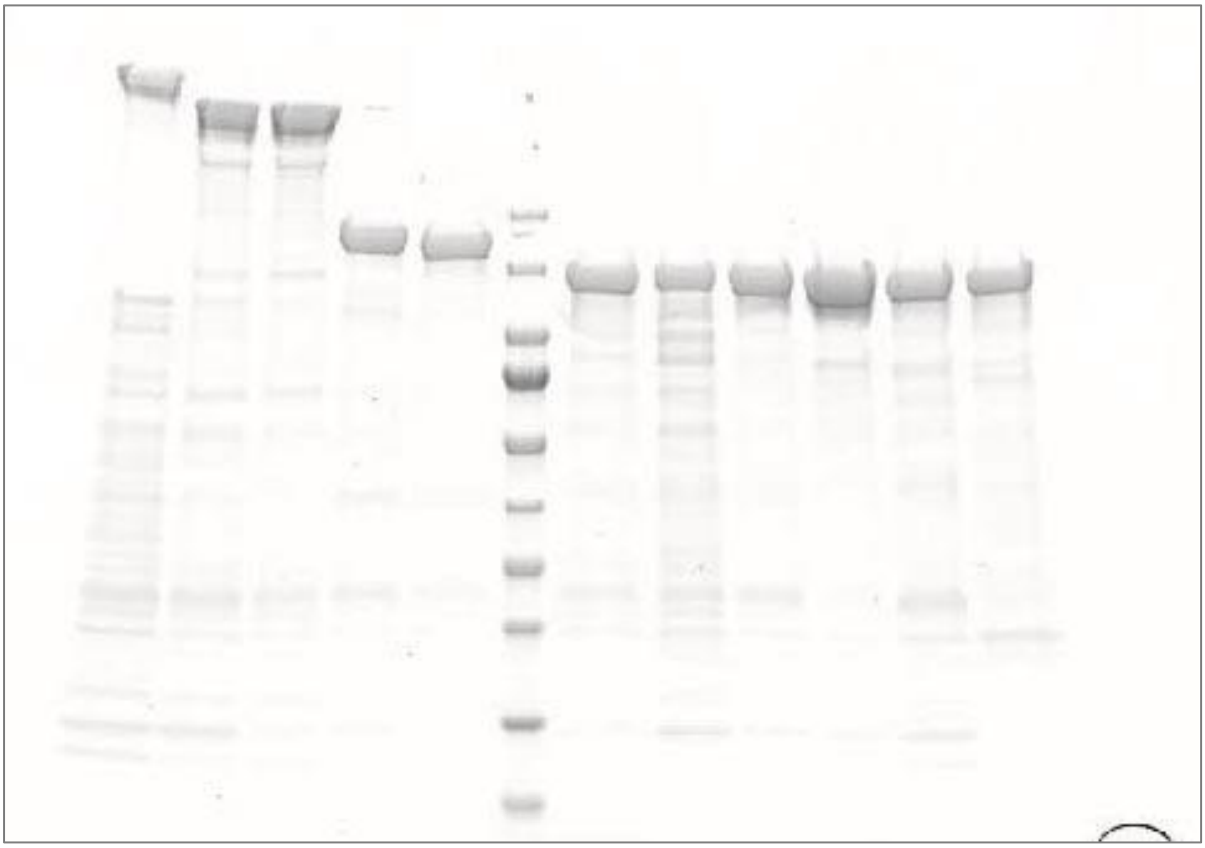

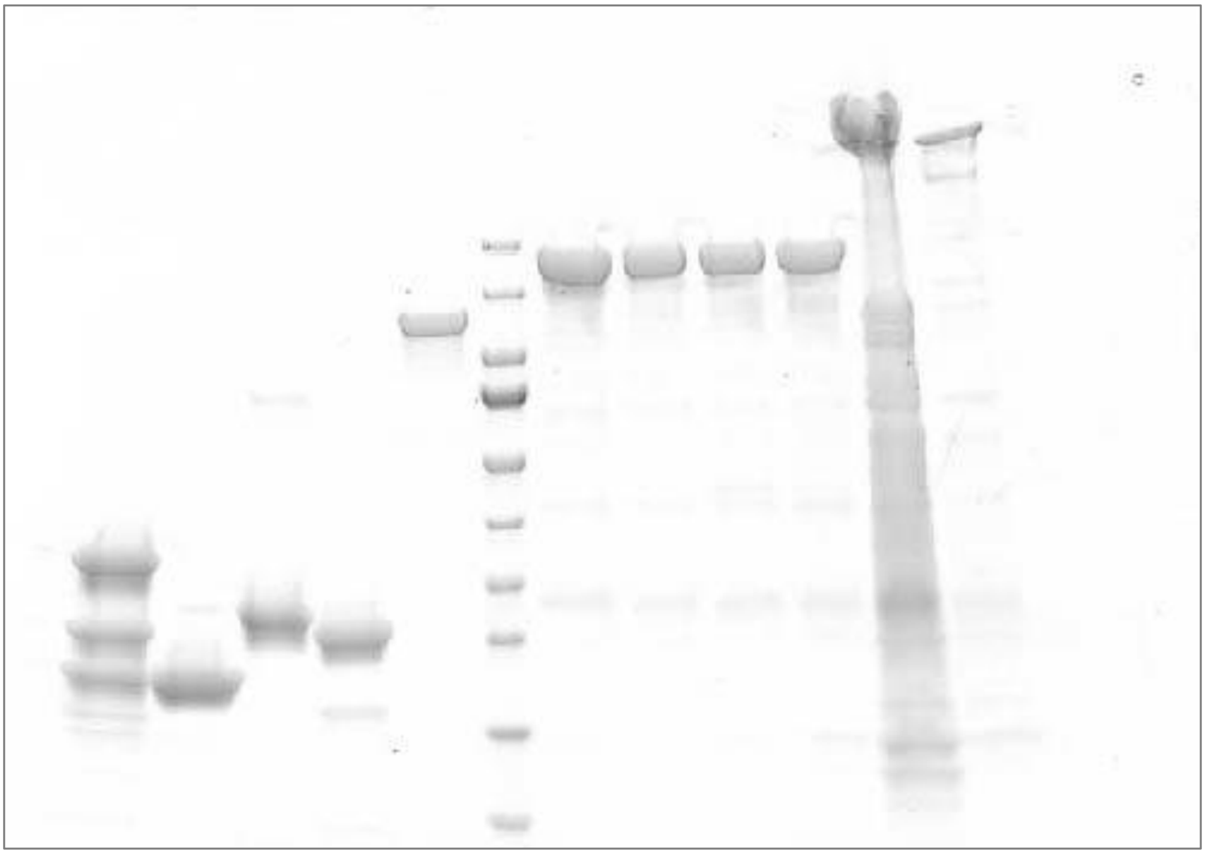

Supplement: Supplementary file 1 — Supplementary Note, Tables 1–3, Figs. 1–13, and supporting data for Supplementary Figs. 1 and 11. [file 41589_2023_1532_MOESM1_ESM.pdf]
