# Supplementary material for: High-throughput reprogramming of an NRPS condensation domain
Source: Nat Chem Biol. 2024 Feb 2;20(6):761–9. doi: 10.1038/s41589-023-01532-x (PMC11142918; doi:10.1038/s41589-023-01532-x)
Supplement: Supplementary file 2 — Reporting Summary [file 41589_2023_1532_MOESM2_ESM.pdf]

nature portfolio

Corresponding author(s): Donald Hilvert

Last updated by author(s): Nov 1, 2023

Reporting Summary

Nature Portfolio wishes to improve the reproducibility of the work that we publish. This form provides structure for consistency and transparency in reporting. For further information on Nature Portfolio policies, see our Editorial Policies and the Editorial Policy Checklist.

Statistics

For all statistical analyses, confirm that the following items are present in the figure legend, table legend, main text, or Methods section.

n/a	Confirmed	
☐	☒	The exact sample size (*n*) for each experimental group/condition, given as a discrete number and unit of measurement
☐	☒	A statement on whether measurements were taken from distinct samples or whether the same sample was measured repeatedly
☒	☐	The statistical test(s) used AND whether they are one- or two-sided<br>*Only common tests should be described solely by name; describe more complex techniques in the Methods section.*
☒	☐	A description of all covariates tested
☒	☐	A description of any assumptions or corrections, such as tests of normality and adjustment for multiple comparisons
☐	☒	A full description of the statistical parameters including central tendency (e.g. means) or other basic estimates (e.g. regression coefficient) AND variation (e.g. standard deviation) or associated estimates of uncertainty (e.g. confidence intervals)
☒	☐	For null hypothesis testing, the test statistic (e.g. *F*, *t*, *r*) with confidence intervals, effect sizes, degrees of freedom and *P* value noted<br>*Give P values as exact values whenever suitable.*
☒	☐	For Bayesian analysis, information on the choice of priors and Markov chain Monte Carlo settings
☒	☐	For hierarchical and complex designs, identification of the appropriate level for tests and full reporting of outcomes
☒	☐	Estimates of effect sizes (e.g. Cohen's *d*, Pearson's *r*), indicating how they were calculated

Our web collection on statistics for biologists contains articles on many of the points above.

Software and code

Policy information about availability of computer code

Data collection	BD FACSDiva™ Software was used to collect data on LSRFortessa Cell Analyzer (BD) as well as on FACSAria III cell sorter (BD).<br>MassLynx was used to collect and analyse data collected on LC-MS (Waters H-class UPLC/SQD-2).<br>Data on the Thermo nanoAcquity UPLC coupled to a Q Exactive mass spectrometer was collected using Thermo Exactive software.<br>Data run on U-HPLC (Ultimate 3000, Dionex) were collected using Chromeleon (Version 7.2 SR4).<br>Diffraction data sets were collected using beamline CMCF-ID of the Canadian Light Source, NE-CAT 24-ID-E beamline at the Advanced Photon Source (APS) and beamline 23ID-B at APS during the CCP4/APS school in macromolecular crystallography.<br>Data of LC-HRMS/MS and LC-HRMS measurements were collected on a Bruker maxis-ESI-Qq-TOF-MS using Bruker Compass DataAnalysis (Version 5.3).
Data analysis	Flow cytometry and flourescence activated cell sorting data was analysed using FlowJo (Versoin 10.5-10.8).<br>MassLynx was used to analyse data collected on LC-MS (Waters H-class UPLC/SQD-2). Calculations of kinetic parameters were performed in Microsoft Excel and plots were generated using GraphPad Prism (Version 8.0.0 - 10.0.3).<br>DNA sequences were analysed using CLC Genomics Workbench (Version 10-11).<br>Data on the Thermo nanoAcquity UPLC coupled to a Q Exactive mass spectrometer was analysed utilizing PEAKS.<br>Data run on U-HPLC (Ultimate 3000, Dionex) were analysed using Chromeleon (Version 7.2 SR4). Calculations of kinetic parameters were performed in Microsoft Excel and plots were generated using GraphPad Prism (Version 8.0.0 - 10.0.3).<br>NMR data was analysed using MestReNova (MNova, Version 14).<br>Diffraction data were indexed and scaled with softwares HKL2000 (Version 2.3.8), HKL3000 (Version 2.3.15) or DIALS (Version 3.8.0). Structure determination was accomplished using Phaser (Version 2.1), Coot and Phenix (Version 1.20.1 and Version 0.9.8.1). The data collection and refinement statistics are summarized in Supplementary Table 2.

Data of LC-HRMS/MS and LC-HRMS measurements were analysed using Bruker Compass DataAnalysis (Version 5.3).

For manuscripts utilizing custom algorithms or software that are central to the research but not yet described in published literature, software must be made available to editors and reviewers. We strongly encourage code deposition in a community repository (e.g. GitHub). See the Nature Portfolio guidelines for submitting code & software for further information.

Data

Policy information about availability of data

All manuscripts must include a data availability statement. This statement should provide the following information, where applicable:

- Accession codes, unique identifiers, or web links for publicly available datasets
- A description of any restrictions on data availability
- For clinical datasets or third party data, please ensure that the statement adheres to our policy

The X-ray crystal structures and diffraction data from this study were deposited in the Research Collaboratory for Structural Bioinformatics Protein Data Bank (PDB) under accession codes 8F7F, 8F7G, 8F7H and 8F7I. Source Data for all figures in the main text and supplementary information have been supplied with the manuscript or as supplementary information files.

Human research participants

Policy information about studies involving human research participants and Sex and Gender in Research.

Reporting on sex and gender	N/A
Population characteristics	N/A
Recruitment	N/A
Ethics oversight	N/A

Note that full information on the approval of the study protocol must also be provided in the manuscript.

Field-specific reporting

Please select the one below that is the best fit for your research. If you are not sure, read the appropriate sections before making your selection.

☒ Life sciences ☐ Behavioural & social sciences ☐ Ecological, evolutionary & environmental sciences

For a reference copy of the document with all sections, see nature.com/documents/nr-reporting-summary-flat.pdf

Life sciences study design

All studies must disclose on these points even when the disclosure is negative.

Sample size	Sample size was not predetermined using statistical methods. To obtain kinetic data at least three biological replicates were performed on different days with different protein batches to account for differences in protein stability. Each biological replicate included duplicates or triplicates to account for pipetting errors. Biological replicates were reproducible and support the findings of this study. Similarly, for comparison of engineered C domain variants, two biological replicates were performed on different days. Biological replicates were reproducible and support the finding of this work. For TycA* stability assessment, one biological experiment was performed to support hypothesized TycA* instability. Although the experiment has not been replicated, similar experiments using slightly different conditions (data not shown in manuscript) supported this hypothesis.
Data exclusions	no data were excluded
Replication	Generated data was generally reproducible. Kinetic measurements were repeated 3-4 times (see above). Kinetic comparison of C domain variants was performed twice and TycA* stability test was performed once.
Randomization	Randomization was not relevant to this study as reagent preparation and culture conditions were kept constant. Experiments were performed with the exact same procedure, controls and data analysis methodology. Samples were analyzed individually and all parameters for protein expression were kept constant.
Blinding	Blinding was not relevant to study as data was collected by unbiased methods (i.e. machines: LC-MS, HPLC, flow cytometry, ...) and individual control samples have been included in every experiment performed and analysed individually.

Reporting for specific materials, systems and methods

We require information from authors about some types of materials, experimental systems and methods used in many studies. Here, indicate whether each material, system or method listed is relevant to your study. If you are not sure if a list item applies to your research, read the appropriate section before selecting a response.

Materials & experimental systems

n/a	Involved in the study
☐	☒ Antibodies
☐	☒ Eukaryotic cell lines
☒	☐ Palaeontology and archaeology
☒	☐ Animals and other organisms
☒	☐ Clinical data
☒	☐ Dual use research of concern

Methods

n/a	Involved in the study
☒	☐ ChIP-seq
☐	☒ Flow cytometry
☒	☐ MRI-based neuroimaging

Antibodies

Antibodies used	anti-c-myc 9E10 from mouse IgG1κ (Millipore Sigma, ROAMYC, 11667203001); Anti-Mouse IgG (whole molecule)–FITC antibody produced in goat (Millipore Sigma, F2012)
Validation	validated by manufacturer (https://www.sigmaaldrich.com/US/en/product/roche/roamyc)

Eukaryotic cell lines

Policy information about cell lines and Sex and Gender in Research

Cell line source(s)	EBY100 originally obtained from Plückthun research group at University of Zurich who ordered the strain from ATCC.
Authentication	not authenticated
Mycoplasma contamination	not tested for mycoplasma contamination
Commonly misidentified lines (See ICLAC register)	n/a

Flow Cytometry

Plots

Confirm that:

☒ The axis labels state the marker and fluorochrome used (e.g. CD4-FITC).

☒ The axis scales are clearly visible. Include numbers along axes only for bottom left plot of group (a 'group' is an analysis of identical markers).

☒ All plots are contour plots with outliers or pseudocolor plots.

☒ A numerical value for number of cells or percentage (with statistics) is provided.

Methodology

Sample preparation	Proteins were displayed on cells and assayed as described in text. Cells were fluorescently labeled via streptavidin-R-PE and anti-myc-FITC and directly subjected to flow cytometry. Cells were gated for single cells via standard procedures (plotting SSC-A vs FSC-A and SSC-H vs SSC-A for flow cytometry analysis and plotting SSC-A vs FSC-A, FSC-H vs FSC-A, and FSC-H vs FSC-W for FACS) and single cells were analyzed as described/shown in manuscript.
Instrument	LSRFortessa Cell Analyzer (BD) and FACSAria III cell sorter (BD)
Software	Software of BD Instruments for data collection and data analysis using FlowJo
Cell population abundance	Single cells represented usually 65-80% of total cell population

Gating strategy	Single cells were gated (plotting SSC-A vs FSC-A and SSC-H vs SSC-A for flow cytometry analysis and plotting SSC-A vs FSC-A, FSC-H vs FSC-A, and FSC-H vs FSC-W for FACS) and single cell population was analyzed and gated using appropriate negative cell poppulation controls that were not fluorescently labeled, displayed inactive enzyme variants or were unable to produce products (see Suppl. Fig. 2 for details)

☒ Tick this box to confirm that a figure exemplifying the gating strategy is provided in the Supplementary Information.
