# Supplementary material for: High-throughput reprogramming of an NRPS condensation domain
Source: Nat Chem Biol. 2024 Feb 2;20(6):761–9. doi: 10.1038/s41589-023-01532-x (PMC11142918; doi:10.1038/s41589-023-01532-x)
Supplement: Supplementary file 3 — Supporting data for Supplementary Figs. 1, 2 and 4–10. [file 41589_2023_1532_MOESM3_ESM.zip › Suppl. Fig. 1/Source Data Suppl. Fig. 1b-c.pdf]

Probability Legend

- over 95%
- 80% to 94%
- 50% to 79%
- 20% to 49%
- 0% to 19%

#	Visible?	Starred?	BioView: 1158 Proteins in 1038 Clusters With 5 Decoys and 22 Hidden	Accession Number	Molecular Weight	Protein Grouping Ambiguity	SrfA_C_CAT
1	☑	☆	- order o22828	xx|SrfA-C_CAT|SrfA-C_CAT	131 kDa	★	258
2	☑	☆	Peptide-N(4)-(N-acetyl-beta-D-glucosaminyl)asparagine amidase F OS=Elizabethkingia miricola OX=1720...	P21163|PNGF_ELIMR	39 kDa		71
+ 3	☑	☆	Cluster of Glyceraldehyde-3-phosphate dehydrogenase 3 OS=Saccharomyces cerevisiae (strain ATCC 204...	P00359|G3P3_YEAST [11]	36 kDa	★	99
+ 4	☑	☆	Cluster of Pyruvate decarboxylase isozyme 1 OS=Saccharomyces cerevisiae (strain ATCC 204508 / S288c...	P06169|PDC1_YEAST [3]	61 kDa	★	85
+ 5	☑	☆	Cluster of Enolase 2 OS=Saccharomyces cerevisiae (strain ATCC 204508 / S288c) OX=559292 GN=ENO2 PE...	P00925|ENO2_YEAST [5]	47 kDa	★	88
6	☑	☆	Fatty acid synthase subunit alpha OS=Saccharomyces cerevisiae (strain ATCC 204508 / S288c) OX=55929...	P19097|FAS2_YEAST	207 kDa		103
7	☑	☆	Fatty acid synthase subunit beta OS=Saccharomyces cerevisiae (strain ATCC 204508 / S288c) OX=559292...	P07149|FAS1_YEAST	229 kDa		111
+ 8	☑	☆	Cluster of Pyruvate kinase 1 OS=Saccharomyces cerevisiae (strain ATCC 204508 / S288c) OX=559292 GN...	P00549|KPYK1_YEAST [4]	55 kDa	★	70
+ 9	☑	☆	Cluster of Fructose-bisphosphate aldolase OS=Saccharomyces cerevisiae (strain ATCC 204508 / S288c) O...	P14540|ALF_YEAST [2]	40 kDa	★	60
10	☑	☆	Invertase 2 OS=Saccharomyces cerevisiae (strain ATCC 204508 / S288c) OX=559292 GN=SUC2 PE=1 SV=1	P00724|INV2_YEAST	61 kDa		64
11	☑	☆	Acetyl-CoA carboxylase OS=Saccharomyces cerevisiae (strain ATCC 204508 / S288c) OX=559292 GN=ACC...	Q00955|ACAC_YEAST	250 kDa		88
+ 12	☑	☆	Cluster of Heat shock protein SSA1 OS=Saccharomyces cerevisiae (strain ATCC 204508 / S288c) OX=5592...	P10591|HSP71_YEAST [13]	70 kDa	★	68
+ 13	☑	☆	Cluster of Elongation factor 2 OS=Saccharomyces cerevisiae (strain ATCC 204508 / S288c) OX=559292 GN...	P32324|EF2_YEAST [3]	93 kDa	★	55
+ 14	☑	☆	Cluster of Phosphoglycerate kinase OS=Saccharomyces cerevisiae (strain ATCC 204508 / S288c) OX=5592...	P00560|PGK_YEAST [5]	45 kDa	★	48
15	☑	☆	Potassium-activated aldehyde dehydrogenase, mitochondrial OS=Saccharomyces cerevisiae (strain ATCC...	P46367|ALDH4_YEAST	57 kDa	★	42
16	☑	☆	Protein URA2 OS=Saccharomyces cerevisiae (strain ATCC 204508 / S288c) OX=559292 GN=URA2 PE=1 SV...	P07259|PYR1_YEAST	245 kDa	★	61
+ 17	☑	☆	Cluster of Elongation factor 1-alpha OS=Saccharomyces cerevisiae (strain ATCC 204508 / S288c) OX=5592...	P02994|EF1A_YEAST [7]	50 kDa	★	42
+ 18	☑	☆	Cluster of Plasma membrane ATPase 1 OS=Saccharomyces cerevisiae (strain ATCC 204508 / S288c) OX=5...	P05030|PMA1_YEAST [4]	100 kDa	★	46
19	☑	☆	Barrierpepsin OS=Saccharomyces cerevisiae (strain ATCC 204508 / S288c) OX=559292 GN=BAR1 PE=1 SV...	P12630|BAR1_YEAST	64 kDa		34
20	☑	☆	Glucan 1,3-beta-glucosidase I/II OS=Saccharomyces cerevisiae (strain ATCC 204508 / S288c) OX=559292...	P23776|EXG1_YEAST	51 kDa		44
21	☑	☆	Elongation factor 3A OS=Saccharomyces cerevisiae (strain ATCC 204508 / S288c) OX=559292 GN=YEF3 PE...	P16521|EF3A_YEAST	116 kDa		45
+ 22	☑	☆	Cluster of Heat shock protein homolog SSE1 OS=Saccharomyces cerevisiae (strain ATCC 204508 / S288c) ...	P32589|HSP7F_YEAST [2]	77 kDa	★	44
23	☑	☆	Protein ZPS1 OS=Saccharomyces cerevisiae (strain ATCC 204508 / S288c) OX=559292 GN=ZPS1 PE=3 SV=1	Q12512|ZPS1_YEAST	28 kDa		31
24	☑	☆	ATP synthase subunit beta, mitochondrial OS=Saccharomyces cerevisiae (strain ATCC 204508 / S288c) OX...	P00830|ATPB_YEAST	55 kDa		42
25	☑	☆	Hexokinase-1 OS=Saccharomyces cerevisiae (strain ATCC 204508 / S288c) OX=559292 GN=HXK1 PE=1 SV...	P04806|HXKA_YEAST	54 kDa	★	39
+ 26	☑	☆	Cluster of Constitutive acid phosphatase OS=Saccharomyces cerevisiae (strain ATCC 204508 / S288c) OX...	P24031|PPA3_YEAST [3]	53 kDa	★	49
+ 27	☑	☆	Cluster of Serine hydroxymethyltransferase, cytosolic OS=Saccharomyces cerevisiae (strain ATCC 204508...	P37291|GLYC_YEAST [3]	52 kDa	★	41
28	☑	☆	Endo-1,3(4)-beta-glucanase 1 OS=Saccharomyces cerevisiae (strain ATCC 204508 / S288c) OX=559292 G...	P53753|ENG1_YEAST	121 kDa		45
29	☑	☆	Major capsid protein OS=Saccharomyces cerevisiae virus L-BC OX=42478 GN=gag PE=3 SV=1	Q87026|GAG_SCVLB	78 kDa		38
30	☑	☆	Alcohol dehydrogenase 1 OS=Saccharomyces cerevisiae (strain ATCC 204508 / S288c) OX=559292 GN=AD...	P00330|ADH1_YEAST	37 kDa	★	29
31	☑	☆	Alcohol dehydrogenase 2 OS=Saccharomyces cerevisiae (strain ATCC 204508 / S288c) OX=559292 GN=AD...	P00331|ADH2_YEAST	37 kDa	★	30
+ 32	☑	☆	Cluster of ATP-dependent molecular chaperone HSC82 OS=Saccharomyces cerevisiae (strain ATCC 20450...	P15108|HSC82_YEAST [3]	81 kDa	★	45
+ 33	☑	☆	Cluster of Bifunctional purine biosynthesis protein ADE17 OS=Saccharomyces cerevisiae (strain ATCC 2045...	P38009|PUR92_YEAST [2]	65 kDa	★	45
34	☑	☆	Acetyl-coenzyme A synthetase 1 OS=Saccharomyces cerevisiae (strain ATCC 204508 / S288c) OX=55929...	Q01574|ACS1_YEAST	79 kDa	★	37
35	☑	☆	Desmoplakin OS=Homo sapiens OX=9606 GN=DSP PE=1 SV=3	P15924|DESP_HUMAN	332 kDa	★	46
36	☑	☆	Bifunctional protein GAL10 OS=Saccharomyces cerevisiae (strain ATCC 204508 / S288c) OX=559292 GN=G...	P04397|GAL10_YEAST	78 kDa		38
+ 37	☑	☆	Cluster of Actin OS=Candida glabrata (strain ATCC 2001 / CBS 138 / JCM 3761 / NBRC 0622 / NRRL Y-65) O...	P60009|ACT_CANGA [6]	42 kDa	★	32

Probability Legend

- over 95%
- 80% to 94%
- 50% to 79%
- 20% to 49%
- 0% to 19%

#	Visible?	Starred?	BioView: 1158 Proteins in 1038 Clusters With 5 Decoys and 22 Hidden	Accession Number	Molecular Weight	Protein Grouping Ambiguity	SrfA_C_CAT
38	☑		Cluster of Ribosome-associated molecular chaperone SSB2 OS=Saccharomyces cerevisiae (strain ATCC 20...	P40150|SSB2_YEAST [2]	67 kDa	★	35
39	☑		Cluster of 60S ribosomal protein L4-A OS=Saccharomyces cerevisiae (strain ATCC 204508 / S288c) OX=55...	P10664|RL4A_YEAST [2]	39 kDa	★	27
40	☑		Cluster of V-type proton ATPase catalytic subunit A OS=Saccharomyces cerevisiae (strain ATCC 204508 / ...	P17255|VATA_YEAST [2]	119 kDa	★	37
41	☑		Galactokinase OS=Saccharomyces cerevisiae (strain ATCC 204508 / S288c) OX=559292 GN=GAL1 PE=1 SV...	P04385|GAL1_YEAST	58 kDa		27
42	☑		Triosephosphate isomerase OS=Saccharomyces cerevisiae (strain ATCC 204508 / S288c) OX=559292 GN=...	P00942|TPIS_YEAST	27 kDa		23
43	☑		Magnesium-activated aldehyde dehydrogenase, cytosolic OS=Saccharomyces cerevisiae (strain ATCC 204...	P54115|ALDH6_YEAST	54 kDa		28
44	☑		Glucan 1,3-beta-glucosidase OS=Saccharomyces cerevisiae (strain ATCC 204508 / S288c) OX=559292 GN...	P15703|BGL2_YEAST	34 kDa		23
45	☑		Probable family 17 glucosidase SCW4 OS=Saccharomyces cerevisiae (strain ATCC 204508 / S288c) OX=55...	P53334|SCW4_YEAST	40 kDa		27
46	☑		Cluster of Glucose-6-phosphate isomerase OS=Saccharomyces cerevisiae (strain ATCC 204508 / S288c) O...	P12709|G6PI_YEAST [2]	61 kDa	★	31
47	☑		ATP-dependent 6-phosphofructokinase subunit alpha OS=Saccharomyces cerevisiae (strain ATCC 204508 ...	P16861|PFKA1_YEAST	108 kDa	★	37
48	☑		5-methyltetrahydropteroyltriglutamate--homocysteine methyltransferase OS=Saccharomyces cerevisia...	P05694|METE_YEAST	86 kDa		31
49	☑		NAD-specific glutamate dehydrogenase OS=Saccharomyces cerevisiae (strain ATCC 204508 / S288c) OX=...	P33327|DHE2_YEAST	124 kDa		35
50	☑		Citrate synthase, mitochondrial OS=Saccharomyces cerevisiae (strain ATCC 204508 / S288c) OX=559292 ...	P00890|CISY1_YEAST	53 kDa	★	29
51	☑		Cluster of ATP synthase subunit alpha, mitochondrial OS=Saccharomyces cerevisiae (strain ATCC 204508 ...	P07251|ATPA_YEAST [2]	59 kDa	★	31
52	☑		Cluster of Pentafunctional AROM polypeptide OS=Saccharomyces cerevisiae (strain JAY291) OX=574961 ...	C7GIN5|ARO1_YEAS2 [3]	175 kDa	★	35
53	☑		Inositol-3-phosphate synthase OS=Saccharomyces cerevisiae (strain ATCC 204508 / S288c) OX=559292 ...	P11986|INO1_YEAST	60 kDa		24
54	☑		2-oxoglutarate dehydrogenase, mitochondrial OS=Saccharomyces cerevisiae (strain ATCC 204508 / S288...	P20967|ODO1_YEAST	114 kDa		27
55	☑		Transketolase 1 OS=Saccharomyces cerevisiae (strain ATCC 204508 / S288c) OX=559292 GN=TKL1 PE=1 ...	P23254|TKT1_YEAST	74 kDa		31
56	☑		Phosphoglycerate mutase 1 OS=Saccharomyces cerevisiae (strain ATCC 204508 / S288c) OX=559292 GN=...	P00950|PMG1_YEAST	28 kDa		24
57	☑		L-2-aminoadipate reductase OS=Saccharomyces cerevisiae (strain ATCC 204508 / S288c) OX=559292 GN...	P07702|LYS2_YEAST	155 kDa		32
58	☑		Cluster of Protein BMH2 OS=Saccharomyces cerevisiae (strain ATCC 204508 / S288c) OX=559292 GN=BMH...	P34730|BMH2_YEAST [2]	31 kDa	★	24
59	☑		ATP-dependent 6-phosphofructokinase subunit beta OS=Saccharomyces cerevisiae (strain ATCC 204508 /...	P16862|PFKA2_YEAST	105 kDa	★	30
60	☑		Cluster of Guanine nucleotide-binding protein subunit beta-like protein OS=Saccharomyces cerevisiae (str...	P38011|GBLP_YEAST [2]	35 kDa	★	25
61	☑		Acetyl-coenzyme A synthetase 2 OS=Saccharomyces cerevisiae (strain ATCC 204508 / S288c) OX=55929...	P52910|ACS2_YEAST	75 kDa	★	29
62	☑		Ketol-acid reductoisomerase, mitochondrial OS=Saccharomyces cerevisiae (strain ATCC 204508 / S288c) ...	P06168|ILV5_YEAST	44 kDa		25
63	☑		Cluster of Cell division control protein 48 OS=Saccharomyces cerevisiae (strain ATCC 204508 / S288c) OX=...	P25694|CDC48_YEAST [6]	92 kDa	★	30
64	☑		Protein YGP1 OS=Saccharomyces cerevisiae (strain ATCC 204508 / S288c) OX=559292 GN=YGP1 PE=1 SV=2	P38616|YGP1_YEAST	37 kDa		24
65	☑		Hexokinase-2 OS=Saccharomyces cerevisiae (strain ATCC 204508 / S288c) OX=559292 GN=HXK2 PE=1 SV...	P04807|HXKB_YEAST	54 kDa	★	26
66	☑		Bifunctional purine biosynthetic protein ADE5,7 OS=Saccharomyces cerevisiae (strain ATCC 204508 / S288...	P07244|PUR2_YEAST	86 kDa		23
67	☑		Heat shock protein SSC1, mitochondrial OS=Saccharomyces cerevisiae (strain ATCC 204508 / S288c) OX=...	P0CS90|HSP77_YEAST (+1)	71 kDa	★	25
68	☑		V-type proton ATPase subunit B OS=Saccharomyces cerevisiae (strain ATCC 204508 / S288c) OX=559292 ...	P16140|VATB_YEAST	58 kDa		22
69	☑		ADP,ATP carrier protein 2 OS=Saccharomyces cerevisiae (strain ATCC 204508 / S288c) OX=559292 GN=PE...	P18239|ADT2_YEAST	34 kDa		22
70	☑		Heat shock protein 60, mitochondrial OS=Saccharomyces cerevisiae (strain ATCC 204508 / S288c) OX=55...	P19882|HSP60_YEAST	61 kDa		23
71	☑		Flavohemoprotein OS=Saccharomyces cerevisiae (strain ATCC 204508 / S288c) OX=559292 GN=YHB1 PE=...	P39676|FHP_YEAST	45 kDa		24
72	☑		60S ribosomal protein L3 OS=Saccharomyces cerevisiae (strain ATCC 204508 / S288c) OX=559292 GN=RP...	P14126|RL3_YEAST	44 kDa		20
73	☑		Aconitate hydratase, mitochondrial OS=Saccharomyces cerevisiae (strain ATCC 204508 / S288c) OX=559...	P19414|ACON_YEAST	85 kDa		24
74	☑		Mitochondrial outer membrane protein porin 1 OS=Saccharomyces cerevisiae (strain ATCC 204508 / S288...	P04840|VDAC1_YEAST	30 kDa		19

Probability Legend: over 95% | 80% to 94% | 50% to 79% | 20% to 49% | 0% to 19%

#	Visible?	Starred?	BioView: 1158 Proteins in 1038 Clusters With 5 Decoys and 22 Hidden	Accession Number	Molecular Weight	Protein Grouping Ambiguity	SrfA_C_CAT
75	☑	☆	Isoleucine--tRNA ligase, cytoplasmic OS=Saccharomyces cerevisiae (strain ATCC 204508 / S288c) OX=559...	P09436|SYIC_YEAST	123 kDa		29
76	☑	☆	Clathrin heavy chain OS=Saccharomyces cerevisiae (strain ATCC 204508 / S288c) OX=559292 GN=CHC1 P...	P22137|CLH_YEAST	187 kDa		28
⊞ 77	☑	☆	Cluster of Adenosylhomocysteinase OS=Saccharomyces cerevisiae (strain ATCC 204508 / S288c) OX=559...	P39954|SAHH_YEAST [2]	49 kDa	★	21
⊞ 78	☑	☆	Cluster of Asparagine synthetase [glutamine-hydrolyzing] 2 OS=Saccharomyces cerevisiae (strain ATCC 2...	P49090|ASNS2_YEAST [2]	65 kDa	★	28
⊞ 79	☑	☆	Cluster of Inosine-5'-monophosphate dehydrogenase 3 OS=Saccharomyces cerevisiae (strain ATCC 20450...	P50095|IMDH3_YEAST [2]	57 kDa	★	23
80	☑	☆	Adenylosuccinate lyase OS=Saccharomyces cerevisiae (strain ATCC 204508 / S288c) OX=559292 GN=ADE...	Q05911|PUR8_YEAST	55 kDa		25
⊞ 81	☑	☆	Cluster of 40S ribosomal protein S1-B OS=Saccharomyces cerevisiae (strain YJM789) OX=307796 GN=RPS...	A6ZM02|RS3A2_YEAS7 [9]	29 kDa	★	22
82	☑	☆	Protein S100-A8 OS=Homo sapiens OX=9606 GN=S100A8 PE=1 SV=1	P05109|S10A8_HUMAN	11 kDa		15
83	☑	☆	Peroxiredoxin TSA1 OS=Saccharomyces cerevisiae (strain ATCC 204508 / S288c) OX=559292 GN=TSA1 PE...	P34760|TSA1_YEAST	22 kDa		17
84	☑	☆	Phosphoribosylformylglycinamidine synthase OS=Saccharomyces cerevisiae (strain ATCC 204508 / S288c...	P38972|PUR4_YEAST	149 kDa		25
85	☑	☆	Phosphoenolpyruvate carboxykinase (ATP) OS=Saccharomyces cerevisiae (strain ATCC 204508 / S288c) ...	P10963|PCKA_YEAST	61 kDa		23
86	☑	☆	1,3-beta-glucanosyltransferase GAS1 OS=Saccharomyces cerevisiae (strain ATCC 204508 / S288c) OX=55...	P22146|GAS1_YEAST	60 kDa		21
87	☑	☆	Aspartate--tRNA ligase, cytoplasmic OS=Saccharomyces cerevisiae (strain ATCC 204508 / S288c) OX=559...	P04802|SYDC_YEAST	64 kDa		21
88	☑	☆	C-1-tetrahydrofolate synthase, cytoplasmic OS=Saccharomyces cerevisiae (strain ATCC 204508 / S288c) ...	P07245|C1TC_YEAST	102 kDa	★	25
89	☑	☆	Cytochrome b-c1 complex subunit 1, mitochondrial OS=Saccharomyces cerevisiae (strain ATCC 204508 / ...	P07256|QCR1_YEAST	50 kDa		22
⊞ 90	☑	☆	Cluster of NADP-specific glutamate dehydrogenase 1 OS=Saccharomyces cerevisiae (strain ATCC 204508 ...	P07262|DHE4_YEAST [2]	50 kDa	★	23
91	☑	☆	Galactose-1-phosphate uridylyltransferase OS=Saccharomyces cerevisiae (strain ATCC 204508 / S288c) ...	P08431|GAL7_YEAST	42 kDa		15
92	☑	☆	Endoplasmic reticulum chaperone BiP OS=Saccharomyces cerevisiae (strain ATCC 204508 / S288c) OX=55...	P16474|BIP_YEAST	74 kDa	★	23
93	☑	☆	Glucokinase-1 OS=Saccharomyces cerevisiae (strain ATCC 204508 / S288c) OX=559292 GN=GLK1 PE=1 SV...	P17709|HXKG_YEAST	55 kDa		20
94	☑	☆	40S ribosomal protein S2 OS=Saccharomyces cerevisiae (strain ATCC 204508 / S288c) OX=559292 GN=RP...	P25443|RS2_YEAST	27 kDa		18
95	☑	☆	Glycerol-3-phosphate dehydrogenase, mitochondrial OS=Saccharomyces cerevisiae (strain ATCC 204508 ...	P32191|GPDM_YEAST	72 kDa		23
96	☑	☆	Peroxiredoxin AHP1 OS=Saccharomyces cerevisiae (strain ATCC 204508 / S288c) OX=559292 GN=AHP1 PE...	P38013|AHP1_YEAST	19 kDa		20
⊞ 97	☑	☆	Cluster of 1,3-beta-glucan synthase component FKS1 OS=Saccharomyces cerevisiae (strain ATCC 204508 ...	P38631|FKS1_YEAST [3]	215 kDa	★	25
98	☑	☆	Saccharopine dehydrogenase [NADP(+), L-glutamate-forming] OS=Saccharomyces cerevisiae (strain ATC...	P38999|LYS9_YEAST	49 kDa		23
99	☑	☆	Histidine biosynthesis trifunctional protein OS=Saccharomyces cerevisiae (strain ATCC 204508 / S288c) O...	P00815|HIS2_YEAST	88 kDa		23
100	☑	☆	Valine--tRNA ligase, mitochondrial OS=Saccharomyces cerevisiae (strain ATCC 204508 / S288c) OX=55929...	P07806|SYV_YEAST	126 kDa		24
101	☑	☆	Malate dehydrogenase, mitochondrial OS=Saccharomyces cerevisiae (strain ATCC 204508 / S288c) OX=5...	P17505|MDHM_YEAST	36 kDa	★	22
102	☑	☆	Protein disulfide-isomerase OS=Saccharomyces cerevisiae (strain ATCC 204508 / S288c) OX=559292 GN=...	P17967|PDI_YEAST	58 kDa		20
103	☑	☆	Heat shock protein 104 OS=Saccharomyces cerevisiae (strain ATCC 204508 / S288c) OX=559292 GN=HSP...	P31539|HS104_YEAST	102 kDa		22
⊞ 104	☑	☆	Cluster of Pyruvate carboxylase 2 OS=Saccharomyces cerevisiae (strain ATCC 204508 / S288c) OX=55929...	P32327|PYC2_YEAST [2]	130 kDa	★	26
105	☑	☆	Importin subunit beta-4 OS=Saccharomyces cerevisiae (strain ATCC 204508 / S288c) OX=559292 GN=KAP...	P40069|IMB4_YEAST	123 kDa		23
106	☑	☆	External NADH-ubiquinone oxidoreductase 1, mitochondrial OS=Saccharomyces cerevisiae (strain ATCC 20...	P40215|NDH1_YEAST	63 kDa		23
107	☑	☆	Clustered mitochondria protein 1 OS=Saccharomyces cerevisiae (strain ATCC 204508 / S288c) OX=55929...	Q03690|CLU_YEAST	145 kDa		24
108	☑	☆	40S ribosomal protein S3 OS=Saccharomyces cerevisiae (strain ATCC 204508 / S288c) OX=559292 GN=RP...	P05750|RS3_YEAST	27 kDa		14
109	☑	☆	Argininosuccinate synthase OS=Saccharomyces cerevisiae (strain ATCC 204508 / S288c) OX=559292 GN=...	P22768|ASSY_YEAST	47 kDa		19
110	☑	☆	Peptidyl-prolyl cis-trans isomerase B OS=Saccharomyces cerevisiae (strain ATCC 204508 / S288c) OX=55...	P23285|CYPB_YEAST	23 kDa		17
111	☑	☆	Eukaryotic translation initiation factor 5A-1 OS=Saccharomyces cerevisiae (strain ATCC 204508 / S288c) ...	P23301|IF5A1_YEAST	17 kDa		15

Probability Legend: over 95% | 80% to 94% | 50% to 79% | 20% to 49% | 0% to 19%

#	Visible?	Starred?	BioView: 1158 Proteins in 1038 Clusters With 5 Decoys and 22 Hidden	Accession Number	Molecular Weight	Protein Grouping Ambiguity	SrfA_C_CAT
112	☑	☆	Hornerin OS=Homo sapiens OX=9606 GN=HRNR PE=1 SV=2	Q86YZ3|HORN_HUMAN	282 kDa		13
113	☑	☆	Pheromone-processing carboxypeptidase KEX1 OS=Saccharomyces cerevisiae (strain JAY291) OX=57496...	C7GWZ2|KEX1_YEAS2 (+2)	83 kDa		19
114	☑	☆	Acetolactate synthase catalytic subunit, mitochondrial OS=Saccharomyces cerevisiae (strain ATCC 20450...	P07342|ILVB_YEAST	75 kDa		19
115	☑	☆	60S ribosomal protein L2-A OS=Saccharomyces cerevisiae (strain ATCC 204508 / S288c) OX=559292 GN=...	P0CX45|RL2A_YEAST (+2)	27 kDa		16
116	☑	☆	Cystathionine beta-synthase OS=Saccharomyces cerevisiae (strain ATCC 204508 / S288c) OX=559292 GN...	P32582|CBS_YEAST	56 kDa		19
117	☑	☆	6-phosphogluconate dehydrogenase, decarboxylating 1 OS=Saccharomyces cerevisiae (strain ATCC 2045...	P38720|6PGD1_YEAST	54 kDa		21
118	☑	☆	Protein SCP160 OS=Saccharomyces cerevisiae (strain ATCC 204508 / S288c) OX=559292 GN=SCP160 PE=...	P06105|SC160_YEAST	135 kDa		21
119	☑	☆	Protein S100-A9 OS=Homo sapiens OX=9606 GN=S100A9 PE=1 SV=1	P06702|S10A9_HUMAN	13 kDa		14
120	☑	☆	Cytochrome b-c1 complex subunit 2, mitochondrial OS=Saccharomyces cerevisiae (strain ATCC 204508 / ...	P07257|QCR2_YEAST	40 kDa		19
121	☑	☆	Dihydrolipoyllysine-residue succinyltransferase component of 2-oxoglutarate dehydrogenase complex, mi...	P19262|ODO2_YEAST	50 kDa		16
⊞ 122	☑	☆	Cluster of S-adenosylmethionine synthase 2 OS=Saccharomyces cerevisiae (strain ATCC 204508 / S288c) ...	P19358|METK2_YEAST [2]	42 kDa	★	16
123	☑	☆	Leucine--tRNA ligase, cytoplasmic OS=Saccharomyces cerevisiae (strain ATCC 204508 / S288c) OX=55929...	P26637|SYLC_YEAST	124 kDa	★	19
124	☑	☆	Phosphoglucomutase 2 OS=Saccharomyces cerevisiae (strain ATCC 204508 / S288c) OX=559292 GN=PGM...	P37012|PGM2_YEAST	63 kDa	★	18
125	☑	☆	Aminopeptidase Y OS=Saccharomyces cerevisiae (strain ATCC 204508 / S288c) OX=559292 GN=APE3 PE=...	P37302|APE3_YEAST	60 kDa		18
126	☑	☆	Ribosome-associated complex subunit SSZ1 OS=Saccharomyces cerevisiae (strain ATCC 204508 / S288c) ...	P38788|SSZ1_YEAST	58 kDa		20
⊞ 127	☑	☆	Cluster of High-affinity hexose transporter HXT7 OS=Saccharomyces cerevisiae (strain ATCC 204508 / S28...	P39004|HXT7_YEAST [4]	63 kDa	★	18
128	☑	☆	Isocitrate dehydrogenase [NADP] cytoplasmic OS=Saccharomyces cerevisiae (strain ATCC 204508 / S288...	P41939|IDHC_YEAST	47 kDa	★	21
129	☑	☆	Glutamate--tRNA ligase, cytoplasmic OS=Saccharomyces cerevisiae (strain ATCC 204508 / S288c) OX=55...	P46655|SYEC_YEAST	81 kDa		17
130	☑	☆	ATP-dependent RNA helicase eIF4A OS=Saccharomyces cerevisiae (strain YJM789) OX=307796 GN=TIF1 P...	A6ZQJ1|IF4A_YEAS7 (+1)	45 kDa		21
131	☑	☆	NADH-cytochrome b5 reductase 2 OS=Saccharomyces cerevisiae (strain YJM789) OX=307796 GN=MCR1 P...	A6ZZH2|MCR1_YEAS7 (+1)	34 kDa		16
132	☑	☆	40S ribosomal protein S0-B OS=Saccharomyces cerevisiae (strain YJM789) OX=307796 GN=RPS0B PE=3 S...	A7A0V3|RSSA2_YEAS7 (+2)	28 kDa		17
133	☑	☆	60S ribosomal protein L7-A OS=Saccharomyces cerevisiae (strain ATCC 204508 / S288c) OX=559292 GN=...	P05737|RL7A_YEAST (+1)	28 kDa	★	17
⊞ 134	☑	☆	Cluster of 60S ribosomal protein L8-A OS=Saccharomyces cerevisiae (strain ATCC 204508 / S288c) OX=55...	P17076|RL8A_YEAST [2]	28 kDa	★	17
⊞ 135	☑	☆	Cluster of Ubiquitin-activating enzyme E1 1 OS=Saccharomyces cerevisiae (strain ATCC 204508 / S288c) O...	P22515|UBA1_YEAST [2]	114 kDa	★	23
136	☑	☆	Protein EGT2 OS=Saccharomyces cerevisiae (strain ATCC 204508 / S288c) OX=559292 GN=EGT2 PE=2 SV=2	P42835|EGT2_YEAST	109 kDa		16
⊞ 137	☑	☆	Cluster of Inorganic pyrophosphatase OS=Saccharomyces cerevisiae (strain ATCC 204508 / S288c) OX=55...	P00817|IPYR_YEAST [2]	32 kDa	★	17
138	☑	☆	2-isopropylmalate synthase OS=Saccharomyces cerevisiae (strain ATCC 204508 / S288c) OX=559292 GN...	P06208|LEU1_YEAST	68 kDa		19
139	☑	☆	Glycerol 2-dehydrogenase (NADP(+)) OS=Saccharomyces cerevisiae (strain ATCC 204508 / S288c) OX=55...	P14065|GCY1_YEAST	35 kDa	★	17
140	☑	☆	Transaldolase OS=Saccharomyces cerevisiae (strain ATCC 204508 / S288c) OX=559292 GN=TAL1 PE=1 SV...	P15019|TAL1_YEAST	37 kDa	★	16
141	☑	☆	Pyruvate dehydrogenase E1 component subunit alpha, mitochondrial OS=Saccharomyces cerevisiae (strai...	P16387|ODPA_YEAST	46 kDa		17
142	☑	☆	Mitochondrial phosphate carrier protein OS=Saccharomyces cerevisiae (strain ATCC 204508 / S288c) OX=...	P23641|MPCP_YEAST	33 kDa		18
143	☑	☆	60S ribosomal protein L5 OS=Saccharomyces cerevisiae (strain ATCC 204508 / S288c) OX=559292 GN=RP...	P26321|RL5_YEAST	34 kDa		18
144	☑	☆	Elongation factor 1-gamma 2 OS=Saccharomyces cerevisiae (strain ATCC 204508 / S288c) OX=559292 GN...	P36008|EF1G2_YEAST	47 kDa	★	18
145	☑	☆	Glycine--tRNA ligase 1, mitochondrial OS=Saccharomyces cerevisiae (strain ATCC 204508 / S288c) OX=55...	P38088|SYG_YEAST	78 kDa		19
146	☑	☆	Eukaryotic translation initiation factor 3 subunit A OS=Saccharomyces cerevisiae (strain ATCC 204508 / S...	P38249|EIF3A_YEAST	110 kDa		18
⊞ 147	☑	☆	Cluster of Homocitrate synthase, cytosolic isozyme OS=Saccharomyces cerevisiae (strain ATCC 204508 / ...	P48570|HOSC_YEAST [2]	47 kDa	★	18
148	☑	☆	Sphingolipid long chain base-responsive protein LSP1 OS=Saccharomyces cerevisiae (strain ATCC 204508 ...	Q12230|LSP1_YEAST	38 kDa	★	16

Probability Legend

- over 95%
- 80% to 94%
- 50% to 79%
- 20% to 49%
- 0% to 19%

#	Visible?	Starred?	BioView: 1158 Proteins in 1038 Clusters With 5 Decoys and 22 Hidden	Accession Number	Molecular Weight	Protein Grouping Ambiguity	SrfA_C_CAT
149	☑	☆	Cluster of Cell wall mannoprotein PIR1 OS=Saccharomyces cerevisiae (strain YJM789) OX=307796 GN=PI...	A6ZZG0|PIR1_YEAS7 [9]	35 kDa	★	11
150	☑	☆	Sterol 24-C-methyltransferase OS=Saccharomyces cerevisiae (strain ATCC 204508 / S288c) OX=559292 G...	P25087|ERG6_YEAST	43 kDa		15
151	☑	☆	Acetyl-CoA hydrolase OS=Saccharomyces cerevisiae (strain ATCC 204508 / S288c) OX=559292 GN=ACH1 ...	P32316|ACH1_YEAST	59 kDa		17
152	☑	☆	Mannose-1-phosphate guanyltransferase OS=Saccharomyces cerevisiae (strain ATCC 204508 / S288c) OX...	P41940|MPG1_YEAST	40 kDa		16
153	☑	☆	Succinate--CoA ligase [ADP-forming] subunit beta, mitochondrial OS=Saccharomyces cerevisiae (strain A...	P53312|SUCB_YEAST	47 kDa		14
154	☑	☆	Transcriptional modulator WTM1 OS=Saccharomyces cerevisiae (strain ATCC 204508 / S288c) OX=559292...	Q12363|WTM1_YEAST	48 kDa		15
155	☑	☆	Polyadenylate-binding protein, cytoplasmic and nuclear OS=Saccharomyces cerevisiae (strain ATCC 2045...	P04147|PABP_YEAST	64 kDa		17
156	☑	☆	Cluster of 60S ribosomal protein L6-B OS=Saccharomyces cerevisiae (strain ATCC 204508 / S288c) OX=55...	P05739|RL6B_YEAST [2]	20 kDa	★	16
157	☑	☆	Fumarate hydratase, mitochondrial OS=Saccharomyces cerevisiae (strain ATCC 204508 / S288c) OX=559...	P08417|FUMH_YEAST	53 kDa		17
158	☑	☆	Dihydrolipoyl dehydrogenase, mitochondrial OS=Saccharomyces cerevisiae (strain ATCC 204508 / S288c) ...	P09624|DLDH_YEAST	54 kDa		15
159	☑	☆	40S ribosomal protein S4-A OS=Saccharomyces cerevisiae (strain ATCC 204508 / S288c) OX=559292 GN=...	P0CX35|RS4A_YEAST (+1)	29 kDa		15
160	☑	☆	40S ribosomal protein S5 OS=Saccharomyces cerevisiae (strain ATCC 204508 / S288c) OX=559292 GN=RP...	P26783|RS5_YEAST	25 kDa		14
161	☑	☆	Homoserine dehydrogenase OS=Saccharomyces cerevisiae (strain ATCC 204508 / S288c) OX=559292 GN...	P31116|DHOM_YEAST	39 kDa		15
162	☑	☆	26S proteasome regulatory subunit RPN2 OS=Saccharomyces cerevisiae (strain ATCC 204508 / S288c) OX...	P32565|RPN2_YEAST	104 kDa		18
163	☑	☆	Carnitine O-acetyltransferase, mitochondrial OS=Saccharomyces cerevisiae (strain ATCC 204508 / S288c)...	P32796|CACP_YEAST	77 kDa		18
164	☑	☆	Nuclear segregation protein BFR1 OS=Saccharomyces cerevisiae (strain ATCC 204508 / S288c) OX=55929...	P38934|BFR1_YEAST	55 kDa		17
165	☑	☆	Cluster of Alanine--tRNA ligase, mitochondrial OS=Saccharomyces cerevisiae (strain ATCC 204508 / S288c...	P40825|SYA_YEAST [2]	110 kDa	★	18
166	☑	☆	60S ribosomal protein L10 OS=Saccharomyces cerevisiae (strain ATCC 204508 / S288c) OX=559292 GN=R...	P41805|RL10_YEAST	25 kDa		13
167	☑	☆	Alanine--glyoxylate aminotransferase 1 OS=Saccharomyces cerevisiae (strain ATCC 204508 / S288c) OX=...	P43567|AGX1_YEAST	42 kDa		15
168	☑	☆	Arginine--tRNA ligase, cytoplasmic OS=Saccharomyces cerevisiae (strain ATCC 204508 / S288c) OX=5592...	Q05506|SYRC_YEAST	70 kDa		18
169	☑	☆	NADPH-dependent alpha-keto amide reductase OS=Saccharomyces cerevisiae (strain ATCC 204508 / S28...	Q07551|KAR_YEAST	36 kDa		16
170	☑	☆	Cell wall protein ECM33 OS=Saccharomyces cerevisiae (strain YJM789) OX=307796 GN=ECM33 PE=3 SV=2	A6ZL22|ECM33_YEAS7 (+2)	44 kDa	★	14
171	☑	☆	Carboxypeptidase Y OS=Saccharomyces cerevisiae (strain ATCC 204508 / S288c) OX=559292 GN=PRC1 PE...	P00729|CBPY_YEAST	60 kDa		14
172	☑	☆	Alcohol dehydrogenase 3, mitochondrial OS=Saccharomyces cerevisiae (strain ATCC 204508 / S288c) OX=...	P07246|ADH3_YEAST	40 kDa	★	14
173	☑	☆	Ribonucleoside-diphosphate reductase small chain 1 OS=Saccharomyces cerevisiae (strain ATCC 204508 /...	P09938|RIR2_YEAST	46 kDa		15
174	☑	☆	Peptidyl-prolyl cis-trans isomerase OS=Saccharomyces cerevisiae (strain ATCC 204508 / S288c) OX=5592...	P14832|CYPH_YEAST	17 kDa	★	13
175	☑	☆	Heat shock protein STI1 OS=Saccharomyces cerevisiae (strain ATCC 204508 / S288c) OX=559292 GN=STI...	P15705|STI1_YEAST	66 kDa		15
176	☑	☆	Phosphoribosylaminoimidazole carboxylase OS=Saccharomyces cerevisiae (strain ATCC 204508 / S288c) ...	P21264|PUR6_YEAST	62 kDa		15
177	☑	☆	Dihydroxy-acid dehydratase, mitochondrial OS=Saccharomyces cerevisiae (strain ATCC 204508 / S288c) ...	P39522|ILV3_YEAST	63 kDa		13
178	☑	☆	Sphingolipid long chain base-responsive protein PIL1 OS=Saccharomyces cerevisiae (strain ATCC 204508 ...	P53252|PIL1_YEAST	38 kDa	★	15
179	☑	☆	Succinate dehydrogenase [ubiquinone] flavoprotein subunit, mitochondrial OS=Saccharomyces cerevisiae...	Q00711|SDHA_YEAST	70 kDa		14
180	☑	☆	Tryptophan synthase OS=Saccharomyces cerevisiae (strain ATCC 204508 / S288c) OX=559292 GN=TRP5 ...	P00931|TRP_YEAST	77 kDa		15
181	☑	☆	Tubulin beta chain OS=Saccharomyces cerevisiae (strain ATCC 204508 / S288c) OX=559292 GN=TUB2 PE=...	P02557|TBB_YEAST	51 kDa		15
182	☑	☆	Carbamoyl-phosphate synthase arginine-specific large chain OS=Saccharomyces cerevisiae (strain ATCC ...	P03965|CARB_YEAST	124 kDa	★	15
183	☑	☆	Mitochondrial outer membrane protein OM45 OS=Saccharomyces cerevisiae (strain ATCC 204508 / S288c...	P16547|OM45_YEAST	45 kDa		14
184	☑	☆	Phosphoribosylaminoimidazole-succinocarboxamide synthase OS=Saccharomyces cerevisiae (strain ATC...	P27616|PUR7_YEAST	35 kDa		15
185	☑	☆	Rotenone-insensitive NADH-ubiquinone oxidoreductase, mitochondrial OS=Saccharomyces cerevisiae (str...	P32340|NDI1_YEAST	57 kDa		15

Probability Legend

- over 95%
- 80% to 94%
- 50% to 79%
- 20% to 49%
- 0% to 19%

#	Visible?	Starred?	BioView: 1158 Proteins in 1038 Clusters With 5 Decoys and 22 Hidden	Accession Number	Molecular Weight	Protein Grouping Ambiguity	SrfA_C_CAT
186	☑	☆	Protein TOS1 OS=Saccharomyces cerevisiae (strain ATCC 204508 / S288c) OX=559292 GN=TOS1 PE=1 SV=1	P38288|TOS1_YEAST	48 kDa		11
187	☑	☆	60S ribosomal protein L13-B OS=Saccharomyces cerevisiae (strain ATCC 204508 / S288c) OX=559292 GN...	P40212|RL13B_YEAST (+1)	23 kDa		11
188	☑	☆	Hydroxymethylglutaryl-CoA synthase OS=Saccharomyces cerevisiae (strain ATCC 204508 / S288c) OX=5...	P54839|HMCS_YEAST	55 kDa		15
189	☑	☆	Protein ARG5,6, mitochondrial OS=Saccharomyces cerevisiae (strain ATCC 204508 / S288c) OX=559292 G...	Q01217|ARG56_YEAST	95 kDa		15
190	☑	☆	Transposon Ty1-LR1 Gag-Pol polyprotein OS=Saccharomyces cerevisiae (strain ATCC 204508 / S288c) OX...	Q12088|YL11B_YEAST (+1)	198 kDa		13
191	☑	☆	Probable 2-methylcitrate dehydratase OS=Saccharomyces cerevisiae (strain ATCC 204508 / S288c) OX=5...	Q12428|PRPD_YEAST	58 kDa		14
192	☑	☆	Nucleolar protein 56 OS=Saccharomyces cerevisiae (strain ATCC 204508 / S288c) OX=559292 GN=NOP56 ...	Q12460|NOP56_YEAST	57 kDa	★	14
193	☑	☆	Cysteine proteinase 1, mitochondrial OS=Saccharomyces cerevisiae (strain YJM789) OX=307796 GN=LAP...	A6ZRK4|BLH1_YEAS7 (+4)	56 kDa		13
⊞ 194	☑	☆	Cluster of Adenylosuccinate synthetase OS=Saccharomyces cerevisiae (strain YJM789) OX=307796 GN=A...	A6ZRM0|PURA_YEAS7 [7]	48 kDa	★	15
195	☑	☆	Mitochondrial import receptor subunit TOM70 OS=Saccharomyces cerevisiae (strain YJM789) OX=307796 ...	A6ZRW3|TOM70_YEAS7 (+1)	70 kDa		13
196	☑	☆	Malate synthase 1 OS=Saccharomyces cerevisiae (strain YJM789) OX=307796 GN=MLS1 PE=3 SV=1	A6ZRW6|MLS1_YEAS7 (+1)	63 kDa		13
197	☑	☆	Cell wall mannoprotein PST1 OS=Saccharomyces cerevisiae (strain YJM789) OX=307796 GN=PST1 PE=3 S...	A6ZY20|PST1_YEAS7 (+1)	46 kDa	★	13
198	☑	☆	60S acidic ribosomal protein P2-beta OS=Saccharomyces cerevisiae (strain ATCC 204508 / S288c) OX=55...	P02400|RLA4_YEAST	11 kDa		9
199	☑	☆	60S ribosomal protein L20-A OS=Saccharomyces cerevisiae (strain ATCC 204508 / S288c) OX=559292 GN...	P0CX23|RL20A_YEAST (+1)	20 kDa		9
200	☑	☆	40S ribosomal protein S18-A OS=Saccharomyces cerevisiae (strain ATCC 204508 / S288c) OX=559292 GN...	P0CX55|RS18A_YEAST (+1)	17 kDa		8
201	☑	☆	Aspartate-semialdehyde dehydrogenase OS=Saccharomyces cerevisiae (strain ATCC 204508 / S288c) OX...	P13663|DHAS_YEAST	40 kDa		13
202	☑	☆	Junction plakoglobin OS=Homo sapiens OX=9606 GN=JUP PE=1 SV=3	P14923|PLAK_HUMAN	82 kDa		14
203	☑	☆	Vacuolar protein sorting-associated protein 1 OS=Saccharomyces cerevisiae (strain ATCC 204508 / S288c...	P21576|VPS1_YEAST	79 kDa		15
204	☑	☆	Isocitrate dehydrogenase [NADP], mitochondrial OS=Saccharomyces cerevisiae (strain ATCC 204508 / S2...	P21954|IDHP_YEAST	48 kDa	★	14
205	☑	☆	Isocitrate lyase OS=Saccharomyces cerevisiae (strain ATCC 204508 / S288c) OX=559292 GN=ICL1 PE=1 S...	P28240|ACEA_YEAST	62 kDa		14
206	☑	☆	Isocitrate dehydrogenase [NAD] subunit 1, mitochondrial OS=Saccharomyces cerevisiae (strain ATCC 204...	P28834|IDH1_YEAST	39 kDa		11
⊞ 207	☑	☆	Cluster of Pyruvate dehydrogenase E1 component subunit beta, mitochondrial OS=Saccharomyces cerevi...	P32473|ODPB_YEAST	40 kDa	★	15
208	☑	☆	GMP synthase [glutamine-hydrolyzing] OS=Saccharomyces cerevisiae (strain ATCC 204508 / S288c) OX=...	P38625|GUAA_YEAST	58 kDa		15
209	☑	☆	40S ribosomal protein S12 OS=Saccharomyces cerevisiae (strain ATCC 204508 / S288c) OX=559292 GN=R...	P48589|RS12_YEAST	15 kDa		11
210	☑	☆	Aromatic/aminoadipate aminotransferase 1 OS=Saccharomyces cerevisiae (strain ATCC 204508 / S288c) ...	P53090|ARO8_YEAST	56 kDa		14
211	☑	☆	Coatomer subunit alpha OS=Saccharomyces cerevisiae (strain ATCC 204508 / S288c) OX=559292 GN=COP...	P53622|COPA_YEAST	136 kDa		16
212	☑	☆	Peroxisomal hydratase-dehydrogenase-epimerase OS=Saccharomyces cerevisiae (strain ATCC 204508 / ...	Q02207|FOX2_YEAST	99 kDa		15
213	☑	☆	Probable family 17 glucosidase SCW10 OS=Saccharomyces cerevisiae (strain ATCC 204508 / S288c) OX=5...	Q04951|SCW10_YEAST	40 kDa	★	11
214	☑	☆	Nucleolar protein 58 OS=Saccharomyces cerevisiae (strain YJM789) OX=307796 GN=NOP58 PE=3 SV=1	A6ZPE5|NOP58_YEAS7 (+1)	57 kDa	★	12
215	☑	☆	60S acidic ribosomal protein P0 OS=Saccharomyces cerevisiae (strain ATCC 204508 / S288c) OX=559292 ...	P05317|RLA0_YEAST	34 kDa		12
216	☑	☆	Glycogen phosphorylase OS=Saccharomyces cerevisiae (strain ATCC 204508 / S288c) OX=559292 GN=GP...	P06738|PHSG_YEAST	103 kDa		14
217	☑	☆	3-isopropylmalate dehydratase OS=Saccharomyces cerevisiae (strain ATCC 204508 / S288c) OX=559292 ...	P07264|LEUC_YEAST	86 kDa		14
218	☑	☆	60S ribosomal protein L27-A OS=Saccharomyces cerevisiae (strain ATCC 204508 / S288c) OX=559292 GN...	P0C2H6|RL27A_YEAST (+1)	16 kDa		9
219	☑	☆	Actin-binding protein OS=Saccharomyces cerevisiae (strain ATCC 204508 / S288c) OX=559292 GN=ABP1 P...	P15891|ABP1_YEAST	66 kDa		13
220	☑	☆	Iron transport multicopper oxidase FET3 OS=Saccharomyces cerevisiae (strain ATCC 204508 / S288c) OX...	P38993|FET3_YEAST	72 kDa	★	14
221	☑	☆	Suppressor protein STM1 OS=Saccharomyces cerevisiae (strain ATCC 204508 / S288c) OX=559292 GN=ST...	P39015|STM1_YEAST	30 kDa		11
222	☑	☆	Protein GVP36 OS=Saccharomyces cerevisiae (strain ATCC 204508 / S288c) OX=559292 GN=GVP36 PE=1 S...	P40531|GVP36_YEAST	37 kDa		13

Probability Legend
- over 95%
- 80% to 94%
- 50% to 79%
- 20% to 49%
- 0% to 19%

#	Visible?	Starred?	BioView: 1158 Proteins in 1038 Clusters With 5 Decoys and 22 Hidden	Accession Number	Molecular Weight	Protein Grouping Ambiguity	SrfA_C_CAT
223	☑	☆	Ribonucleoside-diphosphate reductase small chain 2 OS=Saccharomyces cerevisiae (strain ATCC 204508 /...	P49723|RIR4_YEAST	40 kDa		13
224	☑	☆	Cluster of ATP-dependent RNA helicase DED1 OS=Saccharomyces cerevisiae (strain YJM789) OX=307796 ...	A6ZP47|DED1_YEAS7 [3]	66 kDa	★	12
225	☑	☆	Cytochrome b2, mitochondrial OS=Saccharomyces cerevisiae (strain ATCC 204508 / S288c) OX=559292 G...	P00175|CYB2_YEAST	66 kDa		11
226	☑	☆	60S ribosomal protein L17-A OS=Saccharomyces cerevisiae (strain ATCC 204508 / S288c) OX=559292 GN...	P05740|RL17A_YEAST	21 kDa		8
227	☑	☆	Farnesyl pyrophosphate synthase OS=Saccharomyces cerevisiae (strain ATCC 204508 / S288c) OX=55929...	P08524|FPPS_YEAST	40 kDa		12
228	☑	☆	Dihydrolipoyllysine-residue acetyltransferase component of pyruvate dehydrogenase complex, mitochon...	P12695|ODP2_YEAST	52 kDa		12
229	☑	☆	Acyl-coenzyme A oxidase OS=Saccharomyces cerevisiae (strain ATCC 204508 / S288c) OX=559292 GN=P...	P13711|ACOX_YEAST	84 kDa		12
230	☑	☆	Lysine--tRNA ligase, cytoplasmic OS=Saccharomyces cerevisiae (strain ATCC 204508 / S288c) OX=559292...	P15180|SYKC_YEAST	68 kDa		12
231	☑	☆	Threonine synthase OS=Saccharomyces cerevisiae (strain ATCC 204508 / S288c) OX=559292 GN=THR4 PE...	P16120|THRC_YEAST	57 kDa		13
232	☑	☆	Aspartate aminotransferase, cytoplasmic OS=Saccharomyces cerevisiae (strain ATCC 204508 / S288c) OX...	P23542|AATC_YEAST	46 kDa		11
233	☑	☆	Cell wall protein CWP1 OS=Saccharomyces cerevisiae (strain ATCC 204508 / S288c) OX=559292 GN=CWP1...	P28319|CWP1_YEAST	24 kDa		11
234	☑	☆	Endochitinase OS=Saccharomyces cerevisiae (strain ATCC 204508 / S288c) OX=559292 GN=CTS1 PE=1 SV...	P29029|CHIT_YEAST	59 kDa		12
235	☑	☆	Elongation factor 1-gamma 1 OS=Saccharomyces cerevisiae (strain ATCC 204508 / S288c) OX=559292 GN...	P29547|EF1G1_YEAST	47 kDa	★	13
236	☑	☆	Long-chain-fatty-acid--CoA ligase 1 OS=Saccharomyces cerevisiae (strain ATCC 204508 / S288c) OX=559...	P30624|LCF1_YEAST	78 kDa		13
237	☑	☆	Malate dehydrogenase, peroxisomal OS=Saccharomyces cerevisiae (strain ATCC 204508 / S288c) OX=559...	P32419|MDHP_YEAST	37 kDa		12
238	☑	☆	Aminopeptidase 2, mitochondrial OS=Saccharomyces cerevisiae (strain ATCC 204508 / S288c) OX=55929...	P32454|APE2_YEAST	108 kDa	★	13
239	☑	☆	V-type proton ATPase subunit a, vacuolar isoform OS=Saccharomyces cerevisiae (strain ATCC 204508 / S...	P32563|VPH1_YEAST	96 kDa		13
240	☑	☆	Fimbrin OS=Saccharomyces cerevisiae (strain ATCC 204508 / S288c) OX=559292 GN=SAC6 PE=1 SV=1	P32599|FIMB_YEAST	72 kDa		13
241	☑	☆	Nucleoside diphosphate kinase OS=Saccharomyces cerevisiae (strain ATCC 204508 / S288c) OX=559292 G...	P36010|NDK_YEAST	17 kDa		9
242	☑	☆	Serine hydroxymethyltransferase, mitochondrial OS=Saccharomyces cerevisiae (strain ATCC 204508 / S2...	P37292|GLYM_YEAST	54 kDa	★	12
243	☑	☆	ATP synthase subunit gamma, mitochondrial OS=Saccharomyces cerevisiae (strain ATCC 204508 / S288c)...	P38077|ATPG_YEAST	34 kDa		11
244	☑	☆	Obg-like ATPase 1 OS=Saccharomyces cerevisiae (strain ATCC 204508 / S288c) OX=559292 GN=OLA1 PE=...	P38219|OLA1_YEAST	44 kDa		12
245	☑	☆	Asparagine--tRNA ligase, cytoplasmic OS=Saccharomyces cerevisiae (strain ATCC 204508 / S288c) OX=55...	P38707|SYNC_YEAST	62 kDa		13
246	☑	☆	Coatomer subunit beta OS=Saccharomyces cerevisiae (strain ATCC 204508 / S288c) OX=559292 GN=SEC2...	P41810|COPB_YEAST	109 kDa		12
247	☑	☆	Cys-Gly metallodipeptidase DUG1 OS=Saccharomyces cerevisiae (strain ATCC 204508 / S288c) OX=55929...	P43616|DUG1_YEAST	53 kDa		11
248	☑	☆	Adenosine kinase OS=Saccharomyces cerevisiae (strain ATCC 204508 / S288c) OX=559292 GN=ADO1 PE=...	P47143|ADK_YEAST	36 kDa		11
249	☑	☆	60S ribosomal protein L15-B OS=Saccharomyces cerevisiae (strain ATCC 204508 / S288c) OX=559292 GN...	P54780|RL15B_YEAST	24 kDa		7
250	☑	☆	Elongation factor Tu, mitochondrial OS=Saccharomyces cerevisiae (strain ATCC 204508 / S288c) OX=5592...	P02992|EFTU_YEAST	48 kDa		11
251	☑	☆	ATP synthase subunit 4, mitochondrial OS=Saccharomyces cerevisiae (strain ATCC 204508 / S288c) OX=5...	P05626|ATPF_YEAST	27 kDa		10
252	☑	☆	Cluster of 40S ribosomal protein S14-A OS=Saccharomyces cerevisiae (strain ATCC 204508 / S288c) OX=5...	P06367|RS14A_YEAST [6]	15 kDa	★	10
253	☑	☆	ATP synthase subunit 5, mitochondrial OS=Saccharomyces cerevisiae (strain ATCC 204508 / S288c) OX=5...	P09457|ATPO_YEAST	23 kDa		10
254	☑	☆	Cluster of 40S ribosomal protein S22-A OS=Saccharomyces cerevisiae (strain ATCC 204508 / S288c) OX=5...	P0C0W1|RS22A_YEAST [6]	15 kDa	★	7
255	☑	☆	Glutamine--tRNA ligase OS=Saccharomyces cerevisiae (strain ATCC 204508 / S288c) OX=559292 GN=GLN...	P13188|SYQ_YEAST	93 kDa		11
256	☑	☆	Sodium transport ATPase 1 OS=Saccharomyces cerevisiae (strain ATCC 204508 / S288c) OX=559292 GN=...	P13587|ATN1_YEAST	120 kDa		11
257	☑	☆	Dolichol-phosphate mannosyltransferase OS=Saccharomyces cerevisiae (strain ATCC 204508 / S288c) OX...	P14020|DPM1_YEAST	30 kDa		11
258	☑	☆	Phenylalanine--tRNA ligase beta subunit OS=Saccharomyces cerevisiae (strain ATCC 204508 / S288c) OX=...	P15624|SYFB_YEAST	67 kDa		12
259	☑	☆	Flavoprotein-like protein YCP4 OS=Saccharomyces cerevisiae (strain ATCC 204508 / S288c) OX=559292 G...	P25349|YCP4_YEAST	26 kDa		10

Probability Legend: over 95% | 80% to 94% | 50% to 79% | 20% to 49% | 0% to 19%

#	Visible?	Starred?	BioView: 1158 Proteins in 1038 Clusters With 5 Decoys and 22 Hidden	Accession Number	Molecular Weight	Protein Grouping Ambiguity	SrfA_C_CAT
260	☑	☆	Succinate/fumarate mitochondrial transporter OS=Saccharomyces cerevisiae (strain ATCC 204508 / S288...	P33303|SFC1_YEAST	35 kDa		11
261	☑	☆	eIF-2-alpha kinase activator GCN1 OS=Saccharomyces cerevisiae (strain ATCC 204508 / S288c) OX=5592...	P33892|GCN1_YEAST	297 kDa		12
262	☑	☆	EH domain-containing and endocytosis protein 1 OS=Saccharomyces cerevisiae (strain ATCC 204508 / S28...	P34216|EDE1_YEAST	151 kDa		11
263	☑	☆	D-arabinose dehydrogenase [NAD(P)+] heavy chain OS=Saccharomyces cerevisiae (strain ATCC 204508 / ...	P38115|ARA1_YEAST	39 kDa		11
264	☑	☆	26S proteasome regulatory subunit RPN1 OS=Saccharomyces cerevisiae (strain ATCC 204508 / S288c) OX...	P38764|RPN1_YEAST	109 kDa		10
265	☑	☆	Eukaryotic translation initiation factor 5B OS=Saccharomyces cerevisiae (strain ATCC 204508 / S288c) OX...	P39730|IF2P_YEAST	112 kDa		12
266	☑	☆	Acetyl-CoA acetyltransferase OS=Saccharomyces cerevisiae (strain ATCC 204508 / S288c) OX=559292 GN...	P41338|THIL_YEAST	42 kDa		11
267	☑	☆	Coatomer subunit beta' OS=Saccharomyces cerevisiae (strain ATCC 204508 / S288c) OX=559292 GN=SEC...	P41811|COPB2_YEAST	99 kDa		11
268	☑	☆	40S ribosomal protein S10-B OS=Saccharomyces cerevisiae (strain ATCC 204508 / S288c) OX=559292 GN...	P46784|RS10B_YEAST (+1)	13 kDa		10
269	☑	☆	NADPH dehydrogenase 2 OS=Saccharomyces cerevisiae (strain ATCC 204508 / S288c) OX=559292 GN=OY...	Q03558|OYE2_YEAST	45 kDa		10
270	☑	☆	Mitochondrial distribution and morphology protein 38 OS=Saccharomyces cerevisiae (strain ATCC 204508 ...	Q08179|MDM38_YEAST	65 kDa		9
271	☑	☆	Protoplast secreted protein 2 OS=Saccharomyces cerevisiae (strain ATCC 204508 / S288c) OX=559292 GN...	Q12335|PST2_YEAST	21 kDa		9
272	☑	☆	Formate dehydrogenase 1 OS=Saccharomyces cerevisiae (strain YJM789) OX=307796 GN=FDH1 PE=2 SV=1	A6ZN46|FDH1_YEAS7 (+3)	42 kDa		10
273	☑	☆	Superoxide dismutase [Cu-Zn] OS=Saccharomyces cerevisiae (strain ATCC 204508 / S288c) OX=559292 G...	P00445|SODC_YEAST	16 kDa		8
⊞ 274	☑	☆	Cluster of 60S ribosomal protein L9-A OS=Saccharomyces cerevisiae (strain ATCC 204508 / S288c) OX=55...	P05738|RL9A_YEAST [2]	22 kDa	★	8
275	☑	☆	40S ribosomal protein S9-B OS=Saccharomyces cerevisiae (strain ATCC 204508 / S288c) OX=559292 GN=...	P05755|RS9B_YEAST	22 kDa		9
276	☑	☆	Serine--tRNA ligase, cytoplasmic OS=Saccharomyces cerevisiae (strain ATCC 204508 / S288c) OX=559292...	P07284|SYSC_YEAST	53 kDa		11
⊞ 277	☑	☆	Cluster of 40S ribosomal protein S6-A OS=Saccharomyces cerevisiae (strain ATCC 204508 / S288c) OX=55...	P0CX37|RS6A_YEAST [3]	27 kDa	★	10
278	☑	☆	Vacuolar aminopeptidase 1 OS=Saccharomyces cerevisiae (strain ATCC 204508 / S288c) OX=559292 GN=...	P14904|AMPL_YEAST	57 kDa		11
279	☑	☆	Tropomyosin-1 OS=Saccharomyces cerevisiae (strain ATCC 204508 / S288c) OX=559292 GN=TPM1 PE=1 ...	P17536|TPM1_YEAST	24 kDa		9
280	☑	☆	V-type proton ATPase subunit E OS=Saccharomyces cerevisiae (strain ATCC 204508 / S288c) OX=559292 ...	P22203|VATE_YEAST	26 kDa		9
281	☑	☆	Phosphatidylinositol-3-phosphatase SAC1 OS=Saccharomyces cerevisiae (strain ATCC 204508 / S288c) OX...	P32368|SAC1_YEAST	71 kDa		11
282	☑	☆	Elongation factor 1-beta OS=Saccharomyces cerevisiae (strain ATCC 204508 / S288c) OX=559292 GN=EFB...	P32471|EF1B_YEAST	23 kDa		10
283	☑	☆	Zinc-regulated transporter 1 OS=Saccharomyces cerevisiae (strain ATCC 204508 / S288c) OX=559292 GN...	P32804|ZRT1_YEAST	42 kDa		8
284	☑	☆	UTP--glucose-1-phosphate uridylyltransferase OS=Saccharomyces cerevisiae (strain ATCC 204508 / S288...	P32861|UGPA1_YEAST	56 kDa		11
285	☑	☆	D-lactate dehydrogenase [cytochrome] 1, mitochondrial OS=Saccharomyces cerevisiae (strain ATCC 2045...	P32891|DLD1_YEAST	65 kDa		10
286	☑	☆	Saccharopine dehydrogenase [NAD(+), L-lysine-forming] OS=Saccharomyces cerevisiae (strain ATCC 204...	P38998|LYS1_YEAST	41 kDa		9
287	☑	☆	T-complex protein 1 subunit gamma OS=Saccharomyces cerevisiae (strain ATCC 204508 / S288c) OX=559...	P39077|TCPG_YEAST	59 kDa		10
288	☑	☆	Vesicle-associated membrane protein-associated protein SCS2 OS=Saccharomyces cerevisiae (strain ATC...	P40075|SCS2_YEAST	27 kDa		9
289	☑	☆	Homoisocitrate dehydrogenase, mitochondrial OS=Saccharomyces cerevisiae (strain ATCC 204508 / S288...	P40495|LYS12_YEAST	40 kDa		11
290	☑	☆	V-type proton ATPase subunit H OS=Saccharomyces cerevisiae (strain ATCC 204508 / S288c) OX=559292 ...	P41807|VATH_YEAST	54 kDa		10
⊞ 291	☑	☆	Cluster of T-complex protein 1 subunit theta OS=Saccharomyces cerevisiae (strain ATCC 204508 / S288c) ...	P47079|TCPQ_YEAST [2]	62 kDa	★	11
292	☑	☆	Branched-chain-amino-acid aminotransferase, cytosolic OS=Saccharomyces cerevisiae (strain ATCC 2045...	P47176|BCA2_YEAST	42 kDa	★	10
293	☑	☆	Aminomethyltransferase, mitochondrial OS=Saccharomyces cerevisiae (strain ATCC 204508 / S288c) OX=...	P48015|GCST_YEAST	44 kDa		8
294	☑	☆	Succinate--CoA ligase [ADP-forming] subunit alpha, mitochondrial OS=Saccharomyces cerevisiae (strain A...	P53598|SUCA_YEAST	35 kDa		10
295	☑	☆	Dihydroxyacetone kinase 1 OS=Saccharomyces cerevisiae (strain ATCC 204508 / S288c) OX=559292 GN=...	P54838|DAK1_YEAST	62 kDa		11
296	☑	☆	Alpha,alpha-trehalose-phosphate synthase [UDP-forming] 56 kDa subunit OS=Saccharomyces cerevisiae ...	Q00764|TPS1_YEAST	56 kDa		10

Probability Legend

- over 95%
- 80% to 94%
- 50% to 79%
- 20% to 49%
- 0% to 19%

#	Visible?	Starred?	BioView: 1158 Proteins in 1038 Clusters With 5 Decoys and 22 Hidden	Accession Number	Molecular Weight	Protein Grouping Ambiguity	SrfA_C_CAT
297			Desmoglein-1 OS=Homo sapiens OX=9606 GN=DSG1 PE=1 SV=2	Q02413|DSG1_HUMAN	114 kDa		11
298			Cluster of 60S ribosomal protein L21-A OS=Saccharomyces cerevisiae (strain ATCC 204508 / S288c) OX=5...	Q02753|RL21A_YEAST [2]	18 kDa	★	9
299			Putative glucokinase-2 OS=Saccharomyces cerevisiae (strain ATCC 204508 / S288c) OX=559292 GN=EMI2...	Q04409|EMI2_YEAST	56 kDa	★	10
300			Eukaryotic translation initiation factor 3 subunit B OS=Saccharomyces cerevisiae (strain YJM789) OX=307...	A6ZPJ1|EIF3B_YEAS7 (+1)	88 kDa		11
301			Alcohol dehydrogenase 4 OS=Saccharomyces cerevisiae (strain YJM789) OX=307796 GN=ADH4 PE=3 SV=2	A6ZTT5|ADH4_YEAS7 (+1)	41 kDa		8
302			Adenylate kinase OS=Saccharomyces cerevisiae (strain YJM789) OX=307796 GN=ADK1 PE=3 SV=1	A6ZYI0|KAD2_YEAS7 (+2)	24 kDa		9
303			MICOS complex subunit MIC60 OS=Saccharomyces cerevisiae (strain YJM789) OX=307796 GN=MIC60 PE=...	A6ZZY0|MIC60_YEAS7 (+4)	61 kDa		10
304			Cluster of 60S ribosomal protein L36-B OS=Saccharomyces cerevisiae (strain ATCC 204508 / S288c) OX=5...	O14455|RL36B_YEAST [2]	11 kDa	★	7
305			Cytochrome b-c1 complex subunit 7, mitochondrial OS=Saccharomyces cerevisiae (strain ATCC 204508 / ...	P00128|QCR7_YEAST	15 kDa		8
306			Amidophosphoribosyltransferase OS=Saccharomyces cerevisiae (strain ATCC 204508 / S288c) OX=55929...	P04046|PUR1_YEAST	57 kDa		10
307			60S ribosomal protein L25 OS=Saccharomyces cerevisiae (strain ATCC 204508 / S288c) OX=559292 GN=R...	P04456|RL25_YEAST	16 kDa		5
308			Threonine--tRNA ligase, cytoplasmic OS=Saccharomyces cerevisiae (strain ATCC 204508 / S288c) OX=559...	P04801|SYTC_YEAST	85 kDa		10
309			60S acidic ribosomal protein P2-alpha OS=Saccharomyces cerevisiae (strain ATCC 204508 / S288c) OX=55...	P05319|RLA2_YEAST	11 kDa		8
310			Cytochrome c1, heme protein, mitochondrial OS=Saccharomyces cerevisiae (strain ATCC 204508 / S288c) ...	P07143|CY1_YEAST	34 kDa		8
311			Eukaryotic translation initiation factor 4E OS=Saccharomyces cerevisiae (strain ATCC 204508 / S288c) OX...	P07260|IF4E_YEAST	24 kDa		8
312			Cluster of 40S ribosomal protein S16-A OS=Saccharomyces cerevisiae (strain ATCC 204508 / S288c) OX=5...	P0CX51|RS16A_YEAST [4]	16 kDa	★	8
313			Heat shock protein 26 OS=Saccharomyces cerevisiae (strain ATCC 204508 / S288c) OX=559292 GN=HSP2...	P15992|HSP26_YEAST	24 kDa		9
314			NADPH--cytochrome P450 reductase OS=Saccharomyces cerevisiae (strain ATCC 204508 / S288c) OX=55...	P16603|NCPR_YEAST	77 kDa		10
315			Succinate dehydrogenase [ubiquinone] iron-sulfur subunit, mitochondrial OS=Saccharomyces cerevisiae (...	P21801|SDHB_YEAST	30 kDa		8
316			Mitochondrial protein import protein MAS5 OS=Saccharomyces cerevisiae (strain ATCC 204508 / S288c) O...	P25491|MAS5_YEAST	45 kDa		9
317			Carboxypeptidase S OS=Saccharomyces cerevisiae (strain ATCC 204508 / S288c) OX=559292 GN=CPS1 PE...	P27614|CBPS_YEAST	65 kDa		10
318			Isocitrate dehydrogenase [NAD] subunit 2, mitochondrial OS=Saccharomyces cerevisiae (strain ATCC 204...	P28241|IDH2_YEAST	40 kDa		10
319			Chorismate synthase OS=Saccharomyces cerevisiae (strain ATCC 204508 / S288c) OX=559292 GN=ARO2 ...	P28777|AROC_YEAST	41 kDa		9
320			Trehalose-phosphatase OS=Saccharomyces cerevisiae (strain ATCC 204508 / S288c) OX=559292 GN=TPS...	P31688|TPS2_YEAST	103 kDa		10
321			Coatomer subunit gamma OS=Saccharomyces cerevisiae (strain ATCC 204508 / S288c) OX=559292 GN=S...	P32074|COPG_YEAST	105 kDa		10
322			Glutamine synthetase OS=Saccharomyces cerevisiae (strain ATCC 204508 / S288c) OX=559292 GN=GLN1 ...	P32288|GLNA_YEAST	42 kDa		9
323			Phospho-2-dehydro-3-deoxyheptonate aldolase, tyrosine-inhibited OS=Saccharomyces cerevisiae (strain...	P32449|AROG_YEAST	40 kDa	★	8
324			Phosphoserine aminotransferase OS=Saccharomyces cerevisiae (strain ATCC 204508 / S288c) OX=55929...	P33330|SERC_YEAST	43 kDa		9
325			Protein PET10 OS=Saccharomyces cerevisiae (strain ATCC 204508 / S288c) OX=559292 GN=PET10 PE=1 S...	P36139|PET10_YEAST	31 kDa		10
326			Medium-chain fatty acid ethyl ester synthase/esterase 2 OS=Saccharomyces cerevisiae (strain ATCC 204...	P38295|MCFS2_YEAST	51 kDa		9
327			Putative proline--tRNA ligase YHR020W OS=Saccharomyces cerevisiae (strain ATCC 204508 / S288c) OX=...	P38708|YHI0_YEAST	77 kDa		10
328			Protein transport protein SEC31 OS=Saccharomyces cerevisiae (strain ATCC 204508 / S288c) OX=559292 ...	P38968|SEC31_YEAST	139 kDa		9
329			Eukaryotic initiation factor 4F subunit p150 OS=Saccharomyces cerevisiae (strain ATCC 204508 / S288c) O...	P39935|IF4F1_YEAST	107 kDa		8
330			Manganese-transporting ATPase 1 OS=Saccharomyces cerevisiae (strain ATCC 204508 / S288c) OX=5592...	P39986|ATC6_YEAST	135 kDa		9
331			Glutathione peroxidase-like peroxiredoxin HYR1 OS=Saccharomyces cerevisiae (strain ATCC 204508 / S28...	P40581|GPX3_YEAST	19 kDa		8
332			Prohibitin-1 OS=Saccharomyces cerevisiae (strain ATCC 204508 / S288c) OX=559292 GN=PHB1 PE=1 SV=2	P40961|PHB1_YEAST	31 kDa		9
333			tRNA-aminoacylation cofactor ARC1 OS=Saccharomyces cerevisiae (strain ATCC 204508 / S288c) OX=559...	P46672|ARC1_YEAST	42 kDa		9

Probability Legend

- over 95%
- 80% to 94%
- 50% to 79%
- 20% to 49%
- 0% to 19%

#	Visible?	Starred?	BioView: 1158 Proteins in 1038 Clusters With 5 Decoys and 22 Hidden	Accession Number	Molecular Weight	Protein Grouping Ambiguity	SrfA_C_CAT
334	☑	☆	Myosin tail region-interacting protein MTI1 OS=Saccharomyces cerevisiae (strain ATCC 204508 / S288c) O...	P47068|BBC1_YEAST	128 kDa		10
335	☑	☆	Uncharacterized protein YGR130C OS=Saccharomyces cerevisiae (strain ATCC 204508 / S288c) OX=55929...	P53278|YG3A_YEAST	93 kDa		8
336	☑	☆	Cluster of ATP-dependent permease PDR12 OS=Saccharomyces cerevisiae (strain ATCC 204508 / S288c) ...	Q02785|PDR12_YEAST [2]	171 kDa	★	10
337	☑	☆	Probable 1,3-beta-glucanosyltransferase GAS3 OS=Saccharomyces cerevisiae (strain ATCC 204508 / S288...	Q03655|GAS3_YEAST	57 kDa		9
338	☑	☆	NADP-dependent alcohol dehydrogenase 6 OS=Saccharomyces cerevisiae (strain ATCC 204508 / S288c) O...	Q04894|ADH6_YEAST	40 kDa		7
339	☑	☆	NADP-dependent 3-hydroxy acid dehydrogenase OS=Saccharomyces cerevisiae (strain ATCC 204508 / S2...	Q05016|YM71_YEAST	29 kDa		9
340	☑	☆	rRNA biogenesis protein RRP5 OS=Saccharomyces cerevisiae (strain ATCC 204508 / S288c) OX=559292 G...	Q05022|RRP5_YEAST	193 kDa		10
341	☑	☆	Protein ZEO1 OS=Saccharomyces cerevisiae (strain ATCC 204508 / S288c) OX=559292 GN=ZEO1 PE=1 SV=3	Q08245|ZEO1_YEAST	13 kDa		7
342	☑	☆	Aspartic proteinase yapsin-3 OS=Saccharomyces cerevisiae (strain ATCC 204508 / S288c) OX=559292 GN...	Q12303|YPS3_YEAST	55 kDa		9
343	☑	☆	Alcohol dehydrogenase 1 OS=Scheffersomyces stipitis (strain ATCC 58785 / CBS 6054 / NBRC 10063 / NRR...	O00097|ADH1_PICST	37 kDa	★	7
344	☑	☆	Eukaryotic translation initiation factor 3 subunit C OS=Saccharomyces cerevisiae (strain YJM789) OX=307...	A6ZN26|EIF3C_YEAS7 (+1)	93 kDa		9
345	☑	☆	Aldehyde dehydrogenase 5, mitochondrial OS=Saccharomyces cerevisiae (strain YJM789) OX=307796 GN...	A6ZR27|ALDH5_YEAS7 (+1)	57 kDa	★	9
346	☑	☆	Eisosome protein 1 OS=Saccharomyces cerevisiae (strain JAY291) OX=574961 GN=EIS1 PE=3 SV=1	C7GL88|EIS1_YEAS2 (+1)	93 kDa		9
347	☑	☆	Cytochrome c isoform 1 OS=Saccharomyces cerevisiae (strain ATCC 204508 / S288c) OX=559292 GN=CYC...	P00044|CYC1_YEAST	12 kDa		5
348	☑	☆	Cytochrome c peroxidase, mitochondrial OS=Saccharomyces cerevisiae (strain ATCC 204508 / S288c) OX...	P00431|CCPR_YEAST	40 kDa		8
349	☑	☆	Threonine dehydratase, mitochondrial OS=Saccharomyces cerevisiae (strain ATCC 204508 / S288c) OX=5...	P00927|THDH_YEAST	64 kDa		10
350	☑	☆	40S ribosomal protein S17-A OS=Saccharomyces cerevisiae (strain ATCC 204508 / S288c) OX=559292 GN...	P02407|RS17A_YEAST (+1)	16 kDa		5
351	☑	☆	40S ribosomal protein S13 OS=Saccharomyces cerevisiae (strain ATCC 204508 / S288c) OX=559292 GN=R...	P05756|RS13_YEAST	17 kDa		5
352	☑	☆	Homocysteine/cysteine synthase OS=Saccharomyces cerevisiae (strain ATCC 204508 / S288c) OX=55929...	P06106|CYSD_YEAST	49 kDa		10
353	☑	☆	40S ribosomal protein S19-A OS=Saccharomyces cerevisiae (strain ATCC 204508 / S288c) OX=559292 GN...	P07280|RS19A_YEAST (+1)	16 kDa		9
354	☑	☆	Ornithine aminotransferase OS=Saccharomyces cerevisiae (strain ATCC 204508 / S288c) OX=559292 GN=...	P07991|OAT_YEAST	46 kDa		8
355	☑	☆	Cytochrome b-c1 complex subunit Rieske, mitochondrial OS=Saccharomyces cerevisiae (strain ATCC 2045...	P08067|UCRI_YEAST	23 kDa		8
356	☑	☆	40S ribosomal protein S24-A OS=Saccharomyces cerevisiae (strain ATCC 204508 / S288c) OX=559292 GN...	P0CX31|RS24A_YEAST (+1)	15 kDa		7
357	☑	☆	60S ribosomal protein L19-A OS=Saccharomyces cerevisiae (strain ATCC 204508 / S288c) OX=559292 GN...	P0CX82|RL19A_YEAST (+1)	22 kDa		9
358	☑	☆	rRNA 2'-O-methyltransferase fibrillarin OS=Saccharomyces cerevisiae (strain ATCC 204508 / S288c) OX=5...	P15646|FBRL_YEAST	34 kDa		7
359	☑	☆	Alpha-mannosidase OS=Saccharomyces cerevisiae (strain ATCC 204508 / S288c) OX=559292 GN=AMS1 P...	P22855|MAN1_YEAST	125 kDa		9
360	☑	☆	40S ribosomal protein S7-A OS=Saccharomyces cerevisiae (strain ATCC 204508 / S288c) OX=559292 GN=...	P26786|RS7A_YEAST	22 kDa	★	7
361	☑	☆	Nuclear localization sequence-binding protein OS=Saccharomyces cerevisiae (strain ATCC 204508 / S288c...	P27476|NSR1_YEAST	45 kDa		7
362	☑	☆	Squalene synthase OS=Saccharomyces cerevisiae (strain ATCC 204508 / S288c) OX=559292 GN=ERG9 PE...	P29704|FDFT_YEAST	52 kDa		8
363	☑	☆	Zuotin OS=Saccharomyces cerevisiae (strain ATCC 204508 / S288c) OX=559292 GN=ZUO1 PE=1 SV=1	P32527|ZUO1_YEAST	49 kDa		8
364	☑	☆	Probable glycosidase CRH2 OS=Saccharomyces cerevisiae (strain ATCC 204508 / S288c) OX=559292 GN=...	P32623|CRH2_YEAST	50 kDa		6
365	☑	☆	Carboxylic acid transporter protein homolog OS=Saccharomyces cerevisiae (strain ATCC 204508 / S288c) ...	P36035|JEN1_YEAST	69 kDa		6
366	☑	☆	Probable glucose transporter HXT5 OS=Saccharomyces cerevisiae (strain ATCC 204508 / S288c) OX=5592...	P38695|HXT5_YEAST	66 kDa	★	7
367	☑	☆	Putative metallocarboxypeptidase ECM14 OS=Saccharomyces cerevisiae (strain ATCC 204508 / S288c) OX...	P38836|ECM14_YEAST	50 kDa		6
368	☑	☆	Branched-chain-amino-acid aminotransferase, mitochondrial OS=Saccharomyces cerevisiae (strain ATCC ...	P38891|BCA1_YEAST	44 kDa	★	8
369	☑	☆	FK506-binding nuclear protein OS=Saccharomyces cerevisiae (strain ATCC 204508 / S288c) OX=559292 G...	P38911|FKBP3_YEAST	47 kDa	★	8
370	☑	☆	26S proteasome regulatory subunit 4 homolog OS=Saccharomyces cerevisiae (strain ATCC 204508 / S288...	P40327|PRS4_YEAST	49 kDa		10

Probability Legend

- over 95%
- 80% to 94%
- 50% to 79%
- 20% to 49%
- 0% to 19%

#	Visible?	Starred?	BioView: 1158 Proteins in 1038 Clusters With 5 Decoys and 22 Hidden	Accession Number	Molecular Weight	Protein Grouping Ambiguity	SrfA_C_CAT
371	☑	☆	Nicotinamidase OS=Saccharomyces cerevisiae (strain ATCC 204508 / S288c) OX=559292 GN=PNC1 PE=1 S...	P53184|PNC1_YEAST	25 kDa		8
372	☑	☆	Probable glycosidase CRH1 OS=Saccharomyces cerevisiae (strain ATCC 204508 / S288c) OX=559292 GN=...	P53301|CRH1_YEAST	53 kDa		7
+ 373	☑	☆	Cluster of Glycerol-3-phosphate dehydrogenase [NAD(+)] 1 OS=Saccharomyces cerevisiae (strain ATCC 2...	Q00055|GPD1_YEAST [3]	43 kDa	★	8
374	☑	☆	40S ribosomal protein S15 OS=Saccharomyces cerevisiae (strain ATCC 204508 / S288c) OX=559292 GN=R...	Q01855|RS15_YEAST	16 kDa		6
375	☑	☆	Cofilin OS=Saccharomyces cerevisiae (strain ATCC 204508 / S288c) OX=559292 GN=COF1 PE=1 SV=1	Q03048|COFI_YEAST	16 kDa		8
376	☑	☆	Meiotic sister chromatid recombination protein 1 OS=Saccharomyces cerevisiae (strain ATCC 204508 / S2...	Q03104|MSC1_YEAST	60 kDa		8
377	☑	☆	Tricalbin-3 OS=Saccharomyces cerevisiae (strain ATCC 204508 / S288c) OX=559292 GN=TCB3 PE=1 SV=1	Q03640|TCB3_YEAST	171 kDa		9
378	☑	☆	1,3-beta-glucanosyltransferase GAS5 OS=Saccharomyces cerevisiae (strain ATCC 204508 / S288c) OX=55...	Q08193|GAS5_YEAST	52 kDa		8
379	☑	☆	Small glutamine-rich tetratricopeptide repeat-containing protein 2 OS=Saccharomyces cerevisiae (strain ...	Q12118|SGT2_YEAST	37 kDa		9
380	☑	☆	26S proteasome regulatory subunit RPN5 OS=Saccharomyces cerevisiae (strain ATCC 204508 / S288c) OX...	Q12250|RPN5_YEAST	52 kDa		9
381	☑	☆	Heat shock protein 42 OS=Saccharomyces cerevisiae (strain ATCC 204508 / S288c) OX=559292 GN=HSP4...	Q12329|HSP42_YEAST	43 kDa		8
382	☑	☆	Glutamate synthase [NADH] OS=Saccharomyces cerevisiae (strain ATCC 204508 / S288c) OX=559292 GN...	Q12680|GLT1_YEAST	238 kDa		9
383	☑	☆	Alcohol dehydrogenase 5 OS=Saccharomyces cerevisiae (strain ATCC 204508 / S288c) OX=559292 GN=AD...	P38113|ADH5_YEAST (+1)	38 kDa	★	7
384	☑	☆	Probable secreted beta-glucosidase UTH1 OS=Saccharomyces cerevisiae (strain YJM789) OX=307796 GN...	A7A003|UTH1_YEAS7 (+3)	37 kDa	★	8
+ 385	☑	☆	Cluster of Anionic trypsin-1 OS=Rattus norvegicus OX=10116 GN=Prss1 PE=1 SV=1 (P00762|TRY1_RAT)	P00762|TRY1_RAT [3]	26 kDa	★	5
386	☑	☆	Anthranilate synthase component 1 OS=Saccharomyces cerevisiae (strain ATCC 204508 / S288c) OX=559...	P00899|TRPE_YEAST	57 kDa		8
387	☑	☆	60S ribosomal protein L28 OS=Saccharomyces cerevisiae (strain ATCC 204508 / S288c) OX=559292 GN=R...	P02406|RL28_YEAST	17 kDa		6
+ 388	☑	☆	Cluster of Albumin OS=Bos taurus OX=9913 GN=ALB PE=1 SV=4 (P02769|ALBU_BOVIN)	P02769|ALBU_BOVIN [3]	69 kDa	★	6
389	☑	☆	60S ribosomal protein L26-A OS=Saccharomyces cerevisiae (strain ATCC 204508 / S288c) OX=559292 GN...	P05743|RL26A_YEAST (+1)	14 kDa		5
390	☑	☆	Ubiquitin-40S ribosomal protein S31 OS=Saccharomyces cerevisiae (strain ATCC 204508 / S288c) OX=559...	P05759|RS31_YEAST (+8)	17 kDa		5
391	☑	☆	Phosphomannomutase OS=Saccharomyces cerevisiae (strain ATCC 204508 / S288c) OX=559292 GN=SEC5...	P07283|PMM_YEAST	29 kDa		7
+ 392	☑	☆	Cluster of Ras-related protein SEC4 OS=Saccharomyces cerevisiae (strain ATCC 204508 / S288c) OX=5592...	P07560|SEC4_YEAST [19]	24 kDa	★	8
393	☑	☆	CDP-diacylglycerol--serine O-phosphatidyltransferase OS=Saccharomyces cerevisiae (strain ATCC 204508...	P08456|PSS_YEAST	31 kDa		6
394	☑	☆	Fructose-1,6-bisphosphatase OS=Saccharomyces cerevisiae (strain ATCC 204508 / S288c) OX=559292 GN...	P09201|F16P_YEAST	38 kDa		7
395	☑	☆	Gramicidin S synthase 2 OS=Aneurinibacillus migulanus OX=47500 GN=grsB PE=3 SV=2	P0C063|GRSB_ANEMI (+1)	509 kDa	★	7
396	☑	☆	40S ribosomal protein S11-A OS=Saccharomyces cerevisiae (strain ATCC 204508 / S288c) OX=559292 GN...	P0CX47|RS11A_YEAST (+1)	18 kDa		6
+ 397	☑	☆	Cluster of 60S ribosomal protein L12-A OS=Saccharomyces cerevisiae (strain ATCC 204508 / S288c) OX=5...	P0CX53|RL12A_YEAST [8]	18 kDa	★	7
398	☑	☆	Single-stranded nucleic acid-binding protein OS=Saccharomyces cerevisiae (strain ATCC 204508 / S288c) ...	P10080|SSBP1_YEAST	33 kDa		7
399	☑	☆	Galactose transporter OS=Saccharomyces cerevisiae (strain ATCC 204508 / S288c) OX=559292 GN=GAL2 ...	P13181|GAL2_YEAST	64 kDa	★	7
400	☑	☆	Adenylyl cyclase-associated protein OS=Saccharomyces cerevisiae (strain ATCC 204508 / S288c) OX=559...	P17555|CAP_YEAST	58 kDa		8
401	☑	☆	Small COPII coat GTPase SAR1 OS=Saccharomyces cerevisiae (strain ATCC 204508 / S288c) OX=559292 G...	P20606|SAR1_YEAST	21 kDa		8
402	☑	☆	12 kDa heat shock protein OS=Saccharomyces cerevisiae (strain ATCC 204508 / S288c) OX=559292 GN=H...	P22943|HSP12_YEAST	12 kDa		4
403	☑	☆	Accumulation of dyads protein 2 OS=Saccharomyces cerevisiae (strain ATCC 204508 / S288c) OX=559292 ...	P25613|ADY2_YEAST	31 kDa	★	8
404	☑	☆	3-ketoacyl-CoA thiolase, peroxisomal OS=Saccharomyces cerevisiae (strain ATCC 204508 / S288c) OX=55...	P27796|THIK_YEAST	45 kDa		7
405	☑	☆	Glycolipid 2-alpha-mannosyltransferase OS=Saccharomyces cerevisiae (strain ATCC 204508 / S288c) OX=...	P27809|KRE2_YEAST	51 kDa		7
406	☑	☆	ATP synthase subunit d, mitochondrial OS=Saccharomyces cerevisiae (strain ATCC 204508 / S288c) OX=5...	P30902|ATP7_YEAST	20 kDa		8
407	☑	☆	Cystathionine gamma-lyase OS=Saccharomyces cerevisiae (strain ATCC 204508 / S288c) OX=559292 GN...	P31373|CYS3_YEAST	43 kDa		8

Probability Legend

- over 95%
- 80% to 94%
- 50% to 79%
- 20% to 49%
- 0% to 19%

#	Visible?	Starred?	BioView: 1158 Proteins in 1038 Clusters With 5 Decoys and 22 Hidden	Accession Number	Molecular Weight	Protein Grouping Ambiguity	SrfA_C_CAT
408	☑	☆	Low-affinity glucose transporter HXT3 OS=Saccharomyces cerevisiae (strain ATCC 204508 / S288c) OX=5...	P32466|HXT3_YEAST	63 kDa	★	8
409	☑	☆	Eukaryotic translation initiation factor 2 subunit gamma OS=Saccharomyces cerevisiae (strain ATCC 2045...	P32481|IF2G_YEAST	58 kDa		9
410	☑	☆	Ammonia transport outward protein 2 OS=Saccharomyces cerevisiae (strain ATCC 204508 / S288c) OX=5...	P32907|ATO2_YEAST	31 kDa	★	8
411	☑	☆	26S proteasome regulatory subunit 6A OS=Saccharomyces cerevisiae (strain ATCC 204508 / S288c) OX=5...	P33297|PRS6A_YEAST	48 kDa		9
412	☑	☆	Cluster of 26S proteasome regulatory subunit 6B homolog OS=Saccharomyces cerevisiae (strain ATCC 20...	P33298|PRS6B_YEAST [2]	48 kDa	★	8
413	☑	☆	H/ACA ribonucleoprotein complex subunit CBF5 OS=Saccharomyces cerevisiae (strain ATCC 204508 / S28...	P33322|CBF5_YEAST	55 kDa		8
414	☑	☆	60S ribosomal protein L14-A OS=Saccharomyces cerevisiae (strain ATCC 204508 / S288c) OX=559292 GN...	P36105|RL14A_YEAST (+1)	15 kDa		8
415	☑	☆	Trehalose synthase complex regulatory subunit TSL1 OS=Saccharomyces cerevisiae (strain ATCC 204508 ...	P38427|TSL1_YEAST	123 kDa		8
416	☑	☆	Cluster of GTP-binding protein YPT31/YPT8 OS=Saccharomyces cerevisiae (strain ATCC 204508 / S288c) O...	P38555|YPT31_YEAST [2]	24 kDa	★	8
417	☑	☆	Proteasome subunit alpha type-6 OS=Saccharomyces cerevisiae (strain ATCC 204508 / S288c) OX=55929...	P40302|PSA6_YEAST	26 kDa		7
418	☑	☆	Proteasome subunit alpha type-4 OS=Saccharomyces cerevisiae (strain ATCC 204508 / S288c) OX=55929...	P40303|PSA4_YEAST	28 kDa		8
419	☑	☆	Cluster of Glycerol-1-phosphate phosphohydrolase 1 OS=Saccharomyces cerevisiae (strain ATCC 204508 ...	P41277|GPP1_YEAST [2]	28 kDa	★	8
420	☑	☆	Probable family 17 glucosidase SCW11 OS=Saccharomyces cerevisiae (strain ATCC 204508 / S288c) OX=5...	P53189|SCW11_YEAST	56 kDa		8
421	☑	☆	Mannosyltransferase KTR6 OS=Saccharomyces cerevisiae (strain ATCC 204508 / S288c) OX=559292 GN=...	P54070|KTR6_YEAST	52 kDa		9
422	☑	☆	Importin subunit alpha OS=Saccharomyces cerevisiae (strain ATCC 204508 / S288c) OX=559292 GN=SRP1...	Q02821|IMA1_YEAST	60 kDa		8
423	☑	☆	Citrate/oxoglutarate carrier protein OS=Saccharomyces cerevisiae (strain ATCC 204508 / S288c) OX=559...	Q04013|YHM2_YEAST	34 kDa		6
424	☑	☆	ATP-dependent RNA helicase SUB2 OS=Saccharomyces cerevisiae (strain ATCC 204508 / S288c) OX=5592...	Q07478|SUB2_YEAST	50 kDa		8
425	☑	☆	Thiosulfate:glutathione sulfurtransferase OS=Saccharomyces cerevisiae (strain ATCC 204508 / S288c) OX...	Q12305|RDL1_YEAST	15 kDa		6
426	☑	☆	Reticulon-like protein 2 OS=Saccharomyces cerevisiae (strain ATCC 204508 / S288c) OX=559292 GN=RTN...	Q12443|RTN2_YEAST	44 kDa		8
427	☑	☆	Putative reductase 1 OS=Saccharomyces cerevisiae (strain ATCC 204508 / S288c) OX=559292 GN=YPR1 P...	Q12458|YPR1_YEAST	35 kDa	★	7
428	☑	☆	GTP-binding protein YPT1 OS=Saccharomyces cerevisiae (strain ATCC 204508 / S288c) OX=559292 GN=YP...	P01123|YPT1_YEAST	23 kDa		6
429	☑	☆	Argininosuccinate lyase OS=Saccharomyces cerevisiae (strain ATCC 204508 / S288c) OX=559292 GN=ARG...	P04076|ARLY_YEAST	52 kDa		7
430	☑	☆	Ornithine carbamoyltransferase OS=Saccharomyces cerevisiae (strain ATCC 204508 / S288c) OX=559292 ...	P05150|OTC_YEAST	38 kDa		6
431	☑	☆	60S ribosomal protein L22-A OS=Saccharomyces cerevisiae (strain ATCC 204508 / S288c) OX=559292 GN...	P05749|RL22A_YEAST	14 kDa		5
432	☑	☆	Histidine--tRNA ligase, mitochondrial OS=Saccharomyces cerevisiae (strain ATCC 204508 / S288c) OX=55...	P07263|SYH_YEAST	60 kDa		6
433	☑	☆	Delta-1-pyrroline-5-carboxylate dehydrogenase, mitochondrial OS=Saccharomyces cerevisiae (strain AT...	P07275|PUT2_YEAST	64 kDa		7
434	☑	☆	C-1-tetrahydrofolate synthase, mitochondrial OS=Saccharomyces cerevisiae (strain ATCC 204508 / S288c...	P09440|C1TM_YEAST	106 kDa	★	6
435	☑	☆	60S ribosomal protein L11-A OS=Saccharomyces cerevisiae (strain ATCC 204508 / S288c) OX=559292 GN...	P0C0W9|RL11A_YEAST (+1)	20 kDa		7
436	☑	☆	60S ribosomal protein L31-B OS=Saccharomyces cerevisiae (strain ATCC 204508 / S288c) OX=559292 GN...	P0C2H9|RL31B_YEAST	13 kDa		7
437	☑	☆	40S ribosomal protein S8-A OS=Saccharomyces cerevisiae (strain ATCC 204508 / S288c) OX=559292 GN=...	P0CX39|RS8A_YEAST (+1)	22 kDa		4
438	☑	☆	60S ribosomal protein L1-A OS=Saccharomyces cerevisiae (strain ATCC 204508 / S288c) OX=559292 GN=...	P0CX43|RL1A_YEAST (+1)	24 kDa		6
439	☑	☆	60S ribosomal protein L18-A OS=Saccharomyces cerevisiae (strain ATCC 204508 / S288c) OX=559292 GN...	P0CX49|RL18A_YEAST (+1)	21 kDa		5
440	☑	☆	ADP-ribosylation factor 1 OS=Saccharomyces cerevisiae (strain ATCC 204508 / S288c) OX=559292 GN=AR...	P11076|ARF1_YEAST (+1)	21 kDa		7
441	☑	☆	Cluster of T-complex protein 1 subunit alpha OS=Saccharomyces cerevisiae (strain ATCC 204508 / S288c) ...	P12612|TCPA_YEAST [2]	60 kDa	★	7
442	☑	☆	60S ribosomal protein L30 OS=Saccharomyces cerevisiae (strain ATCC 204508 / S288c) OX=559292 GN=R...	P14120|RL30_YEAST	11 kDa		6
443	☑	☆	Protein transport protein SEC23 OS=Saccharomyces cerevisiae (strain ATCC 204508 / S288c) OX=559292 ...	P15303|SEC23_YEAST	85 kDa		7
444	☑	☆	Protein APA1 OS=Saccharomyces cerevisiae (strain ATCC 204508 / S288c) OX=559292 GN=APA1 PE=1 SV...	P16550|APA1_YEAST	36 kDa		7

Probability Legend

- over 95%
- 80% to 94%
- 50% to 79%
- 20% to 49%
- 0% to 19%

#	Visible?	Starred?	BioView: 1158 Proteins in 1038 Clusters With 5 Decoys and 22 Hidden	Accession Number	Molecular Weight	Protein Grouping Ambiguity	SrfA_C_CAT
445	☑	☆	Proteasome subunit alpha type-1 OS=Saccharomyces cerevisiae (strain ATCC 204508 / S288c) OX=55929...	P21243|PSA1_YEAST	28 kDa		6
446	☑	☆	Proteasome subunit alpha type-2 OS=Saccharomyces cerevisiae (strain ATCC 204508 / S288c) OX=55929...	P23639|PSA2_YEAST	27 kDa		6
+ 447	☑	☆	Cluster of 60S ribosomal protein L24-B OS=Saccharomyces cerevisiae (strain ATCC 204508 / S288c) OX=5...	P24000|RL24B_YEAST [2]	18 kDa	★	6
448	☑	☆	Protein SIS1 OS=Saccharomyces cerevisiae (strain ATCC 204508 / S288c) OX=559292 GN=SIS1 PE=1 SV=1	P25294|SIS1_YEAST	38 kDa		7
449	☑	☆	Acetolactate synthase small subunit, mitochondrial OS=Saccharomyces cerevisiae (strain ATCC 204508 / ...	P25605|ILV6_YEAST	34 kDa		6
450	☑	☆	60S ribosomal protein L16-B OS=Saccharomyces cerevisiae (strain ATCC 204508 / S288c) OX=559292 GN...	P26785|RL16B_YEAST	22 kDa	★	6
451	☑	☆	Alpha-1,2 mannosyltransferase KTR1 OS=Saccharomyces cerevisiae (strain ATCC 204508 / S288c) OX=55...	P27810|KTR1_YEAST	46 kDa		7
452	☑	☆	Thioredoxin reductase 1 OS=Saccharomyces cerevisiae (strain ATCC 204508 / S288c) OX=559292 GN=TR...	P29509|TRXB1_YEAST	34 kDa		6
453	☑	☆	Mannose-6-phosphate isomerase OS=Saccharomyces cerevisiae (strain ATCC 204508 / S288c) OX=55929...	P29952|MPI_YEAST	48 kDa		7
454	☑	☆	Fumarate reductase 1 OS=Saccharomyces cerevisiae (strain ATCC 204508 / S288c) OX=559292 GN=FRD1 ...	P32614|FRDS_YEAST	51 kDa		7
455	☑	☆	Phosphoglucomutase 1 OS=Saccharomyces cerevisiae (strain ATCC 204508 / S288c) OX=559292 GN=PGM...	P33401|PGM1_YEAST	63 kDa	★	7
456	☑	☆	Imidazole glycerol phosphate synthase hisHF OS=Saccharomyces cerevisiae (strain ATCC 204508 / S288c...	P33734|HIS5_YEAST	61 kDa		7
457	☑	☆	Peptidyl-prolyl cis-trans isomerase D OS=Saccharomyces cerevisiae (strain ATCC 204508 / S288c) OX=55...	P35176|CYPD_YEAST	25 kDa	★	7
458	☑	☆	GTP-binding protein YPT52 OS=Saccharomyces cerevisiae (strain ATCC 204508 / S288c) OX=559292 GN=Y...	P36018|YPT52_YEAST	26 kDa	★	6
459	☑	☆	Tyrosine--tRNA ligase, cytoplasmic OS=Saccharomyces cerevisiae (strain ATCC 204508 / S288c) OX=5592...	P36421|SYYC_YEAST	44 kDa		7
460	☑	☆	60S ribosomal protein L32 OS=Saccharomyces cerevisiae (strain ATCC 204508 / S288c) OX=559292 GN=R...	P38061|RL32_YEAST	15 kDa		5
461	☑	☆	Enoyl-[acyl-carrier-protein] reductase, mitochondrial OS=Saccharomyces cerevisiae (strain ATCC 204508 ...	P38071|ETR1_YEAST	42 kDa		7
462	☑	☆	40S ribosomal protein S20 OS=Saccharomyces cerevisiae (strain ATCC 204508 / S288c) OX=559292 GN=R...	P38701|RS20_YEAST	14 kDa		5
463	☑	☆	T-complex protein 1 subunit beta OS=Saccharomyces cerevisiae (strain ATCC 204508 / S288c) OX=55929...	P39076|TCPB_YEAST	57 kDa		7
464	☑	☆	(R,R)-butanediol dehydrogenase OS=Saccharomyces cerevisiae (strain ATCC 204508 / S288c) OX=559292...	P39714|BDH1_YEAST	42 kDa		7
465	☑	☆	Protein SSO2 OS=Saccharomyces cerevisiae (strain ATCC 204508 / S288c) OX=559292 GN=SSO2 PE=1 SV=2	P39926|SSO2_YEAST	34 kDa	★	6
466	☑	☆	E3 ubiquitin-protein ligase RSP5 OS=Saccharomyces cerevisiae (strain ATCC 204508 / S288c) OX=559292 ...	P39940|RSP5_YEAST	92 kDa		7
467	☑	☆	Rab GDP-dissociation inhibitor OS=Saccharomyces cerevisiae (strain ATCC 204508 / S288c) OX=559292 G...	P39958|GDI1_YEAST	51 kDa		6
468	☑	☆	Vacuolar protein 8 OS=Saccharomyces cerevisiae (strain ATCC 204508 / S288c) OX=559292 GN=VAC8 PE=...	P39968|VAC8_YEAST	63 kDa		7
469	☑	☆	Protein FMP52, mitochondrial OS=Saccharomyces cerevisiae (strain ATCC 204508 / S288c) OX=559292 GN...	P40008|FMP52_YEAST	25 kDa		6
470	☑	☆	Probable secreted beta-glucosidase SIM1 OS=Saccharomyces cerevisiae (strain ATCC 204508 / S288c) OX...	P40472|SIM1_YEAST	48 kDa	★	5
471	☑	☆	Protein transport protein SEC24 OS=Saccharomyces cerevisiae (strain ATCC 204508 / S288c) OX=559292 ...	P40482|SEC24_YEAST	104 kDa		9
472	☑	☆	Actin-related protein 3 OS=Saccharomyces cerevisiae (strain ATCC 204508 / S288c) OX=559292 GN=ARP...	P47117|ARP3_YEAST	50 kDa		9
473	☑	☆	40S ribosomal protein S7-B OS=Saccharomyces cerevisiae (strain ATCC 204508 / S288c) OX=559292 GN=...	P48164|RS7B_YEAST	22 kDa	★	7
474	☑	☆	High-affinity glutamine permease OS=Saccharomyces cerevisiae (strain ATCC 204508 / S288c) OX=55929...	P48813|GNP1_YEAST	74 kDa		5
475	☑	☆	Dynamin-related protein DNM1 OS=Saccharomyces cerevisiae (strain ATCC 204508 / S288c) OX=559292 ...	P54861|DNM1_YEAST	85 kDa		8
476	☑	☆	26S proteasome regulatory subunit 8 homolog OS=Saccharomyces cerevisiae (strain ATCC 204508 / S288...	Q01939|PRS8_YEAST	45 kDa		7
477	☑	☆	Mitochondrial 2-oxodicarboxylate carrier 1 OS=Saccharomyces cerevisiae (strain ATCC 204508 / S288c) O...	Q03028|ODC1_YEAST	34 kDa		7
478	☑	☆	Glucose-6-phosphate 1-epimerase OS=Saccharomyces cerevisiae (strain ATCC 204508 / S288c) OX=5592...	Q03161|YMY9_YEAST	34 kDa		7
479	☑	☆	Translation initiation factor RLI1 OS=Saccharomyces cerevisiae (strain ATCC 204508 / S288c) OX=559292...	Q03195|RLI1_YEAST	68 kDa		7
480	☑	☆	26S proteasome regulatory subunit RPN9 OS=Saccharomyces cerevisiae (strain ATCC 204508 / S288c) OX...	Q04062|RPN9_YEAST	46 kDa		7
481	☑	☆	Reticulon-like protein 1 OS=Saccharomyces cerevisiae (strain ATCC 204508 / S288c) OX=559292 GN=RTN...	Q04947|RTN1_YEAST	33 kDa		6

Probability Legend

- over 95%
- 80% to 94%
- 50% to 79%
- 20% to 49%
- 0% to 19%

#	Visible?	Starred?	BioView: 1158 Proteins in 1038 Clusters With 5 Decoys and 22 Hidden	Accession Number	Molecular Weight	Protein Grouping Ambiguity	SrfA_C_CAT
482	☑	☆	Coronin-like protein OS=Saccharomyces cerevisiae (strain ATCC 204508 / S288c) OX=559292 GN=CRN1 PE...	Q06440|CORO_YEAST	73 kDa		7
483	☑	☆	Putative carboxymethylenebutenolidase OS=Saccharomyces cerevisiae (strain ATCC 204508 / S288c) OX...	Q07505|DLHH_YEAST	31 kDa		6
484	☑	☆	Lysophospholipase 3 OS=Saccharomyces cerevisiae (strain ATCC 204508 / S288c) OX=559292 GN=PLB3 P...	Q08108|PLB3_YEAST	75 kDa		8
485	☑	☆	Non-classical export protein 2 OS=Saccharomyces cerevisiae (strain ATCC 204508 / S288c) OX=559292 G...	Q12207|NCE2_YEAST	19 kDa		5
486	☑	☆	Tricalbin-1 OS=Saccharomyces cerevisiae (strain ATCC 204508 / S288c) OX=559292 GN=TCB1 PE=1 SV=1	Q12466|TCB1_YEAST	134 kDa		8
487	☑	☆	Suprabasin OS=Homo sapiens OX=9606 GN=SBSN PE=1 SV=2	Q6UWP8|SBSN_HUMAN	61 kDa		5
488	☑	☆	Probable secreted beta-glucosidase SUN4 OS=Saccharomyces cerevisiae (strain ATCC 204508 / S288c) OX...	P53616|SUN4_YEAST	43 kDa	★	4
489	☑	☆	Nascent polypeptide-associated complex subunit alpha OS=Saccharomyces cerevisiae (strain YJM789) OX...	A6ZT99|NACA_YEAS7 (+1)	19 kDa		4
490	☑	☆	Nascent polypeptide-associated complex subunit beta-1 OS=Saccharomyces cerevisiae (strain YJM789) O...	A6ZWL1|NACB1_YEAS7 (+1)	17 kDa		4
491	☑	☆	Multiprotein-bridging factor 1 OS=Saccharomyces cerevisiae (strain ATCC 204508 / S288c) OX=559292 GN...	O14467|MBF1_YEAST	16 kDa		6
492	☑	☆	Cytochrome c oxidase subunit 6, mitochondrial OS=Saccharomyces cerevisiae (strain ATCC 204508 / S288...	P00427|COX6_YEAST	17 kDa		5
493	☑	☆	Superoxide dismutase [Mn], mitochondrial OS=Saccharomyces cerevisiae (strain ATCC 204508 / S288c) O...	P00447|SODM_YEAST	26 kDa		5
494	☑	☆	Methionine--tRNA ligase, cytoplasmic OS=Saccharomyces cerevisiae (strain ATCC 204508 / S288c) OX=55...	P00958|SYMC_YEAST	86 kDa		6
495	☑	☆	Histone H4 OS=Saccharomyces cerevisiae (strain ATCC 204508 / S288c) OX=559292 GN=HHF1 PE=1 SV=2	P02309|H4_YEAST (+1)	11 kDa		6
496	☑	☆	Cytochrome c oxidase subunit 4, mitochondrial OS=Saccharomyces cerevisiae (strain ATCC 204508 / S288...	P04037|COX4_YEAST	17 kDa		5
⊞ 497	☑	☆	Cluster of 60S ribosomal protein L33-A OS=Saccharomyces cerevisiae (strain ATCC 204508 / S288c) OX=5...	P05744|RL33A_YEAST [2]	12 kDa	★	6
498	☑	☆	cAMP-dependent protein kinase regulatory subunit OS=Saccharomyces cerevisiae (strain ATCC 204508 / ...	P07278|KAPR_YEAST	47 kDa		6
499	☑	☆	Protease B inhibitor 2 OS=Saccharomyces cerevisiae (strain ATCC 204508 / S288c) OX=559292 GN=PBI2 P...	P0CT04|IPB2_YEAST	9 kDa		5
500	☑	☆	DNA-directed RNA polymerase I subunit RPA190 OS=Saccharomyces cerevisiae (strain ATCC 204508 / S28...	P10964|RPA1_YEAST	186 kDa		6
501	☑	☆	Glucose-6-phosphate 1-dehydrogenase OS=Saccharomyces cerevisiae (strain ATCC 204508 / S288c) OX=...	P11412|G6PD_YEAST	58 kDa		6
502	☑	☆	Eukaryotic peptide chain release factor subunit 1 OS=Saccharomyces cerevisiae (strain ATCC 204508 / S2...	P12385|ERF1_YEAST	49 kDa		6
503	☑	☆	Glutamine--fructose-6-phosphate aminotransferase [isomerizing] OS=Saccharomyces cerevisiae (strain ...	P14742|GFA1_YEAST	80 kDa		6
504	☑	☆	Protein translocation protein SEC63 OS=Saccharomyces cerevisiae (strain ATCC 204508 / S288c) OX=559...	P14906|SEC63_YEAST	75 kDa		6
505	☑	☆	Proliferating cell nuclear antigen OS=Saccharomyces cerevisiae (strain ATCC 204508 / S288c) OX=559292...	P15873|PCNA_YEAST	29 kDa		6
506	☑	☆	Pyruvate dehydrogenase complex protein X component, mitochondrial OS=Saccharomyces cerevisiae (str...	P16451|ODPX_YEAST	45 kDa		6
507	☑	☆	Homoserine kinase OS=Saccharomyces cerevisiae (strain ATCC 204508 / S288c) OX=559292 GN=THR1 PE...	P17423|KHSE_YEAST	39 kDa		5
508	☑	☆	Thioredoxin-2 OS=Saccharomyces cerevisiae (strain ATCC 204508 / S288c) OX=559292 GN=TRX2 PE=1 SV...	P22803|TRX2_YEAST	11 kDa	★	6
509	☑	☆	Proteasome subunit alpha type-3 OS=Saccharomyces cerevisiae (strain ATCC 204508 / S288c) OX=55929...	P23638|PSA3_YEAST	29 kDa		6
510	☑	☆	SEC14 cytosolic factor OS=Saccharomyces cerevisiae (strain ATCC 204508 / S288c) OX=559292 GN=SEC14...	P24280|SEC14_YEAST	35 kDa	★	6
511	☑	☆	Peptidyl-prolyl cis-trans isomerase C, mitochondrial OS=Saccharomyces cerevisiae (strain ATCC 204508 / ...	P25719|CYPC_YEAST	20 kDa	★	6
512	☑	☆	60S ribosomal protein L16-A OS=Saccharomyces cerevisiae (strain ATCC 204508 / S288c) OX=559292 GN...	P26784|RL16A_YEAST	22 kDa	★	5
⊞ 513	☑	☆	Cluster of CTP synthase 1 OS=Saccharomyces cerevisiae (strain ATCC 204508 / S288c) OX=559292 GN=U...	P28274|URA7_YEAST [3]	65 kDa	★	5
514	☑	☆	V-type proton ATPase subunit C OS=Saccharomyces cerevisiae (strain ATCC 204508 / S288c) OX=559292 ...	P31412|VATC_YEAST	44 kDa		6
515	☑	☆	Heme-binding protein HMX1 OS=Saccharomyces cerevisiae (strain ATCC 204508 / S288c) OX=559292 GN=...	P32339|HMX1_YEAST	37 kDa		6
516	☑	☆	V-type proton ATPase subunit d OS=Saccharomyces cerevisiae (strain ATCC 204508 / S288c) OX=559292 ...	P32366|VA0D_YEAST	40 kDa		6
517	☑	☆	Diphosphomevalonate decarboxylase OS=Saccharomyces cerevisiae (strain ATCC 204508 / S288c) OX=55...	P32377|MVD1_YEAST	44 kDa		6
518	☑	☆	Actin-related protein 2 OS=Saccharomyces cerevisiae (strain ATCC 204508 / S288c) OX=559292 GN=ARP...	P32381|ARP2_YEAST	44 kDa		6

Probability Legend

over 95%

80% to 94%

50% to 79%

20% to 49%

0% to 19%

#	Visible?	Starred?	BioView: 1158 Proteins in 1038 Clusters With 5 Decoys and 22 Hidden	Accession Number	Molecular Weight	Protein Grouping Ambiguity	SrfA_C_CAT
519	☑	☆	Cell division control protein 12 OS=Saccharomyces cerevisiae (strain ATCC 204508 / S288c) OX=559292 G...	P32468|CDC12_YEAST	47 kDa		5
520	☑	☆	Cluster of Serine/threonine-protein phosphatase PP1-2 OS=Saccharomyces cerevisiae (strain ATCC 20450...	P32598|PP12_YEAST [4]	36 kDa	★	6
521	☑	☆	1,4-alpha-glucan-branching enzyme OS=Saccharomyces cerevisiae (strain ATCC 204508 / S288c) OX=559...	P32775|GLGB_YEAST	81 kDa		6
522	☑	☆	Protein SSO1 OS=Saccharomyces cerevisiae (strain ATCC 204508 / S288c) OX=559292 GN=SSO1 PE=1 SV=2	P32867|SSO1_YEAST	33 kDa	★	5
523	☑	☆	Protein transport protein SEC61 OS=Saccharomyces cerevisiae (strain ATCC 204508 / S288c) OX=559292 ...	P32915|SC61A_YEAST	53 kDa		6
524	☑	☆	Nuclear transport factor 2 OS=Saccharomyces cerevisiae (strain ATCC 204508 / S288c) OX=559292 GN=N...	P33331|NTF2_YEAST	14 kDa		6
525	☑	☆	Heat shock protein 78, mitochondrial OS=Saccharomyces cerevisiae (strain ATCC 204508 / S288c) OX=55...	P33416|HSP78_YEAST	91 kDa		6
526	☑	☆	Eukaryotic translation initiation factor 4B OS=Saccharomyces cerevisiae (strain ATCC 204508 / S288c) OX...	P34167|IF4B_YEAST	49 kDa		6
527	☑	☆	Peroxiredoxin PRX1, mitochondrial OS=Saccharomyces cerevisiae (strain ATCC 204508 / S288c) OX=5592...	P34227|PRX1_YEAST	29 kDa		7
528	☑	☆	Uncharacterized protein MRP8 OS=Saccharomyces cerevisiae (strain ATCC 204508 / S288c) OX=559292 G...	P35719|MRP8_YEAST	25 kDa		5
529	☑	☆	NAD-dependent malic enzyme, mitochondrial OS=Saccharomyces cerevisiae (strain ATCC 204508 / S288c)...	P36013|MAOM_YEAST	74 kDa		6
530	☑	☆	Alpha-1,2-mannosyltransferase MNN2 OS=Saccharomyces cerevisiae (strain ATCC 204508 / S288c) OX=5...	P38069|MNN2_YEAST	68 kDa		5
531	☑	☆	Multisite-specific tRNA:(cytosine-C(5))-methyltransferase OS=Saccharomyces cerevisiae (strain ATCC 20...	P38205|NCL1_YEAST	78 kDa		6
532	☑	☆	Increased sodium tolerance protein 2 OS=Saccharomyces cerevisiae (strain ATCC 204508 / S288c) OX=55...	P38250|IST2_YEAST	106 kDa		4
533	☑	☆	SRP-independent targeting protein 3 OS=Saccharomyces cerevisiae (strain ATCC 204508 / S288c) OX=55...	P38264|PHO88_YEAST	21 kDa		4
534	☑	☆	NADH-cytochrome b5 reductase 1 OS=Saccharomyces cerevisiae (strain ATCC 204508 / S288c) OX=55929...	P38626|NCB5R_YEAST	31 kDa		5
535	☑	☆	Aromatic amino acid aminotransferase 2 OS=Saccharomyces cerevisiae (strain ATCC 204508 / S288c) OX...	P38840|ARO9_YEAST	59 kDa		6
536	☑	☆	Dolichyl-diphosphooligosaccharide--protein glycosyltransferase subunit STT3 OS=Saccharomyces cerevisi...	P39007|STT3_YEAST	82 kDa		6
537	☑	☆	T-complex protein 1 subunit zeta OS=Saccharomyces cerevisiae (strain ATCC 204508 / S288c) OX=55929...	P39079|TCPZ_YEAST	60 kDa		6
538	☑	☆	ABC transporter ATP-binding protein ARB1 OS=Saccharomyces cerevisiae (strain ATCC 204508 / S288c) O...	P40024|ARB1_YEAST	68 kDa		6
539	☑	☆	Protein MMF1, mitochondrial OS=Saccharomyces cerevisiae (strain ATCC 204508 / S288c) OX=559292 GN...	P40185|MMF1_YEAST	16 kDa		6
540	☑	☆	Cluster of D-3-phosphoglycerate dehydrogenase 2 OS=Saccharomyces cerevisiae (strain ATCC 204508 / S...	P40510|SER33_YEAST [2]	51 kDa	★	6
541	☑	☆	Regulation of enolase protein 1 OS=Saccharomyces cerevisiae (strain ATCC 204508 / S288c) OX=559292 ...	P40893|REE1_YEAST	22 kDa		6
542	☑	☆	Probable electron transfer flavoprotein subunit beta OS=Saccharomyces cerevisiae (strain ATCC 204508 /...	P42940|ETFB_YEAST	29 kDa		6
543	☑	☆	T-complex protein 1 subunit eta OS=Saccharomyces cerevisiae (strain ATCC 204508 / S288c) OX=559292 ...	P42943|TCPH_YEAST	60 kDa	★	6
544	☑	☆	Alpha-1,2-mannosyltransferase MNN5 OS=Saccharomyces cerevisiae (strain ATCC 204508 / S288c) OX=5...	P46982|MNN5_YEAST	67 kDa		5
545	☑	☆	Protein PRY1 OS=Saccharomyces cerevisiae (strain ATCC 204508 / S288c) OX=559292 GN=PRY1 PE=1 SV=1	P47032|PRY1_YEAST	31 kDa	★	6
546	☑	☆	Homoaconitase, mitochondrial OS=Saccharomyces cerevisiae (strain ATCC 204508 / S288c) OX=559292 G...	P49367|LYS4_YEAST	75 kDa		6
547	☑	☆	Dicarboxylic amino acid permease OS=Saccharomyces cerevisiae (strain ATCC 204508 / S288c) OX=5592...	P53388|DIP5_YEAST	68 kDa		4
548	☑	☆	Methylenetetrahydrofolate dehydrogenase [NAD(+)] OS=Saccharomyces cerevisiae (strain ATCC 204508 ...	Q02046|MTD1_YEAST	36 kDa		6
549	☑	☆	ATP-dependent RNA helicase HAS1 OS=Saccharomyces cerevisiae (strain ATCC 204508 / S288c) OX=5592...	Q03532|HAS1_YEAST	57 kDa		6
550	☑	☆	RuvB-like protein 1 OS=Saccharomyces cerevisiae (strain ATCC 204508 / S288c) OX=559292 GN=RVB1 PE...	Q03940|RUVB1_YEAST	50 kDa		5
551	☑	☆	Eukaryotic translation initiation factor 3 subunit G OS=Saccharomyces cerevisiae (strain ATCC 204508 / S...	Q04067|EIF3G_YEAST	31 kDa		6
552	☑	☆	Fatty aldehyde dehydrogenase HFD1 OS=Saccharomyces cerevisiae (strain ATCC 204508 / S288c) OX=55...	Q04458|HFD1_YEAST	60 kDa		6
553	☑	☆	Glucose-signaling factor 2 OS=Saccharomyces cerevisiae (strain ATCC 204508 / S288c) OX=559292 GN=G...	Q04697|GSF2_YEAST	46 kDa		5
554	☑	☆	Endoplasmic reticulum transmembrane protein 3 OS=Saccharomyces cerevisiae (strain ATCC 204508 / S2...	Q07451|YET3_YEAST	23 kDa		5
555	☑	☆	[NU+] prion formation protein 1 OS=Saccharomyces cerevisiae (strain ATCC 204508 / S288c) OX=559292 ...	Q08972|NEW1_YEAST	134 kDa		6

Probability Legend

- over 95%
- 80% to 94%
- 50% to 79%
- 20% to 49%
- 0% to 19%

#	Visible?	Starred?	BioView: 1158 Proteins in 1038 Clusters With 5 Decoys and 22 Hidden	Accession Number	Molecular Weight	Protein Grouping Ambiguity	SrfA_C_CAT
556	☑	☆	Chitin biosynthesis protein CHS5 OS=Saccharomyces cerevisiae (strain ATCC 204508 / S288c) OX=559292 ...	Q12114|CHS5_YEAST	74 kDa		6
557	☑	☆	ATP synthase subunit delta, mitochondrial OS=Saccharomyces cerevisiae (strain ATCC 204508 / S288c) O...	Q12165|ATPD_YEAST	17 kDa	★	5
558	☑	☆	26S proteasome regulatory subunit RPN6 OS=Saccharomyces cerevisiae (strain ATCC 204508 / S288c) OX...	Q12377|RPN6_YEAST	50 kDa		6
559	☑	☆	Probable electron transfer flavoprotein subunit alpha, mitochondrial OS=Saccharomyces cerevisiae (strai...	Q12480|ETFA_YEAST	37 kDa		5
560	☑	☆	40S ribosomal protein S21-B OS=Saccharomyces cerevisiae (strain ATCC 204508 / S288c) OX=559292 GN...	Q3E754|RS21B_YEAST	10 kDa		5
561	☑	☆	Histone H3 OS=Saccharomyces cerevisiae (strain ATCC 204508 / S288c) OX=559292 GN=HHT1 PE=1 SV=2	P61830|H3_YEAST (+4)	15 kDa		4
562	☑	☆	Calmodulin-like protein 5 OS=Homo sapiens OX=9606 GN=CALML5 PE=1 SV=2	Q9NZT1|CALL5_HUMAN	16 kDa		3
⊞ 563	☑	☆	Cluster of Chaperone protein DnaK OS=Solibacter usitatus (strain Ellin6076) OX=234267 GN=dnaK PE=3 SV...	Q01PM8|DNAK_SOLUE [2]	69 kDa	★	4
564	☑	☆	Leucine aminopeptidase 2 OS=Saccharomyces cerevisiae (strain YJM789) OX=307796 GN=SCY_4744 PE=...	A6ZS33|LKHA4_YEAS7 (+1)	77 kDa		5
565	☑	☆	Pescadillo homolog OS=Saccharomyces cerevisiae (strain YJM789) OX=307796 GN=NOP7 PE=3 SV=1	A6ZV85|PESC_YEAS7 (+1)	70 kDa		4
566	☑	☆	Protein HRI1 OS=Saccharomyces cerevisiae (strain YJM789) OX=307796 GN=HRI1 PE=3 SV=1	A7A1I2|HRI1_YEAS7 (+7)	28 kDa		4
567	☑	☆	Altered inheritance of mitochondria protein 41, mitochondrial OS=Saccharomyces cerevisiae (strain RM11...	B3LJN9|AIM41_YEAS1 (+3)	21 kDa		5
568	☑	☆	Multifunctional tryptophan biosynthesis protein OS=Saccharomyces cerevisiae (strain ATCC 204508 / S28...	P00937|TRPG_YEAST	53 kDa		5
569	☑	☆	Cystatin-A OS=Homo sapiens OX=9606 GN=CSTA PE=1 SV=1	P01040|CYTA_HUMAN	11 kDa		5
570	☑	☆	Delta-aminolevulinic acid dehydratase OS=Saccharomyces cerevisiae (strain ATCC 204508 / S288c) OX=5...	P05373|HEM2_YEAST	38 kDa		5
571	☑	☆	60S ribosomal protein L23-A OS=Saccharomyces cerevisiae (strain ATCC 204508 / S288c) OX=559292 GN...	P0CX41|RL23A_YEAST (+1)	14 kDa		4
572	☑	☆	60S ribosomal protein L35-A OS=Saccharomyces cerevisiae (strain ATCC 204508 / S288c) OX=559292 GN...	P0CX84|RL35A_YEAST (+1)	14 kDa		5
573	☑	☆	3-hydroxy-3-methylglutaryl-coenzyme A reductase 1 OS=Saccharomyces cerevisiae (strain ATCC 204508...	P12683|HMDH1_YEAST	116 kDa		5
574	☑	☆	Chitin synthase 2 OS=Saccharomyces cerevisiae (strain ATCC 204508 / S288c) OX=559292 GN=CHS2 PE=1...	P14180|CHS2_YEAST	110 kDa		4
575	☑	☆	Proline-specific permease OS=Saccharomyces cerevisiae (strain ATCC 204508 / S288c) OX=559292 GN=P...	P15380|PUT4_YEAST	69 kDa		4
576	☑	☆	Guanylate kinase OS=Saccharomyces cerevisiae (strain ATCC 204508 / S288c) OX=559292 GN=GUK1 PE=...	P15454|KGUA_YEAST	21 kDa		5
577	☑	☆	Phenylalanine--tRNA ligase alpha subunit OS=Saccharomyces cerevisiae (strain ATCC 204508 / S288c) OX...	P15625|SYFA_YEAST	58 kDa		5
578	☑	☆	4-aminobutyrate aminotransferase OS=Saccharomyces cerevisiae (strain ATCC 204508 / S288c) OX=559...	P17649|GABAT_YEAST	53 kDa		4
579	☑	☆	Acetylornithine aminotransferase, mitochondrial OS=Saccharomyces cerevisiae (strain ATCC 204508 / S2...	P18544|ARGD_YEAST	47 kDa		6
580	☑	☆	FK506-binding protein 1 OS=Saccharomyces cerevisiae (strain ATCC 204508 / S288c) OX=559292 GN=FPR...	P20081|FKBP_YEAST	12 kDa		5
581	☑	☆	DNA-directed RNA polymerase I subunit RPA135 OS=Saccharomyces cerevisiae (strain ATCC 204508 / S28...	P22138|RPA2_YEAST	136 kDa		6
582	☑	☆	5'-3' exoribonuclease 1 OS=Saccharomyces cerevisiae (strain ATCC 204508 / S288c) OX=559292 GN=XRN...	P22147|XRN1_YEAST	175 kDa		5
583	☑	☆	Thioredoxin-1 OS=Saccharomyces cerevisiae (strain ATCC 204508 / S288c) OX=559292 GN=TRX1 PE=1 SV...	P22217|TRX1_YEAST	11 kDa	★	5
584	☑	☆	High-affinity glucose transporter HXT2 OS=Saccharomyces cerevisiae (strain ATCC 204508 / S288c) OX=5...	P23585|HXT2_YEAST	60 kDa		4
585	☑	☆	Mitochondrial import receptor subunit TOM40 OS=Saccharomyces cerevisiae (strain ATCC 204508 / S288c...	P23644|TOM40_YEAST	42 kDa		5
586	☑	☆	Saccharolysin OS=Saccharomyces cerevisiae (strain ATCC 204508 / S288c) OX=559292 GN=PRD1 PE=1 SV...	P25375|PRTD_YEAST	82 kDa		5
587	☑	☆	Suppressor of yeast profilin deletion OS=Saccharomyces cerevisiae (strain ATCC 204508 / S288c) OX=559...	P25623|SYP1_YEAST	96 kDa		5
588	☑	☆	Myo-inositol transporter 1 OS=Saccharomyces cerevisiae (strain ATCC 204508 / S288c) OX=559292 GN=I...	P30605|ITR1_YEAST	64 kDa		3
589	☑	☆	Protein S100-A7 OS=Homo sapiens OX=9606 GN=S100A7 PE=1 SV=4	P31151|S10A7_HUMAN	11 kDa		4
590	☑	☆	Acyl-CoA-binding protein OS=Saccharomyces cerevisiae (strain ATCC 204508 / S288c) OX=559292 GN=AC...	P31787|ACBP_YEAST	10 kDa		5
591	☑	☆	Actin cytoskeleton-regulatory complex protein PAN1 OS=Saccharomyces cerevisiae (strain ATCC 204508 ...	P32521|PAN1_YEAST	160 kDa		5
592	☑	☆	Alpha-soluble NSF attachment protein OS=Saccharomyces cerevisiae (strain ATCC 204508 / S288c) OX=5...	P32602|SEC17_YEAST	33 kDa		5

Probability Legend: over 95% | 80% to 94% | 50% to 79% | 20% to 49% | 0% to 19%

#	Visible?	Starred?	BioView: 1158 Proteins in 1038 Clusters With 5 Decoys and 22 Hidden	Accession Number	Molecular Weight	Protein Grouping Ambiguity	SrfA_C_CAT
593	☑	☆	Pleiotropic ABC efflux transporter of multiple drugs OS=Saccharomyces cerevisiae (strain ATCC 204508 / ...	P33302|PDR5_YEAST	170 kDa		5
594	☑	☆	Translationally-controlled tumor protein homolog OS=Saccharomyces cerevisiae (strain ATCC 204508 / S...	P35691|TCTP_YEAST	19 kDa		5
595	☑	☆	Vacuolar protein sorting-associated protein 21 OS=Saccharomyces cerevisiae (strain ATCC 204508 / S288...	P36017|VPS21_YEAST	23 kDa	★	5
596	☑	☆	Mitochondrial intermembrane space import and assembly protein 40 OS=Saccharomyces cerevisiae (strai...	P36046|MIA40_YEAST	45 kDa		4
597	☑	☆	Glycerol-3-phosphate O-acyltransferase 2 OS=Saccharomyces cerevisiae (strain ATCC 204508 / S288c) O...	P36148|GPT2_YEAST	84 kDa		5
598	☑	☆	Succinate dehydrogenase [ubiquinone] cytochrome b small subunit, mitochondrial OS=Saccharomyces ce...	P37298|DHSD_YEAST	20 kDa		4
599	☑	☆	Low specificity L-threonine aldolase OS=Saccharomyces cerevisiae (strain ATCC 204508 / S288c) OX=559...	P37303|GLY1_YEAST	43 kDa		5
600	☑	☆	Pyridoxamine 5'-phosphate oxidase OS=Saccharomyces cerevisiae (strain ATCC 204508 / S288c) OX=559...	P38075|PDX3_YEAST	27 kDa		6
601	☑	☆	Probable quinone oxidoreductase OS=Saccharomyces cerevisiae (strain ATCC 204508 / S288c) OX=55929...	P38230|QOR_YEAST	37 kDa		4
⊞ 602	☑	☆	Cluster of Deoxyhypusine synthase OS=Saccharomyces cerevisiae (strain ATCC 204508 / S288c) OX=5592...	P38791|DHYS_YEAST [2]	43 kDa	★	5
603	☑	☆	V-type proton ATPase subunit F OS=Saccharomyces cerevisiae (strain ATCC 204508 / S288c) OX=559292 ...	P39111|VATF_YEAST	13 kDa		4
604	☑	☆	Long-chain-fatty-acid--CoA ligase 2 OS=Saccharomyces cerevisiae (strain ATCC 204508 / S288c) OX=559...	P39518|LCF2_YEAST	83 kDa		4
605	☑	☆	Homocitrate dehydratase, mitochondrial OS=Saccharomyces cerevisiae (strain ATCC 204508 / S288c) OX...	P39533|ACON2_YEAST	87 kDa		5
606	☑	☆	26S proteasome regulatory subunit RPN3 OS=Saccharomyces cerevisiae (strain ATCC 204508 / S288c) OX...	P40016|RPN3_YEAST	60 kDa		5
607	☑	☆	Protein HMF1 OS=Saccharomyces cerevisiae (strain ATCC 204508 / S288c) OX=559292 GN=HMF1 PE=1 SV...	P40037|HMF1_YEAST	14 kDa		4
608	☑	☆	NADPH-dependent 1-acyldihydroxyacetone phosphate reductase OS=Saccharomyces cerevisiae (strain A...	P40471|AYR1_YEAST	33 kDa		5
609	☑	☆	Glutathione S-transferase 1 OS=Saccharomyces cerevisiae (strain ATCC 204508 / S288c) OX=559292 GN=...	P40582|GST1_YEAST	27 kDa		5
610	☑	☆	Peroxisomal acyl-coenzyme A thioester hydrolase 1 OS=Saccharomyces cerevisiae (strain ATCC 204508 /...	P41903|PTE1_YEAST	40 kDa		5
611	☑	☆	Ran-specific GTPase-activating protein 1 OS=Saccharomyces cerevisiae (strain ATCC 204508 / S288c) OX...	P41920|YRB1_YEAST	23 kDa		5
612	☑	☆	Ubiquitin carboxyl-terminal hydrolase 6 OS=Saccharomyces cerevisiae (strain ATCC 204508 / S288c) OX=...	P43593|UBP6_YEAST	57 kDa		5
613	☑	☆	Glycine dehydrogenase (decarboxylating), mitochondrial OS=Saccharomyces cerevisiae (strain ATCC 204...	P49095|GCSP_YEAST	114 kDa		5
614	☑	☆	54S ribosomal protein L12, mitochondrial OS=Saccharomyces cerevisiae (strain ATCC 204508 / S288c) OX...	P53163|MNP1_YEAST	21 kDa		4
615	☑	☆	Uncharacterized protein YNL134C OS=Saccharomyces cerevisiae (strain ATCC 204508 / S288c) OX=55929...	P53912|YNN4_YEAST	41 kDa		5
616	☑	☆	D-arabinono-1,4-lactone oxidase OS=Saccharomyces cerevisiae (strain ATCC 204508 / S288c) OX=55929...	P54783|ALO_YEAST	59 kDa		5
617	☑	☆	Gamma-glutamyl phosphate reductase OS=Saccharomyces cerevisiae (strain ATCC 204508 / S288c) OX=...	P54885|PROA_YEAST	50 kDa		4
618	☑	☆	Dermcidin OS=Homo sapiens OX=9606 GN=DCD PE=1 SV=2	P81605|DCD_HUMAN	11 kDa		5
619	☑	☆	Nucleolar protein 3 OS=Saccharomyces cerevisiae (strain ATCC 204508 / S288c) OX=559292 GN=NPL3 PE=...	Q01560|NOP3_YEAST	45 kDa		5
620	☑	☆	Mitochondrial import inner membrane translocase subunit TIM44 OS=Saccharomyces cerevisiae (strain A...	Q01852|TIM44_YEAST	49 kDa		6
621	☑	☆	Hypoxanthine-guanine phosphoribosyltransferase OS=Saccharomyces cerevisiae (strain ATCC 204508 / S...	Q04178|HPRT_YEAST	25 kDa		5
622	☑	☆	Hit family protein 1 OS=Saccharomyces cerevisiae (strain ATCC 204508 / S288c) OX=559292 GN=HNT1 PE...	Q04344|HNT1_YEAST	18 kDa		4
623	☑	☆	Protein transport protein SEC13 OS=Saccharomyces cerevisiae (strain ATCC 204508 / S288c) OX=559292 ...	Q04491|SEC13_YEAST	33 kDa		6
624	☑	☆	Uncharacterized protein YMR315W OS=Saccharomyces cerevisiae (strain ATCC 204508 / S288c) OX=5592...	Q04869|YM94_YEAST	38 kDa		5
625	☑	☆	Protein ERP1 OS=Saccharomyces cerevisiae (strain ATCC 204508 / S288c) OX=559292 GN=ERP1 PE=1 SV=1	Q05359|ERP1_YEAST	25 kDa		5
626	☑	☆	m7GpppX diphosphatase OS=Saccharomyces cerevisiae (strain ATCC 204508 / S288c) OX=559292 GN=DC...	Q06151|DCPS_YEAST	41 kDa		5
627	☑	☆	Uncharacterized protein YPR148C OS=Saccharomyces cerevisiae (strain ATCC 204508 / S288c) OX=55929...	Q06523|YP148_YEAST	49 kDa		5
628	☑	☆	Desmocollin-1 OS=Homo sapiens OX=9606 GN=DSC1 PE=1 SV=2	Q08554|DSC1_HUMAN	100 kDa		4
629	☑	☆	Spermidine synthase OS=Saccharomyces cerevisiae (strain ATCC 204508 / S288c) OX=559292 GN=SPE3 P...	Q12074|SPEE_YEAST	33 kDa		5

Probability Legend: over 95% | 80% to 94% | 50% to 79% | 20% to 49% | 0% to 19%

#	Visible?	Starred?	BioView: 1158 Proteins in 1038 Clusters With 5 Decoys and 22 Hidden	Accession Number	Molecular Weight	Protein Grouping Ambiguity	SrfA_C_CAT
630	☑	☆	Ammonia transport outward protein 3 OS=Saccharomyces cerevisiae (strain ATCC 204508 / S288c) OX=5...	Q12359|ATO3_YEAST	30 kDa		5
631	☑	☆	Histone H2A.1 OS=Saccharomyces cerevisiae (strain ATCC 204508 / S288c) OX=559292 GN=HTA1 PE=1 SV...	P04911|H2A1_YEAST (+4)	14 kDa	★	4
632	☑	☆	Oxysterol-binding protein homolog 4 OS=Saccharomyces cerevisiae (strain ATCC 204508 / S288c) OX=55...	P35844|KES1_YEAST	49 kDa		4
633	☑	☆	40S ribosomal protein S27-A OS=Saccharomyces cerevisiae (strain ATCC 204508 / S288c) OX=559292 GN...	P35997|RS27A_YEAST (+1)	9 kDa		4
634	☑	☆	Mitochondrial import receptor subunit TOM22 OS=Saccharomyces cerevisiae (strain ATCC 204508 / S288c...	P49334|TOM22_YEAST	17 kDa		3
635	☑	☆	ARS-binding factor 2, mitochondrial OS=Saccharomyces cerevisiae (strain ATCC 204508 / S288c) OX=559...	Q02486|ABF2_YEAST	22 kDa		3
636	☑	☆	Protein MRH1 OS=Saccharomyces cerevisiae (strain ATCC 204508 / S288c) OX=559292 GN=MRH1 PE=1 S...	Q12117|MRH1_YEAST	36 kDa	★	3
637	☑	☆	Myosin-5 OS=Saccharomyces cerevisiae (strain YJM789) OX=307796 GN=MYO5 PE=3 SV=1	A6ZMG6|MYO5_YEAS7 (+1)	137 kDa		5
638	☑	☆	ATP-dependent RNA helicase DBP5 OS=Saccharomyces cerevisiae (strain YJM789) OX=307796 GN=DBP5 P...	A6ZNQ1|DBP5_YEAS7 (+1)	54 kDa		5
639	☑	☆	Signal transduction protein MDG1 OS=Saccharomyces cerevisiae (strain YJM789) OX=307796 GN=MDG1 P...	A6ZRR2|MDG1_YEAS7 (+4)	40 kDa		3
640	☑	☆	Phosphatidylethanolamine N-methyltransferase OS=Saccharomyces cerevisiae (strain YJM789) OX=3077...	A6ZUG8|CHO2_YEAS7 (+4)	101 kDa		4
641	☑	☆	ATP-dependent RNA helicase DHH1 OS=Saccharomyces cerevisiae (strain YJM789) OX=307796 GN=DHH1 ...	A6ZXG9|DHH1_YEAS7 (+1)	58 kDa		4
642	☑	☆	ATPase GET3 OS=Saccharomyces cerevisiae (strain YJM789) OX=307796 GN=GET3 PE=3 SV=1	A6ZXM9|GET3_YEAS7 (+2)	39 kDa		4
643	☑	☆	Vacuolar protein sorting/targeting protein PEP1 OS=Saccharomyces cerevisiae (strain RM11-1a) OX=285...	B3LNF5|VPS10_YEAS1 (+3)	178 kDa		4
644	☑	☆	RNA polymerase II degradation factor 1 OS=Saccharomyces cerevisiae (strain JAY291) OX=574961 GN=D...	C7GP20|DEF1_YEAS2 (+1)	84 kDa		3
645	☑	☆	ATP phosphoribosyltransferase OS=Saccharomyces cerevisiae (strain ATCC 204508 / S288c) OX=559292 ...	P00498|HIS1_YEAST	32 kDa		4
646	☑	☆	Arginase OS=Saccharomyces cerevisiae (strain ATCC 204508 / S288c) OX=559292 GN=CAR1 PE=1 SV=1	P00812|ARGI_YEAST	36 kDa		4
647	☑	☆	60S acidic ribosomal protein P1-alpha OS=Saccharomyces cerevisiae (strain ATCC 204508 / S288c) OX=55...	P05318|RLA1_YEAST	11 kDa		5
648	☑	☆	Eukaryotic peptide chain release factor GTP-binding subunit OS=Saccharomyces cerevisiae (strain ATCC 2...	P05453|ERF3_YEAST	77 kDa		4
649	☑	☆	Calmodulin OS=Saccharomyces cerevisiae (strain ATCC 204508 / S288c) OX=559292 GN=CMD1 PE=1 SV=1	P06787|CALM_YEAST	16 kDa		3
650	☑	☆	Tubulin alpha-1 chain OS=Saccharomyces cerevisiae (strain ATCC 204508 / S288c) OX=559292 GN=TUB1 ...	P09733|TBA1_YEAST	50 kDa		4
651	☑	☆	Ran GTPase-activating protein 1 OS=Saccharomyces cerevisiae (strain ATCC 204508 / S288c) OX=559292...	P11745|RNA1_YEAST	46 kDa		5
652	☑	☆	Phospho-2-dehydro-3-deoxyheptonate aldolase, phenylalanine-inhibited OS=Saccharomyces cerevisiae (...	P14843|AROF_YEAST	41 kDa	★	4
653	☑	☆	Peroxisomal catalase A OS=Saccharomyces cerevisiae (strain ATCC 204508 / S288c) OX=559292 GN=CTA...	P15202|CATA_YEAST	59 kDa		4
654	☑	☆	General transcriptional corepressor TUP1 OS=Saccharomyces cerevisiae (strain ATCC 204508 / S288c) OX...	P16649|TUP1_YEAST	78 kDa		4
655	☑	☆	Glutaredoxin-2 OS=Saccharomyces cerevisiae (strain ATCC 204508 / S288c) OX=559292 GN=GRX2 PE=1 S...	P17695|GLRX2_YEAST	16 kDa		4
656	☑	☆	Probable proteasome subunit alpha type-7 OS=Saccharomyces cerevisiae (strain ATCC 204508 / S288c) O...	P21242|PSA7_YEAST	32 kDa		4
657	☑	☆	ATP synthase subunit epsilon, mitochondrial OS=Saccharomyces cerevisiae (strain ATCC 204508 / S288c) ...	P21306|ATP5E_YEAST	7 kDa		3
658	☑	☆	Ubiquitin-conjugating enzyme E2 1 OS=Saccharomyces cerevisiae (strain ATCC 204508 / S288c) OX=5592...	P21734|UBC1_YEAST	24 kDa		4
659	☑	☆	Proteasome subunit beta type-4 OS=Saccharomyces cerevisiae (strain ATCC 204508 / S288c) OX=559292...	P22141|PSB4_YEAST	23 kDa		3
660	☑	☆	Proteasome subunit beta type-6 OS=Saccharomyces cerevisiae (strain ATCC 204508 / S288c) OX=559292...	P23724|PSB6_YEAST	27 kDa		4
661	☑	☆	Zinc-alpha-2-glycoprotein OS=Homo sapiens OX=9606 GN=AZGP1 PE=1 SV=2	P25311|ZA2G_HUMAN	34 kDa		6
662	☑	☆	Reduced viability upon starvation protein 161 OS=Saccharomyces cerevisiae (strain ATCC 204508 / S288c...	P25343|RV161_YEAST	30 kDa		4
663	☑	☆	Dihydroorotate dehydrogenase (fumarate) OS=Saccharomyces cerevisiae (strain ATCC 204508 / S288c) ...	P28272|PYRD_YEAST	35 kDa		4
664	☑	☆	Co-chaperone protein SBA1 OS=Saccharomyces cerevisiae (strain ATCC 204508 / S288c) OX=559292 GN=...	P28707|SBA1_YEAST	24 kDa		4
665	☑	☆	Exportin-1 OS=Saccharomyces cerevisiae (strain ATCC 204508 / S288c) OX=559292 GN=CRM1 PE=1 SV=1	P30822|XPO1_YEAST	124 kDa		4
666	☑	☆	Dolichyl-phosphate-mannose--protein mannosyltransferase 2 OS=Saccharomyces cerevisiae (strain ATC...	P31382|PMT2_YEAST	87 kDa		4

Probability Legend: over 95% | 80% to 94% | 50% to 79% | 20% to 49% | 0% to 19%

#	Visible?	Starred?	BioView: 1158 Proteins in 1038 Clusters With 5 Decoys and 22 Hidden	Accession Number	Molecular Weight	Protein Grouping Ambiguity	SrfA_C_CAT
667	☑	☆	Caspase-14 OS=Homo sapiens OX=9606 GN=CASP14 PE=1 SV=2	P31944|CASPE_HUMAN	28 kDa		4
668	☑	☆	ATP-dependent bile acid permease OS=Saccharomyces cerevisiae (strain ATCC 204508 / S288c) OX=5592...	P32386|YBT1_YEAST	189 kDa		4
669	☑	☆	Cell division control protein 3 OS=Saccharomyces cerevisiae (strain ATCC 204508 / S288c) OX=559292 GN...	P32457|CDC3_YEAST	60 kDa		4
670	☑	☆	Cell wall assembly regulator SMI1 OS=Saccharomyces cerevisiae (strain ATCC 204508 / S288c) OX=55929...	P32566|SMI1_YEAST	57 kDa		4
671	☑	☆	Peroxisomal 2,4-dienoyl-CoA reductase SPS19 OS=Saccharomyces cerevisiae (strain ATCC 204508 / S288...	P32573|SPS19_YEAST	31 kDa		4
672	☑	☆	Mitochondrial genome maintenance protein MGM101 OS=Saccharomyces cerevisiae (strain ATCC 204508 ...	P32787|MG101_YEAST	30 kDa		4
673	☑	☆	Endosomal protein P24B OS=Saccharomyces cerevisiae (strain ATCC 204508 / S288c) OX=559292 GN=EM...	P32803|EMP24_YEAST	23 kDa		4
674	☑	☆	GTP-binding nuclear protein GSP1/CNR1 OS=Saccharomyces cerevisiae (strain ATCC 204508 / S288c) OX=...	P32835|GSP1_YEAST (+1)	25 kDa		4
675	☑	☆	Mitochondrial presequence protease OS=Saccharomyces cerevisiae (strain ATCC 204508 / S288c) OX=55...	P32898|CYM1_YEAST	112 kDa		4
676	☑	☆	Dolichyl-diphosphooligosaccharide--protein glycosyltransferase subunit WBP1 OS=Saccharomyces cerevi...	P33767|OSTB_YEAST	49 kDa		4
677	☑	☆	Dolichyl-phosphate-mannose--protein mannosyltransferase 1 OS=Saccharomyces cerevisiae (strain ATC...	P33775|PMT1_YEAST	93 kDa		4
678	☑	☆	Mitochondrial import receptor subunit TOM20 OS=Saccharomyces cerevisiae (strain ATCC 204508 / S288c...	P35180|TOM20_YEAST	20 kDa		4
679	☑	☆	Endoplasmic reticulum transmembrane protein 1 OS=Saccharomyces cerevisiae (strain ATCC 204508 / S2...	P35723|YET1_YEAST	23 kDa		4
680	☑	☆	Alanine/arginine aminopeptidase OS=Saccharomyces cerevisiae (strain ATCC 204508 / S288c) OX=55929...	P37898|AAP1_YEAST	98 kDa	★	4
681	☑	☆	Probable mannosyltransferase KTR3 OS=Saccharomyces cerevisiae (strain ATCC 204508 / S288c) OX=559...	P38130|KTR3_YEAST	47 kDa		4
682	☑	☆	Actin-related protein 2/3 complex subunit 1 OS=Saccharomyces cerevisiae (strain ATCC 204508 / S288c) ...	P38328|ARPC1_YEAST	42 kDa		4
683	☑	☆	Eukaryotic translation initiation factor 5 OS=Saccharomyces cerevisiae (strain ATCC 204508 / S288c) OX=...	P38431|IF5_YEAST	45 kDa		4
684	☑	☆	GrpE protein homolog, mitochondrial OS=Saccharomyces cerevisiae (strain ATCC 204508 / S288c) OX=559...	P38523|GRPE_YEAST	26 kDa		3
685	☑	☆	Proteasome subunit beta type-1 OS=Saccharomyces cerevisiae (strain ATCC 204508 / S288c) OX=559292...	P38624|PSB1_YEAST	24 kDa		4
686	☑	☆	NADPH-dependent aldose reductase GRE3 OS=Saccharomyces cerevisiae (strain ATCC 204508 / S288c) OX...	P38715|GRE3_YEAST	37 kDa		4
687	☑	☆	10 kDa heat shock protein, mitochondrial OS=Saccharomyces cerevisiae (strain ATCC 204508 / S288c) OX...	P38910|CH10_YEAST	11 kDa		4
688	☑	☆	Mitochondrial GTP/GDP carrier protein 1 OS=Saccharomyces cerevisiae (strain ATCC 204508 / S288c) OX=...	P38988|GGC1_YEAST	33 kDa		4
689	☑	☆	Protein AIM2 OS=Saccharomyces cerevisiae (strain ATCC 204508 / S288c) OX=559292 GN=AIM2 PE=1 SV...	P39721|AIM2_YEAST	27 kDa		4
690	☑	☆	Plasma membrane iron permease OS=Saccharomyces cerevisiae (strain ATCC 204508 / S288c) OX=55929...	P40088|FTR1_YEAST	46 kDa		4
691	☑	☆	Uncharacterized protein YNL208W OS=Saccharomyces cerevisiae (strain ATCC 204508 / S288c) OX=55929...	P40159|YNU8_YEAST	20 kDa		4
692	☑	☆	Serine palmitoyltransferase 2 OS=Saccharomyces cerevisiae (strain ATCC 204508 / S288c) OX=559292 G...	P40970|LCB2_YEAST	63 kDa		3
693	☑	☆	Ubiquitin carboxyl-terminal hydrolase RPN11 OS=Saccharomyces cerevisiae (strain ATCC 204508 / S288c)...	P43588|RPN11_YEAST	34 kDa		4
694	☑	☆	Beta-glucosidase-like protein NCA3, mitochondrial OS=Saccharomyces cerevisiae (strain ATCC 204508 / S...	P46955|NCA3_YEAST	35 kDa	★	4
695	☑	☆	Cell wall protein YJL171C OS=Saccharomyces cerevisiae (strain ATCC 204508 / S288c) OX=559292 GN=YJL...	P46992|YJR1_YEAST	43 kDa		4
696	☑	☆	Maintenance of telomere capping protein 1 OS=Saccharomyces cerevisiae (strain ATCC 204508 / S288c) O...	P47018|MTC1_YEAST	53 kDa		4
697	☑	☆	Cell wall protein PRY3 OS=Saccharomyces cerevisiae (strain ATCC 204508 / S288c) OX=559292 GN=PRY3 ...	P47033|PRY3_YEAST	89 kDa	★	4
698	☑	☆	Vacuolar transporter chaperone 4 OS=Saccharomyces cerevisiae (strain ATCC 204508 / S288c) OX=55929...	P47075|VTC4_YEAST	83 kDa		4
699	☑	☆	CAAX prenyl protease 1 OS=Saccharomyces cerevisiae (strain ATCC 204508 / S288c) OX=559292 GN=STE...	P47154|STE24_YEAST	52 kDa		4
700	☑	☆	Serpin B4 OS=Homo sapiens OX=9606 GN=SERPINB4 PE=1 SV=2	P48594|SPB4_HUMAN	45 kDa		4
701	☑	☆	V-type proton ATPase subunit G OS=Saccharomyces cerevisiae (strain ATCC 204508 / S288c) OX=559292 ...	P48836|VATG_YEAST	13 kDa		3
702	☑	☆	60S ribosomal protein L38 OS=Saccharomyces cerevisiae (strain ATCC 204508 / S288c) OX=559292 GN=R...	P49167|RL38_YEAST	9 kDa		3
703	☑	☆	Prohibitin-2 OS=Saccharomyces cerevisiae (strain ATCC 204508 / S288c) OX=559292 GN=PHB2 PE=1 SV=2	P50085|PHB2_YEAST	34 kDa		4

Probability Legend

- over 95%
- 80% to 94%
- 50% to 79%
- 20% to 49%
- 0% to 19%

#	Visible?	Starred?	BioView: 1158 Proteins in 1038 Clusters With 5 Decoys and 22 Hidden	Accession Number	Molecular Weight	Protein Grouping Ambiguity	SrfA_C_CAT
704	☑	☆	Cell wall synthesis protein KNH1 OS=Saccharomyces cerevisiae (strain ATCC 204508 / S288c) OX=559292 ...	P50112|KNH1_YEAST	30 kDa		3
705	☑	☆	Sterol-4-alpha-carboxylate 3-dehydrogenase, decarboxylating OS=Saccharomyces cerevisiae (strain ATC...	P53199|ERG26_YEAST	39 kDa		4
706	☑	☆	ER-derived vesicles protein ERV29 OS=Saccharomyces cerevisiae (strain ATCC 204508 / S288c) OX=55929...	P53337|ERV29_YEAST	35 kDa		4
707	☑	☆	Prenylated Rab acceptor 1 OS=Saccharomyces cerevisiae (strain ATCC 204508 / S288c) OX=559292 GN=Y...	P53633|PRA1_YEAST	19 kDa		3
708	☑	☆	GTP-binding protein RHO5 OS=Saccharomyces cerevisiae (strain ATCC 204508 / S288c) OX=559292 GN=R...	P53879|RHO5_YEAST	37 kDa	★	4
709	☑	☆	Aldehyde dehydrogenase [NAD(P)+] 2 OS=Saccharomyces cerevisiae (strain ATCC 204508 / S288c) OX=5...	P54114|ALDH3_YEAST	55 kDa		4
710	☑	☆	Translation machinery-associated protein 20 OS=Saccharomyces cerevisiae (strain ATCC 204508 / S288c)...	P89886|TMA20_YEAST	20 kDa		4
711	☑	☆	Nuclear migration protein NUM1 OS=Saccharomyces cerevisiae (strain ATCC 204508 / S288c) OX=559292 ...	Q00402|NUM1_YEAST	313 kDa		4
712	☑	☆	ADP-ribose pyrophosphatase OS=Saccharomyces cerevisiae (strain ATCC 204508 / S288c) OX=559292 GN...	Q01976|ADPP_YEAST	26 kDa		3
713	☑	☆	Mitochondrial import inner membrane translocase subunit TIM50 OS=Saccharomyces cerevisiae (strain A...	Q02776|TIM50_YEAST	55 kDa		4
714	☑	☆	High mobility group protein 1 OS=Saccharomyces cerevisiae (strain ATCC 204508 / S288c) OX=559292 GN...	Q03973|HMO1_YEAST	28 kDa		3
715	☑	☆	26S proteasome regulatory subunit RPN7 OS=Saccharomyces cerevisiae (strain ATCC 204508 / S288c) OX...	Q06103|RPN7_YEAST	49 kDa		5
716	☑	☆	Protein HBT1 OS=Saccharomyces cerevisiae (strain ATCC 204508 / S288c) OX=559292 GN=HBT1 PE=1 SV=1	Q07653|HBT1_YEAST	114 kDa		4
717	☑	☆	Peroxisomal membrane protein PMP27 OS=Saccharomyces cerevisiae (strain ATCC 204508 / S288c) OX=5...	Q12462|PEX11_YEAST	27 kDa		4
718	☑	☆	Translation machinery-associated protein 17 OS=Saccharomyces cerevisiae (strain ATCC 204508 / S288c)...	Q12513|TMA17_YEAST	17 kDa		4
719	☑	☆	Mitochondrial 2-oxodicarboxylate carrier 2 OS=Saccharomyces cerevisiae (strain ATCC 204508 / S288c) O...	Q99297|ODC2_YEAST	34 kDa		4
720	☑	☆	GTP-binding protein RHO1 OS=Candida albicans (strain SC5314 / ATCC MYA-2876) OX=237561 GN=RHO1 P...	O42825|RHO1_CANAL (+2)	22 kDa	★	4
721	☑	☆	Nucleosome assembly protein OS=Saccharomyces cerevisiae (strain ATCC 204508 / S288c) OX=559292 G...	P25293|NAP1_YEAST	48 kDa		3
722	☑	☆	26S proteasome regulatory subunit RPN12 OS=Saccharomyces cerevisiae (strain ATCC 204508 / S288c) O...	P32496|RPN12_YEAST	32 kDa		3
723	☑	☆	Pore membrane protein of 33 kDa OS=Saccharomyces cerevisiae (strain ATCC 204508 / S288c) OX=55929...	Q12164|POM33_YEAST	32 kDa		4
724	☑	☆	Uncharacterized protein YNR034W-A OS=Saccharomyces cerevisiae (strain ATCC 204508 / S288c) OX=55...	Q3E841|YN034_YEAST	11 kDa		3
725	☑	☆	Sphingosine-1-phosphate lyase OS=Saccharomyces cerevisiae (strain ATCC 204508 / S288c) OX=559292 ...	Q05567|SGPL_YEAST	66 kDa		4
726	☑	☆	25S rRNA (cytosine(2870)-C(5))-methyltransferase OS=Saccharomyces cerevisiae (strain ATCC 204508 / ...	P40991|NOP2_YEAST	70 kDa		4
727	☑	☆	26S proteasome regulatory subunit 7 homolog OS=Saccharomyces cerevisiae (strain ATCC 204508 / S288...	P33299|PRS7_YEAST	52 kDa	★	4
728	☑	☆	Synaptobrevin homolog YKT6 OS=Saccharomyces cerevisiae (strain ATCC 204508 / S288c) OX=559292 GN...	P36015|YKT6_YEAST	23 kDa		3
729	☑	☆	Probable pyridoxal 5'-phosphate synthase subunit SNZ2 OS=Saccharomyces cerevisiae (strain ATCC 2045...	P53824|SNZ2_YEAST	32 kDa		3
730	☑	☆	MICOS complex subunit MIC10 OS=Saccharomyces cerevisiae (strain ATCC 204508 / S288c) OX=559292 G...	Q96VH5|MIC10_YEAST	10 kDa		3
731	☑	☆	Ras-like protein 2 OS=Saccharomyces cerevisiae (strain ATCC 204508 / S288c) OX=559292 GN=RAS2 PE=...	P01120|RAS2_YEAST	35 kDa	★	4
732	☑	☆	Eukaryotic translation initiation factor 2 subunit alpha OS=Saccharomyces cerevisiae (strain ATCC 204508...	P20459|IF2A_YEAST	35 kDa		4
733	☑	☆	40S ribosomal protein S23-A OS=Saccharomyces cerevisiae (strain ATCC 204508 / S288c) OX=559292 GN...	P0CX29|RS23A_YEAST (+5)	16 kDa		4
734	☑	☆	Histone H2B.1 OS=Saccharomyces cerevisiae (strain ATCC 204508 / S288c) OX=559292 GN=HTB1 PE=1 SV...	P02293|H2B1_YEAST (+3)	14 kDa		3
735	☑	☆	Uncharacterized phosphatase YNL010W OS=Saccharomyces cerevisiae (strain ATCC 204508 / S288c) OX=...	P53981|YNB0_YEAST	27 kDa		3
736	☑	☆	Peptidyl-prolyl cis-trans isomerase CPR6 OS=Saccharomyces cerevisiae (strain ATCC 204508 / S288c) OX...	P53691|PPID_YEAST	42 kDa	★	3
737	☑	☆	Transcription initiation factor TFIID subunit 14 OS=Saccharomyces cerevisiae (strain ATCC 204508 / S288...	P35189|TAF14_YEAST	27 kDa		3
738	☑	☆	Protein YRO2 OS=Saccharomyces cerevisiae (strain ATCC 204508 / S288c) OX=559292 GN=YRO2 PE=1 SV...	P38079|YRO2_YEAST	39 kDa	★	3
739	☑	☆	Single-stranded DNA-binding protein RIM1, mitochondrial OS=Saccharomyces cerevisiae (strain ATCC 204...	P32445|RIM1_YEAST	15 kDa		2
740	☑	☆	Pyruvate kinase OS=Neurospora crassa (strain ATCC 24698 / 74-OR23-1A / CBS 708.71 / DSM 1257 / FGSC...	Q7RVA8|KPYK_NEUCR	58 kDa	★	4

Probability Legend

- over 95%
- 80% to 94%
- 50% to 79%
- 20% to 49%
- 0% to 19%

#	Visible?	Starred?	BioView: 1158 Proteins in 1038 Clusters With 5 Decoys and 22 Hidden	Accession Number	Molecular Weight	Protein Grouping Ambiguity	SrfA_C_CAT
741	☑	☆	Citrate synthase, mitochondrial OS=Aspergillus niger OX=5061 GN=cit-1 PE=2 SV=1	P51044|CISY_ASPNG	52 kDa	★	3
742	☑	☆	Glyceraldehyde-3-phosphate dehydrogenase OS=Borrelia burgdorferi (strain ATCC 35210 / B31 / CIP 1025...	P46795|G3P_BORBU	36 kDa	★	3
743	☑	☆	Protein SDS24 OS=Saccharomyces cerevisiae (strain YJM789) OX=307796 GN=SDS24 PE=3 SV=1	A6ZLF4|SDS24_YEAS7 (+1)	57 kDa		3
744	☑	☆	IML2-like protein SCY_3392 OS=Saccharomyces cerevisiae (strain YJM789) OX=307796 GN=SCY_3392 PE...	A6ZZY2|YKR18_YEAS7 (+1)	82 kDa		3
745	☑	☆	LAS seventeen-binding protein 3 OS=Saccharomyces cerevisiae (strain YJM789) OX=307796 GN=LSB3 PE...	A7A261|LSB3_YEAS7 (+1)	49 kDa		3
746	☑	☆	Histidinol-phosphate aminotransferase OS=Saccharomyces cerevisiae (strain ATCC 204508 / S288c) OX=...	P07172|HIS8_YEAST	43 kDa		3
747	☑	☆	40S ribosomal protein S25-B OS=Saccharomyces cerevisiae (strain ATCC 204508 / S288c) OX=559292 GN...	P0C0T4|RS25B_YEAST (+2)	12 kDa		3
748	☑	☆	Cluster of Protein COS2 OS=Saccharomyces cerevisiae (strain ATCC 204508 / S288c) OX=559292 GN=COS...	P0CX12|COS2_YEAST [3]	45 kDa	★	3
749	☑	☆	60S acidic ribosomal protein P1-beta OS=Saccharomyces cerevisiae (strain ATCC 204508 / S288c) OX=55...	P10622|RLA3_YEAST	11 kDa		4
750	☑	☆	Purine-cytosine permease FCY2 OS=Saccharomyces cerevisiae (strain ATCC 204508 / S288c) OX=559292 ...	P17064|FCY2_YEAST	58 kDa		3
751	☑	☆	General amino-acid permease GAP1 OS=Saccharomyces cerevisiae (strain ATCC 204508 / S288c) OX=559...	P19145|GAP1_YEAST	66 kDa		3
752	☑	☆	Filaggrin OS=Homo sapiens OX=9606 GN=FLG PE=1 SV=3	P20930|FILA_HUMAN	435 kDa		3
753	☑	☆	Protein CBP3, mitochondrial OS=Saccharomyces cerevisiae (strain ATCC 204508 / S288c) OX=559292 GN=...	P21560|CBP3_YEAST	39 kDa		3
754	☑	☆	Protein SLY1 OS=Saccharomyces cerevisiae (strain ATCC 204508 / S288c) OX=559292 GN=SLY1 PE=1 SV=1	P22213|SLY1_YEAST	75 kDa		3
755	☑	☆	Serine palmitoyltransferase 1 OS=Saccharomyces cerevisiae (strain ATCC 204508 / S288c) OX=559292 G...	P25045|LCB1_YEAST	62 kDa		3
756	☑	☆	Proteasome subunit alpha type-5 OS=Saccharomyces cerevisiae (strain ATCC 204508 / S288c) OX=55929...	P32379|PSA5_YEAST	29 kDa		4
757	☑	☆	Mitogen-activated protein kinase HOG1 OS=Saccharomyces cerevisiae (strain ATCC 204508 / S288c) OX=...	P32485|HOG1_YEAST	49 kDa		3
758	☑	☆	Beta-glucan synthesis-associated protein KRE6 OS=Saccharomyces cerevisiae (strain ATCC 204508 / S28...	P32486|KRE6_YEAST	80 kDa		3
759	☑	☆	UV excision repair protein RAD23 OS=Saccharomyces cerevisiae (strain ATCC 204508 / S288c) OX=55929...	P32628|RAD23_YEAST	42 kDa		3
760	☑	☆	Ferric/cupric reductase transmembrane component 1 OS=Saccharomyces cerevisiae (strain ATCC 204508...	P32791|FRE1_YEAST	79 kDa		3
761	☑	☆	Mitochondrial inner membrane i-AAA protease supercomplex subunit YME1 OS=Saccharomyces cerevisia...	P32795|YME1_YEAST	82 kDa		3
762	☑	☆	Eukaryotic translation initiation factor eIF-1 OS=Saccharomyces cerevisiae (strain ATCC 204508 / S288c) ...	P32911|SUI1_YEAST	12 kDa		3
763	☑	☆	Probable 1-acyl-sn-glycerol-3-phosphate acyltransferase OS=Saccharomyces cerevisiae (strain ATCC 204...	P33333|PLSC_YEAST	34 kDa		3
764	☑	☆	Protein SLA2 OS=Saccharomyces cerevisiae (strain ATCC 204508 / S288c) OX=559292 GN=SLA2 PE=1 SV=5	P33338|SLA2_YEAST	109 kDa		3
765	☑	☆	UBX domain-containing protein 1 OS=Saccharomyces cerevisiae (strain ATCC 204508 / S288c) OX=559292...	P34223|UBX1_YEAST	47 kDa		3
766	☑	☆	54S ribosomal protein L11, mitochondrial OS=Saccharomyces cerevisiae (strain ATCC 204508 / S288c) OX...	P36521|RM11_YEAST	29 kDa		3
767	☑	☆	Putative serine carboxypeptidase YBR139W OS=Saccharomyces cerevisiae (strain ATCC 204508 / S288c) ...	P38109|YBY9_YEAST	58 kDa		3
768	☑	☆	ISWI chromatin-remodeling complex ATPase ISW1 OS=Saccharomyces cerevisiae (strain ATCC 204508 / ...	P38144|ISW1_YEAST	131 kDa		3
769	☑	☆	Golgi SNAP receptor complex member 1 OS=Saccharomyces cerevisiae (strain ATCC 204508 / S288c) OX=...	P38736|GOSR1_YEAST	25 kDa		3
770	☑	☆	Glutamine-dependent NAD(+) synthetase OS=Saccharomyces cerevisiae (strain ATCC 204508 / S288c) OX...	P38795|NADE_YEAST	81 kDa		3
771	☑	☆	Aspartyl aminopeptidase 4 OS=Saccharomyces cerevisiae (strain ATCC 204508 / S288c) OX=559292 GN=...	P38821|DNPEP_YEAST	54 kDa		3
772	☑	☆	26S proteasome regulatory subunit RPN10 OS=Saccharomyces cerevisiae (strain ATCC 204508 / S288c) O...	P38886|RPN10_YEAST	30 kDa		3
773	☑	☆	Alpha-1,3-mannosyltransferase MNN1 OS=Saccharomyces cerevisiae (strain ATCC 204508 / S288c) OX=5...	P39106|MNN1_YEAST	89 kDa		4
774	☑	☆	Reduced viability upon starvation protein 167 OS=Saccharomyces cerevisiae (strain ATCC 204508 / S288c...	P39743|RV167_YEAST	53 kDa		3
775	☑	☆	Carnitine O-acetyltransferase YAT2 OS=Saccharomyces cerevisiae (strain ATCC 204508 / S288c) OX=559...	P40017|YAT2_YEAST	103 kDa		3
776	☑	☆	Mitochondrial acidic protein MAM33 OS=Saccharomyces cerevisiae (strain ATCC 204508 / S288c) OX=559...	P40513|MAM33_YEAST	30 kDa		3
777	☑	☆	Protein SCD6 OS=Saccharomyces cerevisiae (strain ATCC 204508 / S288c) OX=559292 GN=SCD6 PE=1 SV=1	P45978|SCD6_YEAST	39 kDa		3

Probability Legend

- over 95%
- 80% to 94%
- 50% to 79%
- 20% to 49%
- 0% to 19%

#	Visible?	Starred?	BioView: 1158 Proteins in 1038 Clusters With 5 Decoys and 22 Hidden	Accession Number	Molecular Weight	Protein Grouping Ambiguity	SrfA_C_CAT
778	☑	☆	3-hydroxyanthranilate 3,4-dioxygenase OS=Saccharomyces cerevisiae (strain ATCC 204508 / S288c) OX=...	P47096|3HAO_YEAST	20 kDa		3
779	☑	☆	Deoxyhypusine hydroxylase OS=Saccharomyces cerevisiae (strain ATCC 204508 / S288c) OX=559292 GN...	P47120|DOHH_YEAST	36 kDa		3
780	☑	☆	Long-chain-fatty-acid--CoA ligase 4 OS=Saccharomyces cerevisiae (strain ATCC 204508 / S288c) OX=559...	P47912|LCF4_YEAST	77 kDa		3
781	☑	☆	Vacuolar acid trehalase OS=Saccharomyces cerevisiae (strain ATCC 204508 / S288c) OX=559292 GN=ATH...	P48016|ATH1_YEAST	137 kDa		3
782	☑	☆	Nucleoporin NUP57 OS=Saccharomyces cerevisiae (strain ATCC 204508 / S288c) OX=559292 GN=NUP57 PE...	P48837|NUP57_YEAST	57 kDa		3
783	☑	☆	2-methoxy-6-polyprenyl-1,4-benzoquinol methylase, mitochondrial OS=Saccharomyces cerevisiae (strain...	P49017|COQ5_YEAST	35 kDa		3
784	☑	☆	6,7-dimethyl-8-ribityllumazine synthase OS=Saccharomyces cerevisiae (strain ATCC 204508 / S288c) OX...	P50861|RIB4_YEAST	19 kDa		3
785	☑	☆	Protein SUR7 OS=Saccharomyces cerevisiae (strain ATCC 204508 / S288c) OX=559292 GN=SUR7 PE=1 SV...	P54003|SUR7_YEAST	34 kDa		3
786	☑	☆	Uncharacterized protein YMR196W OS=Saccharomyces cerevisiae (strain ATCC 204508 / S288c) OX=5592...	Q04336|YM54_YEAST	127 kDa		3
787	☑	☆	Survival factor 1 OS=Saccharomyces cerevisiae (strain ATCC 204508 / S288c) OX=559292 GN=SVF1 PE=1 ...	Q05515|SVF1_YEAST	54 kDa		4
788	☑	☆	Polyamine transporter 3 OS=Saccharomyces cerevisiae (strain ATCC 204508 / S288c) OX=559292 GN=TP...	Q06451|TPO3_YEAST	68 kDa		3
789	☑	☆	Nucleolar complex-associated protein 3 OS=Saccharomyces cerevisiae (strain ATCC 204508 / S288c) OX=...	Q07896|NOC3_YEAST	76 kDa		3
790	☑	☆	Ubiquitin-binding protein CUE5 OS=Saccharomyces cerevisiae (strain ATCC 204508 / S288c) OX=559292 G...	Q08412|CUE5_YEAST	47 kDa		3
791	☑	☆	26S proteasome regulatory subunit RPN8 OS=Saccharomyces cerevisiae (strain ATCC 204508 / S288c) OX...	Q08723|RPN8_YEAST	38 kDa		3
792	☑	☆	Protein PBDC1 homolog OS=Saccharomyces cerevisiae (strain ATCC 204508 / S288c) OX=559292 GN=YPL...	Q08971|YP225_YEAST	17 kDa		4
793	☑	☆	3-keto-steroid reductase OS=Saccharomyces cerevisiae (strain ATCC 204508 / S288c) OX=559292 GN=ER...	Q12452|ERG27_YEAST	40 kDa		3
794	☑	☆	Filaggrin-2 OS=Homo sapiens OX=9606 GN=FLG2 PE=1 SV=1	Q5D862|FILA2_HUMAN	248 kDa		3
795	☑	☆	Cytochrome c oxidase subunit 2 OS=Saccharomyces cerevisiae (strain ATCC 204508 / S288c) OX=559292 ...	P00410|COX2_YEAST	29 kDa		3
796	☑	☆	Orotate phosphoribosyltransferase 1 OS=Saccharomyces cerevisiae (strain ATCC 204508 / S288c) OX=55...	P13298|PYRE_YEAST	25 kDa		3
797	☑	☆	F-actin-capping protein subunit beta OS=Saccharomyces cerevisiae (strain ATCC 204508 / S288c) OX=559...	P13517|CAPZB_YEAST	33 kDa		3
798	☑	☆	Clathrin light chain OS=Saccharomyces cerevisiae (strain ATCC 204508 / S288c) OX=559292 GN=CLC1 PE=...	P17891|CLC1_YEAST	27 kDa		3
799	☑	☆	Casein kinase II subunit alpha' OS=Saccharomyces cerevisiae (strain ATCC 204508 / S288c) OX=559292 G...	P19454|CSK22_YEAST	39 kDa		3
800	☑	☆	Protein kinase MCK1 OS=Saccharomyces cerevisiae (strain ATCC 204508 / S288c) OX=559292 GN=MCK1 P...	P21965|MCK1_YEAST	43 kDa		3
801	☑	☆	NifU-like protein, mitochondrial OS=Saccharomyces cerevisiae (strain ATCC 204508 / S288c) OX=559292 ...	P32860|NFU1_YEAST	29 kDa		3
802	☑	☆	Monothiol glutaredoxin-7 OS=Saccharomyces cerevisiae (strain ATCC 204508 / S288c) OX=559292 GN=G...	P38068|GLRX7_YEAST	23 kDa		3
803	☑	☆	Protein ERP2 OS=Saccharomyces cerevisiae (strain ATCC 204508 / S288c) OX=559292 GN=ERP2 PE=1 SV=1	P39704|ERP2_YEAST	24 kDa		3
804	☑	☆	Tropomyosin-2 OS=Saccharomyces cerevisiae (strain ATCC 204508 / S288c) OX=559292 GN=TPM2 PE=1 ...	P40414|TPM2_YEAST	19 kDa		4
805	☑	☆	Importin subunit beta-1 OS=Saccharomyces cerevisiae (strain ATCC 204508 / S288c) OX=559292 GN=KAP...	Q06142|IMB1_YEAST	95 kDa		3
806	☑	☆	Mitochondrial 2-methylisocitrate lyase OS=Saccharomyces cerevisiae (strain ATCC 204508 / S288c) OX=5...	Q12031|ACEB_YEAST	65 kDa		3
807	☑	☆	FAS1 domain-containing protein YDR262W OS=Saccharomyces cerevisiae (strain ATCC 204508 / S288c) O...	Q12331|YFAS1_YEAST	31 kDa		3
808	☑	☆	L-asparaginase 1 OS=Saccharomyces cerevisiae (strain ATCC 204508 / S288c) OX=559292 GN=ASP1 PE=1...	P38986|ASPG1_YEAST	41 kDa		3
809	☑	☆	Mitochondrial glycine transporter YMC1 OS=Saccharomyces cerevisiae (strain ATCC 204508 / S288c) OX=...	P32331|YMC1_YEAST	33 kDa		3
810	☑	☆	Cytochrome c oxidase subunit 13, mitochondrial OS=Saccharomyces cerevisiae (strain ATCC 204508 / S28...	P32799|COX13_YEAST	15 kDa		3
811	☑	☆	Peroxisomal-coenzyme A synthetase OS=Saccharomyces cerevisiae (strain ATCC 204508 / S288c) OX=55...	P38137|FAT2_YEAST	60 kDa		3
⊞ 812	☑	☆	Cluster of Lon protease homolog, mitochondrial OS=Saccharomyces cerevisiae (strain ATCC 204508 / S28...	P36775|LONM_YEAST [3]	127 kDa	★	3
813	☑	☆	Probable intramembrane protease YKL100C OS=Saccharomyces cerevisiae (strain ATCC 204508 / S288c) ...	P34248|YKK0_YEAST	68 kDa		3
814	☑	☆	Alkylphosphocholine resistance protein LEM3 OS=Saccharomyces cerevisiae (strain ATCC 204508 / S288c)...	P42838|LEM3_YEAST	47 kDa		3

Probability Legend

- over 95%
- 80% to 94%
- 50% to 79%
- 20% to 49%
- 0% to 19%

#	Visible?	Starred?	BioView: 1158 Proteins in 1038 Clusters With 5 Decoys and 22 Hidden	Accession Number	Molecular Weight	Protein Grouping Ambiguity	SrfA_C_CAT
815	☑	☆	Ribose-5-phosphate isomerase OS=Saccharomyces cerevisiae (strain YJM789) OX=307796 GN=RKI1 PE=...	A6ZNU5|RPIA_YEAS7 (+1)	28 kDa		3
816	☑	☆	60S ribosomal protein L39 OS=Saccharomyces cerevisiae (strain ATCC 204508 / S288c) OX=559292 GN=R...	P04650|RL39_YEAST (+9)	6 kDa		3
817	☑	☆	Proteasome subunit beta type-2 OS=Saccharomyces cerevisiae (strain ATCC 204508 / S288c) OX=559292...	P25043|PSB2_YEAST	28 kDa		3
818	☑	☆	Cysteine desulfurase, mitochondrial OS=Saccharomyces cerevisiae (strain ATCC 204508 / S288c) OX=559...	P25374|NFS1_YEAST	54 kDa		3
819	☑	☆	High-affinity methionine permease OS=Saccharomyces cerevisiae (strain ATCC 204508 / S288c) OX=5592...	P50276|MUP1_YEAST	63 kDa		3
820	☑	☆	Protein phosphatase PP2A regulatory subunit A OS=Saccharomyces cerevisiae (strain ATCC 204508 / S28...	P31383|2AAA_YEAST	71 kDa		3
821	☑	☆	Chromatin structure-remodeling complex protein RSC8 OS=Saccharomyces cerevisiae (strain ATCC 20450...	P43609|RSC8_YEAST	63 kDa		3
822	☑	☆	DNA-directed RNA polymerase II subunit RPB1 OS=Saccharomyces cerevisiae (strain ATCC 204508 / S288...	P04050|RPB1_YEAST	192 kDa		4
823	☑	☆	Uncharacterized protein YFR016C OS=Saccharomyces cerevisiae (strain ATCC 204508 / S288c) OX=55929...	P43597|YFI6_YEAST	138 kDa		3
824	☑	☆	Proteasome subunit beta type-7 OS=Saccharomyces cerevisiae (strain ATCC 204508 / S288c) OX=559292...	P30657|PSB7_YEAST	29 kDa		3
825	☑	☆	Cell division control protein 10 OS=Saccharomyces cerevisiae (strain ATCC 204508 / S288c) OX=559292 G...	P25342|CDC10_YEAST	37 kDa		3
826	☑	☆	Ribosome assembly factor MRT4 OS=Saccharomyces cerevisiae (strain ATCC 204508 / S288c) OX=559292...	P33201|MRT4_YEAST	27 kDa		3
827	☑	☆	Putative mitochondrial carnitine O-acetyltransferase OS=Saccharomyces cerevisiae (strain ATCC 204508 ...	P80235|CACM_YEAST	78 kDa		3
828	☑	☆	Actin-like protein ARP8 OS=Saccharomyces cerevisiae (strain ATCC 204508 / S288c) OX=559292 GN=ARP...	Q12386|ARP8_YEAST	100 kDa		3
829	☑	☆	37S ribosomal protein YMR-31, mitochondrial OS=Saccharomyces cerevisiae (strain ATCC 204508 / S288c...	P19955|RT31_YEAST	14 kDa		3
830	☑	☆	Cruciform DNA-recognizing protein 1 OS=Saccharomyces cerevisiae (strain YJM789) OX=307796 GN=CRP1...	A6ZT54|CRP1_YEAS7 (+5)	51 kDa		3
831	☑	☆	ATP synthase subunit g, mitochondrial OS=Saccharomyces cerevisiae (strain ATCC 204508 / S288c) OX=5...	Q12233|ATPN_YEAST	13 kDa		3
832	☑	☆	tRNA (guanine(26)-N(2))-dimethyltransferase, mitochondrial OS=Saccharomyces cerevisiae (strain ATCC ...	P15565|TRM1_YEAST	64 kDa		3
833	☑	☆	Glycine cleavage system H protein, mitochondrial OS=Saccharomyces cerevisiae (strain ATCC 204508 / S2...	P39726|GCSH_YEAST	19 kDa		2
834	☑	☆	Respiratory growth induced protein 1 OS=Saccharomyces cerevisiae (strain YJM789) OX=307796 GN=RGI...	A6ZR21|RGI1_YEAS7 (+4)	19 kDa		2
835	☑	☆	Autophagy-related protein 33 OS=Saccharomyces cerevisiae (strain YJM789) OX=307796 GN=ATG33 PE=...	A7A1N4|ATG33_YEAS7 (+3)	20 kDa		2
836	☑	☆	Cyclin-dependent protein kinase PHO85 OS=Saccharomyces cerevisiae (strain ATCC 204508 / S288c) OX=...	P17157|PHO85_YEAST	35 kDa		2
837	☑	☆	Cell division control protein 42 OS=Saccharomyces cerevisiae (strain ATCC 204508 / S288c) OX=559292 G...	P19073|CDC42_YEAST	21 kDa		2
838	☑	☆	Elongation of fatty acids protein 2 OS=Saccharomyces cerevisiae (strain ATCC 204508 / S288c) OX=55929...	P25358|ELO2_YEAST	40 kDa		2
839	☑	☆	C-8 sterol isomerase OS=Saccharomyces cerevisiae (strain ATCC 204508 / S288c) OX=559292 GN=ERG2 P...	P32352|ERG2_YEAST	25 kDa		2
840	☑	☆	Acyl carrier protein, mitochondrial OS=Saccharomyces cerevisiae (strain ATCC 204508 / S288c) OX=5592...	P32463|ACPM_YEAST	14 kDa		2
841	☑	☆	ATP synthase subunit e, mitochondrial OS=Saccharomyces cerevisiae (strain ATCC 204508 / S288c) OX=5...	P81449|ATPJ_YEAST	11 kDa		2
842	☑	☆	RNA annealing protein YRA1 OS=Saccharomyces cerevisiae (strain ATCC 204508 / S288c) OX=559292 GN=...	Q12159|YRA1_YEAST	25 kDa		2
843	☑	☆	Corneodesmosin OS=Homo sapiens OX=9606 GN=CDSN PE=1 SV=3	Q15517|CDSN_HUMAN (+1)	52 kDa		2
+ 844	☑	☆	Cluster of ATP-dependent RNA helicase DBP3 OS=Saccharomyces cerevisiae (strain YJM789) OX=307796 ...	A6ZUA1|DBP3_YEAS7 [3]	59 kDa	★	3
845	☑	☆	Myosin light chain 1 OS=Saccharomyces cerevisiae (strain ATCC 204508 / S288c) OX=559292 GN=MLC1 PE...	P53141|MLC1_YEAST	16 kDa		3
846	☑	☆	F-actin-capping protein subunit alpha OS=Saccharomyces cerevisiae (strain ATCC 204508 / S288c) OX=55...	P28495|CAPZA_YEAST	31 kDa		2
847	☑	☆	ATP synthase subunit H, mitochondrial OS=Saccharomyces cerevisiae (strain ATCC 204508 / S288c) OX=5...	Q12349|ATP14_YEAST	14 kDa		2
848	☑	☆	Aspartokinase OS=Saccharomyces cerevisiae (strain ATCC 204508 / S288c) OX=559292 GN=HOM3 PE=1 S...	P10869|AK_YEAST	58 kDa		3
+ 849	☑	☆	Cluster of Chaperone protein HscA homolog OS=Laribacter hongkongensis (strain HLHK9) OX=557598 GN=...	C1D4P9|HSCA_LARHH [2]	66 kDa	★	4
850	☑	☆	Phosphatidyl-N-methylethanolamine N-methyltransferase OS=Saccharomyces cerevisiae (strain ATCC 20...	P05375|PLMT_YEAST	23 kDa		2
851	☑	☆	ATP-dependent RNA helicase DBP2 OS=Saccharomyces cerevisiae (strain YJM789) OX=307796 GN=DBP2 P...	A6ZRX0|DBP2_YEAS7 (+1)	61 kDa	★	3

Probability Legend

- over 95%
- 80% to 94%
- 50% to 79%
- 20% to 49%
- 0% to 19%

#	Visible?	Starred?	BioView: 1158 Proteins in 1038 Clusters With 5 Decoys and 22 Hidden	Accession Number	Molecular Weight	Protein Grouping Ambiguity	SrfA_C_CAT
852	☑	☆	FK506-binding protein 4 OS=Saccharomyces cerevisiae (strain ATCC 204508 / S288c) OX=559292 GN=FPR...	Q06205|FKBP4_YEAST	44 kDa	★	3
853	☑	☆	Ras-like protein 1 OS=Saccharomyces cerevisiae (strain ATCC 204508 / S288c) OX=559292 GN=RAS1 PE=...	P01119|RAS1_YEAST	34 kDa	★	3
854	☑	☆	Putative oxidoreductase TDA3 OS=Saccharomyces cerevisiae (strain ATCC 204508 / S288c) OX=559292 G...	P38758|TDA3_YEAST	58 kDa		2
855	☑	☆	Hexokinase OS=Kluyveromyces lactis (strain ATCC 8585 / CBS 2359 / DSM 70799 / NBRC 1267 / NRRL Y-11...	P33284|HXK_KLULA	54 kDa	★	3
856	☑	☆	Outer spore wall assembly protein SHE10 OS=Saccharomyces cerevisiae (strain YJM789) OX=307796 GN=...	A6ZTW2|SHE10_YEAS7 (+3)	67 kDa		3
857	☑	☆	Arginine biosynthesis bifunctional protein ArgJ, mitochondrial OS=Saccharomyces cerevisiae (strain YJM7...	A6ZMC1|ARGJ_YEAS7 (+5)	48 kDa		2
858	☑	☆	Vacuolar transporter chaperone 2 OS=Saccharomyces cerevisiae (strain ATCC 204508 / S288c) OX=55929...	P43585|VTC2_YEAST	95 kDa		3
859	☑	☆	Probable metalloprotease ARX1 OS=Saccharomyces cerevisiae (strain ATCC 204508 / S288c) OX=559292 ...	Q03862|ARX1_YEAST	65 kDa		3
860	☑	☆	37S ribosomal protein S17, mitochondrial OS=Saccharomyces cerevisiae (strain ATCC 204508 / S288c) OX...	Q03246|RT17_YEAST	28 kDa		3
861	☑	☆	DNA-directed RNA polymerases I and III subunit RPAC2 OS=Saccharomyces cerevisiae (strain ATCC 20450...	P28000|RPAC2_YEAST	16 kDa		2
862	☑	☆	Protein CAJ1 OS=Saccharomyces cerevisiae (strain ATCC 204508 / S288c) OX=559292 GN=CAJ1 PE=1 SV=1	P39101|CAJ1_YEAST	45 kDa		2
863	☑	☆	Ribosomal RNA-processing protein 12 OS=Saccharomyces cerevisiae (strain ATCC 204508 / S288c) OX=55...	Q12754|RRP12_YEAST	138 kDa		2
864	☑	☆	S-(hydroxymethyl)glutathione dehydrogenase OS=Saccharomyces cerevisiae (strain ATCC 204508 / S288...	P32771|FADH_YEAST	41 kDa		2
865	☑	☆	Manganese transporter SMF1 OS=Saccharomyces cerevisiae (strain ATCC 204508 / S288c) OX=559292 GN...	P38925|SMF1_YEAST	63 kDa		2
866	☑	☆	Mitochondrial import receptor subunit TOM5 OS=Saccharomyces cerevisiae (strain ATCC 204508 / S288c) ...	P80967|TOM5_YEAST	6 kDa		2
867	☑	☆	Heat shock protein 70 homolog LHS1 OS=Saccharomyces cerevisiae (strain ATCC 204508 / S288c) OX=55...	P36016|LHS1_YEAST	100 kDa		2
868	☑	☆	Phosphatidylserine decarboxylase proenzyme 1, mitochondrial OS=Saccharomyces cerevisiae (strain ATC...	P39006|PSD1_YEAST	57 kDa		2
869	☑	☆	Benzil reductase ((S)-benzoin forming) IRC24 OS=Saccharomyces cerevisiae (strain ATCC 204508 / S288c...	P40580|BZRD_YEAST	29 kDa		2
870	☑	☆	UDP-N-acetylglucosamine pyrophosphorylase OS=Saccharomyces cerevisiae (strain ATCC 204508 / S288c...	P43123|UAP1_YEAST	53 kDa		2
871	☑	☆	Altered inheritance of mitochondria protein 9, mitochondrial OS=Saccharomyces cerevisiae (strain YJM78...	A6ZR36|AIM9_YEAS7 (+4)	72 kDa		2
872	☑	☆	Respiratory growth induced protein 2 OS=Saccharomyces cerevisiae (strain YJM789) OX=307796 GN=RGI...	A6ZVL3|RGI2_YEAS7 (+2)	19 kDa		2
873	☑	☆	60S ribosomal subunit assembly/export protein LOC1 OS=Saccharomyces cerevisiae (strain YJM789) OX=...	A7A239|LOC1_YEAS7 (+1)	24 kDa		2
874	☑	☆	Pheromone alpha factor receptor OS=Saccharomyces cerevisiae (strain ATCC 204508 / S288c) OX=55929...	D6VTK4|STE2_YEAST (+1)	48 kDa		2
875	☑	☆	26S proteasome regulatory subunit RPN13 OS=Saccharomyces cerevisiae (strain ATCC 204508 / S288c) O...	O13563|RPN13_YEAST	18 kDa		2
876	☑	☆	Cytochrome c oxidase subunit 5A, mitochondrial OS=Saccharomyces cerevisiae (strain ATCC 204508 / S28...	P00424|COX5A_YEAST	17 kDa		2
877	☑	☆	Arginase-1 OS=Homo sapiens OX=9606 GN=ARG1 PE=1 SV=2	P05089|ARGI1_HUMAN	35 kDa		2
878	☑	☆	Eukaryotic translation initiation factor 2 subunit beta OS=Saccharomyces cerevisiae (strain ATCC 204508 ...	P09064|IF2B_YEAST	32 kDa		2
879	☑	☆	60S ribosomal protein L43-A OS=Saccharomyces cerevisiae (strain ATCC 204508 / S288c) OX=559292 GN...	P0CX25|RL43A_YEAST (+2)	10 kDa		2
880	☑	☆	Repressible alkaline phosphatase OS=Saccharomyces cerevisiae (strain ATCC 204508 / S288c) OX=55929...	P11491|PPB_YEAST	63 kDa		2
881	☑	☆	Isopentenyl-diphosphate Delta-isomerase OS=Saccharomyces cerevisiae (strain ATCC 204508 / S288c) O...	P15496|IDI1_YEAST	33 kDa		2
882	☑	☆	Ferrochelatase, mitochondrial OS=Saccharomyces cerevisiae (strain ATCC 204508 / S288c) OX=559292 G...	P16622|HEMH_YEAST	45 kDa		2
883	☑	☆	Zinc/cadmium resistance protein OS=Saccharomyces cerevisiae (strain ATCC 204508 / S288c) OX=55929...	P20107|ZRC1_YEAST	48 kDa		2
884	☑	☆	Ribonucleoside-diphosphate reductase large chain 1 OS=Saccharomyces cerevisiae (strain ATCC 204508 /...	P21524|RIR1_YEAST	100 kDa		2
885	☑	☆	Probable oxidoreductase AIM17 OS=Saccharomyces cerevisiae (strain ATCC 204508 / S288c) OX=559292 ...	P23180|AIM17_YEAST	53 kDa		2
886	☑	☆	Ectonucleotide pyrophosphatase/phosphodiesterase 1 OS=Saccharomyces cerevisiae (strain ATCC 20450...	P25353|NPP1_YEAST	85 kDa		2
887	☑	☆	Thioredoxin-3, mitochondrial OS=Saccharomyces cerevisiae (strain ATCC 204508 / S288c) OX=559292 GN...	P25372|TRX3_YEAST	14 kDa		2
888	☑	☆	Glutaredoxin-1 OS=Saccharomyces cerevisiae (strain ATCC 204508 / S288c) OX=559292 GN=GRX1 PE=1 S...	P25373|GLRX1_YEAST	12 kDa		2

Probability Legend

- over 95%
- 80% to 94%
- 50% to 79%
- 20% to 49%
- 0% to 19%

#	Visible?	Starred?	BioView: 1158 Proteins in 1038 Clusters With 5 Decoys and 22 Hidden	Accession Number	Molecular Weight	Protein Grouping Ambiguity	SrfA_C_CAT
889	☑	☆	Pyruvate dehydrogenase E1 component subunit beta, mitochondrial OS=Ascaris suum OX=6253 PE=1 SV=1	P26269|ODPB_ASCSU	39 kDa		2
890	☑	☆	Replication factor A protein 2 OS=Saccharomyces cerevisiae (strain ATCC 204508 / S288c) OX=559292 GN...	P26754|RFA2_YEAST	30 kDa		2
891	☑	☆	Calcium/calmodulin-dependent protein kinase I OS=Saccharomyces cerevisiae (strain ATCC 204508 / S28...	P27466|KCC1_YEAST	50 kDa		2
892	☑	☆	Monoglyceride lipase OS=Saccharomyces cerevisiae (strain ATCC 204508 / S288c) OX=559292 GN=YJU3 P...	P28321|MGLL_YEAST	36 kDa		2
893	☑	☆	Peptidyl-prolyl cis-trans isomerase FPR2 OS=Saccharomyces cerevisiae (strain ATCC 204508 / S288c) OX...	P32472|FKBP2_YEAST	14 kDa		2
894	☑	☆	Lysine-specific permease OS=Saccharomyces cerevisiae (strain ATCC 204508 / S288c) OX=559292 GN=LY...	P32487|LYP1_YEAST	68 kDa		2
895	☑	☆	Guanosine-diphosphatase OS=Saccharomyces cerevisiae (strain ATCC 204508 / S288c) OX=559292 GN=G...	P32621|GDA1_YEAST	57 kDa		2
896	☑	☆	Cobalt uptake protein COT1 OS=Saccharomyces cerevisiae (strain ATCC 204508 / S288c) OX=559292 GN=...	P32798|COT1_YEAST	48 kDa		2
897	☑	☆	37S ribosomal protein S5, mitochondrial OS=Saccharomyces cerevisiae (strain ATCC 204508 / S288c) OX=...	P33759|RT05_YEAST	35 kDa		2
898	☑	☆	Uncharacterized protein YKL187C OS=Saccharomyces cerevisiae (strain ATCC 204508 / S288c) OX=55929...	P34231|YKS7_YEAST	81 kDa		2
899	☑	☆	Mannan endo-1,6-alpha-mannosidase DCW1 OS=Saccharomyces cerevisiae (strain ATCC 204508 / S288c) ...	P36091|DCW1_YEAST	50 kDa		2
900	☑	☆	Sedoheptulose 1,7-bisphosphatase OS=Saccharomyces cerevisiae (strain ATCC 204508 / S288c) OX=5592...	P36136|SHB17_YEAST	31 kDa		2
901	☑	☆	Presequence translocated-associated motor subunit PAM17, mitochondrial OS=Saccharomyces cerevisiae...	P36147|PAM17_YEAST	22 kDa		2
902	☑	☆	V-type proton ATPase subunit a, Golgi isoform OS=Saccharomyces cerevisiae (strain ATCC 204508 / S288c...	P37296|STV1_YEAST	102 kDa		2
903	☑	☆	Valine/tyrosine/tryptophan amino-acid permease 1 OS=Saccharomyces cerevisiae (strain ATCC 204508 / ...	P38085|TAT1_YEAST	69 kDa		2
904	☑	☆	Uridine permease OS=Saccharomyces cerevisiae (strain ATCC 204508 / S288c) OX=559292 GN=FUI1 PE=1...	P38196|FUI1_YEAST	72 kDa		2
905	☑	☆	U6 snRNA-associated Sm-like protein LSm2 OS=Saccharomyces cerevisiae (strain ATCC 204508 / S288c) ...	P38203|LSM2_YEAST	11 kDa		2
906	☑	☆	Sec sixty-one protein homolog OS=Saccharomyces cerevisiae (strain ATCC 204508 / S288c) OX=559292 G...	P38353|SSH1_YEAST	53 kDa		2
907	☑	☆	Signal recognition particle subunit SRP68 OS=Saccharomyces cerevisiae (strain ATCC 204508 / S288c) OX...	P38687|SRP68_YEAST	69 kDa		2
908	☑	☆	Putative aldehyde dehydrogenase-like protein YHR039C OS=Saccharomyces cerevisiae (strain ATCC 2045...	P38694|MSC7_YEAST	71 kDa		2
909	☑	☆	Restriction of telomere capping protein 3 OS=Saccharomyces cerevisiae (strain ATCC 204508 / S288c) OX...	P38804|SDO1L_YEAST	12 kDa		2
910	☑	☆	Eukaryotic translation initiation factor 1A OS=Saccharomyces cerevisiae (strain ATCC 204508 / S288c) OX...	P38912|IF1A_YEAST	17 kDa		2
911	☑	☆	Cell wall synthesis protein KRE9 OS=Saccharomyces cerevisiae (strain ATCC 204508 / S288c) OX=559292 ...	P39005|KRE9_YEAST	30 kDa		2
912	☑	☆	Mitochondrial import inner membrane translocase subunit TIM17 OS=Saccharomyces cerevisiae (strain A...	P39515|TIM17_YEAST	17 kDa		2
913	☑	☆	ER-derived vesicles protein ERV46 OS=Saccharomyces cerevisiae (strain ATCC 204508 / S288c) OX=55929...	P39727|ERV46_YEAST	46 kDa		2
914	☑	☆	Vacuolar-sorting protein SNF7 OS=Saccharomyces cerevisiae (strain ATCC 204508 / S288c) OX=559292 G...	P39929|SNF7_YEAST	27 kDa		2
915	☑	☆	40S ribosomal protein S26-A OS=Saccharomyces cerevisiae (strain ATCC 204508 / S288c) OX=559292 GN...	P39938|RS26A_YEAST (+1)	14 kDa		2
916	☑	☆	U6 snRNA-associated Sm-like protein LSm5 OS=Saccharomyces cerevisiae (strain ATCC 204508 / S288c) ...	P40089|LSM5_YEAST	10 kDa		2
917	☑	☆	Uncharacterized mitochondrial membrane protein FMP10 OS=Saccharomyces cerevisiae (strain ATCC 204...	P40098|FMP10_YEAST	28 kDa		2
918	☑	☆	Coatomer subunit epsilon OS=Saccharomyces cerevisiae (strain ATCC 204508 / S288c) OX=559292 GN=SE...	P40509|COPE_YEAST	34 kDa		2
919	☑	☆	ER membrane protein complex subunit 5 OS=Saccharomyces cerevisiae (strain ATCC 204508 / S288c) OX...	P40540|EMC5_YEAST	16 kDa		2
920	☑	☆	Peroxiredoxin DOT5 OS=Saccharomyces cerevisiae (strain ATCC 204508 / S288c) OX=559292 GN=DOT5 PE...	P40553|DOT5_YEAST	24 kDa		2
921	☑	☆	Ribosome biogenesis protein RLP7 OS=Saccharomyces cerevisiae (strain ATCC 204508 / S288c) OX=5592...	P40693|RLP7_YEAST	37 kDa		2
922	☑	☆	Glutathione reductase OS=Saccharomyces cerevisiae (strain ATCC 204508 / S288c) OX=559292 GN=GLR1 ...	P41921|GSHR_YEAST	53 kDa		2
923	☑	☆	Protein KRI1 OS=Saccharomyces cerevisiae (strain ATCC 204508 / S288c) OX=559292 GN=KRI1 PE=1 SV=1	P42846|KRI1_YEAST	69 kDa		2
924	☑	☆	Citrate synthase 3, mitochondrial OS=Saccharomyces cerevisiae (strain ATCC 204508 / S288c) OX=55929...	P43635|CISY3_YEAST	54 kDa		2
925	☑	☆	GPN-loop GTPase 1 OS=Saccharomyces cerevisiae (strain ATCC 204508 / S288c) OX=559292 GN=NPA3 PE...	P47122|GPN1_YEAST	43 kDa		2

Probability Legend: over 95% | 80% to 94% | 50% to 79% | 20% to 49% | 0% to 19%

#	Visible?	Starred?	BioView: 1158 Proteins in 1038 Clusters With 5 Decoys and 22 Hidden	Accession Number	Molecular Weight	Protein Grouping Ambiguity	SrfA_C_CAT
926	☑	☆	ER membrane protein complex subunit 2 OS=Saccharomyces cerevisiae (strain ATCC 204508 / S288c) OX...	P47133|EMC2_YEAST	34 kDa		2
927	☑	☆	37S ribosomal protein S7, mitochondrial OS=Saccharomyces cerevisiae (strain ATCC 204508 / S288c) OX=...	P47150|RT07_YEAST	28 kDa		2
928	☑	☆	Protein CCC1 OS=Saccharomyces cerevisiae (strain ATCC 204508 / S288c) OX=559292 GN=CCC1 PE=1 SV=1	P47818|CCC1_YEAST	34 kDa		2
929	☑	☆	Dolichyl-diphosphooligosaccharide--protein glycosyltransferase subunit 3 OS=Saccharomyces cerevisiae ...	P48439|OST3_YEAST	39 kDa		2
930	☑	☆	MICOS subunit MIC26 OS=Saccharomyces cerevisiae (strain ATCC 204508 / S288c) OX=559292 GN=MIC26...	P50087|MIC26_YEAST	27 kDa		2
931	☑	☆	ER membrane protein complex subunit 4 OS=Saccharomyces cerevisiae (strain ATCC 204508 / S288c) OX...	P53073|EMC4_YEAST	21 kDa		2
932	☑	☆	Zinc finger protein ZPR1 OS=Saccharomyces cerevisiae (strain ATCC 204508 / S288c) OX=559292 GN=ZPR...	P53303|ZPR1_YEAST	55 kDa		2
933	☑	☆	Coatomer subunit zeta OS=Saccharomyces cerevisiae (strain ATCC 204508 / S288c) OX=559292 GN=RET3...	P53600|COPZ_YEAST	22 kDa		2
934	☑	☆	UPF0674 endoplasmic reticulum membrane protein YNR021W OS=Saccharomyces cerevisiae (strain ATCC ...	P53723|YN8B_YEAST	47 kDa		2
935	☑	☆	Nucleolar GTP-binding protein 2 OS=Saccharomyces cerevisiae (strain ATCC 204508 / S288c) OX=559292 ...	P53742|NOG2_YEAST	55 kDa		2
936	☑	☆	Uncharacterized oxidoreductase YNL181W OS=Saccharomyces cerevisiae (strain ATCC 204508 / S288c) O...	P53878|YNS1_YEAST	47 kDa		2
937	☑	☆	Protein NSG2 OS=Saccharomyces cerevisiae (strain ATCC 204508 / S288c) OX=559292 GN=NSG2 PE=1 SV=1	P53898|NSG2_YEAST	34 kDa		2
938	☑	☆	GTP-binding protein RHO3 OS=Saccharomyces cerevisiae (strain ATCC 204508 / S288c) OX=559292 GN=R...	Q00245|RHO3_YEAST	25 kDa		2
939	☑	☆	Fatty acid-binding protein 5 OS=Homo sapiens OX=9606 GN=FABP5 PE=1 SV=3	Q01469|FABP5_HUMAN	15 kDa		2
940	☑	☆	Cytochrome c oxidase subunit 12, mitochondrial OS=Saccharomyces cerevisiae (strain ATCC 204508 / S28...	Q01519|COX12_YEAST	10 kDa		2
941	☑	☆	Dolichyl-diphosphooligosaccharide--protein glycosyltransferase subunit SWP1 OS=Saccharomyces cerevi...	Q02795|OSTD_YEAST	32 kDa		2
942	☑	☆	t-SNARE VTI1 OS=Saccharomyces cerevisiae (strain ATCC 204508 / S288c) OX=559292 GN=VTI1 PE=1 SV=3	Q04338|VTI1_YEAST	25 kDa		2
943	☑	☆	FACT complex subunit POB3 OS=Saccharomyces cerevisiae (strain ATCC 204508 / S288c) OX=559292 GN=...	Q04636|POB3_YEAST	63 kDa		2
944	☑	☆	Transaminated amino acid decarboxylase OS=Saccharomyces cerevisiae (strain ATCC 204508 / S288c) OX...	Q06408|ARO10_YEAST	71 kDa		2
945	☑	☆	Homocysteine S-methyltransferase 2 OS=Saccharomyces cerevisiae (strain ATCC 204508 / S288c) OX=55...	Q08985|SAM4_YEAST	37 kDa		2
946	☑	☆	Ubiquitin-like protein SMT3 OS=Saccharomyces cerevisiae (strain ATCC 204508 / S288c) OX=559292 GN=...	Q12306|SMT3_YEAST	12 kDa		2
947	☑	☆	Phosphatidylglycerol/phosphatidylinositol transfer protein OS=Saccharomyces cerevisiae (strain ATCC 20...	Q12408|NPC2_YEAST	19 kDa		2
948	☑	☆	Polyamine N-acetyltransferase 1 OS=Saccharomyces cerevisiae (strain ATCC 204508 / S288c) OX=559292...	Q12447|PAA1_YEAST	22 kDa		2
949	☑	☆	Pheromone-regulated membrane protein 4 OS=Saccharomyces cerevisiae (strain ATCC 204508 / S288c) ...	Q12498|PRM4_YEAST	32 kDa		2
950	☑	☆	Eukaryotic translation initiation factor 6 OS=Saccharomyces cerevisiae (strain ATCC 204508 / S288c) OX=...	Q12522|IF6_YEAST	26 kDa		2
951	☑	☆	Pre-rRNA-processing protein PNO1 OS=Saccharomyces cerevisiae (strain ATCC 204508 / S288c) OX=5592...	Q99216|PNO1_YEAST	30 kDa		2
952	☑	☆	2-deoxyglucose-6-phosphate phosphatase 2 OS=Saccharomyces cerevisiae (strain ATCC 204508 / S288c)...	P38773|DOG2_YEAST (+1)	27 kDa		2
953	☑	☆	54S ribosomal protein L40, mitochondrial OS=Saccharomyces cerevisiae (strain ATCC 204508 / S288c) OX...	P36534|RM40_YEAST	34 kDa		2
954	☑	☆	Plakophilin-1 OS=Bos taurus OX=9913 GN=PKP1 PE=2 SV=1	Q28161|PKP1_BOVIN	80 kDa		2
955	☑	☆	Protein transport protein SEC7 OS=Saccharomyces cerevisiae (strain ATCC 204508 / S288c) OX=559292 G...	P11075|SEC7_YEAST	227 kDa		2
956	☑	☆	Membrane protein PTM1 OS=Saccharomyces cerevisiae (strain YJM789) OX=307796 GN=PTM1 PE=3 SV=1	A6ZZS6|PTM1_YEAS7 (+1)	60 kDa		2
957	☑	☆	Mitochondrial import inner membrane translocase subunit TIM9 OS=Saccharomyces cerevisiae (strain AT...	O74700|TIM9_YEAST	10 kDa		2
958	☑	☆	Cytochrome b OS=Saccharomyces cerevisiae (strain ATCC 204508 / S288c) OX=559292 GN=COB PE=1 SV=3	P00163|CYB_YEAST (+1)	44 kDa		2
959	☑	☆	Cytochrome c oxidase subunit 9, mitochondrial OS=Saccharomyces cerevisiae (strain ATCC 204508 / S288...	P07255|COX9_YEAST	7 kDa		2
960	☑	☆	Mitochondrial-processing peptidase subunit alpha OS=Saccharomyces cerevisiae (strain ATCC 204508 / S...	P11914|MPPA_YEAST	53 kDa		2
961	☑	☆	Nucleoporin NSP1 OS=Saccharomyces cerevisiae (strain ATCC 204508 / S288c) OX=559292 GN=NSP1 PE=1...	P14907|NSP1_YEAST	87 kDa		2
962	☑	☆	Protein RER1 OS=Saccharomyces cerevisiae (strain ATCC 204508 / S288c) OX=559292 GN=RER1 PE=1 SV=2	P25560|RER1_YEAST (+1)	22 kDa		2

Probability Legend

- over 95%
- 80% to 94%
- 50% to 79%
- 20% to 49%
- 0% to 19%

#	Visible?	Starred?	BioView: 1158 Proteins in 1038 Clusters With 5 Decoys and 22 Hidden	Accession Number	Molecular Weight	Protein Grouping Ambiguity	SrfA_C_CAT
963	☑	☆	Leu/Val/Ile amino-acid permease OS=Saccharomyces cerevisiae (strain ATCC 204508 / S288c) OX=55929...	P38084|BAP2_YEAST	68 kDa		2
964	☑	☆	Phosphatidate cytidylyltransferase OS=Saccharomyces cerevisiae (strain ATCC 204508 / S288c) OX=5592...	P38221|CDS1_YEAST	52 kDa		2
965	☑	☆	Ergosterol biosynthetic protein 28 OS=Saccharomyces cerevisiae (strain ATCC 204508 / S288c) OX=55929...	P40030|ERG28_YEAST	17 kDa		2
966	☑	☆	DNA damage-inducible protein 1 OS=Saccharomyces cerevisiae (strain ATCC 204508 / S288c) OX=559292 ...	P40087|DDI1_YEAST	47 kDa		2
967	☑	☆	MYG1 protein YER156C OS=Saccharomyces cerevisiae (strain ATCC 204508 / S288c) OX=559292 GN=YER1...	P40093|YEY6_YEAST	38 kDa		2
968	☑	☆	HSP70 co-chaperone SNL1 OS=Saccharomyces cerevisiae (strain ATCC 204508 / S288c) OX=559292 GN=S...	P40548|SNL1_YEAST	18 kDa		2
969	☑	☆	40S ribosomal protein S29-A OS=Saccharomyces cerevisiae (strain ATCC 204508 / S288c) OX=559292 GN...	P41057|RS29A_YEAST	7 kDa		2
970	☑	☆	Ribulose-phosphate 3-epimerase OS=Saccharomyces cerevisiae (strain ATCC 204508 / S288c) OX=55929...	P46969|RPE_YEAST	26 kDa		2
971	☑	☆	Suppressor of kinetochore protein 1 OS=Saccharomyces cerevisiae (strain ATCC 204508 / S288c) OX=559...	P52286|SKP1_YEAST	22 kDa		2
972	☑	☆	Uncharacterized membrane protein YML131W OS=Saccharomyces cerevisiae (strain ATCC 204508 / S288...	Q03102|YMN1_YEAST	40 kDa		2
973	☑	☆	Eukaryotic translation initiation factor 3 subunit J OS=Saccharomyces cerevisiae (strain ATCC 204508 / S2...	Q05775|EIF3J_YEAST	30 kDa		2
974	☑	☆	RuvB-like protein 2 OS=Saccharomyces cerevisiae (strain ATCC 204508 / S288c) OX=559292 GN=RVB2 PE...	Q12464|RUVB2_YEAST	52 kDa		2
975	☑	☆	Plectin OS=Mus musculus OX=10090 GN=Plec PE=1 SV=3	Q9QXS1|PLEC_MOUSE	534 kDa	★	3
976	☑	☆	Translation initiation factor eIF-2B subunit epsilon OS=Saccharomyces cerevisiae (strain ATCC 204508 / S...	P32501|EI2BE_YEAST	81 kDa		2
977	☑	☆	DNA-directed RNA polymerases I, II, and III subunit RPABC1 OS=Saccharomyces cerevisiae (strain ATCC 2...	P20434|RPAB1_YEAST	25 kDa		2
978	☑	☆	Protein YOP1 OS=Saccharomyces cerevisiae (strain ATCC 204508 / S288c) OX=559292 GN=YOP1 PE=1 SV=3	Q12402|YOP1_YEAST	20 kDa		2
979	☑	☆	13 kDa ribonucleoprotein-associated protein OS=Saccharomyces cerevisiae (strain ATCC 204508 / S288c)...	P39990|SNU13_YEAST	14 kDa		3
980	☑	☆	Siderophore iron transporter 1 OS=Saccharomyces cerevisiae (strain ATCC 204508 / S288c) OX=559292 G...	P39980|SIT1_YEAST	71 kDa		3
⊞ 981	☑		Cluster of H/ACA ribonucleoprotein complex subunit GAR1 OS=Chaetomium globosum (strain ATCC 6205 / ...	Q2HH48|GAR1_CHAGB	21 kDa	★	2
982	☑	☆	Malate dehydrogenase, cytoplasmic OS=Saccharomyces cerevisiae (strain ATCC 204508 / S288c) OX=559...	P22133|MDHC_YEAST	41 kDa		2
983	☑	☆	Sideroflexin FSF1 OS=Saccharomyces cerevisiae (strain ATCC 204508 / S288c) OX=559292 GN=FSF1 PE=1 ...	Q12029|FSF1_YEAST	35 kDa		2
984	☑	☆	Methionine aminopeptidase 2 OS=Saccharomyces cerevisiae (strain YJM789) OX=307796 GN=MAP2 PE=3 ...	A6ZKL2|MAP2_YEAS7 (+5)	48 kDa		2
985	☑	☆	Uncharacterized vacuolar membrane protein SCY_4679 OS=Saccharomyces cerevisiae (strain YJM789) O...	A6ZRW8|YNL5_YEAS7 (+1)	74 kDa		2
986	☑	☆	Pyrroline-5-carboxylate reductase OS=Saccharomyces cerevisiae (strain ATCC 204508 / S288c) OX=5592...	P32263|P5CR_YEAST	30 kDa		2
987	☑	☆	Hsp90 co-chaperone HCH1 OS=Saccharomyces cerevisiae (strain ATCC 204508 / S288c) OX=559292 GN=H...	P53834|HCH1_YEAST	17 kDa		2
988	☑	☆	Pumilio homology domain family member 6 OS=Saccharomyces cerevisiae (strain ATCC 204508 / S288c) ...	Q04373|PUF6_YEAST	75 kDa		2
989	☑	☆	Golgi to ER traffic protein 1 OS=Saccharomyces cerevisiae (strain YJM789) OX=307796 GN=GET1 PE=3 SV...	A6ZUX3|GET1_YEAS7 (+3)	27 kDa		2
990	☑	☆	Carboxypeptidase Y inhibitor OS=Saccharomyces cerevisiae (strain ATCC 204508 / S288c) OX=559292 GN...	P14306|CPYI_YEAST	24 kDa		2
991	☑	☆	Proteasome subunit beta type-3 OS=Saccharomyces cerevisiae (strain ATCC 204508 / S288c) OX=559292...	P25451|PSB3_YEAST	23 kDa		2
992	☑	☆	Glyoxylate reductase 1 OS=Saccharomyces cerevisiae (strain ATCC 204508 / S288c) OX=559292 GN=GOR...	P53839|GOR1_YEAST	39 kDa		2
993	☑	☆	UPF0495 protein YPR010C-A OS=Saccharomyces cerevisiae (strain ATCC 204508 / S288c) OX=559292 GN...	A5Z2X5|YP010_YEAST	8 kDa		2
994	☑	☆	Protein ATP11, mitochondrial OS=Saccharomyces cerevisiae (strain ATCC 204508 / S288c) OX=559292 GN...	P32453|ATP11_YEAST	37 kDa		2
995	☑	☆	Succinate dehydrogenase [ubiquinone] cytochrome b subunit, mitochondrial OS=Saccharomyces cerevisi...	P33421|SDH3_YEAST	22 kDa		2
996	☑	☆	FACT complex subunit SPT16 OS=Saccharomyces cerevisiae (strain ATCC 204508 / S288c) OX=559292 GN...	P32558|SPT16_YEAST	119 kDa		2
997	☑	☆	ATP synthase subunit K, mitochondrial OS=Saccharomyces cerevisiae (strain ATCC 204508 / S288c) OX=5...	P81451|ATP19_YEAST	8 kDa		2
998	☑	☆	Canalicular multispecific organic anion transporter 1 OS=Rattus norvegicus OX=10116 GN=Abcc2 PE=1 SV...	Q63120|MRP2_RAT	173 kDa		2
999	☑	☆	Protein-glutamine gamma-glutamyltransferase E OS=Homo sapiens OX=9606 GN=TGM3 PE=1 SV=4	Q08188|TGM3_HUMAN	77 kDa		2

Probability Legend

- over 95%
- 80% to 94%
- 50% to 79%
- 20% to 49%
- 0% to 19%

BioView:
1158 Proteins in 1038 Clusters
With 5 Decoys and 22 Hidden

#	Visible?	Starred?	BioView	Accession Number	Molecular Weight	Protein Grouping Ambiguity	SrfA_C_CAT
1000	☑	☆	Thioredoxin OS=Homo sapiens OX=9606 GN=TXN PE=1 SV=3	P10599|THIO_HUMAN (+1)	12 kDa		2
1001	☑	☆	Mitochondrial escape protein 2 OS=Candida glabrata (strain ATCC 2001 / CBS 138 / JCM 3761 / NBRC 0622 ...	Q6FIK2|YME2_CANGA	98 kDa		2
1002	☑	☆	Glycogen [starch] synthase isoform 1 OS=Saccharomyces cerevisiae (strain ATCC 204508 / S288c) OX=55...	P23337|GYS1_YEAST	81 kDa		2
1003	☑	☆	Serine/threonine-protein kinase SMG1 OS=Mus musculus OX=10090 GN=Smg1 PE=1 SV=3	Q8BKX6|SMG1_MOUSE	410 kDa		2
1004	☑	☆	Arginine--tRNA ligase OS=Photobacterium profundum (strain SS9) OX=298386 GN=argS PE=3 SV=1	#DECOY#Q6LTA1|SYR_PHOPR..	64 kDa		2
1005	☑	☆	Glycine cleavage system H protein 3 OS=Aquifex aeolicus (strain VF5) OX=224324 GN=gcvH3 PE=3 SV=1	O67080|GCSH3_AQUAE	18 kDa		2
1006	☑	☆	Phosphoglucomutase, chloroplastic OS=Brassica napus OX=3708 GN=PGMP PE=2 SV=1	#DECOY#Q9SMM0|PGMP_BRA..	69 kDa		2
1007	☑	☆	Vesicular-fusion protein SEC18 OS=Saccharomyces cerevisiae (strain ATCC 204508 / S288c) OX=559292 G...	P18759|SEC18_YEAST	84 kDa		2
1008	☑	☆	Serine/threonine-protein phosphatase PP1-1 OS=Saccharomyces cerevisiae (strain ATCC 204508 / S288c...	P20604|PP11_YEAST	36 kDa		2
1009	☑	☆	Histone H2A OS=Cryptococcus neoformans var. neoformans serotype D (strain JEC21 / ATCC MYA-565) OX...	P0CN98|H2A_CRYNJ (+2)	14 kDa	★	2
1010	☑	☆	CRAL-TRIO domain-containing protein YKL091C OS=Saccharomyces cerevisiae (strain ATCC 204508 / S28...	P33324|YKJ1_YEAST	36 kDa	★	2
1011	☑	☆	T-complex protein 1 subunit gamma OS=Xenopus laevis OX=8355 GN=cct3 PE=2 SV=2	P50143|TCPG_XENLA	61 kDa	★	2
1012	☑	☆	Cell surface mannoprotein MP65 OS=Candida albicans (strain SC5314 / ATCC MYA-2876) OX=237561 GN=...	Q59XX2|MP65_CANAL	39 kDa	★	2
1013	☑	☆	Fructose-bisphosphate aldolase OS=Aspergillus oryzae (strain ATCC 42149 / RIB 40) OX=510516 GN=fbaA...	Q9HGY9|ALF_ASPOR	40 kDa	★	3
1014	☑	☆	60S ribosomal protein L7 OS=Rattus norvegicus OX=10116 GN=Rpl7 PE=1 SV=2	P05426|RL7_RAT (+5)	30 kDa	★	2
1015	☑	☆	Retinol dehydrogenase 14 OS=Mus musculus OX=10090 GN=Rdh14 PE=1 SV=1	Q9ERI6|RDH14_MOUSE (+1)	36 kDa		2
1016	☑	☆	Lanosterol 14-alpha demethylase OS=Saccharomyces cerevisiae (strain ATCC 204508 / S288c) OX=55929...	P10614|CP51_YEAST	61 kDa		2
1017	☑	☆	Transcription elongation factor SPT5 OS=Saccharomyces cerevisiae (strain ATCC 204508 / S288c) OX=559...	P27692|SPT5_YEAST	116 kDa		2
1018	☑	☆	Mitochondrial import inner membrane translocase subunit TIM8 OS=Saccharomyces cerevisiae (strain AT...	P57744|TIM8_YEAST	10 kDa		2
1019	☑	☆	Nucleoporin NUP133 OS=Saccharomyces cerevisiae (strain ATCC 204508 / S288c) OX=559292 GN=NUP133 ...	P36161|NU133_YEAST	133 kDa		2
1020	☑	☆	Hsp90 co-chaperone AHA1 OS=Saccharomyces cerevisiae (strain ATCC 204508 / S288c) OX=559292 GN=A...	Q12449|AHA1_YEAST	39 kDa		2
1021	☑	☆	Lactoylglutathione lyase OS=Saccharomyces cerevisiae (strain ATCC 204508 / S288c) OX=559292 GN=GL...	P50107|LGUL_YEAST	37 kDa		2
1022	☑	☆	Serine/threonine-protein phosphatase PP2A-4 catalytic subunit OS=Oryza sativa subsp. japonica OX=399...	A3C4N5|PP2A4_ORYSJ (+5)	36 kDa		2
1023	☑	☆	U3 small nucleolar RNA-associated protein 20 OS=Saccharomyces cerevisiae (strain ATCC 204508 / S288c...	P35194|UTP20_YEAST	288 kDa		2
1024	☑	☆	Chaperone protein ClpB 2 OS=Streptomyces avermitilis (strain ATCC 31267 / DSM 46492 / JCM 5070 / NBR...	Q826F2|CLPB2_STRAW	98 kDa		2
1025	☑	☆	Transaldolase NQM1 OS=Saccharomyces cerevisiae (strain ATCC 204508 / S288c) OX=559292 GN=NQM1 P...	P53228|TAL2_YEAST	37 kDa	★	2
1026	☑	☆	Malate dehydrogenase 2, mitochondrial OS=Arabidopsis thaliana OX=3702 GN=At3g15020 PE=1 SV=1	Q9LKA3|MDHM2_ARATH	36 kDa	★	2
1027	☑	☆	Probable succinate dehydrogenase [ubiquinone] flavoprotein subunit, mitochondrial OS=Schizosaccharom...	#DECOY#Q9UTJ7|SDHA_SCHP..	70 kDa		2
1028	☑	☆	Titin OS=Homo sapiens OX=9606 GN=TTN PE=1 SV=4	#DECOY#Q8WZ42|TITIN_HUM..	3816 k...		2
1029	☑	☆	Mannose-1-phosphate guanyltransferase 2 OS=Candida glabrata (strain ATCC 2001 / CBS 138 / JCM 3761 ...	#DECOY#Q6FRY2|MPG12_CAN..	40 kDa		2
1030	☑	☆	Protein TIC 214 OS=Aethionema grandiflorum OX=72657 GN=TIC214 PE=3 SV=1	A4QJQ8|TI214_AETGR	214 kDa		2

The site occupancy of SrfA-C_CAT protein

	m/z of unglycosylated	m/z of glycosylated	Peak area of unglycosylated peptide	Peak area of glycosylated peptide	Site occupancy[1]
K.G**N**MTTHANIQGLVK	495.2683^{3+}	495.593^{3+}	122536294	2150390208	95%
K.PIS**N**ASVYILNEQSQLQPFGAIGELCISGMGVSK	1203.27^{3+}	ND[2]		---	~ 0% glycosyaltion
R.HESGDASINAYLV**N**R.T	549.26^{3+}	549.60^{3+}	32904366	957102918	96.7%

Note:

1. Site occupancy= $\frac{peak\ area\ of\ glycosylated\ peptide}{sum\ of\ peak\ area\ of\ glycosylated+unglycosyalted\ peptide} \times 100\%$

2. ND: not detected
